# Comprehensive comparisons of ocular biometry: A network-based big data analysis

**DOI:** 10.1186/s40662-022-00320-3

**Published:** 2022-12-10

**Authors:** Jinjin Yu, Daizong Wen, Jing Zhao, Yiran Wang, Ke Feng, Ting Wan, Giacomo Savini, Colm McAlinden, Xuanqiao Lin, Lingling Niu, Sisi Chen, Qingyi Gao, Rui Ning, Yili Jin, Xingtao Zhou, Jinhai Huang

**Affiliations:** 1 Eye Institute and Department of Ophthalmology, Institute for Medical and Engineering Innovation, Eye & ENT Hospital, Fudan University; NHC Key Laboratory of Myopia (Fudan University), Key Laboratory of Myopia, Chinese Academy of Medical Sciences, No. 19 Baoqing Road, Xuhui District, Shanghai, 200031 China; 2 Quanzhou Aier Eye Hospital, Quanzhou, Fujian China; 3 Shanghai Research Center of Ophthalmology and Optometry, Shanghai, China; 4 Eye Hospital and School of Ophthalmology and Optometry, Wenzhou Medical University, Wenzhou, Zhejiang China; 5 IRCCS G.B. Bietti Foundation, Rome, Italy; 6 Department of Ophthalmology, Singleton Hospital, Swansea Bay University Health Board, Swansea, UK

**Keywords:** Network-based big data analysis, Ocular biometric parameters, Optical biometry, Ultrasound biometry

## Abstract

**Purpose:**

To systematically compare and rank ocular measurements with optical and ultrasound biometers based on big data.

**Methods:**

PubMed, Embase, the Cochrane Library and the US trial registry (www.ClinicalTrial.gov) were used to systematically search trials published up to October 22nd, 2020. We included comparative studies reporting the following parameters measured by at least two devices: axial length (AL), flattest meridian keratometry (Kf), steepest meridian keratometry (Ks), mean keratometry (Km), astigmatism (AST), astigmatism vectors J_0_ and J_45_, anterior chamber depth (ACD), aqueous depth (AQD), central corneal thickness (CCT), corneal diameter (CD) and lens thickness (LT). A network-based big data analysis was conducted using STATA version 13.1.

**Results:**

Across 129 studies involving 17,181 eyes, 12 optical biometers and two ultrasound biometers (with both contact and immersion techniques) were identified. A network meta-analysis for AL and ACD measurements found that statistically significant differences existed when contact ultrasound biometry was compared with the optical biometers. There were no statistically significant differences among the four swept-source optical coherence tomography (SS-OCT) based devices (IOLMaster 700, OA-2000, Argos and ANTERION). As for Ks, Km and CD, statistically significant differences were found when the Pentacam AXL was compared with the IOLMaster and IOLMaster 500. There were statistically significant differences for CCT when the OA-2000 was compared to Pentacam AXL, IOLMaster 700, Lenstar, AL-Scan and Galilei G6.

**Conclusion:**

For AL and ACD, contact ultrasound biometry obtains the lower values compared with optical biometers. The Pentacam AXL achieves the lowest values for keratometry and CD. The smallest value for CCT measurement is found with the OA-2000.

**Supplementary Information:**

The online version contains supplementary material available at 10.1186/s40662-022-00320-3.

## Background

Precise measurements of ocular biometric parameters are extremely important in the practice of ophthalmology and ophthalmic surgery. These measurements mainly include axial length (AL), anterior chamber depth (ACD), aqueous depth (AQD), keratometry, central corneal thickness (CCT), corneal diameter (CD, also known as white-to-white, WTW) and lens thickness (LT) [1]. AL is an essential parameter for calculating intraocular lens (IOL) power in cataract surgery [2] and monitoring the progression of myopia. ACD and AQD can be used to assess angle closure glaucoma, monitor anterior segment changes during accommodation and select anterior chamber phakic IOLs. Keratometry is used to calculate the IOL power and for other purposes (e.g., the diagnosis and grading of keratoconus or contact lens fitting). CCT is utilized when considering patients for refractive surgery [3] to reduce the risk of postoperative ectasia. In order to select the most appropriately sized IOL to be placed in the anterior chamber, an accurate measurement of the CD is necessary [4, 5]. LT influences the depth of the anterior chamber and can explain the cause and mechanism of glaucoma. It also influences the effective position of the IOL and can be a research topic exploring the pathogenesis and treatment of presbyopia [6].

For more than half a century, A-mode ultrasound (A-scan) has been the historic standard measurement of AL [7]. The ultrasonic technique has some disadvantages, mainly due to its contact with the cornea. Further, the applanation technique results in AL measurements 0.1 to 0.3 mm shorter than those by the immersion technique [8]. Recently, the continuous advances in non-contact, multi-parameter integrated measurement optical devices using different techniques have opened new doors in ocular anterior segment imaging and measurement. Optical biometers include the IOLMaster (Carl Zeiss Meditec AG, Jena, Germany), AL-Scan (Nidek Co. Ltd., Gamagori, Japan) and OA-1000 (Tomey, Nagoya, Japan), which are based on partial coherence interferometry (PCI) [9]; Pentacam AXL (Oculus, Wetzlar, Germany) and Galiei G6 (Ziemer, Port, Switzerland), which add a Scheimpflug camera to PCI; Lenstar LS900 (Haag-Streit, Koniz, Switzerland), which is based on optical low-coherence reflectometry (OLCR); Aladdin (Topcon Europe, Visia Imaging, San Giovanni Valdarno, Arezzo, Italy), which is based on optical low-coherence interferometry (OLCI); and IOLMaster 700 (Carl Zeiss Meditec AG, Jena, Germany), OA-2000 (Tomey, Nagoya, Japan), Argos (Movu, Santa Clara, CA), and ANTERION (Heidelberg Engineering GmbH, Heidelberg, Germany), which are based on swept-source optical coherence tomography (SS-OCT).

Many clinical studies have compared these instruments to verify the agreement of their measurements [10–18]. However, there is no definite conclusion about the comparison of all instruments as a single comparative study. In addition, there is no study that compared all instruments at the same time. The purpose of this network-based big data analysis is to systematically review the existing evidence and compare the measurement differences among all optical and ultrasound biometers as well as to guide clinical decisions.

## Methods

This systematic review complies with the preferred reporting items for systematic reviews and meta-analyses (PRISMA) network meta-analysis extension statement [19].

### Search methods

A systematic literature review was conducted using PubMed, Embase, the Cochrane Library and the US trial registry (www.ClinicalTrial.gov) published up to 22nd, October 2020. The full search strategies are shown in Additional file 1: Appendix I. We also manually examined the reference lists of clinical trials, related meta-analyses and systematic reviews to identify relevant studies.

### Eligibility criteria

Trials were included if they met the following criteria: (1) design: comparative study; (2) treated population: all population whether or not with eye diseases; (3) measurements: eyes were measured with A-scan (contact ultrasound and immersion ultrasound), IOLMaster, IOLMaster 500, AL-Scan, OA-1000, Galilei G6, Pentacam AXL, Lenstar, Aladdin, IOLMaster 700, OA-2000, Argos, or ANTERION; (4) comparisons: two or more measuring instruments (as listed above); (5) at least one of the following outcomes: AL, keratometry in the flattest meridian (Kf), keratometry in the steepest meridian (Ks), mean keratometry [Km = (Kf + Ks)/2], astigmatism (AST), astigmatism vectors J_0_ (J_0_ = [− (Ks − Kf)/2 × cos(2 × axis)]), J_45_ (J_45_ = [− (Ks − Kf)/2 × sin(2 × axis)]), ACD, AQD, CCT, LT, and CD (or WTW); (6) measurements acquired by the same operator between two or more devices. We excluded papers that contained a small sample size (less than 10) or contained obviously wrong values of the included outcome parameters, such as Ks higher than Kf. When titles or abstracts were ambiguous, the full text was reviewed for eligibility.

### Outcome measurements

The following parameters were assessed in this review: AL (mm), Kf (D), Ks (D), Km (D), AST (D), J_0_ (D), J_45_ (D), ACD (mm), AQD (mm), CCT (μm), CD (mm), and LT (mm). Original parameters were obtained from the articles as far as possible and parameters that could not be obtained were calculated if possible.

### Study selection and data extraction

Screening was performed by two independent investigators (YW, TW). They retrieved full-text articles that appeared relevant after reviewing the titles and abstracts. They independently assessed full-text articles for final eligibility. Any discrepancies were resolved by focused discussion or consultation with an additional investigator (JY). Two investigators (YW, TW) independently extracted information into an electronic database, including the author, the publication time, outcomes, and quantitative results for treatment effects. For data that were missing or could not be directly obtained, we contacted the authors of the trial reports or used GetData GraphDigitizer 2.24 (http://getdata-graph-digitizer.com) to obtain data from figures.

### Risk of bias assessment

To evaluate the study quality, we used the Quality Assessment of Diagnostic Accuracy Studies (QUADAS) tool for diagnostic studies, which has been strictly evaluated, verified, and recommended by the Cochrane Library. In this method, a total of 14 items were evaluated by "Yes", "No" or "Uncertain". In 2008, according to the opinions of the screening and diagnostic research methodology group of the Cochrane Library, items 3, 8 and 9 of QUADAS were included in the unnecessary evaluation items. Therefore, the remaining 11 items were chosen to assess study quality [20].

### Statistical analysis

STATA statistical software (version 13.0, Stata Corporation, College Station, TX, USA) was used to perform statistical analyses. For binary outcomes, relative effect sizes were calculated as odds ratios (OR) with 95% confidence intervals (CI). For continuous outcomes, relative effect sizes were calculated as weighted mean differences (WMD) with 95% CI. We used visual inspection of the I^2^ statistic [21] (value of 50% or more indicated substantial heterogeneity) to investigate the possibility of statistical heterogeneity. To incorporate indirect comparisons, we performed network meta-analyses using the *mvmeta* command in STATA version 13.1 [22] to estimate pooled ORs and WMD with 95% credible intervals (CrI). We ranked instruments based on the analysis of ranking probabilities and the surface under the cumulative ranking curve (SUCRA) [23]. The SUCRA values, expressed as a percentage, show the relative probability of an instrument to get the maximum parameters’ value. Inconsistency between direct and indirect evidence was assessed by a "node-splitting" approach and the design-by-treatment interaction model assuming consistency throughout the entire network [24]. In order to explore the potential sources of heterogeneity and inconsistency, we performed a subgroup analyses comparing two population groups: healthy vs. diseased (cataract). To avoid the potential influences of age and AL on the measurement results, we limited the subgroups to adults and normal AL range (22 to 26 mm). A funnel plot was used to evaluate publication bias in the results between small and large studies [25]. We also performed additional comparison between the groups according to the principle of the measurements.

## Results

### Literature selection results

**Fig. 1:**
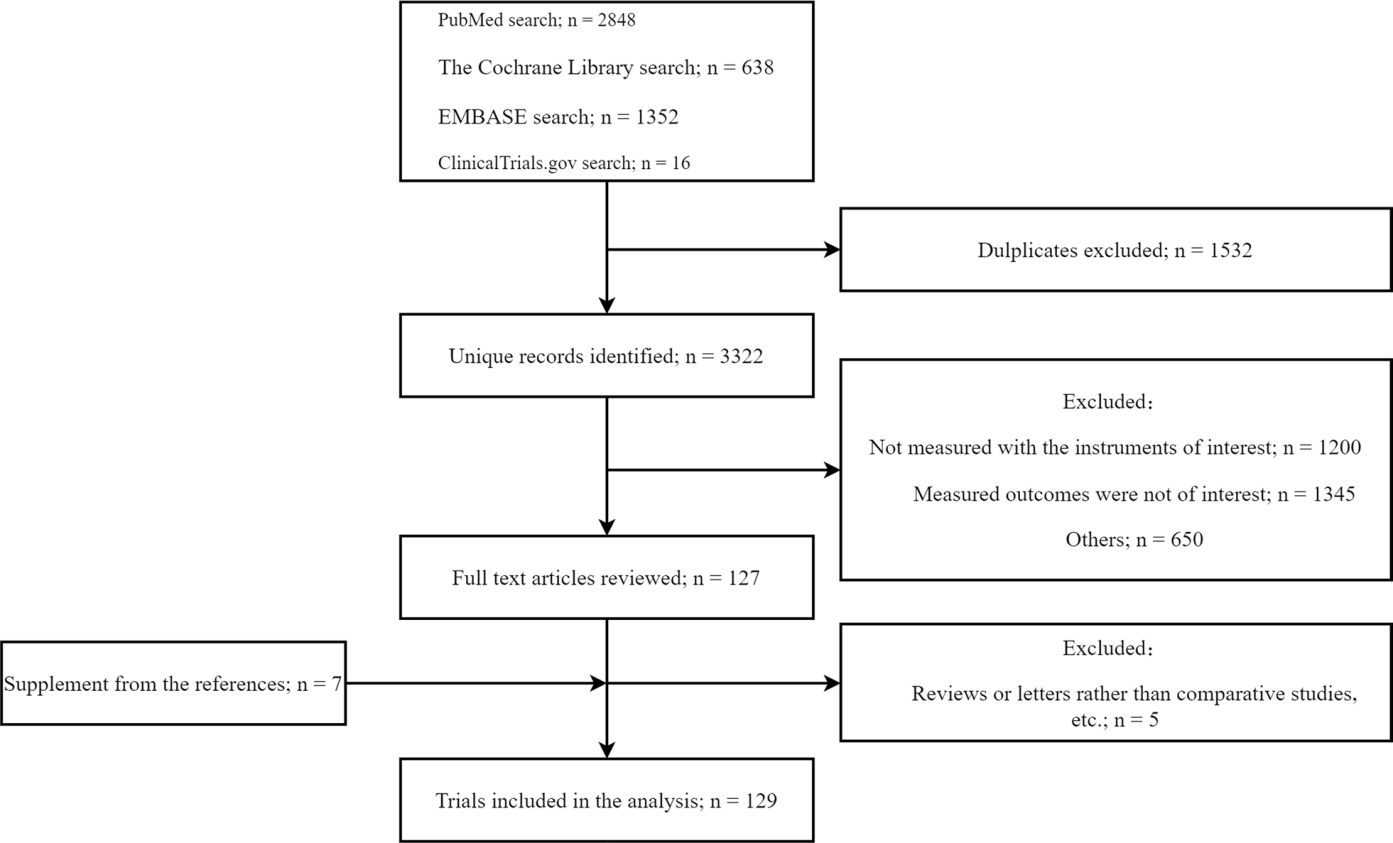
PRISMA flow diagram

This initial literature search yielded 4854 papers. After duplicates were excluded, 3322 studies remained. Of these, 127 studies matched the inclusion criteria, and 7 additional single papers were added from other reference sources listed above. Five of the 134 papers were excluded as they were reviews or letters rather than comparative studies, or they did not include any primary or secondary outcome data. Ultimately, 129 studies met our criteria and were included in our network meta-analysis (Fig. 1).

### Study characteristics and network geometry

**Fig. 2:**
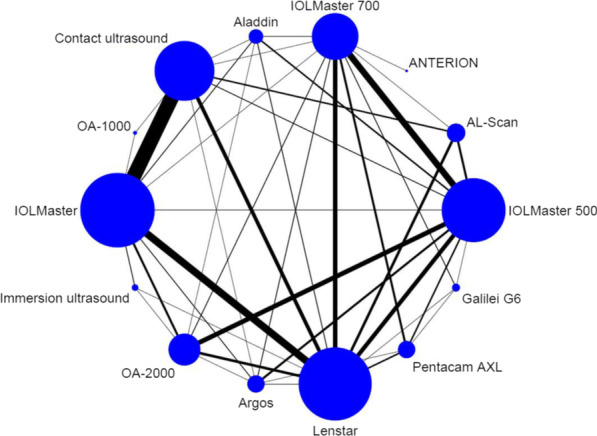
Network of direct comparison for the ophthalmological biometric measurement instruments. Each node represents one instrument. The size of the node is proportional to the number of eyes included in the instrument. The edges represent direct comparisons and the width of the edge is proportional to the number of trials

A summary of all eligible studies published until 2020 is shown in the Additional file 1: Appendix I Table S1. A total of 17,181 eyes were measured by one of 12 optical biometry and ultrasound biometry (with both contact and immersion techniques), with a total of five different techniques (Fig. 2). Almost all trials involved only two devices (92.2%). Among the included 129 trials, 43 (33.3%) recruited healthy or ametropia subjects, 85 (65.9%) recruited participants with cataract, 3 (2.3%) recruited participants who underwent cataract surgery, 1 (0.78%) recruited participants with glaucoma, 1 (0.78%) recruited participants with keratoconus, 1 (0.78%) recruited participants with silicone-filled eyes, and 5 (3.9%) recruited mixed participants.

### Risk of bias assessment results

The risk of bias from the trials included in our study is shown in Additional file 1: Appendix I Table S2. The evaluation of some trials in items 1–5 were “No” or “Not clear”, but all trials gained the full “Yes” for items 6–11. In general, all trials were regarded as high-quality.

### Results of meta-analysis

#### Direct comparisons

**Fig. 3:**
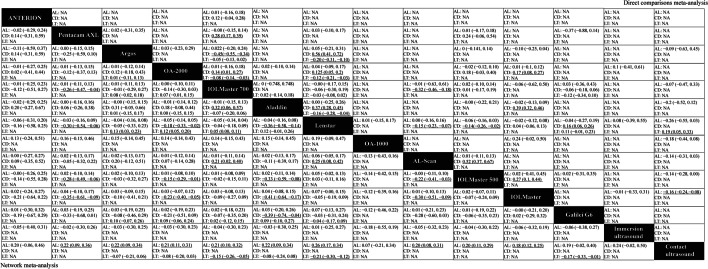
Meta-analysis results comparing all instruments with respect to axial length (AL), corneal diameter (CD), and lens thickness (LT). The upper right shows the direct comparisons meta-analysis between each pair of formulas and the bottom left shows the network meta-analysis between each pair of formulas. Weighted mean differences (95% confidence intervals) are calculated by column. NA, not available. The underline data indicate the statistical significant effect

**Fig. 4:**
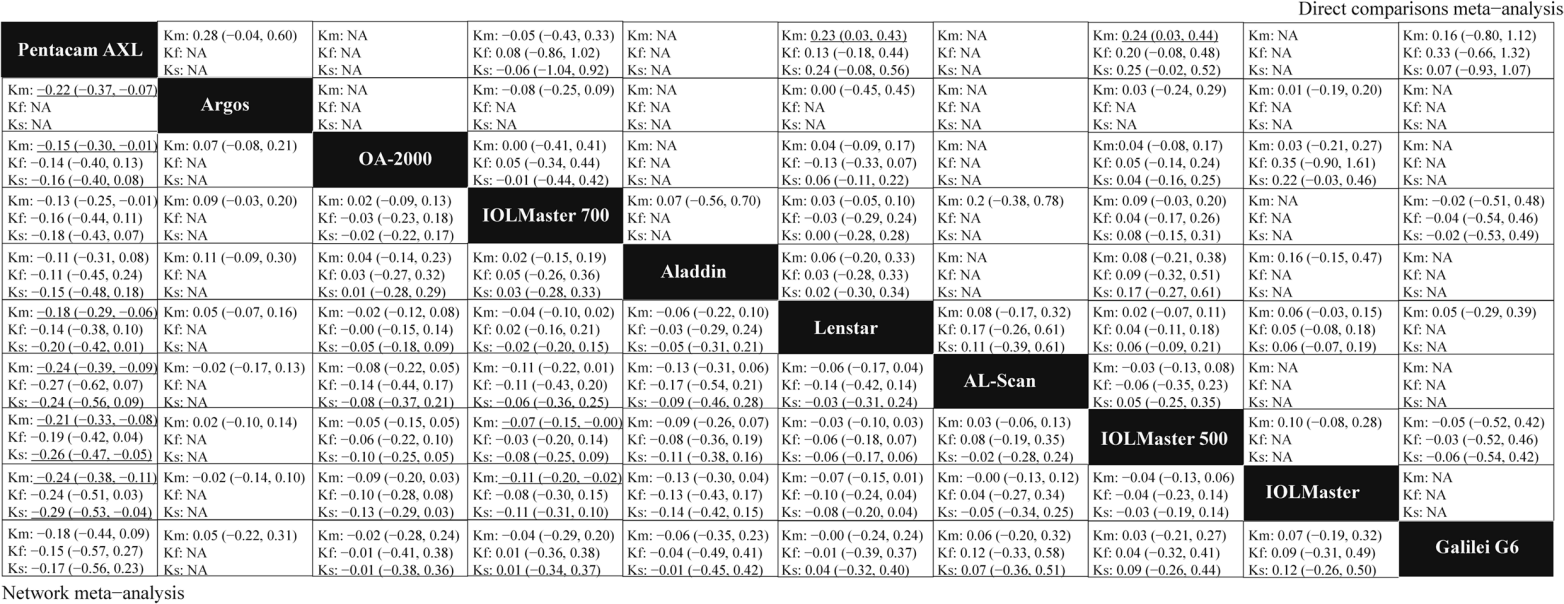
Meta-analysis results comparing all instruments with respect to keratometry. The upper right shows the direct comparisons meta-analysis between each pair of formulas and the bottom left shows the network meta-analysis between each pair of formulas. Weighted mean differences (95% confidence intervals) are calculated by column. Ks, keratometry in the steepest meridian; Kf, keratometry in the flattest meridian; Km, mean keratometry; NA, not available. The underline data indicate the statistical significant effect

**Fig. 5:**
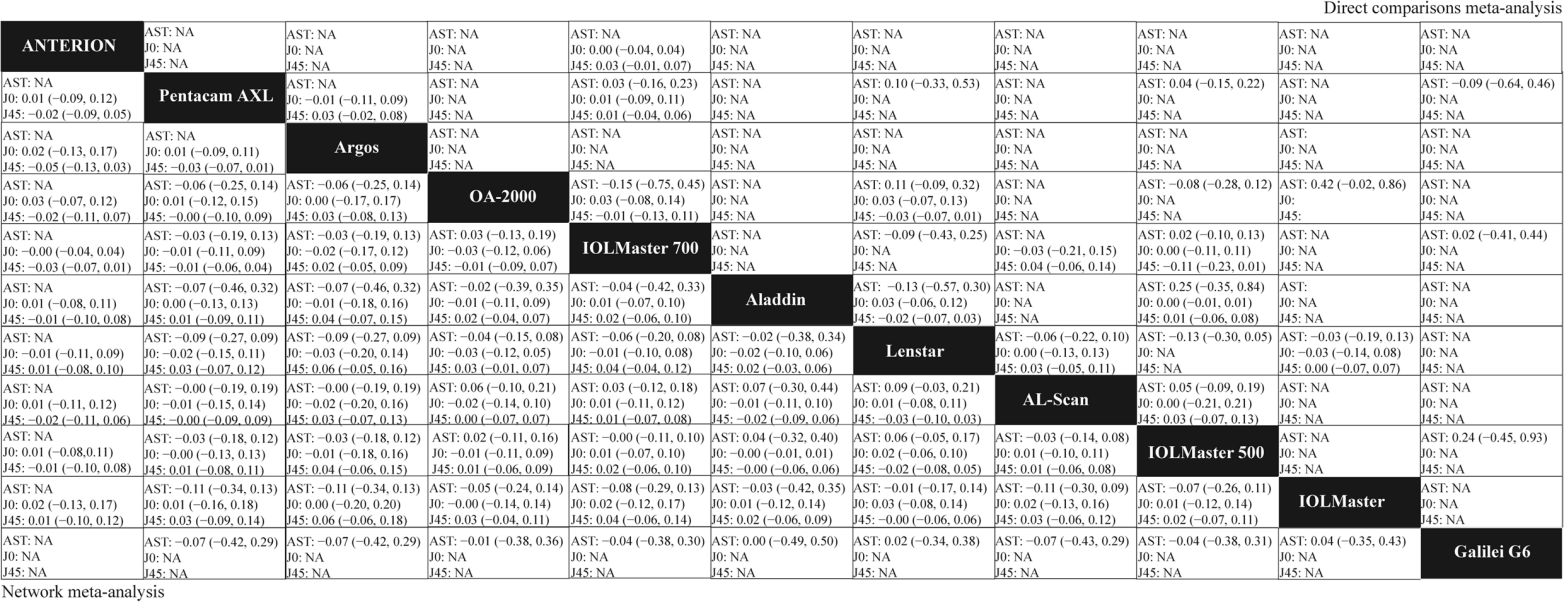
Meta-analysis results comparing all instruments with respect to astigmatism and astigmatism vectors. The upper right shows the direct comparisons meta-analysis between each pair of formulas and the bottom left shows the network meta-analysis between each pair of formulas. Weighted mean differences (95% confidence intervals) are calculated by column. J_0_, anterior corneal power vectors for the cardinal (axes at 90° and 180°) meridians; J_45_, anterior corneal power vectors for the oblique (axes at 45° and 135°) meridians; AST, astigmatism; NA, not available. The underline data indicate the statistical significant effect

**Fig. 6:**
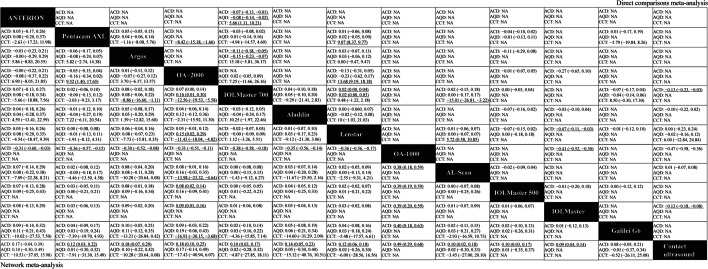
Meta-analysis results comparing all instruments with respect to anterior chamber depth (ACD), aqueous depth (AQD), and central corneal thickness (CCT). The upper right shows the direct comparisons meta-analysis between each pair of formulas and the bottom left shows the network meta-analysis between each pair of formulas. Weighted mean differences (95% confidence intervals) are calculated by column. NA, not available. The underline data indicate the statistical significant effect

Figures 3, 4, 5 and 6 (upper right) and Additional file 1: Appendix I Tables S3–S14 show the direct comparisons between each pair of instruments. In total, 112 studies involving 14 instruments were available for the comparison of the AL. Direct comparisons found that contact ultrasound measured shorter AL when compared with the IOLMaster (WMD =  − 0.159 mm). With regards to measurements of Kf, Ks and astigmatism, there were no statistically significant differences among the various instruments. With respect to the Km, statistically significant differences existed when the Pentacam AXL was compared with the IOLMaster 500 (WMD =  − 0.235 D) and the Lenstar (WMD =  − 0.233 D). When considering the ACD, statistically significant differences existed when contact ultrasound was compared with the IOLMaster (WMD =  − 0.133 mm), the IOLMaster 700 (WMD =  − 0.13 mm), and the OA-1000 (WMD =  − 0.47 mm). Besides, there were statistically significant differences between the IOLMaster 700 and the following devices (WMD from large to small): Argos (WMD =  − 0.113 mm), ANTERION (WMD =  − 0.07 mm), and Lenstar (WMD =  − 0.019 mm). We also found that the Lenstar obtained higher CCT measurements when compared to the OA-2000 (WMD = 13.683 μm) and the Pentacam AXL (WMD = 9.071 μm). There was also a statistical difference between the OA-2000 and the Pentacam AXL (WMD =  − 8.42 μm). As for the measurement of the CD, there were no significant differences among the devices except the Lenstar and the IOLMaster 700, the IOLMaster and the Lenstar, the IOLMaster 500 and the OA-2000, the Galilei G6 and the IOLMaster 700, the IOLMaster 500 and the Pentacam AXL, the IOLMaster 500 and the IOLMaster 700, the IOLMaster 700 and the ANTERION.

#### Combination of direct and indirect comparisons

Figure 3 shows the results of the AL based on network meta-analyses that combine direct and indirect comparisons. As shown, statistically significant differences existed when the contact ultrasound biometry was compared with the following devices (WMD from large to small): Lenstar (WMD =  − 0.26 mm), Pentacam AXL (WMD =  − 0.22 mm), Argos (WMD =  − 0.22 mm), Aladdin (WMD =  − 0.22 mm), OA-2000 (WMD =  − 0.21 mm), IOLMaster 700 (WMD =  − 0.21 mm), AL-Scan (WMD =  − 0.20 mm), IOLMaster 500 (WMD =  − 0.20 mm) and IOLMaster (WMD =  − 0.18 mm). There were no statistically significant difference among the other instruments. As for the ranking results, the instruments were arranged based on the measured value of AL from the maximum to the minimum on the SUCRA values: Lenstar (80.1%), immersion ultrasound (63.3%), Pentacam AXL (60.8%), Argos (60.5%), Aladdin (60.3%), OA-2000 (55.4%), IOLMaster 700 (55.4%), IOLMaster 500 (50.7%), ANTERION (50.2%), AL-Scan (49.9%), Galilei G6 (47.8%), IOLMaster (39.8%), OA-1000 (22.3%), and contact ultrasound (3.5%) (Additional file 2: Fig. S1 and Additional file 1: Appendix I Table S15).

The results of the keratometry findings from the network meta-analyses are shown in Fig. 4. With respect to Kf, there was no statistically significant difference among the optical biometers (*P* > 0.05). The instruments were ranked consulting the maximum to minimum Kf values depending on the SUCRA values: AL-Scan (76.2%), IOLMaster (75.5%), IOLMaster 500 (63%), IOLMaster 700 (52.1%), Galilei G6 (48.8%), OA-2000 (41.9%), Lenstar (41.3%), Aladdin (38.5%), Pentacam AXL (12.8%). As for Ks, only when the Pentacam AXL was compared with the IOLMaster 500 (WMD =  − 0.26 D) and the IOLMaster (WMD =  − 0.29 D), a statistically significant difference existed. The rank results were as follows: IOLMaster (80.1%), IOLMaster 500 (75.3%), AL-Scan (62.8%), Lenstar (53.5%), IOLMaster 700 (46.3%), Galilei G6 (45.5%), Aladdin (40.1%), OA-2000 (37.5%), Pentacam AXL (9%). For Km, statistically significant differences existed when the Pentacam AXL was compared with the following devices (WMD from large to small): AL-Scan (WMD =  − 0.24 D), IOLMaster (WMD =  − 0.24 D), Argos (WMD =  − 0.22 D), IOLMaster 500 (WMD =  − 0.21 D), Lenstar (WMD =  − 0.18 D), OA-2000 (WMD =  − 0.15 D) and IOLMaster 700 (WMD =  − 0.13 D). As for the ranking results, the instruments were ranked as follows: IOLMaster (82.8%), AL-Scan (79.1%), Argos (71.4%), IOLMaster 500 (66.6%), Galilei G6 (52%), Lenstar (49.2%), OA-2000 (40.2%), IOLMaster 700 (28.5%), Aladdin (27.3%), Pentacam AXL (2.9%) (Additional file 2: Fig. S2 and Additional file 1: Appendix I Tables S16–S18).

Figure 5 shows the results for astigmatism. We found that there were no statistically significant differences between any of the studied instruments (*P* > 0.05) considering the AST, J_0_ and J_45_. As for the ranking results, the Lenstar obtained the maximum measured value of AST and J_0_ (70.9%, 65.4%, respectively), and got the minimum measured value of J_45_ (25%) (Additional file 2: Figure S3 and Additional file 1: Appendix I Tables S19–S21).

The results of ACD, AQD and CCT are shown in Fig. 6. When considering the ACD, statistically significant differences existed between contact ultrasound biometry and the following devices (WMD from large to small): OA-1000 (WMD =  − 0.49 mm), Argos (WMD =  − 0.18 mm), OA-2000 (WMD =  − 0.18 mm), Aladdin (WMD =  − 0.14 mm), Pentacam AXL (WMD =  − 0.12 mm), Lenstar (WMD =  − 0.12 mm), IOLMaster 700 (WMD =  − 0.10 mm), AL-Scan (WMD =  − 0.10 mm), IOLMaster 500 (WMD =  − 0.10 mm) and IOLMaster (WMD =  − 0.09 mm). We also observed significant differences between the OA-2000 and the IOLMaster 700 (WMD = 0.07 mm), the OA-2000 and the IOLMaster 500 (WMD = 0.08 mm), the OA-2000 and the IOLMaster (WMD = 0.09 mm). When the OA-1000 was compared with the following devices (WMD from large to small), statistically significant differences were found: Galilei G6 (WMD = 0.40 mm), IOLMaster 500 (WMD = 0.39 mm), IOLMaster (WMD = 0.39 mm), AL-Scan (WMD = 0.38 mm), IOLMaster 700 (WMD = 0.38 mm), Lenstar (WMD = 0.36 mm), Pentacam AXL (WMD = 0.36 mm), Aladdin (WMD = 0.35 mm), OA-2000 (WMD = 0.31 mm), ANTERION (WMD = 0.31 mm), and Argos (WMD = 0.30 mm). There were no significant differences between the other comparisons of the studied instruments.

The rank from the maximum result to the minimum are as follows: OA-1000 (99.8%), OA-2000 (78.5%), Argos (76.7%), ANTERION (64.9%), Aladdin (58.6%), Lenstar (52.1%), Pentacam AXL (51.7%), AL-Scan (37.6%), IOLMaster 700 (36.8%), IOLMaster 500 (32.7%), IOLMaster (29.7%), Galilei G6 (29.6%), contact ultrasound biometry (1.4%) (Additional file 2: Fig. S1). There were significant differences between the OA-2000 with the IOLMaster 700 (WMD = 0.16 mm) and the Lenstar (WMD = 0.15 mm) when taking the AQD into account. According to the SUCRA, the rank from the maximum result to the minimum are as follows: OA-2000 (88.8%), Argos (71.8%), ANTERION (62.9%), Aladdin (53.2%), AL-Scan (41.6%), Lenstar (40.8%), IOLMaster 700 (39.9%), contact ultrasound (39.4%), IOLMaster 500 (39.4%), Pentacam AXL (38%), Galilei G6 (34.3%).

In addition, there were statistically significant differences in measuring CCT when the OA-2000 was compared with the Pentacam AXL (WMD =  − 9.52 μm), IOLMaster 700 (WMD =  − 12.56 μm), Lenstar (WMD =  − 11.43 μm), AL-Scan (WMD =  − 13.98 μm), and Galilei G6 (WMD =  − 16.91 μm). The CCT measuring instruments were ranked depending on the SUCRA values as follows: Galilei G6 (82.1%), AL-Scan (74.6%), contact ultrasound (73.7%), IOLMaster 700 (68.8%), Lenstar (59.5%), Pentacam AXL (49%), ANTERION (38.8%), Argos (22.4%), Aladdin (20.4%), OA-2000 (10.6%) (Additional file 1: Appendix I Tables S22–S24).

Figure 3 shows the results of the CD and LT measurements based on network meta-analyses that combined direct and indirect comparisons. With respect to CD, statistically significant differences existed when the Pentacam AXL was compared with the IOLMaster 700 (WMD =  − 0.26 mm), the Lenstar (WMD =  − 0.30 mm), the IOLMaster 500 (WMD =  − 0.28 mm), and the IOLMaster (WMD =  − 0.35 mm). Statistically significant differences also existed between the OA-2000 and the Lenstar (WMD =  − 0.18 mm), the OA-2000 and the IOLMaster 500 (WMD =  − 0.15 mm), the OA-2000 and the IOLMaster (WMD =  − 0.23 mm), the Aladdin and the IOLMaster 700 (WMD =  − 0.32 mm), the Aladdin and the Lenstar (WMD =  − 0.36 mm), the Aladdin and the IOLMaster 500 (WMD =  − 0.33 mm), the Aladdin and the IOLMaster (WMD =  − 0.41 mmm), the Aladdin and the Galilei G6 (WMD =  − 0.39 mm), the Lenstar and the AL-Scan (WMD = 0.25 mm), the AL-Scan and the IOLMaster 500 (WMD =  − 0.22 mm), and the AL-Scan and the IOLMaster (WMD =  − 0.30 mm). As for the ranking results, the order of CD obtained from the maximum to the minimum based on the SUCRA values are as follows: IOLMaster (84.3%), Galilei G6 (77.4%), Lenstar (74.2%), IOLMaster 500 (66.9%), IOLMaster 700 (63.5%), Argos (60.8%), ANTERION (42.1%), OA-2000 (33.9%), AL-Scan (22.2%), Pentacam AXL (15.2%), Aladdin (9.4%). Considering LT, the ranking results from the maximum to the minimum based on the SUCRA values are as follows: contact ultrasound (94.1%), Argos (68.8%), OA-2000 (63.7%), Aladdin (61%), IOLMaster 700 (30.2%), Galilei G6 (26.3%), Lenstar (5.7%) (Additional file 2: Figures S1–S2 and Additional file 1: Appendix I Tables S25–S26).

#### Inconsistency

Node-splitting analysis between contact ultrasound biometry and the Lenstar for closed-loop comparisons in terms of AL showed significant inconsistency (*P* < 0.05). Similar results included: the Lenstar and the OA-2000 for Kf, the IOLMaster and the Lenstar for Kf and AST, the IOLMaster and contact ultrasound biometry for ACD, the Lenstar and contact ultrasound biometry for ACD, the IOLMaster and the OA-2000 for ACD, the IOLMaster 500 and the OA-2000 for ACD, the AL-Scan and the Lenstar for CD, the Argos and the Lenstar for CD, the Argos and the IOLMaster 700 for CD. We also used the design-by-treatment interactions model and found that global inconsistency existed for Kf, ACD, CCT and CD (*P* = 0.0041, *P* < 0.001, *P* < 0.001, *P* < 0.001, respectively) (Additional file 1: Appendix I Tables S27–S38).

#### Subgroup analysis

The results of the subgroup analysis also found no global inconsistency existing for AL, Ks, Km, AST, J_0_, J_45_, AQD and LT, and did not significantly change the results of the original network meta-analysis. There were 14 trials involving 9 instruments in the subgroups for the Kf measurement in cataract subjects. This process produced no significant inconsistency in the results. Statistically significant differences existed between the OA-2000 and the Pentacam AXL (WMD = 0.4 D); the OA-2000 and the Lenstar (WMD = 0.28 D) (full process and data shown in Additional file 3: Appendix II Tables S1–S18 and Additional file 3: Appendix II Tables S22–S39). Taking ACD into consideration, the subgroup in healthy subjects prompted no significant inconsistency in the results. Statistically significant differences only existed between the OA-2000 and the IOLMaster 500 (WMD = 0.07 mm); the Lenstar and the IOLMaster (WMD = 0.08 mm); the Pentacam AXL and contact ultrasound biometry (WMD = 0.13 mm); the Argos and contact ultrasound biometry (WMD = 0.18 mm); the OA-2000 and contact ultrasound biometry (WMD = 0.10 mm); the Lenstar and contact ultrasound biometry (WMD = 0.11 mm); the IOLMaster and contact ultrasound biometry (WMD = 0.06 mm). For CCT and CD, the subgroup in healthy subjects both found no significant inconsistency in the results. When considering the measurement of the CCT, there was no statistically significant difference between the Argos and the IOLMaster 700, which differs from the network meta-analysis. As for the measurement of the CD, statistically significant differences only existed when the OA-2000 was compared to the Lenstar and the IOLMaster.

Since there were global inconsistencies noted for Kf, ACD and CCT, we further performed comparison between groups according to the principle of the measurements. With respect to Kf, there was no statistically significant difference among the different measurement principles (*P* > 0.05). The principle of the measurements was ranked consulting the maximum to minimum Kf values depending on the SUCRA values: automated keratometer (AL-Scan, IOLMaster, IOLMaster 500, IOLMaster 700, Lenstar), Placido (Galilei G6, OA-2000, Aladdin), Scheimpflug (Pentacam AXL). The results were consistent with the results of the original network meta-analysis. When considering the measurement of the CCT, there was also no statistically significant difference among the different principles (*P* > 0.05). The principle of the measurements was ranked consulting the maximum to the minimum CCT values depending on the SUCRA values: A-Scan ultrasound (contact ultrasound), Scheimpflug (Galilei G6, AL-Scan, Pentacam AXL), OLCR (Lenstar), SS-OCT (IOLMaster 700, ANTERION, Argos, OA-2000), OLCI (Aladdin). The results are essentially in agreement with the results of the original network meta-analysis. For the ACD, there were statistically significant differences between the A-Scan ultrasound and the following principle: PCI, OLCR, OLCI, SS-OCT, Scheimpflug. Statistically significant differences also existed between the SS-OCT and the PCI. These results were consistent with the results from the original network meta-analysis (Additional file 3: Appendix II Tables S19–S21 and Additional file 3: Appendix II Tables S40–S42).

#### Publication bias

Comparison-adjusted funnel plots for each parameter are provided in Additional file 1: Appendix I Figs. S1–S12. Most of these plots except ACD showed that the included studies lie symmetrically around the “0” line (vertical line). However, the significant publication bias in the ACD did not show up when we performed subgroup analysis for the ACD measurement in healthy subjects (Additional file 3: Appendix II Figs. S1–S18).

## Discussion

This is the first network-based big data meta-analysis that comprehensively compares the instruments and techniques used for ophthalmic biometry. We performed an in-depth statistical comparison of 12 optical instruments and two ultrasound biometry methods by combining the data from 129 studies involving 17,181 eyes. The network meta-analysis demonstrated that when considering the measurement of AL and ACD, contact ultrasound biometry obtained lower values compared to all optical biometers. When considering the measurement of LT, contact ultrasound biometry obtained larger values compared to Galilei G6, IOLMaster 700 and Lenstar. Looking at the four SS-OCT based devices (IOLMaster 700, OA-2000, Argos and ANTERION), no statistical differences existed. In addition, the Pentacam AXL achieved the lowest values of the keratometry and CD. As for the AST, J_0_ and J_45_, there were no statistically significant differences among the instruments included in this study. Besides, we found that the lowest value of CCT measurement was given by the OA-2000, compared with the following instruments: IOLMaster 700, Lenstar, Pentacam AXL, AL-Scan and Galilei G6.

Many studies found that A-scan contact ultrasound biometry measured smaller AL and ACD and larger LT values compared to optical biometers which is consistent with our conclusion [26–30]. The discrepancy for AL and ACD occurs because with contact ultrasound biometry the probe is likely to compress the cornea; with regards to LT, the difference may depend on the index of refraction used by optical biometers to convert the optical path length into a geometrical distance [28]. SS-OCT has some advantages over other optical technologies used for optical biometry, such as long-range OCT imaging or deeper light penetration [31]. Montes-Mico et al. [32] summarized the outcomes reported among four SS-OCT based devices (IOLMaster 700, OA-2000, Argos and ANTERION), and found that the mean differences in AL, ACD and LT measurements for repeatability and reproducibility among the four devices were close to zero. Moreover, many studies reported that agreement between these devices was good. Here, our results are in tandem with previous findings.

Our study also found that the minimum value of Km, Kf and Ks measurement were all given by the Pentacam AXL. It was worth mentioning that the mean K value was a little flatter when measured by the Pentacam AXL compared to the Lenstar [33]. Maria Muzyka-Woźniak et al. [34] also reported that flatter K values were obtained with the Pentacam AXL in comparison to the IOLMaster 500. The Pentacam AXL measures K values at 138,000 reference points orientated in circles at approximately 3.0 mm optical zones on the cornea, which is different with other devices; it is the only instrument that does not rely on corneal reflection [35]. As for J_0_, J_45_ and AST, the network meta-analysis results showed no statistically significant differences among the following devices: OA-2000, IOLMaster, Argos, IOLMaster 500, Aladdin, Pentacam AXL, AL-scan, IOLMaster 700 and Lenstar.

In this study, the lowest value of CCT measurement was given by the OA-2000 when compared with the following instruments: IOLMaster 700, Lenstar, Pentacam AXL, AL-Scan and Galilei G6. The maximum value of CCT measurement was obtained by the Galilei G6 (according to the SUCRA). The difference may be explained by the differences in algorithms and analysis programs of the two devices in boundary determination. The Galilei G6 CCT uses the Scheimpflug principle and measures CCT from the air-tear film surfaces to the posterior corneal surface. The OA-2000 uses a 1060 nm swept source laser to measure the CCT from the anterior corneal surface to the posterior corneal surface. Since the former technology can measure beyond the anterior surface, corneal thickness and posterior corneal curvature can be evaluated with high precision [36].

Here, the Aladdin and Pentacam AXL gave lower values of CD compared to the IOLMaster 700, IOLMaster 500, Lenstar and IOLMaster. There was no statistically significant difference between the Aladdin and the Pentacam AXL. In addition, according to the SUCRA, the IOLMaster was most likely to obtain the maximum CD value. Sabatino et al. [37] described that the IOLMaster produced a greater mean value for CD than the Aladdin. Huang et al. [15] and Cruysberg et al. [38] arrived at the same conclusion. Further, Yeu et al. [39] found that the CD distance showed statistically significant differences (− 0.4 mm on average) between the Aladdin and the Lenstar. This may be attributed to Aladdin’s use of corneal topography whereas the IOLMaster uses photographic techniques to determine the CD [18, 40]. Based on these results, the CD measurements with the Aladdin and the IOLMaster could not be used interchangeably.

In relation to Kf, ACD, CCT and CD measurements, our results indicate that there is too much heterogeneity to draw reliable conclusions. Differences in population and AL may influence the measurements [38, 41]. Therefore, we performed subgroup analyses in two population groups: healthy *vs.* diseased (cataract), and limited the subgroups to adults and normal AL range (22 to 26 mm). The subgroup analysis for the Kf measurement in cataract subjects found no significant inconsistency. However, there was a marked inconsistency amongst the healthy subjects, which can be due to few studies that have directly compared healthy subjects with these devices (such as the OA-2000 vs. the IOLMaster). Regarding ACD, CCT and CD, we also conducted subgroup analyses and found no inconsistency in healthy subjects but a marked inconsistency amongst the cataract subjects. This may be due to the different wavelength in the light source used by the various devices, thus causing the results to be largely affected by the different degree of turbidity of the refractive medium. The various degrees of cataractous lens opacification (cortical, nuclear or posterior subcapsular) may be the cause of inconsistency in cataract subjects [42]. However, since the included articles lack sufficient data for this type of subgroup analysis, it is recommended that future studies could pay more attention to this aspect.

Our study also had other limitations. There were some differences in characteristics of included studies (such as the racial diversity of studied populations, varying degrees of sample size, quality of study methods employed, operator competency, the time interval between equipment measurements and publication bias) that may influence both heterogeneity in direct comparisons and transitivity in indirect comparison in subgroup analyses. To explore the possible impact of these factors on the results, more high-quality studies with concordant features are needed to enhance the statistical effectiveness and quality of evidence in the future. Since new ophthalmic technologies are invented continuously, we have not included all the available instruments in clinical practice, but only focused on the anterior segment and AL biometry.

## Conclusion

This network-based big data analysis demonstrated that when considering the measurement of AL and ACD, contact ultrasound biometry obtains lower values compared with optical biometers. For LT, contact ultrasound biometry obtains larger values compared with Galilei G6, IOLMaster 700 and Lenstar. The Pentacam AXL was also shown to achieve the lowest values with respect to keratometry and CD. Additionally, it was demonstrated that the lowest value of CCT measurement was given by the OA-2000, compared with the following instruments: IOLMaster 700, Lenstar, Pentacam AXL, AL-Scan, and Galilei G6.

## Supplementary Information

**Additional file 1.** Search strategy.

**Additional file 2. Supplemental Figure 1.** Formula rank in axial length (AL), anterior chamber depth (ACD), and lens thickness (LT). **a** Ranking probability results in the AL; **b** Ranking probability results in the ACD; **c** Ranking probability results in LT. **Supplemental Figure 2.** Formula rank in keratometry and corneal diameter (CD). **a** Ranking probability results in the keratometry in the flattest meridian (Kf); **b** Ranking probability results in the keratometry in the steepest meridian (Ks); **c** Ranking probability results in the mean keratometry (Km); **d** Ranking probability results in CD. **Supplemental Figure 3.** Formula rank in astigmatism. **a** Ranking probability results in the J0; **b** Ranking probability results in the J45; **c** Ranking probability results in astigmatism. J0, anterior corneal power vectors for the cardinal (axes at 90 degrees and 180 degrees) meridians; J45, anterior corneal power vectors for the oblique (axes at 45 degrees and 135 degrees) meridians.

**Additional file 3.** Supplementary tables.

## Data Availability

All data generated or analysed during this study are included in this published article.

## Abbreviations

AL: Axial length
Kf: Flattest meridian keratometry
Ks: Steepest meridian keratometry
Km: Mean keratometry
AST: Astigmatism
ACD: Anterior chamber depth
AQD: Aqueous depth
CCT: Central corneal thickness
CD: Corneal diameter
WTW: White-to-white
LT: Lens thickness
OCT: Optical coherence tomography
IOL: Intraocular lens
PCI: Partial coherence interferometry
OLCR: Optical low-coherence reflectometry
OLCI: Optical low-coherence interferometry
SS-OCT: Swept-source optical coherence tomography
OR: Odds ratios
CI: Confidence intervals
WMD: Weighted mean differences
CrI: Credible intervals
SUCRA: Surface under the cumulative ranking curve
