# Supplementary material for: Comprehensive comparisons of ocular biometry: A network-based big data analysis
Source: Eye Vis (Lond). 2022 Dec 10;10:1. doi: 10.1186/s40662-022-00320-3 (PMC9808957; doi:10.1186/s40662-022-00320-3)
Supplement: Supplementary file 1 — Additional file 1. Search strategy. [file 40662_2022_320_MOESM1_ESM.docx]

**Search strategy**

-----------------------------------------

**MEDLINE (PubMed)**

(ultrasonography[MeSH Terms] OR ultrasonics[MeSH Terms] OR ultraso* OR A-scan OR “partial coherence interferometer” OR PCI OR IOLMaster OR AL-Scan OR OA-1000 OR Pentacam AXL OR “Galilei G6” OR “optical low-coherence reflectometry” OR OLCR OR Lenstar OR “optical low-coherence interferometry” OR OLCI OR Aladdin OR tomography, optical coherence[MeSH Terms] OR “optical coherence tomography” OR SS-OCT OR OA-2000 OR Argos OR Anterion) AND (axial length, Eye[MeSH Terms] OR “axial length” OR AL OR astigmatism[MeSH Terms] OR astigmatism OR keratometry OR “corneal curvature” OR AST OR “corneal power” OR “anterior chamber depth” OR ACD OR “aqueous depth” OR AQD OR “central corneal thickness” OR CCT OR “white to white” OR WTW OR “corneal diameter” OR CD OR “Lens Thickness” OR LT) AND (agreement OR consistency OR accuracy OR precise)

---------------------------------------

**Cochrane Central Register of Controlled Trials (CENTRAL) in The Cochrane Library (Wiley)**

#1 MeSH descriptor: [ultrasonography] explode all trees

#2 MeSH descriptor: [ultrasonics] explode all trees

#3 ultraso*

#4 A-scan

#5 “partial coherence interferometer”

#6 IOLMaster

#7 AL-Scan

#8 OA-1000

#9 Pentacam AXL

#10 “Galilei G6”

#11 “optical low-coherence reflectometry”

#12 Lenstar

#13 “optical low-coherence interferometry”

#14 Aladdin

#15 MeSH descriptor: [tomography, optical coherence] explode all trees

#16 “optical coherence tomography”

#17 OA-2000

#18 Argos

#19 Anterion

#20 #1 or #2 or #3 or #4 or #5 or #6 or #7 or #8 or #9 or #10 or #11 or #12 or #13 or #14 or #15 or #16 or #17 or #18 or #19

#21 MeSH descriptor: [axial length, Eye] explode all trees

#22 “axial length”

#23 MeSH descriptor: [Astigmatism] explode all trees

#24 astigmatism

#25 keratometry

#26 “corneal curvature”

#27 “corneal power”

#28 “anterior chamber depth”

#29 “aqueous depth”

#30 “central corneal thickness”

#31 “white to white”

#32 “corneal diameter”

#33 ““Lens Thickness””

#34 #21 or #22 or #23 or #24 or #25 or #26 or #27 or #28 or #29 or #30 or #31 or #32 or #33

#35 #20 and #34

---------------------------------------

**EMBASE (Ovid)**

(‘ultrasonography’/exp OR ‘ultrasonics’/exp OR ultraso* OR A-scan OR ‘partial coherence interferometer’ OR PCI OR IOLMaster OR AL-Scan OR OA-1000 OR Pentacam AXL OR ‘Galilei G6’ OR ‘optical low-coherence reflectometry’ OR OLCR OR Lenstar OR ‘optical low-coherence interferometry’ OR OLCI OR Aladdin OR ‘tomography, optical coherence’/exp OR ‘optical coherence tomography’ OR SS-OCT OR OA-2000 OR Argos OR Anterion) AND (‘axial length, Eye’/exp OR ‘axial length’ OR AL OR ‘astigmatism’/exp OR astigmatism OR keratometry OR ‘corneal curvature’ OR AST OR ‘corneal power’ OR ‘anterior chamber depth’ OR ACD OR ‘aqueous depth’ OR ‘AQD’ OR ‘central corneal thickness’ OR CCT OR ‘white to white’ OR WTW OR ‘corneal diameter’ OR CD OR ‘lens thickness’ OR LT) AND (agreement OR consistency OR accuracy OR precise)

---------------------------------------

**ClinicalTrials.gov**

(A-scan OR IOLMaster OR AL-Scan OR OA-1000 OR Galilei G6 OR Pentacam AXL OR Lenstar OR Aladdin OR SS-OCT) AND (axial length OR keratometry OR corneal curvature OR anterior chamber depth OR aqueous depth OR thickness OR white to white)

Appendix I Table 1: Summary of studies included in the network meta-analysis

| Study (Author, year) | Country | Population | Devices | Age (mean±SD, years) | Total | Axial  length (mm) | Kf (D) | Ks (D) | Km (D) | J0 (D) | J45 (D) | AST (D) | ACD (mm) | AQD (mm) | CCT (μm) | CD (mm) | LT (mm) |
| --- | --- | --- | --- | --- | --- | --- | --- | --- | --- | --- | --- | --- | --- | --- | --- | --- | --- |
| Akman et al. 2016 [1] | Turkey | Cataract | IOLMaster 700 | 68.32±12.71 | 188 | 23.622±1.32 | 43.41±1.73 | 44.42±1.83 | 43.94±1.20 | - | - | 1.043±2.610 | 3.23±0.37 | - | - | - | - |
|  |  |  | IOLMaster 500 |  |  | 23.627±1.25 | 43.46±1.77 | 44.50±1.81 | 44.07±1.25 | - | - | 1.21±2.50 | 3.31±0.38 | - | - | - | - |
| Aktas et al. 2015 [2] | Turkey | Cataract | AL-Scan | 66.87±9.11 | 70 | 23.29±0.79 | - | - | - | - | - | - | - | - | - | - | - |
|  |  |  | Contact ultrasound |  |  | 23.23±0.81 | - | - |  |  | - | - | - | - | - | - |  |
| Alvani et al. 2016 [3] | Iran | Glaucoma | Lenstar | 52.90±11.98 | 31 | 23.65±1.4 | - | - | 44.19±1.54 | - | - | - | 3.13±0.44 | - | - | - | 4.26±0.40 |
|  |  |  | Contact ultrasound |  |  | 23.43±1.38 | - | - | - | - | - | - | 3.09±0.45 | - | - | - | - |
| An et al. 2019 [4] | Korea | Cataract | IOLMaster | 66.70±10.54 | 128 | 24.56±2.16 | - | - | 44.14±1.47 | - | - | - | - | - | - | - | - |
|  |  |  | Argos |  |  | 24.58±2.22 | - | - | 44.16±1.50 | - | - | - | - | - | - | - | - |
|  |  |  | Contact ultrasound |  |  | 24.49±2.19 | - | - | - | - | - | - | - | - | - | - | - |
| Arriola-Villalobos et al. 2017 [5] | Spain | Cataract | IOLMaster 700 | 76.3±6.8 | 80 | 23.536±1.54 | - | - | 44.56±1.62 | - | - | - | 3.09±0.33 | 2.54±0.34 | 544.74±34.45 | 11.81±0.44 | 4.71±0.40 |
|  |  |  | Lenstar |  |  | 23.531±1.29 | - | - | 44.62±1.63 | - | - | - | 3.11±0.36 | 2.56±0.34 | 542.23±33.32 | 11.82±0.59 | 4.67±0.38 |
| Asena et al. 2018 [6] | Turkey | Cataract | IOLMaster 700 | 68.32±12.71 | 101 | - | 43.45±1.70 | 44.36±1.75 | 43.90±1.67 | −0.019±0.360 | 0.103±0.450 | 0.99±0.79 | - | - | - | - | - |
|  |  |  | IOLMaster 500 |  |  | - | 43.49±1.65 | 44.43±1.65 | 43.96±1.60 | −0.018±0.410 | −0.011±0.440 | 1.04±0.81 | - | - | - | - | - |
| Attas-Fox et al. 2007 [7] | Israel | Healthy | IOLMaster | 61±10 | 23 | 23.25±1.10 | - | - | - | - | - | - | - | - | - | - | - |
|  |  |  | Contact ultrasound |  |  | 23.18±1.07 | - | - | - | - | - | - | - | - | - | - | - |
| Aydin et al. 2016 [8] | Turkey | Hypermetropia | Lenstar | 54.78±12.77 | 52 | 21.6±0.8 | - | - | - | - | - | - | 2.9±0.5 | - | - | - | 4.2±0.4 |
|  |  |  | Contact ultrasound |  |  | 21.5±0.7 | - | - | - | - | - | - | 3.1±0.3 | - | - | - | 4.3±0.4 |
| Bai et al. 2007 [9] | China | Cataract | IOLMaster | 67.23±12.18 | 137 | 24.37±3.04 | - | - | - | - | - | - | 2.94±0.49 | - | - |  | - |
|  |  |  | Contact ultrasound |  |  | 23.81±2.83 | - | - | - | - | - | - | 2.58±0.51 | - | - | - | - |
| Bi et al. 2020 [10] | China | Myopia | Pentacam AXL | 22.36±4.67 | 112 | - | - | - | - | - | - | - | - | - | 530.17±25.08 | - | - |
|  |  |  | OA-2000 |  |  | - | - | - | - | - | - | - | - | - | 521.75±26.51 | - | - |
|  |  |  | IOLMaster 700 |  |  | - | - | - | - | - | - | - | - | - | 519.53±28.15 | - | - |
| Buckhurst et al. 2009 [11] | UK | Cataract | Lenstar | 76.4±9.1 | 112 | 23.25±2.21 | 42.78±2.83 | 43.88±2.74 | 43.37±2.02 | - | - | 1.09±4.00 | 3.19±0.93 | - | - | 12.08±0.86 | - |
|  |  |  | IOLMaster |  |  | 23.24±2.19 | 42.82±2.83 | 43.93±2.82 | 43.37±1.95 | - | - | 1.01±3.80 | 3.09±1.02 | - | - | 12.15±0.95 | - |
| Bullimore et al. 2019 [12] | USA | Healthy | IOLMaster 700 | 33.7±9.2 | 48 | 24.25±1.24 | - | - | - | - | - | −1.12±0.85 | 3.66±0.32 | - | 555±35 | 12.2±0.5 | 3.80±0.36 |
|  |  |  | IOLMaster 500 |  |  | 24.21±1.24 | - | - | - | - | - | −1.14±0.84 | 3.66±0.30 | - | - | 12.3±0.6 | - |
|  |  |  | Lenstar |  |  | - | - | - | - | - | - | - | - | - | 550±35 | - | 3.76±0.34 |
|  |  | Cataract | IOLMaster 700 | 62.3±8.1 | 51 | 24.01±1.29 | - | - | - | - | - | −0.83±0.62 | 3.26±0.34 | - | 552±30 | 12.1±0.4 | 4.55±0.39 |
|  |  |  | IOLMaster 500 |  |  | 23.99±1.29 | - | - | - | - | - | −0.83±0.60 | 3.27±0.33 | - | - | 12.1±0.4 | 4.47±0.42 |
|  |  |  | Lenstar |  |  | - | - | - | - | - | - | - | - | - | 548±31 | - | - |
| Caglar et al. 2016 [13] | Turkey | Healthy | AL-Scan | 39.24±14.37 | 85 | 23.31±0.90 | - | - | - | - | - | - | 3.35±0.40 | - | - | - | - |
|  |  |  | Contact ultrasound |  |  | 23.01±0.92 | - | - | - | - | - | - | 3.32±0.41 | - | - | - | -- |
| Calvo-Sanz et al. 2018 [14] | Spain | Cataract | IOLMaster 700 | 73.12±2.63 | 55 | 23.89±1.99 | - | - | 44.06±1.67 | - | - | - | 3.14±0.39 | 2.59±0.40 | 545.81±32.60 | - | 4.6±0.42 |
|  |  |  | Aladdin |  |  | 23.89±2.01 | - | - | 44.13±1.72 | - | - | - | 3.18±0.38 | 2.64±0.38 | 536.52±32.25 | - | 4.62±0.43 |
| Can et al. 2019 [15] | Turkey | Healthy | AL-Scan | 33.6 ±9.7 | 80 | - | - | - | - | - | - | - | - | - | 554.6±30.9 | - | - |
|  |  |  | Lenstar |  |  | - | - | - | - | - | - | - | - | - | 542.9±31.3 | - | - |
| Can et al. 2016 [16] | Turkey | Healthy | AL-Scan | 33.1±9.7 | 70 | 23.69±1.08 | - | - | 43.28±1.45 | - | - | - | 3.56±0.38 | 3.03±0.40 | 533±28 | 12.01±0.50 | - |
|  |  |  | Lenstar |  |  | 23.68±1.09 | - | - | 43.32±1.50 | - | - | - | 3.51±0.39 | 3.0±0.4 | 522±28 | 12.14±0.49 | - |
| Carkeet et al. 2004 [17] | China | Child | IOLMaster | 10.6±0.8 | 179 | 24.14±1.06 | - | - | - | - | - | - | 3.69±0.22 | - | - | - | - |
|  |  |  | Contact ultrasound |  |  | 24.00±1.05 | - | - | - | - | - | - | 3.60±0.27 | - | - | - | - |
| Cech et al. 2014 [18] | Beskydy | Cataract | IOLMaster | 72 | 335 | 23.15 | - | - | - | - | - | - | - | - | - | - | - |
|  |  |  | Contact ultrasound |  |  | 23.00 |  | - | - | - | - | - | - | - | - | - | - |
| Chan et al. 2019 [19] | China | Cataract | IOLMaster 700 | 68.7±8.6 | 52 | 24.230±1.623 | - | - | 43.986±1.480 | −0.147±0.460 | −0.058±0.270 | - | 3.03±0.43 | 2.49±0.45 | 543.821±30.650 | 11.894±0.360 | - |
|  |  |  | AL-Scan |  |  | 24.221±1.620 | - | - | 44.194±1.520 | −0.177±0.490 | −0.021±0.260 | - | 3.04±0.44 | 2.49±0.43 | 528.808±30.740 | 11.568±0.380 | - |
| Chen et al. 2011 [20] | Austria | Cataract | Lenstar | 76.2 | 109 | 23.34±1.20 | - | - | 43.23±1.41 | - | - | - | - | - | - | - | - |
|  |  |  | IOLMaster |  |  | 23.36±1.14 | - | - | 43.35±1.48 | - | - | - | - | - | - | - | - |
|  |  |  | IOLMaster 500 |  | 59 | 23.65±1.19 | - | - | 43.21±1.62 | - | - | - | 3.13±0.58 | - | - | 11.88±0.47 | - |
|  |  |  | IOLMaster |  |  | 23.67±1.20 | - | - | 43.25±1.60 | - | - | - | 3.12±0.49 | - | - | 12.15±0.47 | - |
| Cheng et al. 2020 [21] | China | Cataract | IOLMaster 700 | 65.09±11.25 | 133 | 24.47±2.20 | - | - | 44.34±1.58 | - | - | - | 3.16±0.43 | 2.63±0.44 | 545.84±35.06 | 11.80±0.48 | 4.45±0.47 |
|  |  |  | Lenstar |  |  | 24.48±2.22 | - | - | 44.36±1.62 | - | - | - | 3.22±0.44 | 2.66±0.43 | 541.24±34.10 | 11.53±0.45 | 4.29±0.48 |
| Cho et al. 2018 [22] | South Korea | Cataract | IOLMaster 700 | 66.2 ± 10.1 | 137 | 23.82±1.67 | - | - | 44.29±1.27 | - | - | - | 3.16±0.51 | - | - | - | - |
|  |  |  | IOLMaster 500 |  | 127 | 23.79±1.67 | - | - | 44.39±1.28 | - | - | - | - | - | - | - | - |
|  |  |  | Contact ultrasound |  | 137 | 23.75±1.67 | - | - | - | - | - | - | 3.03±0.28 | - | - | - | - |
| Cinar et al. 2013 [23] | Turkey | keratoconus | Lenstar | 19.06±5.33 | 42 | 23.42±1.08 | - | - | - | - | - | - | 3.87±0.32 | 3.44±0.32 | 451.0±42.1 | - | 3.50±0.19 |
|  |  |  | Contact ultrasound |  |  | 23.52±1.10 | - | - | - | - | - | - | 3.87±0.32 | 3.42±0.32 | 457.0±45.9 | - | 3.64±0.22 |
| Cruysberg et al. 2009 [24] | Netherlands | Healthy | Lenstar | 25.9±8.6 | 76 | 23.92±1.23 | 43.27±1.69 | 44.12±1.69 | 43.72±1.14 | - | - | 0.84±2.42 | 3.66±0.29 | - | - | 12.17±0.53 | - |
|  |  |  | IOLMaster |  |  | 23.90±1.22 | 43.32±1.61 | 44.23±1.53 | 43.78±1.09 | - | - | 1.12±2.21 | 3.61±0.26 | - | - | 12.36±0.32 | - |
| Cvetkovic et al. 2016 [25] | Serbia | Cataract | IOLMaster 500 | 71.21±1.68 | 29 | 24.04±0.29 | - | - | - | - | - | - | - | - | - | - | - |
|  |  |  | Contact ultrasound |  |  | 23.89±0.28 | - | - | - | - | - | - | - | - | - | - | - |
| Doğan et al. 2014 [26] | Turkey | Cataract | AL-Scan | 68.00±13.15 | 42 | 23.75±1.16 | 43.26±1.55 | 44.59±2.41 | 43.968±1.360 | - | - | 1.4±2.98 | 3.21±0.40 | - | - | - | - |
|  |  |  | Lenstar |  |  | 23.79±1.17 | 42.99±1.56 | 44.52±2.07 | 43.84±1.28 | - | - | 1.6±2.59 | 3.23±0.39 | - | - | - | - |
| Du et al. 2019 [27] | China | Cataract | OA-2000 | 57.39±10.74 | 46 | 29.08±2.31 | - | - | 44.60±1.27 | - | - | - | 3.64±0.56 | - | - | - | - |
|  |  |  | IOLMaster 500 |  |  | 29.06±2.30 | - | - | 44.59±1.27 | - | - | - | 3.70±0.55 | - | - | - | - |
| Einan-Lifshitz et al. 2017 [28] | Israel | Cataract | immersion ultrasound | 70.63 | 27 | 23.24±0.94 | - | - | - | - | - | - | - | - | - | - | - |
|  |  |  | OA-2000 |  |  | 23.14±0.98 | - | - | - | - | - | - | - | - | - | - | - |
| El-Baha et al. 2009 [29] | Egypt | Silicone-filled eyes | IOLMaster | 47.0±12.1 | 22 | 24.28±2.84 | - | - | - | - | - | - | - | - | - | - | - |
|  |  |  | Contact ultrasound |  |  | 24.56±2.11 | - | - | - | - | - | - | - | - | - | - | - |
| Elbaz et al. 2007 [30] | Israel | Healthy | IOLMaster | 56.3±14.0 | 22 | - | - | - | 43.92 ± 1.298 | - | - | - | 3.01 ± 0.36 | - | - | - | - |
|  |  |  | Contact ultrasound |  |  | - | - | - | - | - | - | - | 3.01 ± 0.36 | - | - | - | - |
| El Chehab et al. 2018 [31] | France | Cataract | IOLMaster 700 | 72.4 ± 1.5 | 128 | 23.53±1.91 | - | - | 43.81±0.25 | - | - | - | 3.59±0.07 | 3.05±0.07 | 539.09±6.32 | 11.97±0.07 | 4.63±0.08 |
|  |  |  | Lenstar |  |  | 23.53±1.91 | - | - | 43.84±0.38 | - | - | - | 3.61±0.07 | 3.07±0.07 | 540.13±5.20 | 12.06±0.07 | 4.66±0.08 |
| Eleftheriadis et al. 2003 [32] | UK | Cataract | IOLMaster | 75.42±7.58 | 100 | 23.36±0.85 | - | - | - | - | - | - | - | - | - | - | - |
|  |  |  | Contact ultrasound |  |  | 22.89±0.83 | - | - | - | - | - | - | - | - | - | - | - |
| Epitropoulos et al. 2014 [33] | USA | Cataract | IOLMaster 500 | 71.5±0.5 | 97 | - | - | - | 44.12±1.54 | - | - | - | 3.05±0.39 | - | - | - | - |
|  |  |  | Lenstar |  | 89 | - | - | - | 44.06±1.56 | - | - | - | 3.15±0.36 | - | - | - | - |
| Findl et al. 2003 [34] | Austria | Cataract | IOLMaster | - | 622 | 23.47±1.42 | - | - | - | - | - | - | 3.10±0.43 | - | - | - | - |
|  |  |  | Contact ultrasound |  | 622 | 23.33±1.44 | - | - | - | - | - | - | 2.90±0.47 | - | - | - | - |
| Fișuș et al. 202 0 [35] | Austria | Cataract | IOLMaster 700 | 70.3 ± 9.6 | 389 | 23.55±1.18 | - | - | - | 0.31±0.28 | 0.32±0.30 | - | 3.13±0.43 | 2.59±0.43 | 553.52±33.38 | - | 4.59±0.43 |
|  |  |  | Anterion |  |  | 23.54±1.18 | - | - | - | 0.31±0.30 | 0.29±0.29 | - | 3.20±0.42 | 2.67±0.45 | 547.86±32.05 | - | 4.65±0.43 |
| Fontes et al. 2011 [36] | Brazil | Cataract | IOLMasger | 69.8±13.1 | 50 | 23.22±1.00 | - | - | - | - | - | - | - | - | - | - | - |
|  |  |  | Immersion ultrasound | 70.0±9.3 | 70 | 23.22±1.06 | - | - | - | - | - | - | - | - | - | - | - |
| Gao et al. 2017 [37] | China | Healthy | OA-2000 | 29.4±9.6 | 99 | 24.81±1.51 | 43.09±1.39 | 44.03±1.49 | 43.56±1.41 | −0.40±0.35 | 0.03±0.13 | 0.94±0.57 | 3.57±0.34 | 3.04±0.34 | 523.97±31.59 | 11.77±0.38 | 3.86±0.37 |
|  |  |  | Lenstar |  |  | 24.80±1.51 | 43.09±1.40 | 44.01±1.51 | 43.55±1.43 | −0.37±0.36 | 0.00±0.15 | 0.92±0.57 | 3.57±0.35 | 3.03±0.35 | 536.80±32.38 | 11.90±0.41 | 3.78±0.38 |
| Ghaffari et al. 2019 [38] | Iran | Cataract | OA-2000 | 61.4±8.3 | 58 | 23.27±1.21 | 44.26±1.75 | 45.22±1.92 | 44.72±1.29 |  |  | 1.10±2.66 | 3.28±0.38 |  |  |  |  |
|  |  |  | IOLMaster 500 |  |  | 23.05±0.98 | 44.22±1.55 | 45.34±1.90 | 44.80±1.27 |  |  | 1.18±2.60 | 3.14±0.37 |  |  |  |  |
| Goebels et al. 2015 [39] | Germany | Cataract | OA-2000 | 71±12 | 138 | 23.30±1.27 | 43.66±1.125 | 44.18±1.21 | 43.92±0.82 | - | - | 0.59±1.69 | 3.71±0.51 | 3.16±0.51 | 536.54±33.33 | - | 4.73±0.48 |
|  |  |  | Lenstar |  |  | 23.3±1.3 | 43.27±1.35 | 44.41±1.35 | 43.90±0.95 | - | - | 1.24±1.81 | 3.09±0.47 | 2.49±0.49 | 552.42±33.67 | - | 4.63±0.48 |
|  |  |  | IOLMaster |  |  | 23.36±1.28 | 43.38±1.35 | 44.41±1.30 | 43.84±1.00 | - | - | 1.01±1.74 | 3.00±0.45 | - | - | - | - |
| Goebelset al. 2013 [40] | Germany | Cataract | OA-1000 | 72.0±9.5 | 133 | 22.97±1.10 | - | - | - | - | - | - | 3.40±0.46 | - | - | - | - |
|  |  |  | IOLMaster |  |  | 23.21±1.08 | - | - | - | - | - | - | 2.99±0.41 | - | - | - | - |
|  |  |  | Contact ultrasound |  |  | 22.79±1.04 | - | - | - | - | - | - | 2.93±0.43 | - | - | - | - |
| Goto et al. 2020 [41] | Japan | Cataract | Argos | 75.3 ± 8.3 | 268 | 24.12±1.54 | - | - | - | - | - | - | - | - | - | - | - |
|  |  |  | OA-2000 |  |  | 24.15±1.58 | - | - | - | - | - | - | - | - | - | - | - |
|  |  |  | IOLMaster 500 |  |  | 24.14±1.58 | - | - | - | - | - | - | - | - | - | - | - |
|  |  | Postoperative | Argos |  |  | 24.12±1.54 | - | - | - | - | - | - | - | - | - | - | - |
|  |  |  | OA-2000 |  |  | 24.05±1.59 | - | - | - | - | - | - | - | - | - | - | - |
|  |  |  | IOLMaster 500 |  |  | 24.05±1.58 | - | - | - | - | - | - | - | - | - | - | - |
| Goyal et al. 2003 [42] | UK | Cataract | IOLMaster | 74.85±11.5 | 80 | 23.55±1.76 | - | - | - | - | - | - | - | - | - | - | - |
|  |  |  | Contact ultrasound |  |  | 23.35±1.81 | - | - | - | - | - | - | - | - | - | - | - |
| Güler et al. 2016 [43] | Turkey | Cataract (AL: 22–26) | AL-Scan | 65.73±5.62 | 45 | 23.49±1.00 | 43.25±1.75 | 44.14±1.85 | 43.69±1.79 | - | - | 0.99±2.50 | 3.57±0.43 | 3.02±0.42 | 547.91±30.66 | 11.83±0.40 | - |
|  |  |  | Lenstar |  |  | 23.5±1.00 | 43.13±1.75 | 43.98±1.80 | 43.55±1.76 | - | - | 0.85±2.55 | 3.60±0.42 | 3.05±0.43 | 547.42±30.47 | 11.97±0.40 | - |
|  |  | Cataract (AL: >26) | AL-Scan | 63.54±6.45 | 20 | 27.39±0.78 | 42.9±1.49 | 43.89±1.52 | 43.39±1.49 | - | - | 1.08±2.15 | 3.64±0.35 | 3.10±0.35 | 539.50±23.62 | 11.83±0.44 | - |
|  |  |  | Lenstar |  |  | 27.40±0.79 | 42.83±1.50 | 43.81±1.50 | 43.31±1.48 | - | - | 0.85±2.00 | 3.67±0.35 | 3.13±0.35 | 538.05±23.04 | 11.94±0.44 | - |
| Guo et al. 2019 [44] | China | High myopia | OA-2000 | 48.42±10.77 | 45 | 27.84±1.32 | - | - | 44.41±1.62 | - | - | - | 3.51±0.38 | - | - | 11.48±0.51 | - |
|  |  |  | IOLMaster 500 |  |  | 27.85±1.30 | - | - | 44.46±1.63 | - | - | - | 3.52±0.34 | - | - | 11.55±0.36 | - |
|  |  | Healthy | OA-2000 | 47.49±9.63 | 45 | 23.49±1.29 | - | - | 44.61±1.6 | - | - | - | 3.00±0.33 | - | - | 11.37±0.46 | - |
|  |  |  | IOLMaster 500 |  |  | 23.47±1.30 | - | - | 44.64±1.66 | - | - | - | 2.89±0.36 | - | - | 11.39±0.35 | - |
| Gursoy et al. 2011 [45] | Turkey | child | Lenstar | 10.48±2.11 | 557 | 23.07±0.79 | - | - | - | - | - | - | 3.14±0.24 | 2.60±0.23 | 549.22±30.22 | - | 3.34±0.18 |
|  |  |  | Contact ultrasound |  | 530 | 22.36±0.82 | - | - | - | - | - | - | 2.87±0.36 | - | - | - | 3.64±0.29 |
| Haddad et al. 2020 [46] | South Carolina | Cataract | Pentacam AXL | 69.3±9.2 | 166 | 24.03±1.37 | 43.34±2.08 | 44.18±2.15 | 43.75±2.09 | - | - | 0.71±2.85 | 3.24±0.41 | - | - | - | - |
|  |  |  | IOLMaster 500 |  |  | 24.03±1.37 | 43.40±2.18 | 44.35±2.20 | 43.85±2.16 | - | - | 1.03±3.10 | 3.19±0.44 | - | - | - | - |
| Hashemi et al. 2005 [47] | Iran | myopia | Contact ultrasound | 30.3±8.5 | 88 | - | - | - | - | - | - | - | 3.70±0.31 | - | - | - | - |
|  |  |  | IOLMaster |  |  | - | - | - | - | - | - | - | 3.79±0.30 | - | - | - | - |
| Henriquez et al. 2020 [48] | Peru | Cataract | Pentacam AXL | >45 | 45 | 24.64±1.16 | 42.66±2.40 | 44.18±2.40 | 43.48±2.29 | - | - | 1.48±1.48 | 3.20±0.45 | 2.67±0.46 | 541.09±33.21 | - | - |
|  |  |  | Galilei G6 |  |  | 24.27±1.31 | 42.99±2.41 | 44.25±2.42 | 43.64±2.35 | - | - | 1.39±1.16 | 3.20±0.41 | 2.68±0.41 | 535.30±34.76 | - | 3.98±0.82 |
|  |  |  | IOLMaster 700 |  |  | 24.20±1.28 | 42.74±2.14 | 44.12±2.35 | 43.41±2.16 | - | - | 1.40±1.23 | 3.32±0.46 | 2.78±0.45 | 533.14±35.56 | - | 4.27±0.90 |
| Hoffer et al. 2016 [49] | Germany | Cataract | IOLMaster 700 | 72.9±18.8 | 183 | 23.61±1.27 | 43.40±1.43 | 44.28±1.50 | 43.84±1.43 | - | - | 0.99±1.96 | 3.22±0.44 | 2.67±0.43 | 553.51±35.98 | - | 4.59±0.43 |
|  |  |  | Lenstar |  |  | 23.60±1.27 | 43.37±1.43 | 44.28±1.49 | 43.82±1.43 | - | - | 0.9±1.9 | 3.19±0.44 | 2.62±0.45 | 559.20±37.01 | - | 4.62±0.44 |
| Hoffer et al. 2016 [50] | USA | Cataract | AL-Scan | 72.0±9.1 | 86 | 23.46±0.99 | - | - | 43.84±1.49 | - | - | - | 2.96±0.38 | - | - | - | - |
|  |  |  | IOLMaster 500 |  |  | 23.46±0.99 | - | - | 43.76±1.46 | - | - | - | 2.83±0.38 | - | - | - | - |
| Hoffer et al. 2010 [51] | USA | Cataract | IOLMaster | 74.13±7.12 | 50 | 23.68±1.04 | - | - | 43.69±1.92 | - | - | - | 2.98±0.49 | - | - | - | - |
|  |  |  | Lenster |  |  | 23.71±1.04 | - | - | 43.58±1.87 | - | - | - | 3.11±0.47 | - | - | - | - |
|  |  | Healthy | IOLMaster | 66.22±6.43 | 50 | 23.7±1.2 | - | - | 43.53±2.13 | - | - | - | 2.95±0.39 | - | - | - | - |
|  |  |  | Lenster |  |  | 23.72±1.21 | - | - | 43.41±2.13 | - | - | - | 3.10±0.41 | - | - | - | - |
| Hoffer et al. 2016 [52] | USA | Cataract | Aladdin | 75±10 | 60 | 23.59±0.99 | 43.33±1.71 | 44.22±1.68 | 43.77±1.67 | −0.04±0.46 | 0.00±0.28 | 0.77±2.40 | 3.13±0.43 | - | - | - | - |
|  |  |  | IOLMaster 500 |  |  | 23.58±1.00 | 43.38±1.71 | 44.41±1.70 | 43.90±1.67 | −0.01±0.57 | 0.02±0.31 | 1.16±2.39 | 2.95±0.44 | - | - | - | - |
|  | China | Healthy | Aladdin | 25.54±2.98 | 56 | 25.13±1.02 | 43.12±1.50 | 44.09±1.73 | 43.61±1.60 | −0.02±0.02 | −0.49±0.26 | 0.99±2.14 | 3.72±0.25 | - | - | - | - |
|  |  |  | IOLMaster 500 |  |  | 25.13±1.03 | 43.25±1.56 | 44.24±1.77 | 43.74±1.64 | −0.02±0.02 | −0.49±0.29 | 1.10±2.36 | 3.67±0.27 | - | - | - | - |
| Holzer et al. 2009 [53] | Germany | Healthy | Lenstar | 27.25±10.32 | 200 | 23.58±0.92 | 42.41±1.48 | 43.33±1.48 | 42.93±1.04 | - | - | 0.89±2.16 | 3.64±0.26 | - | - | - | - |
|  |  |  | IOLMaster 500 |  |  | 23.57±0.91 | 42.45±1.48 | 43.37±1.49 | 42.93±1.04 | - | - | 0.74±2.01 | 3.48±0.42 | - | - | - | - |
| Hua 2018 [54] | China | Healthy | OA-2000 | 27.43±7.37 | 108 | 24.56±1.30 | 42.81±1.44 | 43.84±1.61 | 43.32±1.48 | - | - | - | 3.59±0.32 | - | - | 11.88±0.39 | - |
|  |  |  | IOLMaster |  |  | 24.52±1.27 | 43.81±1.46 | 44.03±1.68 | 43.48±1.55 | - | - | - | 3.59±0.33 | - | - | 12.05±0.43 | -- |
| Huang et al. 2012 [55] | China | Noncycloplegia | Lenstar | 22.1±4.7 | 43 | 25.68±1.56 | 42.90±1.61 | 44.22±1.87 | 43.56±1.70 | - | - | - | 3.73±0.30 | - | - | 12.21±0.38 | - |
|  |  |  | IOLMaster |  |  | 25.66±1.57 | 42.82±1.58 | 44.25±1.78 | 43.57±1.64 | - | - | - | 3.73±0.26 | - | - | 12.19±0.37 | - |
| Huang et al. 2019 [56] | China | Cataract | IOLMaster 700 | 68.87 ± 9.82 | 166 | 23.24±1.02 | - | - | - | - | - | - | - | - | - | - | - |
|  |  |  | OA-2000 |  | 166 | 23.22±1.01 | - | - | - | - | - | - | - | - | - | - | - |
|  |  |  | Argos |  | 170 | 23.22±0.99 | - | - | - | - | - | - | - | - | - | - | - |
|  |  |  | IOLMaster 500 |  | 138 | 23.22±1.05 | - | - | - | - | - | - | - | - | - | - | - |
| Huang et al. 2016 [57] | China | Healthy | OA-2000 | 21.71±4.11 | 65 | 25.68±1.16 | 42.47±1.11 | 43.57±1.22 | 43.02±1.12 | - | - | 1.07±1.76 | 3.78±0.26 | - | - | 11.63±0.35 | - |
|  |  |  | IOLMaster 500 |  |  | 25.66±1.18 | 42.65±1.08 | 43.68±1.22 | 43.03±1.14 | - | - | 1.05±1.64 | 3.76±0.28 | - | - | 12.19±0.45 | - |
| Hui et al. 2014 [58] | China | Cataract | Lenstar | 64.6±13.4 | 122 | 24.08±2.12 | 43.38±1.57 | 44.16±1.58 | 43.77±1.55 | - | - | 0.79±2.23 | 3.18±0.42 | - | - | - | - |
|  |  |  | IOLMaster 500 |  |  | 24.06±2.11 | 43.43±1.58 | 44.28±1.62 | 43.86±1.57 | - | - | 0.76±2.30 | 3.20±0.45 | - | - | - | - |
| Hussaindeen et al. 2018 [59] | India | Healthy | Argos | 13.88± 1.69 | 376 | 23.94±1.01 | - | - | 43.62±1.59 | - | - | - | - | - | - | - | - |
|  |  |  | IOLMaster |  |  | 23.83±1.03 | - | - | 43.64±1.61 | - | - | - | - | - | - | - | - |
| Jung et al. 2017 [60] | Korea | Cataract | IOLMaster 700 | 60.4±9.6 | 94 | 24.54±2.08 | 43.10±2.07 | 44.21±2.08 | 43.65±2.01 | - | - | 1.11±2.84 | 3.35±0.39 | 2.77±0.40 | 541.27±33.95 | 11.98±0.41 | 4.12±0.79 |
|  |  |  | Galilei G6 |  |  | 24.53±2.07 | 42.95±2.04 | 44.13±2.08 | 43.54±2.03 | - | - | 1.2±3.0 | 3.31±0.47 | 2.76±0.46 | 552.95±34.35 | 11.92±0.45 | 4.07±0.62 |
| Kaswin et al. 2014 [61] | France | Cataract | AL-Scan | 72.6±4.2 | 50 | 23.71±0.88 | - | - | 43.93±0.46 | - | - | - | 3.17±0.41 | - | - | - | - |
|  |  |  | IOLMaster 500 |  |  | 23.71±0.88 | - | - | 43.91±0.45 | - | - | - | 3.12±0.38 | - | - | - | - |
| Kaya et al. 2016 [62] | Turkey | Cataract | Aladdin | 67.9±7.6 | 37 | 23.4±0.7 | - | - | - | - | - | - | 3.1±0.2 | - | - | - | - |
|  |  |  | Contact ultrasound |  |  | 23.2±0.7 | - | - | - | - | - | - | 3.0±0.3 | - | - | - | - |
| Sharma et al. 2018 [63] | India | Cataract | Contact ultrasound | - | 337 | 23.66±1.88 | - | - | - | - | - | - | - | - | - | - | - |
|  |  |  | IOLMaster |  |  | 23.63±1.90 | - | - | - | - | - | - | - | - | - | - | - |
| Kongsap et al. 2016 [64] | Thailand | Cataract | OA-2000 | 55.81±11.51 | 88 | 23.12±1.34 | - | - | 44.5±1.8 | - | - | - | 3.06±0.39 | - | - | 11.64±0.77 | -- |
|  |  |  | IOLMaster 500 |  |  | 23.18±1.08 | - | - | 44.60±1.81 | - | - | - | 3.15±0.39 | - | - | 11.86±0.48 | - |
| Kunert et al. 2016 [65] | Germany | Cataract | IOLMaster 700 | 68.20±9.89 | 120 | 23.655±1.830 | - | - | - | - | - | - | 3.06±0.42 | 2.500±0.385 | 550.20±36.22 | - | 4.646±0.400 |
|  |  |  | IOLMaster 500 |  |  | 23.650±1.822 | - | - | - | - | - | - | 3.04±0.41 | - | - | - | - |
|  |  |  | Lenster |  |  | - | - | - | - | - | - | - | - | - | 550.20±36.22 | - | 4.626±0.400 |
| Kurian et al. 2016 [66] | India | Cataract | IOLMaster 700 | 60.6±9.6 | 100 | 23.5 | - | - | 44.0±1.4 | - | - | 0.76 | 3.21±0.37 | 2.70±0.38 | 529±33 | - | 4.29 |
|  |  |  | Lenstar |  |  | 23.4 | - | - | 44.0±1.4 | - | - | 0.71 | 3.25±0.39 | 2.72±0.38 | 523±32 | - | 4.25 |
| Lam et al. 2001 [67] | China | Healthy | IOLMaster | 19.30±0.55 | 26 | 24.44±1.21 | - | - | - | - | - | - | 3.60±0.25 | - | - | - | - |
|  |  |  | Contact ultrasound |  |  | 24.54±1.09 | - | - | - | - | - | - | 3.44±0.24 | - | - | - | - |
| Li et al. 2018 [68] | China | Cataract | immersion ultrasound | 59.20±6.57 | 45 | 23.69±1.15 | - | - | - | - | - | - | - | - | - | - | - |
|  |  |  | Lenstar |  |  | 23.61±1.14 | - | - | - | - | - | - | - | - | - | - | - |
| Li et al. 2016 [69] | China | Cataract | AL-Scan | 68.41±8.19 | 92 | 23.13±0.81 | - | - | 44.80±1.39 | 0.06±0.40 | −0.04±0.28 | 0.84±0.51 | 2.96±0.39 | 2.42±0.39 | 534.02±26.60 | 11.23±0.43 | - |
|  |  |  | Lenstar |  |  | 23.12±0.82 | - | - | 44.69±1.41 | 0.06±0.46 | −0.07±0.30 | 0.91±0.62 | 2.95±0.39 | 2.42±0.38 | 532.91±28.60 | 11.40±0.43 | - |
| Li et al. 2020 [70] | China | Cataract | OA-2000 | - | 843 | 28.78±1.77 | 43.14±2.47 | 44.18±2.63 | 43.668±1.810 | - | - | 1.02±3.65 | 3.48±0.36 | - | - | - | - |
|  |  |  | IOLMaster 500 |  |  | 28.78±1.76 | 43.15±2.49 | 44.19±2.68 | 43.725±1.900 | - | - | 0.69±3.60 | 3.54±0.38 | - | - | - | - |
|  |  |  | Lenster |  |  | 28.85±1.77 | 43.13±2.53 | 44.17±2.70 | 43.775±1.830 | - | - | 1.12±3.53 | 3.48±0.37 | - | - | - | - |
| Liampa et al. 2010 [71] | Switzerland | Cataract | Lenstar | 73.5±9.5 | 145 | 23.46±1.16 | - | - | 44.0±1.3 | - | - | - | 3.19±0.39 | - | - | - | - |
|  |  |  | IOLMaster |  |  | 23.49±1.15 | - | - | 44.06±1.30 | - | - | - | 2.99±0.39 | - | - | - | - |
| Liao et al. 2020 [72] | China | Healthy | IOLMaster 700 | 23.0±5.4 | 103 | 24.08±0.95 | 43.12±1.43 | 44.13±1.58 | 43.61±1.49 | 0.06±0.40 | 0.00±0.41 | 0.45±2.11 | 3.57±0.26 | 3.04±0.26 | 546.77±33.19 | 12.01±0.40 | 3.62±0.20 |
|  |  |  | OA-2000 |  |  | 24.08±0.95 | 43.07±1.45 | 44.14±1.59 | 43.61±1.50 | 0.03±0.41 | 0.01±0.44 | 0.6±2.3 | 3.57±0.26 | 3.02±0.26 | 529.69±31.67 | 11.87±0.53 | 3.7±0.2 |
| Mandal et al. 2013 [73] | Germany | mix | Aladdin | - | 97 | 23.65±1.36 | - | - | 43.80±1.47 | - | - | - | 3.28±0.47 | - | - | - | - |
|  |  |  | IOLMaster 500 |  |  | 23.64±1.36 | - | - | 43.84±1.41 | - | - | - | 3.28±0.43 | - | - | - | - |
| McAlinden et al. 2017 [74] | China | Healthy | Aladdin | 28.4±8.7 | 102 | 24.96±1.41 | 42.95±1.45 | 43.93±1.54 | 43.44±1.47 | −0.42±0.32 | 0.00±0.16 | 1.19±2.10 | 3.62±0.31 | - | - | 11.55±0.42 | - |
|  |  |  | Lenstar |  |  | 24.95±1.42 | 43.01±1.46 | 43.95±1.55 | 43.48±1.48 | −0.39±0.34 | −0.02±0.17 | 0.89±2.20 | 3.62±0.34 | - | - | 11.89±0.44 | - |
| McAlinden et al. 2016 [75] | China | Cataract | OA-2000 | 69.57±9.48 | 377 | 23.20±0.84 | - | - | - | - | - | - | - | - | - | - | - |
|  |  |  | IOLMaster |  | 241 | 23.21±0.82 | - | - | - | - | - | - | - | - | - | - | - |
|  |  |  | Aladdin |  | 326 | 23.22±0.82 | - | - | - | - | - | - | - | - | - | - | - |
| Moon et al. 2014 [76] | Korea | Cataract | AL-Scan | 67.4±9.6 | 98 | 23.56±1.15 | - | - | - | - | - | - | 3.14±0.39 | - | - | - | - |
|  |  |  | Contact ultrasound |  |  | 23.53±1.17 | - | - | - | - | - | - | 3.17±0.34 | - | - | - | -- |
| Mueller et al. 2016 [77] | Germany | Healthy | IOLMaster 500 | 37.9±16.2 | 123 | 23.80±1.14 | 42.880±1.125 | 43.83±1.16 | 43.38±1.16 | - | - | 0.95±1.57 | - | - | - | 12.19±0.44 | - |
|  |  |  | Lenstar | - |  | 23.84±1.15 | 42.830±1.125 | 43.72±1.16 | 43.27±1.16 | - | - | 1.05±1.61 | - | - | - | 12.27±0.45 | - |
| Muzyka-Woz´niak et al. 2018 [78] | Poland | Cataract | Pentacam AXL | 58 ± 15 | 87 | 23.27±1.83 | 43.10±1.16 | 44.180±1.125 | 43.600±1.205 | - | - | 1.11±1.67 | - | 3.02±0.39 | - | 11.5±0.4 | - |
|  |  |  | IOLMaster 500 |  |  | 23.29±1.82 | 43.38±1.16 | 44.470±1.089 | 43.95±1.16 | - | - | 1.01±1.63 | - | 3.01±0.39 | - | 11.9±0.5 | - |
| Nemethet al. 2003 [79] | Hungary | Mix | IOLMaster | 67.9±16.3 | 252 | 23.73±2.05 | - | - | - | - | - | - | 3.18±0.50 | - | - | - | - |
|  |  |  | Contact ultrasound |  |  | 23.34±1.95 | - | - | - | - | - | - | 2.91±0.50 | - | - | - | - |
| Olsen et al. 2007 [80] | Denmark | Cataract | IOLMaster | 76.50±8.42 | 461 | 23.45±1.04 | - | - | - | - | - | - | - | - | - | - | - |
|  |  |  | Contact ultrasound |  |  | 23.07±1.06 | - | - | - | - | - | - | - | - | - | - | - |
| Omoto et al. 2019 [81] | Japan | Cataract | Argos | 67.0 ± 9.9 | 106 | 25.14±1.90 | - | - | 44.06±1.2 | - | - | - | 3.33±0.42 | 2.82±0.41 | 533±32 | - | 4.47±0.44 |
|  |  |  | IOLMaster 700 |  |  | 25.22±1.95 | - | - | 43.89±1.2 | - | - | - | 3.23±0.42 | 2.62±0.43 | 559±32 | - | 4.46±0.43 |
| Pedro et al. 2020 [82] | Spain | Healthy | Anterion | 60.94±18.22 | 53 | - | - | - | - | - | - | - | - | - | - | 11.84±0.41 | - |
|  |  |  | IOLMaster 700 |  |  | - | - | - | - | - | - | - | - | - | - | 11.96±0.41 | - |
| Rabsilber et al. 2010 [83] | Germany | Cataract | Lenster | 70.0±10.6 | 100 | 23.55±1.38 | 43.44±1.125 | 44.35±1.089 | 43.89±1.125 | - | - | 0.88±1.62 | 3.09±0.40 | - | - | - | - |
|  |  |  | IOLMaster |  |  | 23.54±1.37 | 43.49±1.125 | 44.467±1.13 | 43.95±1.16 | - | - | 0.94±1.64 | 3.04±0.40 | - | - | - | - |
| Rajan et al. 2002 [84] | UK | Cataract | IOLMaster | 67±6 | 46 | 23.47±1.10 | - | - | - | - | - | - | - | - | - | - | - |
|  |  |  | Contact ultrasound | 71±8 | 50 | 23.43±1.20 | - | - | - | - | - | - | - | - | - | - | - |
|  |  | pseudophakic | IOLMaster | 67±6 | 46 | 23.35±1.10 | - | - | - | - | - | - | - | - | - | - | - |
|  |  |  | Contact ultrasound | 71±8 | 50 | 23.54±1.20 | - | - | - | - | - | - | - | - | - | - | - |
| Raymond et al. 2009 [85] | Austria | Cataract | IOLMaster | 73.71 | 84 | 23.39 | - | - | - | - | - | - | - | - | - | - | - |
|  |  |  | Contact ultrasound | 73.55 | 85 | 23.22 | - | - | - | - | - | - | - | - | - | - | - |
| Passi et al. 2018 [86] | USA | Cataract | IOLMaster 700 | 67±10 | 64 | 23.70±1.24 | 43.70±1.93 | 44.80±1.92 | 44.33±1.36 | - | - | 0.97±2.70 | 3.14±0.38 | - | - | - | 4.66±0.71 |
|  |  |  | Lenstar |  |  | 23.70±1.25 | 43.7±1.9 | 44.8±1.9 | 44.27±1.34 | - | - | 0.86±2.66 | 3.16±0.38 | - | - | - | 4.52±0.67 |
| Pereira et al. 2018 [87] | Portugal | Cataract | Lenstar | 76.2 ± 6.8 | 136 | 23.06±0.76 | 43.99±1.32 | 44.80±1.35 | 44.42±0.92 | - | - | 0.91±1.79 | 3.07±0.33 | 2.54±0.33 | 537.79±3.04 | - | - |
|  |  |  | Pentacam AXL |  |  | 23.03±0.35 | 43.86±1.30 | 44.56±1.36 | 44.15±1.00 | - | - | 0.81±1.86 | 3.06±0.37 | 2.52±0.35 | 528.7±2.89 | - | - |
| Reddy et al. 2004 [88] | England | Cataract | IOLMaster | 72 | 81 | - | - | - | - | - | - | - | 3.33±0.61 | - | - | - | - |
|  |  |  | Contact ultrasound |  | 76 | - | - | - | - | - | - | - | 2.87±0.55 | - | - | - | - |
| Reitblat et al. 2018 [89] | Israel | Cataract | OA-2000 | 64.2±9.8 | 140 | 24.72±1.67 | - | - | 43.83±1.42 | - | - | 1.51±1.14 | 3.39±0.42 | - | - | 12.05±0.45 | 4.46±0.49 |
|  |  |  | IOLMaster 500 |  |  | 24.72±1.68 | - | - | 43.83±1.41 | - | - | 1.56±1.18 | 3.34±0.42 | - | - | 12.09±0.42 | - |
|  |  |  | Lenstar |  |  | 24.75±1.69 | - | - | 43.76±1.40 | - | - | 1.52±1.17 | 3.34±0.40 | - | - | 12.17±0.43 | 4.41±0.47 |
| Rohrer et al. 2009 [90] | Switzerland | Mix | Lenstar | 66.9 | 144 | 24.1±2.2 | 43.60±1.25 | 44.82±1.09 | 44.15±0.83 | - | - | 1.29±1.71 | 3.19±0.48 | - | - | - | - |
|  |  |  | IOLMaster |  |  | 24.1±2.2 | 43.66±1.25 | 44.820±1.125 | 44.26±0.82 | - | - | 1.08±1.68 | 3.17±0.49 | - | - | - | - |
| Rose et al. 2003 [91] | Austria | Cataract | IOLMaster | 72 | 51 | 23.36±1.24 | - | - | - | - | - | - | - | - | - | - | - |
|  |  |  | Contact ultrasound |  |  | 23.21±1.30 | - | - | - | - | - | - | - | - | - | - | - |
| Ruiz-Mesa et al. 2017 [92] | Spain | Cataract | Pentacam AXL | 64.35±10.46 | 40 | 23.66±1.61 | - | - | 43.42±1.42 | - | - | - | 3.18±0.46 | 2.65±0.45 | 542.0±27.7 | - | - |
|  |  |  | Lenstar |  |  | 23.646±1.610 | - | - | 43.46±1.38 | - | - | - | 3.22±0.43 | 2.69±0.44 | 548.3±29.2 | - | - |
|  |  | Healthy | Pentacam AXL | 34.15±8.63 | 40 | 24.701±1.600 | - | - | 43.89±1.30 | - | - | - | 3.69±0.42 | 3.14±0.43 | 525.8±37.1 | - | - |
|  |  |  | Lenstar |  |  | 24.833±1.630 | - | - | 44.06±1.31 | - | - | - | 3.67±0.42 | 3.15±0.44 | 529.3±37.1 | - | - |
| Sabatino et al. 2016 [93] | UK | Cataract | IOLMaster | - | 215 | 23.67±1.04 | - | - | 43.48±1.65 | - | - | - | 3.13±0.36 | - | - | 12.08±0.38 | - |
|  |  |  | Aladdin |  |  | 23.71±1.03 | - | - | 43.32±1.60 | - | - | - | 3.16±0.30 | - | - | 11.69±0.40 | - |
| Sabatino et al. 2019 [94] | UK | Cataract | IOLMaster 700 | 67.9±10.4 | 218 | 23.79±1.30 | - | - | 43.71±1.05 | - | - | - | 3.19±0.44 | 2.63±0.45 | 535.11±40.83 | 12.00±0.55 | 4.38±0.47 |
|  |  |  | Argos |  |  | 23.78±1.26 | - | - | 43.760±1.035 | - | - | - | 3.31±0.43 | 2.75±0.44 | 530.53±39.18 | 12.45±0.54 | 4.46±0.48 |
| Salouti et al. 2020 [95] | Iran | Healthy | Pentacam AXL | 29±5 | 97 | 24.53±0.72 | - | - | 43.81±1.16 | −0.60±0.43 | −0.07±0.25 | - | 3.28±0.28 | 2.73±0.26 | 545.45±27.3 | 11.86±0.39 | - |
|  |  |  | IOLMaster 700 |  |  | 24.55±0.72 | - | - | 43.98±1.17 | −0.57±0.44 | −0.04±0.24 | - | 3.23±0.28 | 2.68±0.28 | 548.42±28.20 | 12.08±0.38 | - |
| Saucedo-Urdapilleta et al. 2019 [96] | Mexico | Cataract | IOLMaster 500 | 69.00±11.12 | 55 | 23.94±2.16 | - | - | 43.55±1.65 | - | - | - | 3.08±0.43 | - | - | 11.93±0.47 | - |
|  |  |  | IOLMaster 700 |  |  | 24.10±2.44 | - | - | 43.61±1.60 | - | - | - | 3.11±0.43 | - | - | 11.78±0.52 | - |
| Savini et al. 2009 [97] | American | Cataract | immersion ultrasound | 76.5±8.4 | 41 | 23.46±1.49 | - | - | - | - | - | - | - | - | - | - | - |
|  |  |  | IOLMaster |  |  | 23.5±1.5 | - | - | 43.97±1.44 | - | - | - | - | - | - | - | - |
| Schultz et al. 2016 [98] | Germany | Cataract | IOLMaster 500 | 68.5±11.9 | 105 | - | 41.97±1.04 | 43.342±1.110 | 42.67±0.76 | - | - | 1.46±1.50 | - | - | - | - | - |
|  |  |  | AL-Scan |  |  | - | 42.03±1.07 | 43.29±1.11 | 42.63±0.81 | - | - | 1.24±1.59 | - | - | - | - | - |
| Sel et al. 2017 [99] | Germany | Healthy | Pentacam AXL | 38.52±12.90 | 50 | 23.56±1.10 | - | - | 43.76±1.24 | 0.49±0.43 | −0.06±0.22 | - | 3.40±0.33 | - | - | - | - |
|  |  |  | IOLMaster 700 |  |  | 23.61±1.07 | - | - | 47.12±1.24 | 0.47±0.41 | −0.076±0.220 | - | 3.35±0.33 | - | - | - | - |
| Shajari et al. 2017 [100] | Germany | Healthy | IOLMaster 500 | - | 79 | - | - | - | - | - | - | 0.94±0.67 | - | - | - | - | - |
|  |  |  | Pentacam AXL |  |  | - | - | - | - | - | - | 0.91±0.66 | - | - | - | - | - |
|  |  |  | IOLMaster 700 |  |  | - | - | - | - | - | - | 0.96±0.67 | - | - | - | - | - |
| Shajari et al. 2016 [101] | Germany | Healthy | IOLMaster 500 | 36.50±15.50 | 40 | - | - | - | - | - | - | - | 3.5±0.4 | 2.9±0.4 | - | 12.0±0.3 | - |
|  |  |  | Lenstar |  |  | - | - | - | - | - | - | - | 3.5±0.4 | 2.9±0.4 | - | 12.3±0.4 | - |
| Shammas et al. 2016 [102] | USA | Cataract | Argos | 74±9 | 107 | 23.35±0.92 | - | - | 44.00±1.05 | - | - | - | 3.08±0.34 | 2.55±0.33 | 530±30 | 11.26±0.59 | 4.73±0.41 |
|  |  |  | IOLMaster 500 |  | 91 | 23.32±0.91 | - | - | 44.06±1.022 | - | - | - | 2.88±0.33 | - | - | - | -- |
|  |  |  | Lenstar |  | 107 | 23.40±0.92 | - | - | 44.00±1.05 | - | - | - | 3.11±0.37 | 2.58±0.36 | 530±40 | 11.82±0.57 | 4.53±0.39 |
| Shen et al. 2014 [103] | China | Child | Lenstar | 10.9±2.0 | 110 | 23.90±1.28 | 43.44±1.25 | 44.53±1.25 | 44.05±0.91 | - | - | 1.02±1.78 | 3.62±0.26 | - | - | 12.08±0.46 | - |
|  |  |  | IOLMaster |  |  | 23.88±1.27 | 43.44±1.25 | 44.64±1.25 | 44.08±0.89 | - | - | 1.13±1.78 | 3.58±0.25 | - | - | 12.09±0.43 | - |
| Shen et al. 2019 [104] | China | 26<AL≤28 mm | IOLMaster 500 | 59.5±20.6 | 73 | 26.88±0.60 | 43.30±1.49 | 44.34±1.80 | 43.90±1.15 | - | - | 1.05±2.26 | 3.46±0.40 | - | - | - | - |
|  |  |  | IOLMaster 700 |  |  | 26.89±0.60 | 43.26±1.49 | 44.26±1.78 | 43.8±1.2 | - | - | 0.95±2.40 | 3.43±0.39 | - | - | - | - |
|  |  | 28<AL<30 mm | IOLMaster 500 |  | 46 | 28.95±0.66 | 43.17±1.60 | 44.43±1.64 | 43.82±1.10 | - | - | 1.21±2.37 | 3.5±0.421 | - | - | - | - |
|  |  |  | IOLMaster 700 |  |  | 29.04±0.59 | 43.07±1.51 | 44.34±1.57 | 43.60±1.09 | - | - | 1.26±2.02 | 3.43±0.40 | - | - | - | - |
|  |  | AL≥30 mm | IOLMaster 500 |  | 33 | 32.12±1.49 | 43.25±1.53 | 44.76±1.74 | 44.08±1.15 | - | - | 1.39±2.33 | 3.57±0.30 | - | - | - | - |
|  |  |  | IOLMaster 700 |  |  | 32.13±1.47 | 43.30±1.57 | 44.64±1.84 | 44.0±1.2 | - | - | 1.12±2.40 | 3.52±0.27 | - | - | - | - |
| Shin et al. 2016 [105] | South Korea | Cataract | Galilei G6 | 65.3±10.7 | 140 | 23.34±0.99 | - | - | 44.64±1.48 | - | - | - | 3.24±0.45 | - | - | 11.61±0.42 | 4.42±0.47 |
|  |  |  | Lenstar |  |  | 23.38±1.01 | - | - | 44.59±1.46 | - | - | - | 3.25±0.46 | - | - | 11.45±0.43 | 4.31±0.49 |
| Shu et al. 2020 [106] | China | Healthy | OA-2000 | 11.98±2.15 | 51 | 25.15±0.99 | - | - | 43.15±1.45 | - | - | - | 3.73±0.19 | - | - | 11.96±0.43 | - |
|  |  |  | IOLMaster |  |  | 25.14±0.96 | - | - | 43.22±1.41 | - | - | - | 3.62±0.20 | - | - | 12.14±0.58 | - |
| Song et al. 2020 [107] | USA | Cataract | IOLMaster 500 | 65±10 | 112 | 24.12±1.74 | - | - | 44.23±1.48 | - | - | - | 3.04±0.46 | - | - | - | - |
|  |  |  | IOLMaster 700 |  |  | 24.12±1.73 | - | - | 44.12±1.51 | - | - | - | 3.15±0.48 | - | - | - | - |
|  |  |  | Lenstar |  |  | 24.13±1.75 | - | - | 44.16±1.49 | - | - | - | 3.16±0.44 | - | - | - | - |
| Srivannaboon et al. 2014 [108] | Thailand | Cataract | AL-Scan | 65.28±10.56 | 137 | 23.51±1.02 | - | - | 44.31±1.63 | - | - | - | 3.11±0.44 | - | - | 11.51±0.75 | - |
|  |  |  | IOLMaster 500 |  |  | 23.50±1.02 | - | - | 44.22±1.6 | - | - | - | 3.14±0.43 | - | - | 12.03±0.44 | - |
| Srivannaboon et al. 2015 [109] | Thailand | Cataract | AL-Scan | - | 137 | - | - | - | 44.31±1.63 | 0.40±0.84 | -0.02±0.43 | 0.85±0.58 | - | - | - | - | - |
|  |  |  | IOLMaster 500 |  |  | - | - | - | 44.22±1.60 | 0.40±0.91 | 0.01±0.42 | 0.88±0.63 | - | - | - | - | - |
| Srivannaboon et al. 2015 [110] | Thailand | Cataract | IOLMaster 700 | 64.53±11.20 | 91 | 23.5±1.18 | - | - | 44.46±1.46 | - | - | - | 2.98±0.38 | - | - | 11.85±0.41 | - |
|  |  |  | IOLMaster 500 |  |  | 23.48±1.17 | - | - | 44.47±1.45 | - | - | - | 2.94±0.42 | - | - | 11.95±0.39 | - |
| Su et al. 2008 [111] | China | Healthy | IOLMaster | 51.5±17.9 | 90 | - | - | - | - | - | - | - | 3.20±0.45 | - | - | - | - |
|  |  |  | Contact ultrasound |  |  | - | - | - | - | - | - | - | 3.12±0.44 | - | - | - | - |
|  |  | Pseudophakic | IOLMaster |  | 94 | - | - | - | - | - | - | - | 4.06±0.46 | - | - | - | - |
|  |  |  | Contact ultrasound |  |  | - | - | - | - | - | - | - | 3.81±0.41 | - | - | - | - |
|  |  | Phakic | IOLMaster |  | 90 | - | - | - | - | - | - | - | 3.20±0.45 | - | - | - | - |
|  |  |  | Contact ultrasound |  |  | - | - | - | - | - | - | - | 3.12±0.44 | - | - | - | - |
| Suto et al. 2015 [112] | Japan | Cataract | AL-Scan | 72.1±9 | 450 | 23.64±1.34 | - | - | 44.63±1.54 | - | - | - | 3.01±0.44 | - | - | - | - |
|  |  |  | IOLMaster 500 |  |  | 23.66±1.34 | - | - | 44.58±1.56 | - | - | - | 3.02±0.42 | - | - | - | - |
| Tu et al. 2020 [113] | China | Healthy | Pentacam AXL | 37.55±18.21 | 145 | 24.74±1.45 | - | - | 43.44±1.40 | −0.34±0.43 | −0.01±0.19 | - | 3.51±0.44 | 3.00±0.43 | 536.47±30.00 | - | - |
|  |  |  | Argos |  |  | 24.76±1.42 | - | - | 43.72±1.39 | −0.35±0.47 | 0.02±0.20 | - | 3.56±0.43 | 3.04±0.42 | 535.31±30.13 | - | - |
| Ueda et al. 2007 [114] | Japan | Cataract | IOLMaster | 74.5±8 | 59 | 23.33±1.49 | - | - | - | - | - | - | - | - | - | - | - |
|  |  |  | Contact ultrasound |  |  | 23.12±1.46 | - | - | - | - | - | - | - | - | - | - | - |
| Vasavada et al. 2020 [115] | India | Cataract | Lenstar | 58.40±11.14 | 124 | 23.72±1.58 | 43.44±1.89 | 44.37±1.94 | 43.90±1.89 | - | - | 0.92±3.72 | 3.16±0.36 | 2.63±0.35 | 525.64±27.04 | - | 4.18±0.48 |
|  |  |  | A-2000 |  |  | 23.78±1.76 | 43.54±1.86 | 44.42±1.93 | 43.97±1.86 | - | - | 0.87±2.69 | 3.15±0.37 | 2.63±0.36 | 513.21±29.24 | - | 4.44±0.44 |
| Ventura et al. 2017 [116] | Brazil | Cataract | Galilei G6 | 66.81±7.2 | 88 | 23.58±1.12 | 43.6±1.62 | 44.43±1.63 | 44.01±1.60 | - | - | 1.05±2.29 | 3.17±0.42 | - | - | - | - |
|  |  |  | IOLMaster 500 |  |  | 23.56±1.10 | 43.63±1.66 | 44.49±1.60 | 44.06±1.61 | - | - | 0.81±2.38 | 3.17±0.40 | - | - | - | - |
| Visser et al. 2012 [117] | Netherlands | Healthy | IOLMaster | 26.6±9.0 | 30 | - | - | - | - | - | - | 0.51±0.63 | - | - | - | - | - |
|  |  |  | Lenstar |  |  | - | - | - | - | - | - | 0.53±0.63 | - | - | - | - | - |
| Wang et al. 2017 [118] | China | AL≤22 mm | IOLMaster | - | 20 | 21.46±0.40 | - | - | - | - | - | - | 2.82±0.41 | - | - | - | - |
|  |  |  | Contact ultrasound |  |  | 21.48±0.41 | - | - | - | - | - | - | 2.81±0.35 | - | - | - | - |
|  |  | 22<AL≤24.5mm | IOLMaster |  | 78 | 23.14±0.63 | - | - | - | - | - | - | 3.10±0.47 | - | - | - | - |
|  |  |  | Contact ultrasound |  |  | 23.13±0.62 | - | - | - | - | - | - | 3.04±0.50 | - | - | - | - |
|  |  | 24.5<AL≤26mm | IOLMaster |  | 34 | 25.27±0.59 | - | - | - | - | - | - | 3.60±0.52 | - | - | - | - |
|  |  |  | Contact ultrasound |  |  | 25.24±0.56 | - | - | - | - | - | - | 3.55±0.62 | - | - | - | - |
|  |  | 26<AL≤28 mm | IOLMaster |  | 31 | 27.03±0.64 | - | - | - | - | - | - | 3.51±0.30 | - | - | - | - |
|  |  |  | Contact ultrasound |  |  | 26.97±0.59 | - | - | - | - | - | - | 3.42±0.24 | - | - | - | - |
|  |  | AL＞28 mm | IOLMaster |  | 26 | 31.01±1.53 | - | - | - | - | - | - | 3.61±0.34 | - | - | - | - |
|  |  |  | Contact ultrasound |  |  | 30.76±1.40 | - | - | - | - | - | - | 3.50±0.28 | - | - | - | - |
| Wang et al. 2019 [119] | China | Cataract | Pentacam AXL | 64±11 | 223 | 23.53±1.17 | - | - | 43.98±1.56 | - | - | - | 3.09±0.42 | - | - | 11.36±0.42 | - |
|  |  |  | IOLMaster 700 |  |  | 23.57±1.16 | - | - | 44.17±1.60 | - | - | - | 3.06±0.42 | - | - | 11.69±0.45 | - |
|  |  |  | IOLMaster 500 |  |  | 23.54±1.15 | - | - | 44.19±1.57 | - | - | - | 3.05±0.41 | - | - | 11.45±0.42 | - |
| Wang et al. 2008 [120] | China | AL≥25 mm, Cataract | IOLMaster |  | 68 | 28.06±1.88 | - | - | - | - | - | - | - | - | - | - | - |
|  |  |  | Contact ultrasound |  |  | 27.96±1.75 | - | - | - | - | - | - | - | - | - | - | - |
| Wang et al. 2016 [121] | China | Healthy | Lenstar | 58±17 | 84 | 23.17±0.78 | - | - | - | - | - | - | - | - | - | - | - |
|  |  |  | IOLMaster |  |  | 23.18±0.77 | - | - | - | - | - | - | - | - | - | - | - |
|  |  |  | Contact ultrasound |  |  | 22.94±0.75 | - | - | - | - | - | - | - | - | - | - | - |
|  |  | High myopia | Lenstar | 50±20 | 49 | 26.74±2.04 | - | - | - | - | - | - | - | - | - | - | - |
|  |  |  | IOLMaster |  |  | 26.73±2.05 | - | - | - | - | - | - | - | - | - | - | - |
|  |  |  | Contact ultrasound |  |  | 26.49±1.98 | - | - | - | - | - | - | - | - | - | - | - |
| Whang et al. 2018 [122] | South Korea | Cataract | IOLMaster 500 | 64.84±8.76 | 153 | 24.65±2.35 | - | - | 44.19±1.47 | - | - | - | 3.30±0.46 | - | - | - | - |
|  |  |  | Argos |  |  | 24.62±2.29 | - | - | 44.18±1.45 | - | - | - | 3.31±0.46 | - | - | - | - |
| Wissa et al. 2012 [123] | Egypt | Cataract | IOLMaster | 64.3±10.9 | 163 | 24.05±2.76 | - | - | - | - | - | - | 3.31±0.45 | - | - | - | - |
|  |  |  | Contact ultrasound |  |  | 24.45±2.73 | - | - | - | - | - | - | 3.32±0.46 | - | - | - | - |
| Yang et al. 2017 [124] | South Korea | Mix | IOLMaster 700 | 59.49±16.73 | 219 | 27.11±3.00 | - | - | - | - | - | - | - | - | - | - | - |
|  |  |  | IOLMaster |  |  | 27.05±2.99 | - | - | - | - | - | - | - | - | - | - | - |
| Yeu et al. 2019 [125] | USA | Cataract | Aladdin | 67.9±7.2 | 101 | 23.88±1.23 | 43.63±1.65 | 44.59±1.76 | 44.01±1.28 | - | - | 0.94±2.36 | 3.24±0.36 | 2.71±0.36 | 540±40 | 11.63±0.41 | 4.61±0.39 |
|  |  |  | Lenstar |  |  | 23.89±1.23 | 43.61±1.67 | 44.61±1.77 | 44.09±1.21 | - | - | 1.01±2.39 | 3.25±0.37 | 2.69±0.38 | 550±40 | 12.03±0.49 | 4.45±0.45 |
| Yu et al. 2017 [126] | China | Cataract | Lenstar | 64.74±8.65 | 125 | - | 43.95±1.40 | 44.89±1.40 | 44.42±1.38 | 0.11±0.46 | −0.02±0.28 | 0.93±2.11 | - | - | - | - | - |
|  |  |  | IOLMaster |  |  | - | 44.00±1.38 | 44.92±1.42 | 44.46±1.37 | 0.08±0.43 | −0.02±0.24 | 0.95±1.97 | - | - | - | - | - |
| Zhang et al. 2016 [127] | China | Cataract | Lenstar | 72.6±8.4 | 158 | 24. 52±1. 73 | - | - | 43.86±1.45 | - | - | - | 2.71±0.38 | 2.18±0.40 | 536.54±27.90 | - | - |
|  |  |  | Contact ultrasound |  |  | 24. 28±1. 70 | - | - | - | - | - | - | 2.85±0.40 | - | - | - | - |
| Zhang et al. 2015 [128] | China | Healthy | IOLMaster | 25.0±3.6 | 138 | - | - | - | - | - | - | - | 3.73±0.23 | - | - | - | - |
|  |  |  | Contact ultrasound |  |  | - | - | - | - | - | - | - | 3.69±0.22 | - | - | - | - |
| Zhao et al. 2013 [129] | China | Healthy | Lenstar | 26.7±6.1 | 56 | 26.18±1.30 | 42.93±1.41 | 43.86±1.74 | 43.30±1.12 | - | - | 0.86±2.25 | 3.73±0.27 | 3.18±0.29 | 546.61±35 | 12.01±0.47 | - |
|  |  |  | IOLMaster |  |  | 26.06±1.26 | 42.94±1.35 | 44.11±1.64 | 43.58±1.07 | - | - | 1.14±2.07 | 3.74±0.29 | - | - | 11.94±0.32 | - |

*AL* = axial length; *Km* = keratometry in the steepest meridian; *Kf* = keratometry in the flattest meridian; *Ks* = mean keratometry; *J0* = anterior corneal power vectors for the cardinal (axes at 90 degrees and 180 degrees) meridians; *J45* = anterior corneal power vectors for the oblique (axes at 45 degrees and 135 degrees) meridians; *AST* = astigmatism; *ACD* = anterior chamber depth; *AQD* = aqueous depth; *CCT* = central corneal thickness; *CD* = corneal diameter; *LT* = lens thickness

Appendix I Table 2. The risk of bias of the trials included in the current study

| Item | Was the spectrum of patients representative of the patients who will receive the test in practice? | Were selection criteria clearly described? | Is the time period between reference standard and index test short enough to be reasonably sure that the target condition did not change between the two tests? | Did the whole sample or a random selection of the sample, receive verification using a reference standard of diagnosis? | Did patients receive the same reference standard regardless of the index test result? | Was the reference standard independent of the index test (i.e. the index test did not form part of the reference standard)? | Were the index test results interpreted without knowledge of the results of the reference standard? | Were the reference standard results interpreted without knowledge of the results of the index test? | Were the same clinical data available when test results were interpreted as would be available when the test is used in practice? | Were uninterpretable/ intermediate test results reported? | Were withdrawals from the study explained? |
| --- | --- | --- | --- | --- | --- | --- | --- | --- | --- | --- | --- |
| Akman et al. 2016 [1] | Yes | Yes | Yes | Yes | Yes | Yes | Yes | Yes | Yes | Yes | Yes |
| Aktas et al. 2015 [2] | Yes | Yes | Not Clear | Yes | Yes | Yes | Yes | Yes | Yes | Yes | Yes |
| Alvani et al. 2016 [3] | Yes | Yes | Not Clear | Yes | Yes | Yes | Yes | Yes | Yes | Yes | Yes |
| An et al. 2019 [4] | Yes | Yes | Yes | Yes | Yes | Yes | Yes | Yes | Yes | Yes | Yes |
| Arriola-Villalobos et al. 2017 [5] | Yes | Yes | Yes | Yes | Yes | Yes | Yes | Yes | Yes | Yes | Yes |
| Asena et al. 2018 [6] | Yes | Yes | Yes | Yes | Yes | Yes | Yes | Yes | Yes | Yes | Yes |
| Attas-Fox et al. 2007 [7] | Yes | Yes | Yes | Yes | Yes | Yes | Yes | Yes | Yes | Yes | Yes |
| Aydin et al. 2016 [8] | Yes | Yes | Not clear | Yes | Yes | Yes | Yes | Yes | Yes | Yes | Yes |
| Bai et al. 2007 [9] | Yes | Yes | Not clear | Yes | Yes | Yes | Yes | Yes | Yes | Yes | Yes |
| Bi et al. 2020 [10] | Yes | Yes | Yes | Yes | Yes | Yes | Yes | Yes | Yes | Yes | Yes |
| Buckhurst et al. 2009 [11] | Yes | Yes | Not clear | Yes | Yes | Yes | Yes | Yes | Yes | Yes | Yes |
| Bullimore et al. 2019 [12] | Yes | Yes | Yes | Yes | Yes | Yes | Yes | Yes | Yes | Yes | Yes |
| Caglar et al. 2016 [13] | Yes | Yes | Yes | Yes | Yes | Yes | Yes | Yes | Yes | Yes | Yes |
| Calvo-Sanz et al. 2018 [14] | Yes | Yes | Not clear | Yes | Yes | Yes | Yes | Yes | Yes | Yes | Yes |
| Can et al. 2019 [15] | Yes | Not clear | Not clear | Yes | Yes | Yes | Yes | Yes | Yes | Yes | Yes |
| Can et al. 2016 [16] | Yes | Not clear | Not clear | Yes | Yes | Yes | Yes | Yes | Yes | Yes | Yes |
| Carkeet et al. 2004 [17] | Yes | No | Not clear | Yes | Yes | Yes | Yes | Yes | Yes | Yes | Yes |
| Cech et al. 2014 [18] | Yes | No | Not clear | Yes | Yes | Yes | Yes | Yes | Yes | Yes | Yes |
| Can et al. 2019 [19] | Yes | Not clear | Not clear | Yes | Yes | Yes | Yes | Yes | Yes | Yes | Yes |
| Chen et al. 2011 [20] | Yes | Yes | Not clear | Yes | Yes | Yes | Yes | Yes | Yes | Yes | Yes |
| Cheng et al. 2019 [21] | Yes | Yes | Yes | Yes | Yes | Yes | Yes | Yes | Yes | Yes | Yes |
| Cho et al. 2018 [22] | Yes | Yes | Not Clear | Yes | Yes | Yes | Yes | Yes | Yes | Yes | Yes |
| Cinar et al. 2013 [23] | Yes | Yes | Not Clear | Yes | Yes | Yes | Yes | Yes | Yes | Yes | Yes |
| Cruysberg et al. 2009 [24] | Yes | Yes | Not Clear | Yes | Yes | Yes | Yes | Yes | Yes | Yes | Yes |
| Cvetkovic et al. 2016 [25] | Yes | No | Not Clear | Yes | Yes | Yes | Yes | Yes | Yes | Yes | Yes |
| Doğan et al. 2014 [26] | Yes | No | Not Clear | Yes | Yes | Yes | Yes | Yes | Yes | Yes | Yes |
| Du et al. 2019 [27] | Yes | No | Not Clear | Yes | Yes | Yes | Yes | Yes | Yes | Yes | Yes |
| Einan-Lifshitz et al. 2017 [28] | Yes | Not clear | Not Clear | Yes | Yes | Yes | Yes | Yes | Yes | Yes | Yes |
| El-Baha et al. 2009 [29] | Yes | Yes | Not Clear | Yes | Yes | Yes | Yes | Yes | Yes | Yes | Yes |
| Elbaz et al. 2007 [30] | Yes | Yes | Not Clear | Yes | Yes | Yes | Yes | Yes | Yes | Yes | Yes |
| El Chehab et al. 2018 [31] | Yes | Not clear | Not Clear | Yes | Yes | Yes | Yes | Yes | Yes | Yes | Yes |
| Eleftheriadis et al. 2003 [32] | Yes | Yes | Not Clear | Yes | Yes | Yes | Yes | Yes | Yes | Yes | Yes |
| Epitropoulos et al. 2014 [33] | Yes | Yes | Not Clear | Yes | Yes | Yes | Yes | Yes | Yes | Yes | Yes |
| Findl et al. 2003 [34] | Yes | Yes | Yes | Yes | Yes | Yes | Yes | Yes | Yes | Yes | Yes |
| Fișuș et al. 2020 [35] | Yes | Not clear | Yes | Yes | Yes | Yes | Yes | Yes | Yes | Yes | Yes |
| Fontes et al. 2011 [36] | Yes | Yes | Yes | Yes | Yes | Yes | Yes | Yes | Yes | Yes | Yes |
| Gao et al. 2017 [37] | Yes | Not clear | Not Clear | Yes | Yes | Yes | Yes | Yes | Yes | Yes | Yes |
| Ghaffari et al. 2019 [38] | Yes | Not clear | Yes | Yes | Yes | Yes | Yes | Yes | Yes | Yes | Yes |
| Goebels et al. 2015 [39] | Yes | Not clear | Not Clear | Yes | Yes | Yes | Yes | Yes | Yes | Yes | Yes |
| Goto et al. 2020 [40] | Yes | Not clear | Yes | Yes | Yes | Yes | Yes | Yes | Yes | Yes | Yes |
| Goyal et al. 2003 [41] | Yes | Not clear | Not Clear | Yes | Yes | Yes | Yes | Yes | Yes | Yes | Yes |
| Güler et al. 2016 [42] | Yes | Yes | Yes | Yes | Yes | Yes | Yes | Yes | Yes | Yes | Yes |
| Guo et al. 2019 [43] | Yes | Not clear | Yes | Yes | Yes | Yes | Yes | Yes | Yes | Yes | Yes |
| Gursoy et al. 2011 [44] | Yes | Not clear | Not Clear | Yes | Yes | Yes | Yes | Yes | Yes | Yes | Yes |
| Haddad et al. 2020 [45] | Yes | Not clear | Yes | Yes | Yes | Yes | Yes | Yes | Yes | Yes | Yes |
| Hashemi et al. 2005 [46] | Yes | Yes | Yes | Yes | Yes | Yes | Yes | Yes | Yes | Yes | Yes |
| Henriquez et al. 2020 [47] | Yes | Yes | Yes | Yes | Yes | Yes | Yes | Yes | Yes | Yes | Yes |
| Hoffer et al. 2016 [48] | Yes | Yes | Yes | Yes | Yes | Yes | Yes | Yes | Yes | Yes | Yes |
| Hoffer et al. 2016 [49] | Yes | Yes | Yes | Yes | Yes | Yes | Yes | Yes | Yes | Yes | Yes |
| Hoffer et al. 2010 [50] | Yes | No | Yes | Yes | Yes | Yes | Yes | Yes | Yes | Yes | Yes |
| Hoffer et al. 2016 [51] | Yes | Yes | Yes | Yes | Yes | Yes | Yes | Yes | Yes | Yes | Yes |
| Holzer et al. 2009 [52] | Yes | No | Yes | Yes | Yes | Yes | Yes | Yes | Yes | Yes | Yes |
| Hua et al. 2018 [53] | Yes | Yes | Yes | Yes | Yes | Yes | Yes | Yes | Yes | Yes | Yes |
| Huang et al. 2012 [54] | Yes | Yes | Yes | Yes | Yes | Yes | Yes | Yes | Yes | Yes | Yes |
| Huang et al. 2019 [55] | Yes | Yes | Yes | Yes | Yes | Yes | Yes | Yes | Yes | Yes | Yes |
| Huang et al. 2016 [56] | Yes | Yes | Yes | Yes | Yes | Yes | Yes | Yes | Yes | Yes | Yes |
| Hui et al. 2014 [57] | Yes | No | Yes | Yes | Yes | Yes | Yes | Yes | Yes | Yes | Yes |
| Hussaindeen et al. 2018 [58] | Yes | Yes | Yes | Yes | Yes | Yes | Yes | Yes | Yes | Yes | Yes |
| Jung et al. 2017 [59] | Yes | Yes | Yes | Yes | Yes | Yes | Yes | Yes | Yes | Yes | Yes |
| Kaswin et al. 2014 [60] | Yes | Yes | Yes | Yes | Yes | Yes | Yes | Yes | Yes | Yes | Yes |
| Kaya et al. 2016 [61] | Yes | Yes | Yes | Yes | Yes | Yes | Yes | Yes | Yes | Yes | Yes |
| Khan et al. 2019 [62] | Yes | Yes | Yes | Yes | Yes | Yes | Yes | Yes | Yes | Yes | Yes |
| Kongsap et al. 2016 [63] | Yes | Yes | Yes | Yes | Yes | Yes | Yes | Yes | Yes | Yes | Yes |
| Kunert et al. 2016 [64] | Yes | Yes | Yes | Yes | Yes | Yes | Yes | Yes | Yes | Yes | Yes |
| Kurian et al. 2016 [65] | Yes | Yes | Yes | Yes | Yes | Yes | Yes | Yes | Yes | Yes | Yes |
| Lam et al. 2001 [66] | Yes | No | Yes | Yes | Yes | Yes | Yes | Yes | Yes | Yes | Yes |
| Li et al. 2018 [67] | Yes | Yes | Yes | Yes | Yes | Yes | Yes | Yes | Yes | Yes | Yes |
| Li et al. 2016 [68] | Yes | Yes | Yes | Yes | Yes | Yes | Yes | Yes | Yes | Yes | Yes |
| Li et al. 2020 [69] | Yes | Not clear | Yes | Yes | Yes | Yes | Yes | Yes | Yes | Yes | Yes |
| Liampa et al. 2010 [70] | Yes | Yes | Yes | Yes | Yes | Yes | Yes | Yes | Yes | Yes | Yes |
| Liao et al. 2020 [71] | Yes | Yes | Yes | Yes | Yes | Yes | Yes | Yes | Yes | Yes | Yes |
| Mandal et al. 2013 [72] | Yes | No | Yes | Yes | Yes | Yes | Yes | Yes | Yes | Yes | Yes |
| McAlinden et al. 2017 [73] | Yes | Yes | Yes | Yes | Yes | Yes | Yes | Yes | Yes | Yes | Yes |
| McAlinden et al. 2016 [74] | Yes | Yes | Yes | Yes | Yes | Yes | Yes | Yes | Yes | Yes | Yes |
| Moon et al. 2014 [75] | Yes | Yes | Yes | Yes | Yes | Yes | Yes | Yes | Yes | Yes | Yes |
| Mueller et al. 2016 [76] | Yes | Yes | Yes | Yes | Yes | Yes | Yes | Yes | Yes | Yes | Yes |
| Muzyka-Woz´niak et al. 2018 [77] | Yes | Yes | Yes | Yes | Yes | Yes | Yes | Yes | Yes | Yes | Yes |
| Nemeth et al. 2003 [78] | Yes | No | Yes | Yes | Yes | Yes | Yes | Yes | Yes | Yes | Yes |
| Olsen et al. 2007 [79] | Yes | Yes | Yes | Yes | Yes | Yes | Yes | Yes | Yes | Yes | Yes |
| Omoto et al. 2019 [80] | Yes | Yes | Yes | Yes | Yes | Yes | Yes | Yes | Yes | Yes | Yes |
| Pedro et al. 2020 [81] | Yes | Yes | Yes | Yes | Yes | Yes | Yes | Yes | Yes | Yes | Yes |
| Rabsilber et al. 2010 [82] | Yes | Yes | Yes | Yes | Yes | Yes | Yes | Yes | Yes | Yes | Yes |
| Rajan et al. 2002 [83] | Yes | Yes | Yes | No | No | Yes | Yes | Yes | Yes | Yes | Yes |
| Rajan et al. 2002 [84] | Yes | Yes | Yes | No | No | Yes | Yes | Yes | Yes | Yes | Yes |
| Raymond et al. 2009 [85] | Yes | No | Yes | No | No | Yes | Yes | Yes | Yes | Yes | Yes |
| Passi et al. 2018 [86] | Yes | Yes | Yes | Yes | Yes | Yes | Yes | Yes | Yes | Yes | Yes |
| Pereira et al. 2018 [87] | Yes | Yes | Yes | Yes | Yes | Yes | Yes | Yes | Yes | Yes | Yes |
| Reddy et al. 2004 [88] | Yes | No | Yes | Yes | Yes | Yes | Yes | Yes | Yes | Yes | Yes |
| Reitblat et al. 2018 [89] | Yes | Yes | Yes | Yes | Yes | Yes | Yes | Yes | Yes | Yes | Yes |
| Rohrer et al. 2009 [90] | Yes | Yes | Yes | Yes | Yes | Yes | Yes | Yes | Yes | Yes | Yes |
| Rose et al. 2003 [91] | Yes | Yes | Yes | Yes | Yes | Yes | Yes | Yes | Yes | Yes | Yes |
| Ruiz-Mesa et al. 2017 [92] | Yes | Yes | Yes | Yes | Yes | Yes | Yes | Yes | Yes | Yes | Yes |
| Sabatino et al. 2016 [93] | Yes | Yes | Yes | Yes | Yes | Yes | Yes | Yes | Yes | Yes | Yes |
| Sabatino et al. 2019 [94] | Yes | Yes | Yes | Yes | Yes | Yes | Yes | Yes | Yes | Yes | Yes |
| Salouti et al. 2020 [95] | Yes | Yes | Yes | Yes | Yes | Yes | Yes | Yes | Yes | Yes | Yes |
| Saucedo-Urdapilleta et al. 2019 [96] | Yes | Yes | Yes | Yes | Yes | Yes | Yes | Yes | Yes | Yes | Yes |
| Savini et al. 2009 [97] | Yes | Yes | Yes | Yes | Yes | Yes | Yes | Yes | Yes | Yes | Yes |
| Schultz et al. 2016 [98] | Yes | Yes | Yes | Yes | Yes | Yes | Yes | Yes | Yes | Yes | No |
| Sel et al. 2017 [99] | No | Yes | Yes | Yes | Yes | Yes | Yes | Yes | Yes | Yes | Yes |
| Shajari et al. 2017 [100] | Yes | Yes | Yes | Yes | Yes | Yes | Yes | Yes | Yes | Yes | Yes |
| Shajari et al. 2016 [101] | No | Yes | Yes | Yes | Yes | Yes | Yes | Yes | Yes | Yes | Yes |
| Shammas et al. 2016 [102] | Yes | Yes | Yes | Yes | Yes | Yes | Yes | Yes | Yes | Yes | Yes |
| Shen et al. 2014 [103] | Yes | Yes | Yes | Yes | Yes | Yes | Yes | Yes | Yes | Yes | Yes |
| Shen et al. 2019 [104] | Yes | Yes | Yes | Yes | Yes | Yes | Yes | Yes | Yes | Yes | Yes |
| Shin et al. 2016 [105] | Yes | Yes | Yes | Yes | Yes | Yes | Yes | Yes | Yes | Yes | Yes |
| Shu et al. 2020 [106] | Yes | Yes | Yes | Yes | Yes | Yes | Yes | Yes | Yes | Yes | Yes |
| Song et al. 2020 [107] | Yes | Not clear | Yes | Yes | Yes | Yes | Yes | Yes | Yes | Yes | Yes |
| Srivannaboon et al. 2014 [108] | Yes | Yes | Yes | Yes | Yes | Yes | Yes | Yes | Yes | Yes | Yes |
| Srivannaboon et al. 2015 [109] | Yes | Yes | Yes | Yes | Yes | Yes | Yes | Yes | Yes | Yes | Yes |
| Srivannaboon et al. 2015 [110] | Yes | Yes | Yes | Yes | Yes | Yes | Yes | Yes | Yes | Yes | Yes |
| Su et al. 2008 [111] | Yes | Yes | Yes | Yes | Yes | Yes | Yes | Yes | Yes | Yes | Yes |
| Suto et al. 2015 [112] | Yes | Yes | Yes | Yes | Yes | Yes | Yes | Yes | Yes | Yes | Yes |
| Tu et al. 2020 [113] | Yes | Yes | Yes | Yes | Yes | Yes | Yes | Yes | Yes | Yes | Yes |
| Ueda et al. 2007 [114] | Yes | No | Yes | Yes | Yes | Yes | Yes | Yes | Yes | Yes | Yes |
| Vasavada et al. 2020 [115] | Yes | Yes | Yes | Yes | Yes | Yes | Yes | Yes | Yes | Yes | Yes |
| Ventura et al. 2017 [116] | Yes | Yes | Yes | Yes | Yes | Yes | Yes | Yes | Yes | Yes | Yes |
| Visser et al. 2012 [117] | No | Yes | Yes | Yes | Yes | Yes | Yes | Yes | Yes | Yes | Yes |
| Wang et al. 2017 [118] | Yes | Yes | Yes | Yes | Yes | Yes | Yes | Yes | Yes | Yes | Yes |
| Wang et al. 2019 [119] | Yes | Yes | Yes | Yes | Yes | Yes | Yes | Yes | Yes | Yes | Yes |
| Wang et al. 2008 [120] | Yes | No | Yes | Yes | Yes | Yes | Yes | Yes | Yes | Yes | Yes |
| Wang et al. 2016 [121] | Yes | Yes | Yes | Yes | Yes | Yes | Yes | Yes | Yes | Yes | Yes |
| Whang et al. 2018 [122] | Yes | Yes | Yes | Yes | Yes | Yes | Yes | Yes | Yes | Yes | Yes |
| Wissa et al. 2012 [123] | Yes | Yes | Yes | Yes | Yes | Yes | Yes | Yes | Yes | Yes | Yes |
| Yang et al. 2017 [124] | Yes | No | Yes | Yes | Yes | Yes | Yes | Yes | Yes | Yes | Yes |
| Yeu et al. 2019 [125] | Yes | Yes | Yes | Yes | Yes | Yes | Yes | Yes | Yes | Yes | Yes |
| Yu et al. 2017 [126] | Yes | Yes | Yes | Yes | Yes | Yes | Yes | Yes | Yes | Yes | Yes |
| Zhang et al. 2016 [127] | Yes | Yes | Yes | Yes | Yes | Yes | Yes | Yes | Yes | Yes | Yes |
| Zhang et al. 2015 [128] | Yes | Yes | Yes | Yes | Yes | Yes | Yes | Yes | Yes | Yes | Yes |
| Zhao et al. 2013 [129] | Yes | Yes | Yes | Yes | Yes | Yes | Yes | Yes | Yes | Yes | Yes |

**Appendix I Table 1 References**

1. Akman A, Asena L, Güngör SG. Evaluation and comparison of the new swept source OCT-based IOLMaster 700 with the IOLMaster 500. Br J Ophthalmol. 2016;100(9):1201-5.

2. Aktas S, Aktas H, Tetikoglu M, Sagdk HM, Özcura F. Refractive results using a new optical biometry device: comparison with ultrasound biometry data. Medicine (Baltimore). 2015;94(48):e2169.

3. Alvani A, Pakravan M, Esfandiari H, Yaseri M, Yazdani S, Ghahari E. Biometric changes after trabeculectomy with contact and non-contact biometry. Optom Vis Sci. 2016;93(2):136-40.

4. An Y, Kang EK, Kim H, Kang MJ, Byun YS, Joo CK. Accuracy of swept-source optical coherence tomography based biometry for intraocular lens power calculation: a retrospective cross-sectional study. BMC Ophthalmol. 2019;19(1):30.

5. Arriola-Villalobos P, Almendral-Gómez J, Garzón N, et al. Agreement and clinical comparison between a new swept-source optical coherence tomography-based optical biometer and an optical low-coherence reflectometry biometer. Eye (Lond). 2017;31(3):437-42.

6. Asena L, Akman A, Güngör SG, Dursun Altınörs D. Comparison of keratometry obtained by a swept source OCT-based biometer with a standard optical biometer and Scheimpflug imaging. Curr Eye Res. 2018;43(7):882-8.

7. Attas-Fox L, Zadok D, Gerber Y, Morad Y, Eting E, Benamou N, et al. Axial length measurement in eyes with diabetic macular edema: a-scan ultrasound versus IOLMaster. Ophthalmology. 2007;114(8):1499-504.

8. Aydin R, Karaman Erdur S, Serefoglu Cabuk K, Karahan E, Kaynak S. Comparision of optical low coherence reflectometry versus ultrasonic biometry in high hypermetropia. Eye Contact Lens. 2018;44 Suppl 1:S115-7.

9. Bai QH, Wang JL, Wang QQ, Yan QC, Zhang JS. The measurement of anterior chamber depth andaxial length with the IOLMaster compared withContact ultrasonic axial scan. Int J Ophthalmol. 2007;1(4):151-4.

10. Bi WJ, Wu J, Guo Z, Liu K, Wang Y. Five methods for measuring central corneal thickness in myopic patients. International Eye Science. 2020;20(7):1286-9.

11. Buckhurst PJ, Wolffsohn JS, Shah S, Naroo SA, Davies LN, Berrow EJ. A new optical low coherence reflectometry device for ocular biometry in Cataract patients. Br J Ophthalmol. 2009;93(7):949-53.

12. Bullimore MA, Slade S, Yoo P, Otani T. An evaluation of the IOLMaster 700. Eye Contact Lens. 2019;45(2):117-23.

13. Çağlar Ç, Kocamış Sİ, Demir E, Durmuş M. Comparison of the measurements of a novel optical biometry: Nidek AL-Scan with Sirius and a ultrasound biometry. Int Ophthalmol. 2017;37(3):491-8.

14. Calvo-Sanz JA, Portero-Benito A, Arias-Puente A. Efficiency and measurements agreement between swept-source OCT and low-coherence interferometry biometry systems. Graefes Arch Clin Exp Ophthalmol. 2018;256(3):559-66.

15. Can E, Eser-Ozturk H, Duran M, Cetinkaya T, Ariturk N. Comparison of central corneal thickness measurements using different imaging devices and ultrasound pachymetry. Indian J Ophthalmol. 2019;67(4):496-99.

16. Can E, Duran M, Çetinkaya T, Arıtürk N. Comparison of anterior segment measurements with optical low-coherence reflectometry and partial-coherence interferometry optical biometers. Middle East Afr J Ophthalmol. 2016;23(4):288-92.

17. Carkeet A, Saw SM, Gazzard G, Tang W, Tan DT. Repeatability of IOLMaster biometry in children. Optom Vis Sci. 2004;81(11):829-34.

18. Cech R, Utíkal T, Juhaszova J. [Comparison of optical and ultrasound biometry and assessment of using both methods in practice]. Cesk Slov Oftalmol. 2014;70(1):3-9.

19. Chan TCY, Wan KH, Tang FY, Wang YM, Yu M, Cheung C. Repeatability and agreement of a swept-source optical coherence tomography-based biometer IOLMaster 700 versus a Scheimpflug imaging-based biometer AL-Scan in cataract patients. Eye Contact Lens. 2020;46(1):35-45.

20. Chen YA, Hirnschall N, Findl O. Evaluation of 2 new optical biometry devices and comparison with the current gold standard biometer. J Cataract Refract Surg. 2011 37(3):513-7.

21. Cheng H, Li J, Cheng B, Wu M. Refractive predictability using two optical biometers and refraction types for intraocular lens power calculation in cataract surgery. Int Ophthalmol. 2020;40(7):1849-56.

22. Cho YJ, Lim TH, Choi KY, Cho BJ. Comparison of ocular biometry using new swept-source optical coherence tomography-based optical biometer with other devices. Korean J Ophthalmol. 2018;32(4):257-64.

23. Cınar Y, Cingü AK, Sahin M, Sahin A, Yüksel H, Türkcü FM, et al. Comparison of optical versus ultrasonic biometry in keratoconic eyes. J Ophthalmol. 2013;2013:481238.

24. Cruysberg LP, Doors M, Verbakel F, Berendschot TT, De Brabander J, Nuijts RM. Evaluation of the Lenstar LS 900 non-Contact biometer. Br J Ophthalmol. 2010;94(1):106-10.

25. Cvetkovic A, Sreckovic S, Petrovic M. Comparison of biometric values and intraocular lens power calculations obtained by ultrasound and optical biometry. Serbian Journal of Experimental and Clinical Research. 2016;17(4):321-26.

26. Doğan M, Polat O, Karadaş M, Küsbeci T, Yavaş GF, İnanet S, et al. Comparison of partial coherence interferometry and optic low coherence reflectometry for intraocular lens power calculation in cataract patients. Turk Oftalmoloiji Dergisi. 2014;44(6):419-23.

27. Du YL, Wang G, Huang HC, Lin LY, Jin C, Liu LF, et al. Comparison of OA-2000 and IOL Master 500 using in cataract patients with high myopia. Int J Ophthalmol. 2019;12(5):844-47.

28. Einan-Lifshitz A, Rozenberg A, Wang L, Koch DD, Shoshany N, Zadok D, et al. Accuracy and feasibility of axial length measurements by a new optical low-coherence reflectometry-based device in eyes with posterior subcapsular cataract. J Cataract Refract Surg. 2017;43(7):898-901.

29. El-Baha SM, Hemeida TS. Comparison of refractive outcome using intraoperative biometry and partial coherence interferometry in silicone oil-filled eyes. Retina. 2009;29(1):64-8.

30. Elbaz U, Barkana Y, Gerber Y, Avni I, Zadok D. Comparison of different techniques of anterior chamber depth and keratometric measurements. Am J Ophthalmol. 2007;143(1):48-53.

31. El Chehab H, Agard E, Dot C. Comparison of two biometers: a swept-source optical coherence tomography and an optical low-coherence reflectometry biometer. Eur J Ophthalmol. 2019;29(5):547-54.

32. Eleftheriadis H. IOLMaster biometry: refractive results of 100 consecutive cases. Br J Ophthalmol. 2003;87(8):960-63.

33. Epitropoulos A. Axial length measurement acquisition rates of two optical biometers in cataractous eyes. Clin Ophthalmol. 2014;8:1369-76.

34. Findl O, Kriechbaum K, Sacu S, Kiss B, Polak K, Nepp J, et al. Influence of operator experience on the performance of ultrasound biometry compared to optical biometry before cataract surgery. J Cataract Refract Surg. 2003;29(10):1950-5.

35. Fisus AD, Hirnschall ND, Findl O. Comparison of two swept-source optical coherence tomography-based biometry devices. J Cataract Refract Surg. 2020; 10.1097/j.jcrs.0000000000000373.

36. Fontes BM, Fontes BM, Castro E. Intraocular lens power calculation by measuring axial length with partial optical coherence and ultrasonic biometry. Arq Bras Oftalmol. 2011;74(3):166-70.

37. Gao R, Chen H, Savini G, Miao Y, Wang X, Yang J, et al. Comparison of ocular biometric measurements between a new swept-source optical coherence tomography and a common optical low coherence reflectometry. Sci Rep. 2017;7(1):2484.

38. Ghaffari R, Mahmoudzadeh R, Mohammadi SS, Salabati M, Latifi G, Ghassemi H. Assessing the validity of measurements of swept-source and partial coherence interferometry devices in cataract patients. Optom Vis Sci. 2019;96(10):745-50.39. Goebels S, Pattmöller M, Eppig T, Cayless A, Seitz B, Langenbucher A. Comparison of 3 biometry devices in cataract patients. J Cataract Refract Surg. 2015;41(11):2387-93.

40. Goebels SC, Seitz B, Langenbucher A. Comparison of the new biometer OA-1000 with IOLMaster and Tomey AL-3000. Curr Eye Res. 2013;38(9):910-6.

41. Goto S, Maeda N, Noda T, Ohnuma K, Iehisa I, Koh S, et al. Change in optical axial length after cataract surgery: segmental method vs composite method. J Cataract Refract Surg. 2020;46(5):710-5.

42. Goyal R, North RV, Morgan JE. Comparison of laser interferometry and ultrasound A-scan in the measurement of axial length. Acta Ophthalmol Scand. 2003;81(4):331-5.

43. Güler E, Kulak AE, Totan Y, Yuvarlak A, Hepşen İF. Comparison of a new optical biometry with an optical low-coherence reflectometry for ocular biometry. Contact Lens Anterior Eye, 2016;39(5):336-41.

44. Guo XX, You R, Li SS, Yang XF, Zhao L, Zhang F, et al. Comparison of ocular parameters of two biometric measurement devices in highly myopic eyes. Int J Ophthalmol. 2019;12(10):1548-54.

45. Gursoy H, Sahin A, Basmak H, Ozer A, Yildirim N, Colak E. Lenstar versus ultrasound for ocular biometry in a pediatric population. Optom Vis Sci. 2011;88(8):912-9.

46. Haddad JS, Barnwell E, Rocha KM, Ambrosio R Jr, Waring Iv GO. Comparison of biometry measurements using standard partial coherence interferometry versus new Scheimpflug tomography with integrated axial length capability. Clin Ophthalmol. 2020;14:353-8.

47. Hashemi H, Yazdani K, Mehravaran S, Fotouhi A. Anterior chamber depth measurement with A-scan ultrasonography, Orbscan II, and IOLMaster. Optom Vis Sci. 2005;82(10):900-4.

48. Henriquez MA, Zúñiga R, Camino M, Camargo J, Ruiz-Montenegro K, Izquierdo L Jr. Effectiveness and agreement of 3 optical biometers in measuring axial length in the eyes of patients with mature cataracts. J Cataract Refract Surg. 2020;46(9):1222-8.

49. Hoffer KJ, Hoffmann PC, Savini G. Comparison of a new optical biometer using swept-source optical coherence tomography and a biometer using optical low-coherence reflectometry. J Cataract Refract Surg. 2016;42(8):1165-72.

50. Hoffer KJ, Savini G. Comparison of AL-Scan and IOLMaster 500 partial coherence interferometry optical biometers. J Refract Surg. 2016;32(10):694-8.

51. Hoffer KJ, Shammas HJ, Savini G. Comparison of 2 laser instruments for measuring axial length. J Cataract Refract Surg. 2010;36(4):644-8.

52. Hoffer KJ, Shammas HJ, Savini G, Huang J. Multicenter study of optical low-coherence interferometry and partial-coherence interferometry optical biometers with patients from the United States and China. J Cataract Refract Surg. 2016;42(1):62-7.

53. Holzer MP, Mamusa M, Auffarth GU. Accuracy of a new partial coherence interferometry analyser for biometric measurements. Br J Ophthalmol. 2009;93(6):807-10.

54. Hua Y, Qiu W, Xiao Q, Wu Q. Precision (repeatability and reproducibility) of ocular parameters obtained by the Tomey OA-2000 biometer compared to the IOLMaster in Healthy eyes. PLoS One. 2018;13(2):e0193023.

55. Huang J, McAlinden C, Su B, Pesudovs K, Feng Y, Hua Y, et al. The effect of cycloplegia on the lenstar and the IOLMaster biometry. Optom Vis Sci. 2012;89(12):1691-6.

56. Huang J, Chen H, Li Y, Chen Z, Gao R, Yu J, et al. Comprehensive comparison of axial length measurement with three swept-source OCT-based biometers and partial coherence interferometry. J Refract Surg. 2019;35(2):115-20.

57. Huang J, Savini G, Hoffer KJ, Chen H, Lu W, Hu Q, et al. Repeatability and interobserver reproducibility of a new optical biometer based on swept-source optical coherence tomography and comparison with IOLMaster. Br J Ophthalmol. 2017;101(4):493-8.

58. Hui S, Yi L. Comparison of two optical biometers in intraocular lens power calculation. Indian J Ophthalmol. 2014;62(9):931-4.

59. Hussaindeen JR, Mariam EG, Arunachalam S, Bhavatharini R, Gopalakrishnan A, Narayanan A, et al. Comparison of axial length using a new swept-source optical coherence tomography-based biometer-ARGOS with partial coherence interferometry-based biometer-IOLMaster among school children. PLoS One. 2018;13(12):e0209356.

60. Jung S, Chin HS, Kim NR, Lee KW, Jung JW. Comparison of repeatability and agreement between swept-source optical biometry and dual-Scheimpflug topography. J Ophthalmol. 2017;2017:1516395.

61. Kaswin G, Rousseau A, Mgarrech M, Barreau E, Labetoulle M. Biometry and intraocular lens power calculation results with a new optical biometry device: comparison with the gold standard. J Cataract Refract Surg. 2014;40(4):593-600.

62. Kaya F, Koçak I, Aydin A, Baybora H, Karadayi K. Comparison of intraocular lens power calculation using a standard ultrasonic biometer and a new optical biometer. International Eye Science. 2016;16(5):807-10.

63. Sharma A, Sharma AV. Comparison of accuracy between ultrasound B scan and partial coherence interferometry (IOL master) in IOL (intraocular lens) power calculation. Indian J Clin Exp Ophthalmol. 2018;4(4):492-8.

64. Kongsap P. Comparison of a new optical biometer and a standard biometer in cataract patients. Eye Vis (Lond). 2016;3:27.

65. Kunert KS, Peter M, Blum M, Haigis W, Sekundo W, Schütze J, et al. Repeatability and agreement in optical biometry of a new swept-source optical coherence tomography-based biometer versus partial coherence interferometry and optical low-coherence reflectometry. J Cataract Refract Surg. 2016;42(1):76-83.

66. Kurian M, Negalur N, Das S, Puttaiah NK, Haria D, J TS, et al. Biometry with a new swept-source optical coherence tomography biometer: Repeatability and agreement with an optical low-coherence reflectometry device. J Cataract Refract Surg. 2016;42(4):577-81.

67. Lam AKC, Chan R, Pang PC. The repeatability and accuracy of axial length and anterior chamber depth measurements from the IOLMaster. Ophthalmic Physiol Opt. 2001;21(6):477-83.

68. Li Y, Li HX, Liu YC, Guo YT, Gao JM, Wu B, et al. Comparison of immersion ultrasound and low coherence reflectometry for ocular biometry in cataract patients. Int J Ophthalmol. 2018;11(6):966-9.

69. Li J, Chen H, Savini G, Lu W, Yu X, Bao F, et al. Measurement agreement between a new biometer based on partial coherence interferometry and a validated biometer based on optical low-coherence reflectometry. J Cataract Refract Surg. 2016;42(1):68-75.

70. Li XT, Chen BC, Li HE, et al. The application of scanning optical coherence biometry in high myopia Cataract. Chinese Journal of Experimental Ophthalmology. 2020(02):128-33.

71. Liampa Z, Kynigopoulos M, Pallas G, Gerding H. Comparison of two partial coherence interferometry devices for ocular biometry. Klin Monbl Augenheilkd. 2010;227(4):285-8.

72. Liao X, Peng Y, Liu B, Tan QQ, Lan CJ. Agreement of ocular biometric measurements in young healthy eyes between IOLMaster 700 and OA-2000. Sci Rep. 2020;10(1):3134.

73. Mandal P, Berrow EJ, Naroo SA, Wolffsohn JS, Uthoff D, Holland D, et al. Validity and repeatability of the Aladdin ocular biometer. Br J Ophthalmol. 2014;98(2):256-8.

74. McAlinden C, Gao R, Yu A, Wang X, Yang J, Yu Y, et al. Repeatability and agreement of ocular biometry measurements: Aladdin versus Lenstar. Br J Ophthalmol. 2017;101(9):1223-9.

75. McAlinden C, Wang Q, Gao R, Zhao W, Yu A, Li Y, et al. Axial length measurement failure rates with biometers using swept-source optical coherence tomography compared to partial-coherence interferometry and optical low-coherence interferometry. Am J Ophthalmol. 2017;173:64-9.

76. Moon SW, Lim SH, Lee HY. Accuracy of biometry for intraocular lens implantation using the new partial coherence interferometer, AL-scan. Korean J Ophthalmol. 2014;28(6):444-50.

77. Mueller A, Thomas BC, Auffarth GU, Holzer MP. Comparison of a new image-guided system versus partial coherence interferometry, Scheimpflug imaging, and optical low-coherence reflectometry devices: keratometry and repeatability. J Cataract Refract Surg. 2016;42(5):672-8.

78. Muzyka-Woźniak M, Oleszko A. Comparison of anterior segment parameters and axial length measurements performed on a Scheimpflug device with biometry function and a reference optical biometer. Int Ophthalmol. 2019;39(5):1115-22.

79. Németh J, Fekete O, Pesztenlehrer N. Optical and ultrasound measurement of axial length and anterior chamber depth for intraocular lens power calculation. J Cataract Refract Surg. 2003;29(1):85-8.

80. Olsen T. Improved accuracy of intraocular lens power calculation with the Zeiss IOLMaster. Acta Ophthalmol Scand. 2007;85(1):84-7.

81. Omoto MK, Torii H, Masui S, Ayaki M, Tsubota K, Negishi K. Ocular biometry and refractive outcomes using two swept-source optical coherence tomography-based biometers with segmental or equivalent refractive indices. Sci Rep. 2019;9(1):6557.

82. Tañá-Rivero P, Aguilar-Córcoles S, [Rodríguez](https://pubmed.ncbi.nlm.nih.gov/?term=Rodr%C3%ADguez-Prats+JL&cauthor_id=32860152)-Prats JL, [Montés-Micó](https://pubmed.ncbi.nlm.nih.gov/?term=Mont%C3%A9s-Mic%C3%B3+R&cauthor_id=32860152) R, Ruiz-Mesa R. Agreement of white-to-white measurements with swept-source OCT, Scheimpflug and color LED devices. Int Ophthalmol. 2021;41(1):57-65.

83. Rabsilber TM, Jepsen C, Auffarth GU, Holzer MP. Intraocular lens power calculation: clinical comparison of 2 optical biometry devices. J Cataract Refract Surg. 2010;36(2):230-4.

84. Rajan MS, Keilhorn I, Bell JA. Partial coherence laser interferometry vs conventional ultrasound biometry in intraocular lens power calculations. Eye (Lond). 2002;16(5):552-6.

85. Raymond S, Favilla I, Santamaria L. Comparing ultrasound biometry with partial coherence interferometry for intraocular lens power calculations: a randomized study. Invest Ophthalmol Vis Sci. 2009;50(6):2547-52.

86. Passi SF, Thompson AC, Gupta PK. Comparison of agreement and efficiency of a swept source-optical coherence tomography device and an optical low-coherence reflectometry device for biometry measurements during Cataract evaluation. Clin Ophthalmol. 2018;12:2245-51.

87. Pereira JMM, Neves A, Alfaiate P, Santos M, Aragão H, Sousa JC. Lenstar^®^ LS 900 vs Pentacam^®^-AXL: comparative study of ocular biometric measurements and intraocular lens power calculation. Eur J Ophthalmol. 2018;28(6):645-51.

88. Reddy AR, Pande MV, Finn P, El-Gogary H. Comparative estimation of anterior chamber depth by ultrasonography, Orbscan II, and IOLMaster. J Cataract Refract Surg. 2004;30(6):1268-71.

89. Reitblat O, Levy A, Kleinmann G, Assia EI. Accuracy of intraocular lens power calculation using three optical biometry measurement devices: the OA-2000, Lenstar-LS900 and IOLMaster-500. Eye (Lond). 2018;32(7):1244-52.

90. Rohrer K, Frueh BE, Wälti R, Clemetson IA, Tappeiner C, Goldblum D. Comparison and evaluation of ocular biometry using a new noncontact optical low-coherence reflectometer. Ophthalmology. 2009;116(11):2087-92.

91. Rose LT, Moshegov CN. Comparison of the Zeiss IOLMaster and applanation A-scan ultrasound: biometry for intraocular lens calculation. Clin Exp Ophthalmol. 2003;31(2):121-4.

92. Ruiz-Mesa R, Abengozar-Vela A, Ruiz-Santos M. Comparison of a new Scheimpflug imaging combined with partial coherence interferometry biometer and a low-coherence reflectometry biometer. J Cataract Refract Surg. 2017;43(11):1406-12.

93. Sabatino F, Findl O, Maurino V. Comparative analysis of optical biometers. J Cataract Refract Surg. 2016;42(5):685-93.

94. Sabatino F, Matarazzo F, Findl O, Maurino V. Comparative analysis of 2 swept-source optical coherence tomography biometers. J Cataract Refract Surg. 2019;45(8):1124-9.

95. Salouti R, Kamalipour A, Masihpour N, Zamani M, Ghoreyshi M, Salouti K, et al. Effect of photorefractive keratectomy on agreement of anterior segment variables obtained by a swept-source biometer vs a Scheimpflug-based tomographer. J Cataract Refract Surg. 2020;46(9):1229-35.

96. Saucedo-Urdapilleta R, González-Godínez S, Mayorquín-Ruiz M, Moragrega-Adame E, Velasco-Barona C, Gonzalez-Salinas R. Comparative analysis and repeatability assessment of IOL Master 500 versus IOL Master 700 biometry in cataract patients. Revista Mexicana de Oftalmología. 2019;93(3):107-12.

97. Savini G, Barboni P, Carbonelli M, Hoffer KJ. Accuracy of Scheimpflug corneal power measurements for intraocular lens power calculation. J Cataract Refract Surg. 2009;35(7):1193-7.

98. Schultz M, Oberheide U, Kermani O. Comparability of an image-guided system with other instruments in measuring corneal keratometry and astigmatism. J Cataract Refract Surg. 2016;42(6):904-12.

99. Sel S, Stange J, Kaiser D, Kiraly L. Repeatability and agreement of Scheimpflug-based and swept-source optical biometry measurements. Cont Lens Anterior Eye. 2017;40(5):318-22.

100. Shajari M, Cremonese C, Petermann K, Singh P, Müller M, Kohnen T.. Comparison of axial length, corneal curvature, and anterior chamber depth measurements of 2 recently introduced devices to a known biometer. Am J Ophthalmol. 2017;178:58-64.

101. Shajari M, Lehmann UC, Kohnen T. Comparison of corneal diameter and anterior chamber depth measurements using 4 different devices. Cornea. 2016;35(6):838-42.

102. Shammas HJ, Ortiz S, Shammas MC, Kim SH, Chong C. Biometry measurements using a new large-coherence-length swept-source optical coherence tomographer. J Cataract Refract Surg. 2016;42(1):50-61.

103. Shen PY, Ding XH, Zhong XW, Chen HB, Xing JQ, He MG. Comparison of two optical biometers in Chinese school-aged children. Int Eye Sci. 2014;14(11):1921-6.

104. Shen L, Li DJ, Wang ZY, Chen W, Zhao Q, Li YF, et al. Agreement of biometry parameters measured by IOLMaster 700 with IOLMaster 500 in Cataract eyes with high myopia. Chin J Exp Ophthalmol. 2019(04):292-6.

105. Shin MC, Chung SY, Hwang HS, Han KE. Comparison of two optical biometers. Optom Vis Sci. 2016;93(3):259-65.

106. Shu B, Bao F, Savini G, Lu W, Tu R, Chen H, et al. Effect of orthokeratology on precision and agreement assessment of a new swept-source optical coherence tomography biometer. Eye Vis (Lond). 2020;7:13.

107. Shin SJ, Yeh YD, Young HJ, Sun JH. Comparison of ocular biometry and refractive outcomes using IOL Master 500, IOL Master 700, and Lenstar LS900. Korean J Ophthalmol. 2020;34(2):126-32.

108. Srivannaboon S, Chirapapaisan C, Chonpimai P, Koodkaew S. Comparison of ocular biometry and intraocular lens power using a new biometer and a standard biometer. J Cataract Refract Surg. 2014;40(5):709-15.

109. Srivannaboon S, Chirapapaisan C, Chonpimai P, Koodkaew S. Comparison of corneal astigmatism measurements of 2 optical biometer models for toric intraocular lens selection. J Cataract Refract Surg. 2015;41(2):364-71.

110. Srivannaboon S, Chirapapaisan C, Chonpimai P, Loket S. Clinical comparison of a new swept-source optical coherence tomography-based optical biometer and a time-domain optical coherence tomography-based optical biometer. J Cataract Refract Surg. 2015;41(10):2224-32.

111. Su PF, Lo AY, Hu CY, Chang SW. Anterior chamber depth measurement in phakic and pseudophakic eyes. Optom Vis Sci. 2008;85(12):1193-200.

112. Suto C, Shimamura E, Watanabe I. Comparison of 2 optical biometers and evaluation of the Camellin-Calossi intraocular lens formula for normal Cataractous eyes. J Cataract Refract Surg. 2015;41(11):2366-72.

113. Tu R, Yu J, Savini G, Ye J, Ning R, Xiong J, et al. Agreement between two optical biometers based on large coherence length SS-OCT and Scheimpflug imaging/partial coherence interferometry. J Refract Surg. 2020;36(7):459-65.

114. Ueda T, Taketani F, Ota T, Hara Y. Impact of nuclear cataract density on postoperative refractive outcome: IOL Master versus ultrasound. Ophthalmologica. 2007;221(6):384-7.

115. Vasavada SA, Patel P, Vaishnav VR, Ashena Z, Srivastava S, Vasavada V, et al. Comparison of optical low-coherence reflectometry and swept-source OCT-based biometry devices in dense cataracts. J Refract Surg. 2020;36(8):557-64.

116. Ventura BV, Ventura MC, Wang L, Koch DD, Weikert MP. Comparison of biometry and intraocular lens power calculation performed by a new optical biometry device and a reference biometer. J Cataract Refract Surg. 2017;43(1):74-9.

117. Visser N, Berendschot TT, Verbakel F, de Brabander J, Nuijts RM. Comparability and repeatability of corneal astigmatism measurements using different measurement technologies. J Cataract Refract Surg. 2012;38(10):1764-70.

118. Wang CC, Wu GF, Yu XQ. A comparative study on the accuracy of axial length and anterior chamber depth in Cataract patients with A-scan and IOLMaster. International Eye Science. 2017;17(09):1697-9.

119. Wang ZY, Yang WL, Li DJ, Chen W, Zhao Q, Li YF, et al. Comparison of biometry with the Pentacam AXL, IOLMaster 700 and IOLMaster 500 in cataract patients. Zhonghua Yan Ke Za Zhi. 2019(07):515-21.

120. Wang JK, Hu CY, Chang SW. Intraocular lens power calculation using the IOLMaster and various formulas in eyes with long axial length. J Cataract Refract Surg. 2008;34(2):262-7.

121. Wang XG, Dong J, Pu YL, Liu HJ, Wu Q. Comparison axial length measurements from three biometric instruments in high myopia. Int J Ophthalmol. 2016;9(6):876-80.

122. Whang WJ, Yoo YS, Kang MJ, Joo CK. Predictive accuracy of partial coherence interferometry and swept-source optical coherence tomography for intraocular lens power calculation. Sci Rep. 2018;8(1):13732.

123. Wissa AR, Wahba SS, Roshdy MM. Agreement and relationship between ultrasonic and partial coherence interferometry measurements of axial length and anterior chamber depth. Clin Ophthalmol. 2012;6:193-8.

124. Yang JY, Kim HK, Kim SS. Axial length measurements: comparison of a new swept-source optical coherence tomography-based biometer and partial coherence interferometry in myopia. J Cataract Refract Surg. 2017;43(3):328-32.

125. Yeu E. Agreement of ocular biometry measurements between 2 biometers. J Cataract Refract Surg. 2019;45(8):1130-4.

126. Yu SS, Song H, Tang X. Repeatability of Ophtha Top topography and comparison with IOL-Master and LenstarLS900 in cataract patients. Int J Ophthalmol. 2017;10(11):1703-9.

127. Zhang J, Lian JC, Zhang SS, Yu Q, Zhou JB. Comparison of the biometric measurements obtained using the Lenstar, Pentacam and ultrasound pachymetry methods in cataract patients. International Eye Science. 2016;16(4):594-9.

128. Zhang J, Zhang SS, Yu Q, Wu JX, Lian JC. Comparison of anterior chamber depths measured using the Pentacam, the IOLMaster, and ultrasound pachymetry. International Eye Science. 2015;15(8):1313-8.

129. Zhao J, Chen Z, Zhou Z, Ding L, Zhou X. Evaluation of the repeatability of the Lenstar and comparison with two other non-contact biometric devices in myopes. Clin Exp Optom. 2013;96(1):92-9.

Appendix I Table 3. Results of direct-comparison meta-analysis in axial length (mm)

| Devices | Number of studies | WMD (95% CI) | I^2^ |
| --- | --- | --- | --- |
| IOLMaster 500 vs. IOLMaster 700 | 12 | 0.022 (−0.099, 0.144) | 29.30% |
| Contact ultrasound vs. AL-Scan | 3 | −0.138 (−0.307, 0.03) | 4.90% |
| Contact ultrasound vs. Lenstar | 7 | −0.26 (−0.552, 0.031) | 83.50% |
| Lenstar vs. IOLMaster 700 | 7 | −0.008 (−0.167, 0.152) | 0.00% |
| Contact ultrasound vs. IOLMaster | 28 | −0.159 (−0.237, −0.082) | 29.40% |
| IOLMaster vs. Lenstar | 14 | −0.023 (−0.123, 0.076) | 0.00% |
| Aladdin vs. IOLMaster 700 | 1 | 0 (−0.748, 0.748) | 0.00% |
| AL-Scan vs. Lenstar | 5 | 0 (−0.158, 0.158) | 0.00% |
| IOLMaster vs. IOLMaster 500 | 1 | 0.02 (−0.411, 0.451) | 0.00% |
| IOLMaster 500 vs. Lenstar | 8 | −0.066 (−0.161, 0.029) | 0.00% |
| Contact ultrasound vs. IOLMaster 500 | 2 | −0.137 (−0.275, 0.001) | 0.00% |
| Contact ultrasound vs. IOLMaster 700 | 1 | −0.07 (−0.465, 0.325) | 0.00% |
| IOLMaster 500 vs. OA-2000 | 9 | −0.015 (−0.124, 0.095) | 0.00% |
| Immersion ultrasound vs. OA-2000 | 1 | 0.1 (−0.412, 0.612) | 0.00% |
| Immersion ultrasound vs. IOLMaster | 2 | −0.01 (−0.333, 0.313) | 0.00% |
| Lenstar vs. OA-2000 | 5 | 0.036 (−0.09, 0.162) | 0.00% |
| IOLMaster vs. OA-2000 | 4 | 0.01 (−0.1, 0.12) | 0.00% |
| Contact ultrasound vs. OA-1000 | 1 | −0.18 (−0.439, 0.079) | 0.00% |
| IOLMaster vs. OA-1000 | 1 | 0.24 (−0.024, 0.504) | 0.00% |
| IOLMaster 500 vs. AL-Scan | 4 | 0.007 (−0.113, 0.127) | 0.00% |
| IOLMaster 500 vs. Aladdin | 3 | −0.007 (−0.222, 0.208) | 0.00% |
| IOLMaster 700 vs. OA-2000 | 2 | 0.012 (−0.155, 0.179) | 0.00% |
| IOLMaster 500 vs. Argos | 4 | 0 (−0.141, 0.14) | 0.00% |
| IOLMaster vs. Argos | 2 | −0.104 (−0.245, 0.037) | 0.00% |
| Galilei G6 vs. IOLMaster 700 | 2 | 0.034 (−0.363, 0.431) | 0.00% |
| Contact ultrasound vs. Aladdin | 1 | −0.2 (−0.519, 0.119) | 0.00% |
| Immersion ultrasound vs. Lenstar | 1 | 0.08 (−0.393, 0.553) | 0.00% |
| Lenstar vs. Aladdin | 2 | 0.001 (−0.254, 0.257) | 0.00% |
| IOLMaster vs. Aladdin | 2 | −0.02 (−0.132, 0.093) | 0.00% |
| Aladdin vs. OA-2000 | 1 | 0.02 (−0.103, 0.143) | 0.00% |
| IOLMaster 500 vs. Pentacam AXL | 3 | 0.008 (−0.165, 0.18) | 0.00% |
| IOLMaster 700 vs. Argos | 2 | 0.022 (−0.196, 0.24) | 0.00% |
| Lenstar vs. Pentacam AXL | 3 | 0.032 (−0.103, 0.168) | 0.00% |
| IOLMaster 700 vs. Pentacam AXL | 4 | −0.005 (−0.147, 0.137) | 4.00% |
| Lenstar vs. Argos | 1 | 0.05 (−0.205, 0.305) | 0.00% |
| Galilei G6 vs. Lenstar | 1 | −0.04 (−0.274, 0.194) | 0.00% |
| Galilei G6 vs. IOLMaster 500 | 1 | 0.02 (−0.308, 0.348) | 0.00% |
| IOLMaster vs. IOLMaster 700 | 1 | −0.06 (−0.621, 0.501) | 0.00% |
| Contact ultrasound vs. Argos | 1 | −0.09 (−0.63, 0.45) | 0.00% |
| AL-Scan vs. IOLMaster 700 | 1 | −0.01 (−0.633, 0.613) | 0.00% |
| Argos vs. Pentacam AXL | 1 | 0.02 (−0.31, 0.35) | 0.00% |
| IOLMaster 700 vs. ANTERION | 1 | 0.01 (−0.156, 0.176) | 0.00% |
| OA-2000 vs. Argos | 1 | 0.03 (−0.234, 0.294) | 0.00% |
| Galilei G6 vs. Pentacam AXL | 1 | −0.37 (−0.881, 0.141) | 0.00% |

*WMD* = weighted mean differences; *CI* = confidence interval Appendix I Table 4. Results of direct-comparison meta-analysis in keratometry in the flattest meridian (D)

| Devices | Number of studies | WMD (95% CI) | I^2^ |
| --- | --- | --- | --- |
| IOLMaster 500 vs. IOLMaster 700 | 5 | 0.043 (−0.174, 0.26) | 0.00% |
| IOLMaster vs. Lenstar | 9 | 0.047 (−0.082, 0.175) | 0.00% |
| AL-Scan vs. Lenstar | 3 | 0.173 (−0.26, 0.605) | 0.00% |
| Lenstar vs. OA-2000 | 4 | −0.131 (−0.33, 0.068) | 32.20% |
| IOLMaster vs. OA-2000 | 2 | 0.354 (−0.901, 1.608) | 96.30% |
| Lenstar vs. IOLMaster 700 | 2 | −0.025 (−0.293, 0.243) | 0.00% |
| IOLMaster 500 vs. Aladdin | 2 | 0.093 (−0.323, 0.509) | 0.00% |
| IOLMaster 500 vs. Lenstar | 4 | 0.037 (−0.107, 0.181) | 0.00% |
| IOLMaster 500 vs. OA-2000 | 3 | 0.048 (−0.142, 0.239) | 0.00% |
| Galilei G6 vs. IOLMaster 700 | 2 | −0.038 (−0.536, 0.461) | 0.00% |
| Lenstar vs. Aladdin | 2 | 0.025 (−0.276, 0.326) | 0.00% |
| IOLMaster 500 vs. Pentacam AXL | 2 | 0.201 (−0.075, 0.477) | 0.00% |
| Lenstar vs. Pentacam AXL | 1 | 0.13 (−0.181, 0.441) | 0.00% |
| IOLMaster 500 vs. AL-Scan | 1 | −0.06 (−0.345, 0.225) | 0.00% |
| Galilei G6 vs. IOLMaster 500 | 1 | −0.03 (−0.515, 0.455) | 0.00% |
| Galilei G6 vs. Pentacam AXL | 1 | 0.33 (−0.664, 1.324) | 0.00% |
| IOLMaster 700 vs. Pentacam AXL | 1 | 0.08 (−0.859, 1.019) | 0.00% |
| IOLMaster 700 vs. OA-2000 | 1 | 0.05 (−0.343, 0.443) | 0.00% |

*WMD* = weighted mean differences; *CI* = confidence interval

Appendix I Table 5. Results of direct-comparison meta-analysis in keratometry in the steepest meridian (D)

| Devices | Number of studies | WMD (95% CI) | I^2^ |
| --- | --- | --- | --- |
| IOLMaster 500 vs. IOLMaster 700 | 5 | 0.082 (−0.15, 0.314) | 0.00% |
| IOLMaster vs. Lenstar | 9 | 0.06 (−0.067, 0.186) | 0.00% |
| AL-Scan vs. Lenstar | 3 | 0.113 (−0.388, 0.614) | 0.00% |
| Lenstar vs. OA-2000 | 4 | 0.056 (−0.11, 0.221) | 0.00% |
| IOLMaster vs. OA-2000 | 2 | 0.217 (−0.028, 0.463) | 0.00% |
| Lenstar vs. IOLMaster 700 | 2 | 0.00 (−0.278, 0.278) | 0.00% |
| IOLMaster 500 vs. Aladdin | 2 | 0.171 (−0.271, 0.614) | 0.00% |
| IOLMaster 500 vs. Lenstar | 4 | 0.063 (−0.086, 0.212) | 0.00% |
| IOLMaster 500 vs. OA-2000 | 3 | 0.044 (−0.163, 0.251) | 0.00% |
| Galilei G6 vs. IOLMaster 700 | 2 | −0.024 (−0.533, 0.485) | 0.00% |
| Lenstar vs. Aladdin | 2 | 0.02 (−0.3, 0.34) | 0.00% |
| IOLMaster 500 vs. Pentacam AXL | 2 | 0.25 (−0.02, 0.521) | 0.00% |
| Lenstar vs. Pentacam AXL | 1 | 0.24 (−0.082, 0.562) | 0.00% |
| IOLMaster 500 vs. AL-Scan | 1 | 0.05 (−0.25, 0.35) | 0.00% |
| Galilei G6 vs. IOLMaster 500 | 1 | −0.06 (−0.537, 0.417) | 0.00% |
| Galilei G6 vs. Pentacam AXL | 1 | 0.07 (−0.926, 1.066) | 0.00% |
| IOLMaster 700 vs. Pentacam AXL | 1 | −0.06 (−1.041, 0.921) | 0.00% |
| IOLMaster 700 vs. OA-2000 | 1 | −0.01 (−0.443, 0.423) | 0.00% |

*WMD* = weighted mean differences; *CI* = confidence interval

Appendix I Table 6. Results of direct-comparison meta-analysis in mean keratometry (D)

| Devices | Number of studies | WMD (95% CI) | I^2^ |
| --- | --- | --- | --- |
| IOLMaster 500 vs. IOLMaster 700 | 10 | 0.087 (−0.03, 0.204) | 0.00% |
| Lenstar vs. IOLMaster 700 | 7 | 0.025 (−0.046, 0.095) | 0.00% |
| IOLMaster vs. Lenstar | 13 | 0.06 (−0.03, 0.15) | 0.00% |
| Aladdin vs. IOLMaster 700 | 1 | 0.07 (−0.564, 0.704) | 0.00% |
| AL-Scan vs. Lenstar | 5 | 0.077 (−0.168, 0.323) | 0.00% |
| IOLMaster vs. IOLMaster 500 | 2 | 0.103 (−0.078, 0.284) | 0.00% |
| IOLMaster 500 vs. OA-2000 | 8 | 0.044 (−0.081, 0.169) | 0.00% |
| IOLMaster 500 vs. Lenstar | 9 | 0.016 (−0.073, 0.105) | 0.00% |
| Lenstar vs. OA-2000 | 5 | 0.04 (−0.088, 0.167) | 0.00% |
| IOLMaster vs. OA-2000 | 3 | 0.03 (−0.207, 0.268) | 0.00% |
| IOLMaster 500 vs. AL-Scan | 6 | −0.027 (−0.129, 0.075) | 0.00% |
| IOLMaster 500 vs. Aladdin | 3 | 0.083 (−0.21, 0.376) | 0.00% |
| IOLMaster vs. Argos | 2 | 0.009 (−0.185, 0.202) | 0.00% |
| Galilei G6 vs. IOLMaster 700 | 2 | −0.016 (−0.507, 0.475) | 0.00% |
| Lenstar vs. Aladdin | 2 | 0.063 (−0.199, 0.325) | 0.00% |
| IOLMaster 500 vs. Pentacam AXL | 3 | 0.235 (0.033, 0.436) | 0.00% |
| IOLMaster 700 vs. Argos | 2 | −0.082 (−0.25, 0.086) | 0.00% |
| Lenstar vs. Pentacam AXL | 3 | 0.233 (0.033, 0.434) | 0.00% |
| IOLMaster vs. Aladdin | 1 | 0.16 (−0.147, 0.467) | 0.00% |
| IOLMaster 700 vs. Pentacam AXL | 4 | −0.05 (−0.43, 0.331) | 67.30% |
| IOLMaster 500 vs. Argos | 2 | 0.028 (−0.235, 0.291) | 0.00% |
| Lenstar vs. Argos | 1 | 0 (−0.449, 0.449) | 0.00% |
| Galilei G6 vs. Lenstar | 1 | 0.05 (−0.294, 0.394) | 0.00% |
| Galilei G6 vs. IOLMaster 500 | 1 | −0.05 (−0.524, 0.424) | 0.00% |
| AL-Scan vs. IOLMaster 700 | 1 | 0.2 (−0.379, 0.779) | 0.00% |
| Argos vs. Pentacam AXL | 1 | 0.28 (−0.041, 0.601) | 0.00% |
| Galilei G6 vs. Pentacam AXL | 1 | 0.16 (−0.799, 1.119) | 0.00% |
| IOLMaster 700 vs. OA-2000 | 1 | 0 (−0.408, 0.408) | 0.00% |

*WMD* = weighted mean differences; *CI* = confidence interval

Appendix I Table 7. Results of direct-comparison meta-analysis in J_0_ (D)

| Devices | Number of studies | WMD (95% CI) | I^2^ |
| --- | --- | --- | --- |
| IOLMaster 500 vs. IOLMaster 700 | 1 | 0 (−0.106, 0.106) | 0.00% |
| Lenstar vs. OA-2000 | 1 | 0.03 (−0.069, 0.129) | 0.00% |
| IOLMaster 500 vs. Aladdin | 2 | 0 (−0.007, 0.007) | 0.00% |
| AL-Scan vs. Lenstar | 1 | 0 (−0.125, 0.125) | 0.00% |
| Lenstar vs. Aladdin | 1 | 0.03 (−0.061, 0.121) | 0.00% |
| IOLMaster 700 vs. Pentacam AXL | 2 | 0.012 (−0.086, 0.11) | 0.00% |
| IOLMaster 500 vs. AL-Scan | 1 | 0 (−0.207, 0.207) | 0.00% |
| IOLMaster vs. Lenstar | 1 | −0.03 (−0.14, 0.08) | 0.00% |
| AL-Scan vs. IOLMaster 700 | 1 | −0.03 (−0.213, 0.153) | 0.00% |
| Argos vs. Pentacam AXL | 1 | −0.01 (−0.114, 0.094) | 0.00% |
| IOLMaster 700 vs. ANTERION | 1 | 0 (−0.04, 0.04) | 0.00% |
| IOLMaster 700 vs. OA-2000 | 1 | 0.03 (−0.081, 0.141) | 0.00% |

*J_0_* = anterior corneal power vectors for the cardinal (axes at 90 degrees and 180 degrees) meridians; *WM*D = weighted mean differences; *CI* = confidence interval

Appendix I Table 8. Results of direct-comparison meta-analysis in J_45_ (D)

| Devices | Number of studies | WMD (95% CI) | I^2^ |
| --- | --- | --- | --- |
| IOLMaster 500 vs. IOLMaster 700 | 1 | −0.11 (−0.233, 0.013) | 0.00% |
| Lenstar vs. OA-2000 | 1 | −0.03 (−0.069, 0.009) | 0.00% |
| IOLMaster 500 vs. Aladdin | 2 | 0.01 (−0.064, 0.083) | 0.00% |
| AL-Scan vs. Lenstar | 1 | 0.03 (−0.054, 0.114) | 0.00% |
| Lenstar vs. Aladdin | 1 | −0.02 (−0.065, 0.025) | 0.00% |
| IOLMaster 700 vs. Pentacam AXL | 2 | 0.01 (−0.043, 0.064) | 0.00% |
| IOLMaster 500 vs. AL-Scan | 1 | 0.03 (−0.071, 0.131) | 0.00% |
| IOLMaster vs. Lenstar | 1 | 0 (−0.065, 0.065) | 0.00% |
| AL-Scan vs. IOLMaster 700 | 1 | 0.04 (−0.062, 0.142) | 0.00% |
| Argos vs. Pentacam AXL | 1 | 0.03 (−0.015, 0.075) | 0.00% |
| IOLMaster 700 vs. ANTERION | 1 | 0.03 (−0.011, 0.071) | 0.00% |
| IOLMaster 700 vs. OA-2000 | 1 | −0.01 (−0.126, 0.106) | 0.00% |

*J_45_* = anterior corneal power vectors for the oblique (axes at 45 degrees and 135 degrees) meridians; *WMD* = weighted mean differences; *CI* = confidence interval

Appendix I Table 9. Results of direct-comparison meta-analysis in astigmatism (D)

| Devices | Number of studies | WMD (95% CI) | I^2^ |
| --- | --- | --- | --- |
| IOLMaster 500 vs. IOLMaster 700 | 8 | 0.017 (−0.096, 0.131) | 0.00% |
| IOLMaster vs. Lenstar | 9 | −0.027 (−0.186, 0.131) | 0.00% |
| AL-Scan vs. Lenstar | 4 | −0.064 (−0.222, 0.095) | 0.00% |
| Lenstar vs. OA-2000 | 5 | 0.111 (−0.093, 0.316) | 49.30% |
| IOLMaster vs. OA-2000 | 1 | 0.42 (−0.017, 0.857) | 0.00% |
| Lenstar vs. IOLMaster 700 | 3 | −0.088 (−0.428, 0.253) | 0.00% |
| IOLMaster 500 vs. Aladdin | 2 | 0.246 (−0.352, 0.844) | 0.00% |
| IOLMaster 500 vs. Lenstar | 5 | −0.126 (−0.299, 0.047) | 14.80% |
| IOLMaster 500 vs. OA-2000 | 4 | −0.08 (−0.278, 0.119) | 1.00% |
| Galilei G6 vs. IOLMaster 700 | 2 | 0.016 (−0.409, 0.441) | 0.00% |
| Lenstar vs. Aladdin | 2 | −0.134 (−0.573, 0.304) | 0.00% |
| IOLMaster 500 vs. Pentacam AXL | 3 | 0.036 (−0.147, 0.219) | 0.00% |
| Lenstar vs. Pentacam AXL | 1 | 0.1 (−0.334, 0.534) | 0.00% |
| IOLMaster 500 vs. AL-Scan | 2 | 0.05 (−0.086, 0.186) | 0.00% |
| IOLMaster 700 vs. Pentacam AXL | 2 | 0.034 (−0.16, 0.229) | 0.00% |
| Galilei G6 vs. IOLMaster 500 | 1 | 0.24 (−0.45, 0.93) | 0.00% |
| Galilei G6 vs. Pentacam AXL | 1 | −0.09 (−0.639, 0.459) | 0.00% |
| IOLMaster 700 vs. OA-2000 | 1 | −0.15 (−0.753, 0.453) | 0.00% |

*WMD* = weighted mean differences; *CI* = confidence interval

Appendix I Table 10. Results of direct-comparison meta-analysis in anterior chamber depth (mm)

| Devices | Number of studies | WMD (95% CI) | I^2^ |
| --- | --- | --- | --- |
| IOLMaster 500 vs. IOLMaster 700 | 11 | 0.004 (−0.029, 0.037) | 2.70% |
| Contact ultrasound vs. Lenstar | 5 | 0.003 (−0.228, 0.235) | 96.20% |
| Lenstar vs. IOLMaster 700 | 7 | 0.019 (0.003, 0.035) | 0.00% |
| Contact ultrasound vs. IOLMaster | 19 | −0.133 (−0.183, −0.083) | 75.30% |
| IOLMaster vs. Lenstar | 11 | −0.069 (−0.11, −0.027) | 32.40% |
| Contact ultrasound vs. AL-Scan | 2 | 0.005 (−0.073, 0.084) | 0.00% |
| Aladdin vs. IOLMaster 700 | 1 | 0.04 (−0.104, 0.184) | 0.00% |
| AL-Scan vs. Lenstar | 5 | 0.007 (−0.059, 0.073) | 0.00% |
| IOLMaster vs. IOLMaster 500 | 1 | −0.01 (−0.204, 0.184) | 0.00% |
| Contact ultrasound vs. IOLMaster 700 | 1 | −0.13 (−0.227, −0.033) | 0.00% |
| IOLMaster 500 vs. OA-2000 | 8 | −0.007 (−0.066, 0.052) | 59.40% |
| IOLMaster 500 vs. Lenstar | 8 | −0.065 (−0.153, 0.022) | 87.90% |
| Lenstar vs. OA-2000 | 5 | −0.128 (−0.307, 0.051) | 96.10% |
| IOLMaster vs. OA-2000 | 3 | −0.271 (−0.647, 0.104) | 98.10% |
| Contact ultrasound vs. OA-1000 | 1 | −0.47 (−0.577, −0.363) | 0.00% |
| IOLMaster vs. OA-1000 | 1 | −0.41 (−0.515, −0.305) | 0.00% |
| IOLMaster 500 vs. AL-Scan | 4 | −0.024 (−0.092, 0.044) | 46.50% |
| IOLMaster 500 vs. Aladdin | 3 | −0.065 (−0.155, 0.024) | 37.20% |
| Galilei G6 vs. IOLMaster 700 | 2 | −0.066 (−0.167, 0.036) | 0.00% |
| Contact ultrasound vs. Aladdin | 1 | −0.1 (−0.216, 0.016) | 0.00% |
| Lenstar vs. Aladdin | 2 | 0.004 (−0.062, 0.071) | 0.00% |
| IOLMaster 700 vs. Argos | 2 | −0.113 (−0.179, −0.047) | 0.00% |
| Lenstar vs. Pentacam AXL | 3 | 0.01 (−0.061, 0.08) | 0.00% |
| IOLMaster vs. Aladdin | 1 | −0.03 (−0.096, 0.036) | 0.00% |
| IOLMaster 700 vs. Pentacam AXL | 4 | −0.03 (−0.08, 0.019) | 0.00% |
| IOLMaster 500 vs. Argos | 2 | −0.106 (−0.292, 0.08) | 86.00% |
| Lenstar vs. Argos | 1 | 0.03 (−0.065, 0.125) | 0.00% |
| Galilei G6 vs. Lenstar | 1 | −0.01 (−0.118, 0.098) | 0.00% |
| Galilei G6 vs. IOLMaster 500 | 1 | 0 (−0.121, 0.121) | 0.00% |
| IOLMaster 500 vs. Pentacam AXL | 2 | −0.044 (−0.103, 0.015) | 0.00% |
| AL-Scan vs. IOLMaster 700 | 1 | 0.015 (−0.153, 0.183) | 0.00% |
| Argos vs. Pentacam AXL | 1 | 0.05 (−0.05, 0.15) | 0.00% |
| IOLMaster 700 vs. ANTERION | 1 | −0.07 (−0.129, −0.011) | 0.00% |

*WMD* = weighted mean differences; *CI* = confidence interval

Appendix I Table 11. Results of direct-comparison meta-analysis in aqueous depth (mm)

| Devices | Number of studies | WMD (95% CI) | I^2^ |
| --- | --- | --- | --- |
| Lenstar vs. IOLMaster 700 | 5 | 0.018 (0.002, 0.034) | 0.00% |
| Aladdin vs. IOLMaster 700 | 1 | 0.05 (−0.096, 0.196) | 0.00% |
| AL-Scan vs. Lenstar | 4 | 0.001 (−0.072, 0.073) | 0.00% |
| Contact ultrasound vs. Lenstar | 1 | −0.02 (−0.157, 0.117) | 0.00% |
| Lenstar vs. OA-2000 | 3 | −0.225 (−0.62, 0.17) | 97.90% |
| Galilei G6 vs. IOLMaster 700 | 2 | −0.039 (−0.14, 0.062) | 0.00% |
| IOLMaster 500 vs. Pentacam AXL | 1 | −0.01 (−0.126, 0.106) | 0.00% |
| IOLMaster 700 vs. Argos | 2 | −0.151 (−0.227, −0.074) | 19.60% |
| Lenstar vs. Pentacam AXL | 3 | 0.021 (−0.048, 0.091) | 0.00% |
| IOLMaster 500 vs. Lenstar | 1 | 0 (−0.175, 0.175) | 0.00% |
| Lenstar vs. Argos | 1 | 0.03 (−0.063, 0.123) | 0.00% |
| Lenstar vs. Aladdin | 1 | −0.02 (−0.122, 0.082) | 0.00% |
| AL-Scan vs. IOLMaster 700 | 1 | 0 (−0.169, 0.169) | 0.00% |
| IOLMaster 700 vs. Pentacam AXL | 2 | 0.006 (−0.144, 0.155) | 58.20% |
| Argos vs. Pentacam AXL | 1 | 0.04 (−0.058, 0.138) | 0.00% |
| IOLMaster 700 vs. ANTERION | 1 | −0.08 (−0.142, −0.018) | 0.00% |
| Galilei G6 vs. Pentacam AXL | 1 | 0.01 (−0.17, 0.19) | 0.00% |
| IOLMaster 700 vs. OA-2000 | 1 | 0.02 (−0.051, 0.091) | 0.00% |

*WMD* = weighted mean differences; *CI* = confidence interval

Appendix I Table 12. Results of direct-comparison meta-analysis in central corneal thickness (μm)

| Devices | Number of studies | WMD (95% CI) | I^2^ |
| --- | --- | --- | --- |
| Lenstar vs. IOLMaster 700 | 8 | 0.483 (−1.218, 2.183) | 3.30% |
| Aladdin vs. IOLMaster 700 | 1 | −9.29 (−21.409, 2.829) | 0.00% |
| AL-Scan vs. Lenstar | 5 | 5.717 (0.584, 10.851) | 19.90% |
| Contact ultrasound vs. Lenstar | 1 | 6 (−12.836, 24.836) | 0.00% |
| Lenstar vs. OA-2000 | 3 | 13.683 (9.185, 18.18) | 0.00% |
| Galilei G6 vs. IOLMaster 700 | 2 | 8.503 (−0.296, 17.301) | 12.00% |
| IOLMaster 700 vs. Argos | 2 | 15.182 (−5.808 ,36.172) | 92.60% |
| Lenstar vs. Pentacam AXL | 3 | 9.071 (8.367, 9.774) | 0.00% |
| Lenstar vs. Argos | 1 | 0 (−9.474, 9.474) | 0.00% |
| Lenstar vs. Aladdin | 1 | 10 (−1.032, 21.032) | 0.00% |
| IOLMaster 700 vs. OA-2000 | 2 | 7.247 (−11.664, 26.157) | 90.9% |
| IOLMaster 700 vs. Pentacam AXL | 3 | −4.943 (−14.571, 4.686) | 70.00% |
| OA-2000 vs. Pentacam AXL | 1 | −8.42 (−15.179, −1.661) | 0.00% |
| AL-Scan vs. IOLMaster 700 | 1 | −15.013 (−26.811, −3.215) | 0.00% |
| Argos vs. Pentacam AXL | 1 | −1.16 (−8.081, 5.761) | 0.00% |
| IOLMaster 700 vs. ANTERION | 1 | 5.66 (1.114, 10.206) | 0.00% |
| Galilei G6 vs. Pentacam AXL | 1 | −5.79 (−19.836, 8.256) | 0.00% |

*WMD* = weighted mean differences; *CI* = confidence interval

Appendix I Table 13. Results of direct-comparison meta-analysis in corneal diameter (mm)

| Devices | Number of studies | WMD (95% CI) | I^2^ |
| --- | --- | --- | --- |
| Lenstar vs. IOLMaster 700 | 3 | −0.055 (−0.297, 0.187) | 95.00% |
| IOLMaster vs. Lenstar | 5 | 0.035 (−0.059, 0.128) | 45.90% |
| AL-Scan vs. Lenstar | 4 | −0.148 (−0.229, −0.067) | 0.00% |
| IOLMaster vs. IOLMaster 500 | 1 | 0.27 (0.1, 0.44) | 0.00% |
| Lenstar vs. OA-2000 | 2 | 0.125 (0.049, 0.2) | 0.00% |
| IOLMaster vs. OA-2000 | 2 | 0.172 (0.077, 0.268) | 0.00% |
| IOLMaster 500 vs. OA-2000 | 5 | 0.183 (−0.032, 0.398) | 90.30% |
| Galilei G6 vs. IOLMaster 700 | 1 | −0.06 (−0.183, 0.063) | 0.00% |
| Lenstar vs. Aladdin | 2 | 0.368 (0.283, 0.454) | 0.00% |
| IOLMaster 500 vs. Lenstar | 3 | −0.141 (−0.264, −0.018) | 68.30% |
| IOLMaster 500 vs. Pentacam AXL | 2 | 0.24 (−0.064, 0.544) | 93.40% |
| IOLMaster vs. Aladdin | 1 | 0.39 (0.316, 0.464) | 0.00% |
| IOLMaster 700 vs. Argos | 1 | −0.45 (−0.552, −0.348) | 0.00% |
| IOLMaster 500 vs. IOLMaster 700 | 5 | 0.013 (−0.166, 0.192) | 88.10% |
| Lenstar vs. Argos | 1 | 0.56 (0.405, 0.715) | 0.00% |
| Galilei G6 vs. Lenstar | 1 | 0.16 (0.06, 0.26) | 0.00% |
| IOLMaster 500 vs. AL-Scan | 1 | 0.52 (0.374, 0.666) | 0.00% |
| IOLMaster 700 vs. Pentacam AXL | 2 | 0.281 (0.174, 0.388) | 60.70% |
| AL-Scan vs. IOLMaster 700 | 1 | −0.32 (−0.462, −0.178) | 0.00% |
| IOLMaster 700 vs. OA-2000 | 1 | 0.14 (0.012, 0.268) | 0.00% |
| IOLMaster 700 vs. ANTERION | 1 | 0.12 (−0.036, 0.276) | 0.00% |

*WMD* = weighted mean differences; *CI* = confidence interval

Appendix I Table 14. Results of direct-comparison meta-analysis in lens thickness (mm)

| Devices | Number of studies | WMD (95% CI) | I^2^ |
| --- | --- | --- | --- |
| Lenstar vs. IOLMaster 700 | 9 | −0.03 (−0.08, 0.021) | 51.00% |
| Contact ultrasound vs. Lenstar | 3 | 0.192 (0.054, 0.33) | 88.10% |
| Aladdin vs. IOLMaster 700 | 1 | 0.02 (−0.139, 0.179) | 0.00% |
| Lenstar vs. OA-2000 | 4 | −0.121 (−0.212, −0.031) | 62.00% |
| Galilei G6 vs. IOLMaster 700 | 2 | −0.124 (−0.341, 0.093) | 24.20% |
| IOLMaster 700 vs. Argos | 2 | −0.054 (−0.125, 0.017) | 0.00% |
| Lenstar vs. Argos | 1 | −0.2 (−0.309, −0.091) | 0.00% |
| Galilei G6 vs. Lenstar | 1 | 0.11 (−0.005, 0.225) | 0.00% |
| Lenstar vs. Aladdin | 1 | −0.16 (−0.276, −0.044) | 0.00% |
| IOLMaster 700 vs. OA-2000 | 1 | −0.08 (−0.135, −0.025) | 0.00% |

*WMD* = weighted mean differences; *CI* = confidence interval

Appendix I Table 15. Results of network rank test in axial length

| Devices | SUCRA value (%) | PrBest (%) |
| --- | --- | --- |
| Contact ultrasound | 3.5 | 0.0 |
| OA-1000 | 22.3 | 4.4 |
| IOLMaster | 39.8 | 0.4 |
| Galilei G6 | 47.8 | 9.1 |
| AL-Scan | 49.9 | 2.8 |
| ANTERION | 50.2 | 17.0 |
| IOLMaster 500 | 50.7 | 0.5 |
| IOLMaster 700 | 55.4 | 0.8 |
| OA-2000 | 55.4 | 1.9 |
| Aladdin | 60.3 | 7.0 |
| Argos | 60.5 | 8.5 |
| Pentacam AXL | 60.8 | 8.0 |
| Immersion ultrasound | 63.3 | 30.8 |
| Lenstar | 80.1 | 8.8 |

*SCURA* = surface under the cumulative ranking curve; *PrBest* = the probability of having the maximum valueAppendix I Table 16. Results of network rank test in keratometry in the flattest meridian

| Devices | SUCRA value (%) | PrBest (%) |
| --- | --- | --- |
| Pentacam AXL | 12.8 | 0.5 |
| Aladdin | 38.5 | 6.8 |
| Lenstar | 41.3 | 0.4 |
| OA-2000 | 41.9 | 1.2 |
| Galilei G6 | 48.8 | 20.0 |
| IOLMaster 700 | 52.1 | 5.3 |
| IOLMaster 500 | 63.0 | 3.0 |
| IOLMaster | 75.5 | 22.6 |
| AL-Scan | 76.2 | 40.2 |

*SCURA* = surface under the cumulative ranking curve; *PrBest* = the probability of having the maximum value

Appendix I Table 17. Results of network rank test in keratometry in the steepest meridian

| Devices | SUCRA value (%) | PrBest (%) |
| --- | --- | --- |
| Pentacam AXL | 9.0 | 0.0 |
| OA-2000 | 37.5 | 0.5 |
| Aladdin | 40.1 | 7.6 |
| Galilei G6 | 45.5 | 17.4 |
| IOLMaster 700 | 46.3 | 4.0 |
| Lenstar | 53.5 | 0.8 |
| AL-Scan | 62.8 | 24.6 |
| IOLMaster 500 | 75.3 | 14.0 |
| IOLMaster | 80.1 | 31.1 |

*SCURA* = surface under the cumulative ranking curve; *PrBest* = the probability of having the maximum value

Appendix I Table 18. Results of network rank test in mean keratometry

| Devices | SUCRA value (%) | PrBest (%) |
| --- | --- | --- |
| Pentacam AXL | 2.9 | 0.0 |
| Aladdin | 27.3 | 1.7 |
| IOLMaster 700 | 28.5 | 0.0 |
| OA-2000 | 40.2 | 1.7 |
| Lenstar | 49.2 | 0.0 |
| Galilei G6 | 52.0 | 22.9 |
| IOLMaster 500 | 66.6 | 1.9 |
| Argos | 71.4 | 17.4 |
| AL-Scan | 79.1 | 28.0 |
| IOLMaster | 82.8 | 26.4 |

*SCURA* = surface under the cumulative ranking curve; *PrBest* = the probability of having the maximum value

Appendix I Table 19. Results of network rank test in J_0_

| Devices | SUCRA value (%) | PrBest (%) |
| --- | --- | --- |
| OA-2000 | 36.7 | 4.0 |
| IOLMaster | 43.7 | 14.7 |
| Argos | 45.9 | 18.3 |
| IOLMaster 500 | 46.4 | 2.4 |
| Aladdin | 46.5 | 2.9 |
| Pentacam AXL | 48.8 | 10.9 |
| AL-Scan | 52.6 | 16.8 |
| IOLMaster 700 | 56.4 | 4.4 |
| ANTERION | 57.5 | 10.5 |
| Lenstar | 65.4 | 15.1 |

*J_0_* = anterior corneal power vectors for the cardinal (axes at 90 degrees and 180 degrees) meridians; *SCURA* = surface under the cumulative ranking curve; *PrBest* = the probability of having the maximum value

Appendix I Table 20. Results of network rank test in J_45_

| Devices | SUCRA value (%) | PrBest (%) |
| --- | --- | --- |
| Lenstar | 25.0 | 0.0 |
| IOLMaster | 33.3 | 5.0 |
| ANTERION | 32.9 | 1.4 |
| Aladdin | 45.0 | 3.5 |
| IOLMaster 500 | 46.1 | 7.5 |
| Pentacam AXL | 51.1 | 1.6 |
| AL-Scan | 58.7 | 11.8 |
| OA-2000 | 61.4 | 12.0 |
| IOLMaster 700 | 66.6 | 9.0 |
| Argos | 79.8 | 48.2 |

*J_45_* = anterior corneal power vectors for the oblique (axes at 45 degrees and 135 degrees) meridians; *SCURA* = surface under the cumulative ranking curve; *PrBest* = the probability of having the maximum value

Appendix I Table 21. Results of network rank test in astigmatism

| Devices | SUCRA value (%) | PrBest (%) |
| --- | --- | --- |
| AL-Scan | 28.9 | 1.0 |
| Pentacam AXL | 31.2 | 2.2 |
| IOLMaster 500 | 42.7 | 0.9 |
| IOLMaster 700 | 42.2 | 1.6 |
| OA-2000 | 53.7 | 5.2 |
| Galilei G6 | 54.7 | 28.8 |
| Aladdin | 55.7 | 28.4 |
| IOLMaster | 70.0 | 24.3 |
| Lenstar | 70.9 | 7.6 |

*SCURA* = surface under the cumulative ranking curve; *PrBest* = the probability of having the maximum value

Appendix I Table 22. Results of network rank test in anterior chamber depth

| Devices | SUCRA value (%) | PrBest (%) |
| --- | --- | --- |
| Contact ultrasound | 1.4 | 0.0 |
| Galilei G6 | 29.6 | 0.0 |
| IOLMaster | 29.7 | 0.0 |
| IOLMaster 500 | 32.7 | 0.0 |
| IOLMaster 700 | 36.8 | 0.0 |
| AL-Scan | 37.6 | 0.0 |
| Lenstar | 52.1 | 0.0 |
| Pentacam AXL | 51.7 | 0.0 |
| Aladdin | 58.6 | 0.0 |
| ANTERION | 64.9 | 1.5 |
| Argos | 76.7 | 0.1 |
| OA-2000 | 78.5 | 0.1 |
| OA-1000 | 99.8 | 98.3 |

*SCURA* = surface under the cumulative ranking curve; *PrBest* = the probability of having the maximum value

Appendix I Table 23. Results of network rank test in aqueous depth

| Devices | SUCRA value (%) | PrBest (%) |
| --- | --- | --- |
| Galilei G6 | 34.3 | 2.3 |
| Pentacam AXL | 38.0 | 0.3 |
| Contact ultrasound | 39.4 | 8.8 |
| IOLMaster 500 | 39.4 | 3.8 |
| IOLMaster 700 | 39.9 | 0.1 |
| Lenstar | 40.8 | 0.0 |
| AL-Scan | 41.6 | 1.1 |
| Aladdin | 53.2 | 6.4 |
| ANTERION | 62.9 | 20.4 |
| Argos | 71.8 | 11.0 |
| OA-2000 | 88.8 | 45.8 |

*SCURA* = surface under the cumulative ranking curve; *PrBest* = the probability of having the maximum value

Appendix I Table 24. Results of network rank test in central corneal thickness

| Devices | SUCRA value (%) | PrBest (%) |
| --- | --- | --- |
| OA-2000 | 10.6 | 0.0 |
| Aladdin | 20.4 | 0.5 |
| Argos | 22.4 | 0.0 |
| ANTERION | 38.8 | 3.8 |
| Pentacam AXL | 49.0 | 1.1 |
| Lenstar | 59.5 | 0.4 |
| IOLMaster 700 | 68.8 | 2.0 |
| Contact ultrasound | 73.7 | 45.2 |
| AL-Scan | 74.6 | 10.0 |
| Galilei G6 | 82.1 | 37.0 |

*SCURA* = surface under the cumulative ranking curve; *PrBest* = the probability of having the maximum value

Appendix I Table 25. Results of network rank test in white-to-white

| Devices | SUCRA value (%) | PrBest (%) |
| --- | --- | --- |
| Aladdin | 9.4 | 0.0 |
| Pentacam AXL | 15.2 | 0.0 |
| AL-Scan | 22.2 | 0.0 |
| OA-2000 | 33.9 | 0.0 |
| ANTERION | 42.1 | 9.4 |
| Argos | 60.8 | 13.2 |
| IOLMaster 700 | 63.5 | 1.8 |
| IOLMaster 500 | 66.9 | 3.6 |
| Lenstar | 74.2 | 3.3 |
| Galilei G6 | 77.4 | 35.1 |
| IOLMaster | 84.3 | 33.6 |

*SCURA* = surface under the cumulative ranking curve; *PrBest* = the probability of having the maximum value

Appendix I Table 26. Results of network rank test in lens thickness

| Devices | SUCRA value (%) | PrBest (%) |
| --- | --- | --- |
| Lenstar | 5.7 | 0.0 |
| Galilei G6 | 26.3 | 0.7 |
| IOLMaster 700 | 30.2 | 0.0 |
| Aladdin | 61.0 | 10.7 |
| OA-2000 | 63.7 | 5.2 |
| Argos | 68.8 | 9.9 |
| Contact ultrasound | 94.1 | 73.5 |

*SCURA* = surface under the cumulative ranking curve; *PrBest* = the probability of having the maximum value

Appendix I Table 27. Node-splitting analysis of inconsistency in axial length

| Devices | Direct estimate (95% Cl) | | Indirect estimate (95% Cl) | | Difference (95% Cl) | | *P* value |
| --- | --- | --- | --- | --- | --- | --- | --- |
|  | Coefficient | SE | Coefficient | SE | Coefficient | SE |  |
| Contact ultrasound vs. IOLMaster | 0.1638769 | 0.0369925 | 0.2522724 | 0.0683568 | −0.0883955 | 0.0769597 | 0.251 |
| Contact ultrasound vs. IOLMaster 500 | 0.1269284 | 0.1014758 | 0.2216557 | 0.051569 | −0.0947273 | 0.1135975 | 0.404 |
| Contact ultrasound vs. AL-Scan | 0.1369675 | 0.0976176 | 0.2314087 | 0.0719059 | −0.0944412 | 0.1211983 | 0.436 |
| Contact ultrasound vs. OA-1000 | 0.1799984 | 0.1578059 | −0.3146425 | 0.2824426 | 0.4946409 | 0.319884 | 0.122 |
| Contact ultrasound vs. Lenstar | 0.4252887 | 0.0675476 | 0.1833459 | 0.0468385 | 0.2419428 | 0.0805667 | 0.003 |
| Contact ultrasound vs. Aladdin | 0.1999989 | 0.1845305 | 0.2211661 | 0.0675991 | −0.0211673 | 0.1965226 | 0.914 |
| Contact ultrasound vs. IOLMaster 700 | 0.0699997 | 0.2194396 | 0.2176806 | 0.0561981 | −0.1476809 | 0.2265214 | 0.514 |
| Contact ultrasound vs. Argos | 0.0896014 | 0.2888596 | 0.2247956 | 0.066234 | −0.1351942 | 0.2963165 | 0.648 |
| Immersion ultrasound vs. IOLMaster | 0.0108818 | 0.1780396 | −0.1434436 | 0.1913293 | 0.1543254 | 0.2613481 | 0.555 |
| Immersion ultrasound vs. Lenstar | −0.0799999 | 0.2564527 | 0.0442818 | 0.1535727 | −0.1242818 | 0.298919 | 0.678 |
| Immersion ultrasound vs. OA-2000 | −0.1000004 | 0.2753493 | −0.0138056 | 0.1533347 | −0.0861948 | 0.3151647 | 0.784 |
| Galilei G6 vs. IOLMaster 500 | −0.0200005 | 0.1886337 | 0.0293971 | 0.122272 | −0.0493976 | 0.2247957 | 0.826 |
| Galilei G6 vs. Lenstar | 0.039999 | 0.1478464 | 0.0925066 | 0.1398402 | −0.0525076 | 0.2035039 | 0.796 |
| Galilei G6 vs. IOLMaster 700 | −0.0370385 | 0.2118992 | 0.0395377 | 0.1205824 | −0.0765762 | 0.2435944 | 0.753 |
| Galilei G6 vs. Pentacam AXL | 0.3867986 | 0.2742331 | −0.036502 | 0.1223554 | 0.4233006 | 0.2992998 | 0.157 |
| IOLMaster vs. IOLMaster 500 | −0.0220256 | 0.2365117 | 0.020702 | 0.0452274 | −0.0427276 | 0.2407978 | 0.859 |
| IOLMaster vs. OA-1000 | −0.2399998 | 0.1600207 | 0.2546405 | 0.2786786 | −0.4946403 | 0.319884 | 0.122 |
| IOLMaster vs. Lenstar | 0.0265135 | 0.0568954 | 0.1106381 | 0.0517788 | −0.0841246 | 0.0772058 | 0.276 |
| IOLMaster vs. Aladdin | 0.0227568 | 0.0858064 | 0.0480778 | 0.0849913 | −0.025321 | 0.1211667 | 0.834 |
| IOLMaster vs. IOLMaster 700 | 0.0600014 | 0.29906 | 0.0242373 | 0.0532059 | 0.0357641 | 0.3037561 | 0.906 |
| IOLMaster vs. OA-2000 | −0.008932 | 0.0783445 | 0.050224 | 0.0641189 | −0.059156 | 0.1014138 | 0.56 |
| IOLMaster vs. Argos | 0.0979021 | 0.1063973 | 0.0033036 | 0.0750086 | 0.0945985 | 0.1300998 | 0.467 |
| IOLMaster 500 vs. AL-Scan | −0.0050616 | 0.0774183 | −0.0033762 | 0.0762561 | −0.0016854 | 0.1086602 | 0.988 |
| IOLMaster 500 vs. Lenstar | 0.0731126 | 0.0618299 | 0.0397158 | 0.0498279 | 0.0333968 | 0.0796935 | 0.675 |
| IOLMaster 500 vs. Aladdin | 0.0067723 | 0.1206558 | 0.0198997 | 0.0726827 | −0.0131274 | 0.1408568 | 0.926 |
| IOLMaster 500 vs. IOLMaster 700 | −0.0183846 | 0.0574973 | 0.0353663 | 0.0628586 | −0.0537509 | 0.0851679 | 0.528 |
| IOLMaster 500 vs. OA-2000 | 0.0182668 | 0.06763 | −0.002619 | 0.064552 | 0.0208858 | 0.0934878 | 0.823 |
| IOLMaster 500 vs. Argos | −0.0007816 | 0.0856438 | 0.0296761 | 0.0798426 | −0.0304576 | 0.1175077 | 0.795 |
| IOLMaster 500 vs. Pentacam AXL | −0.0072723 | 0.1045923 | 0.0313393 | 0.0752582 | −0.0386116 | 0.1287262 | 0.764 |
| AL-Scan vs. Lenstar | 0.0016266 | 0.0921324 | 0.0880414 | 0.068506 | −0.0864149 | 0.1148353 | 0.452 |
| AL-Scan vs. IOLMaster 700 | 0.0100002 | 0.3293144 | 0.0103958 | 0.0650732 | −0.0003956 | 0.3356822 | 0.999 |
| Lenstar vs. Aladdin | −0.0011043 | 0.1442821 | −0.0444381 | 0.0680975 | 0.0433338 | 0.1595427 | 0.786 |
| Lenstar vs. IOLMaster 700 | 0.0076271 | 0.0899511 | −0.066488 | 0.0539352 | 0.0741152 | 0.1048828 | 0.48 |
| Lenstar vs. OA-2000 | −2.67E−02 | 0.0803887 | −5.58E−02 | 0.0593162 | 0.0291528 | 0.0998103 | 0.77 |
| Lenstar vs. Argos | −0.0495873 | 0.1567525 | −0.0352108 | 0.065803 | −0.0143765 | 0.1701391 | 0.933 |
| Lenstar vs. Pentacam AXL | −0.0346713 | 0.1039041 | −0.0346268 | 0.0769016 | −0.0000445 | 0.1292929 | 1 |
| Aladdin vs. IOLMaster 700 | −6.58E−10 | 0.391101 | −0.0105661 | 0.0702064 | 0.0105661 | 0.3973524 | 0.979 |
| Aladdin vs. OA-2000 | −0.0203001 | 0.1077669 | −0.0024671 | 0.08297 | −0.017833 | 0.1359398 | 0.896 |
| IOLMaster 700 vs. OA-2000 | −0.0111317 | 0.1055917 | 0.0055817 | 0.0620475 | −0.0167134 | 0.1224691 | 0.891 |
| IOLMaster 700 vs. Argos | −0.025816 | 0.1323494 | 0.0198264 | 0.0717946 | −0.0456424 | 0.1505712 | 0.762 |
| IOLMaster 700 vs. Pentacam AXL | 0.016049 | 0.0864553 | 0.0081067 | 0.0875259 | 0.0079423 | 0.1229548 | 0.948 |
| IOLMaster 700 vs. ANTERION | −0.0099983 | 0.1209813 | −0.2966746 | 723.6344 | 0.2866762 | 723.6344 | 1 |
| OA-2000 vs. Argos | −0.0302078 | 0.1604523 | 0.0161149 | 0.0729317 | −0.0463228 | 0.1762996 | 0.793 |
| Argos vs. Pentacam AXL | −0.0200005 | 0.1896855 | 0.006952 | 0.082555 | −0.0269524 | 0.2068717 | 0.896 |

*CI* = confidence intervals; *SE* = standard error

Appendix I Table 28. Node-splitting analysis of inconsistency in keratometry in the flattest meridian

| Devices | Direct estimate (95% Cl) | | Indirect estimate (95% Cl) | | Overall (95% Cl) | | *P* value |
| --- | --- | --- | --- | --- | --- | --- | --- |
|  | Coefficient | SE | Coefficient | SE | Coefficient | SE |  |
| Galilei G6 vs. IOLMaster 500 | 0.0300026 | 0.2640877 | 0.0594164 | 0.2678534 | −0.0294138 | 0.3761486 | 0.938 |
| Galilei G6 vs. IOLMaster 700 | 0.0339615 | 0.2640569 | −0.0082281 | 0.2718769 | 0.0421895 | 0.3775042 | 0.911 |
| Galilei G6 vs. Pentacam AXL | −0.3294432 | 0.5151152 | −0.1118204 | 0.235994 | −0.2176228 | 0.5665472 | 0.701 |
| IOLMaster vs. lenstar | −0.0450498 | 0.0731999 | −0.5509658 | 0.214118 | 0.505916 | 0.2266042 | 0.026 |
| IOLMaster vs. OA-2000 | −0.221481 | 0.1453115 | −0.0174449 | 0.1240329 | −0.204036 | 0.1918 | 0.287 |
| IOLMaster 500 vs. AL-Scan | 0.0599975 | 0.1735725 | 0.119538 | 0.2380034 | −0.0595405 | 0.2945726 | 0.84 |
| IOLMaster 500 vs. Lenstar | −0.0376027 | 0.0886442 | −0.0788137 | 0.1026912 | 0.041211 | 0.1357366 | 0.761 |
| IOLMaster 500 vs. Aladdin | −0.0927861 | 0.2222991 | −0.0791478 | 0.1809545 | −0.0136384 | 0.286635 | 0.962 |
| IOLMaster 500 vs. IOLMaster 700 | −0.0426553 | 0.1204076 | −0.0168736 | 0.13327 | −0.0257817 | 0.1796147 | 0.886 |
| IOLMaster 500 vs. OA-2000 | −0.0538415 | 0.1176496 | −0.0672025 | 0.1204801 | 0.0133609 | 0.1680401 | 0.937 |
| IOLMaster 500 vs. Pentacam AXL | −0.1955938 | 0.1576561 | −0.1914353 | 0.1829347 | −0.0041584 | 0.241507 | 0.986 |
| AL-Scan vs. Lenstar | −0.1716092 | 0.2278791 | −0.1120688 | 0.1866758 | −0.0595404 | 0.2945698 | 0.84 |
| Lenstar vs. Aladdin | −0.0245959 | 0.1674135 | −0.0382346 | 0.23266 | 0.0136387 | 0.2866376 | 0.962 |
| Lenstar vs. IOLMaster 700 | 0.0238862 | 0.1590079 | 0.0242631 | 0.1185154 | −0.0003769 | 0.1982914 | 0.998 |
| Lenstar vs. OA-2000 | 0.1373481 | 0.0854986 | −0.2331144 | 0.1149942 | 0.3704625 | 0.1442688 | 0.01 |
| Lenstar vs. Pentacam AXL | −0.1300011 | 0.1862776 | −0.1451836 | 0.163575 | 0.0151824 | 0.2479035 | 0.951 |
| IOLMaster 700 vs. OA-2000 | −0.0499992 | 0.2222487 | −0.0226663 | 0.1207805 | −0.0273329 | 0.2529475 | 0.914 |
| IOLMaster 700 vs. Pentacam AXL | −0.0649675 | 0.4871216 | −0.1718086 | 0.1459085 | 0.1068412 | 0.507809 | 0.833 |

*CI* = confidence intervals; *SE* = standard error

Appendix I Table 29. Node-splitting analysis of inconsistency in keratometry in the steepest meridian

| Devices | Direct estimate (95% Cl) | | Indirect estimate (95% Cl) | | Overall (95% Cl) | | *P* value |
| --- | --- | --- | --- | --- | --- | --- | --- |
|  | Coefficient | SE | Coefficient | SE | Coefficient | SE |  |
| Galilei G6 vs. IOLMaster 500 | 0.0600014 | 0.2434809 | 0.1329097 | 0.2612927 | −0.0729083 | 0.357151 | 0.838 |
| Galilei G6 vs. IOLMaster 700 | 0.0230581 | 0.2597691 | 0.0063429 | 0.2529515 | 0.0167152 | 0.3622514 | 0.963 |
| Galilei G6 vs. Pentacam AXL | −0.0689206 | 0.5080715 | −0.1833866 | 0.2189632 | 0.114466 | 0.5531472 | 0.836 |
| IOLMaster vs. Lenstar | −0.0599841 | 0.064578 | −0.307636 | 0.2051487 | 0.2476519 | 0.2153258 | 0.25 |
| IOLMaster vs. OA-2000 | −0.2215936 | 0.1252373 | −0.0597925 | 0.1088829 | −0.1618011 | 0.1661001 | 0.33 |
| IOLMaster 500 vs. AL-Scan | −0.0499992 | 0.1531946 | 0.0611243 | 0.2628609 | −0.1111235 | 0.304244 | 0.715 |
| IOLMaster 500 vs. Lenstar | −0.0626334 | 0.0760012 | −0.0449949 | 0.0974319 | −0.0176385 | 0.1235893 | 0.887 |
| IOLMaster 500 vs. Aladdin | −0.1713864 | 0.2256174 | −0.0713267 | 0.1742901 | −0.1000597 | 0.2850969 | 0.726 |
| IOLMaster 500 vs. IOLMaster 700 | −0.0816907 | 0.1184303 | −0.0769277 | 0.1234574 | −0.004763 | 0.1710774 | 0.978 |
| IOLMaster 500 vs. OA-2000 | −0.0439987 | 0.1056559 | −0.1664294 | 0.1097753 | 0.1224307 | 0.1524222 | 0.422 |
| IOLMaster 500 vs. Pentacam AXL | −0.2504796 | 0.1378482 | −0.2720101 | 0.1645779 | 0.0215304 | 0.2146812 | 0.92 |
| AL-Scan vs. Lenstar | −0.1125933 | 0.2556458 | −0.0014697 | 0.1649535 | −0.1111236 | 0.304244 | 0.715 |
| Lenstar vs. Aladdin | −0.0200004 | 0.1631344 | −0.1200604 | 0.2338106 | 0.10006 | 0.2850969 | 0.726 |
| Lenstar vs. IOLMaster 700 | 4.20E−11 | 0.1418331 | −0.038427 | 0.1133616 | 0.038427 | 0.1815695 | 0.832 |
| Lenstar vs. OA-2000 | −0.0558014 | 0.0845142 | −0.029743 | 0.1179738 | −0.0260584 | 0.1459636 | 0.858 |
| Lenstar vs. Pentacam AXL | −0.2399979 | 0.164319 | −0.1748593 | 0.1450926 | −0.0651386 | 0.219209 | 0.766 |
| IOLMaster 700 vs. OA-2000 | 0.0099983 | 0.2208655 | −0.0320381 | 0.1117717 | 0.0420364 | 0.2475368 | 0.865 |
| IOLMaster 700 vs. Pentacam AXL | 0.06029 | 0.5006917 | −0.1970399 | 0.1337391 | 0.2573299 | 0.5181395 | 0.619 |

*CI* = confidence intervals; *SE* = standard error

Appendix I Table 30. Node-splitting analysis of inconsistency in mean keratometry

| Devices | Direct estimate (95% Cl) | | Indirect estimate (95% Cl) | | Overall (95% Cl) | | *P* value |
| --- | --- | --- | --- | --- | --- | --- | --- |
|  | Coefficient | SE | Coefficient | SE | Coefficient | SE |  |
| Galilei G6 vs. IOLMaster 500 | 0.0500031 | 0.241964 | 0.0229895 | 0.1454885 | 0.0270136 | 0.2823358 | 0.924 |
| Galilei G6 vs. Lenstar | −0.0499992 | 0.1757027 | 0.0467211 | 0.1728413 | −0.0967204 | 0.2464662 | 0.695 |
| Galilei G6 vs. IOLMaster 700 | 0.0137929 | 0.2504318 | −0.0618908 | 0.1432127 | 0.0756836 | 0.2879862 | 0.793 |
| Galilei G6 vs. Pentacam AXL | −0.1563212 | 0.4890819 | −0.1781805 | 0.1400377 | 0.0218593 | 0.5084992 | 0.966 |
| IOLMaster vs. IOLMaster 500 | −0.103259 | 0.0923845 | −0.0134406 | 0.0540382 | −0.0898184 | 0.10705 | 0.401 |
| IOLMaster vs. Lenstar | −0.0603509 | 0.0458982 | −0.0881707 | 0.0791348 | 0.0278198 | 0.091483 | 0.761 |
| IOLMaster vs. Aladdin | −1.60E−01 | 0.1567475 | −0.116146 | 0.1034244 | −0.0438538 | 0.1877934 | 0.815 |
| IOLMaster vs. OA-2000 | −0.0089054 | 0.1168365 | −0.1143203 | 0.0679449 | 0.1054149 | 0.1339816 | 0.431 |
| IOLMaster vs. Argos | −0.0086706 | 0.0987953 | −0.0291043 | 0.0802519 | 0.0204337 | 0.1272827 | 0.872 |
| IOLMaster 500 vs. AL-Scan | 0.0269002 | 0.0518625 | 0.0607997 | 0.1198352 | −0.0338995 | 0.1305764 | 0.795 |
| IOLMaster 500 vs. Lenstar | −0.0157559 | 0.0452364 | −0.0486547 | 0.0486591 | 0.0328988 | 0.0664403 | 0.62 |
| IOLMaster 500 vs. Aladdin | −0.0830377 | 0.1493977 | −0.0978569 | 0.1027025 | 0.0148192 | 0.1812939 | 0.935 |
| IOLMaster 500 vs. IOLMaster 700 | −0.0868662 | 0.0596633 | −0.0648607 | 0.0478254 | −0.0220055 | 0.0764706 | 0.774 |
| IOLMaster 500 vs. OA-2000 | −0.0456977 | 0.0637516 | −0.0626439 | 0.0852099 | 0.0169461 | 0.1067221 | 0.874 |
| IOLMaster 500 vs. Argos | −0.0275821 | 0.1342413 | 0.0266595 | 0.0688959 | −0.0542416 | 0.1508219 | 0.719 |
| IOLMaster 500 vs. Pentacam AXL | −0.2348231 | 0.1027911 | −0.1903666 | 0.0783087 | −0.0444564 | 0.1292505 | 0.731 |
| AL-Scan vs. Lenstar | −0.0774384 | 0.1250967 | −0.0598794 | 0.0610355 | −0.017559 | 0.1391924 | 0.9 |
| AL-Scan vs. IOLMaster 700 | −0.1999969 | 0.2951955 | −0.1019338 | 0.0591348 | −0.0980631 | 0.3010603 | 0.745 |
| Lenstar vs. Aladdin | −0.0632564 | 0.1336358 | −0.0613628 | 0.1059198 | −0.0018936 | 0.1705213 | 0.991 |
| Lenstar vs. IOLMaster 700 | −0.0246317 | 0.0359796 | −0.0863517 | 0.0564483 | 0.06172 | 0.0669377 | 0.357 |
| Lenstar vs. OA-2000 | −0.0505148 | 0.0641766 | 0.0236437 | 0.07784 | −0.0741585 | 0.0989424 | 0.454 |
| Lenstar vs. Argos | 2.83E−11 | 0.2291288 | 0.049667 | 0.0610712 | −0.049667 | 0.237128 | 0.834 |
| Lenstar vs. Pentacam AXL | −0.2331701 | 0.102274 | −0.1448757 | 0.0748787 | −0.0882944 | 0.1267549 | 0.486 |
| Aladdin vs. IOLMaster 700 | −0.0699997 | 0.323259 | 0.0264988 | 0.0894752 | −0.0964985 | 0.3354134 | 0.774 |
| IOLMaster 700 vs. OA-2000 | 6.52E−11 | 0.2083243 | 0.0233229 | 0.0574144 | −0.0233229 | 0.2160913 | 0.914 |
| IOLMaster 700 vs. Argos | 0.0819381 | 0.0857455 | 0.0947576 | 0.0794678 | −0.0128195 | 0.1169077 | 0.913 |
| IOLMaster 700 vs. Pentacam AXL | −0.0374751 | 0.0994032 | −0.19208 | 0.0779031 | 0.1546049 | 0.1263855 | 0.221 |
| Argos vs. Pentacam AXL | −0.2800026 | 0.1638355 | −0.2062233 | 0.0856538 | −0.0737793 | 0.1848747 | 0.69 |

*CI* = confidence intervals; *SE* = standard error

Appendix I Table 31. Node-splitting analysis of inconsistency in J_0_

| Devices | Direct estimate (95% Cl) | | Indirect estimate (95% Cl) | | Overall (95% Cl) | | *P* value |
| --- | --- | --- | --- | --- | --- | --- | --- |
|  | Coefficient | SE | Coefficient | SE | Coefficient | SE |  |
| IOLMaster vs. Lenstar | 0.03 | 0.0563205 | 0.0198593 | 6.523439 | 0.0101407 | 6.523682 | 0.999 |
| IOLMaster 500 vs. AL-Scan | −1.03E−10 | 0.1058059 | 0.0086109 | 0.0634437 | −0.0086109 | 0.1233693 | 0.944 |
| IOLMaster 500 vs. Aladdin | −0.0000478 | 0.0037766 | −0.0345306 | 0.0856541 | 0.0344828 | 0.0857373 | 0.688 |
| IOLMaster 500 vs. IOLMaster 700 | −2.68E−10 | 0.0542911 | 0.0344416 | 0.0734875 | −0.0344416 | 0.091367 | 0.706 |
| AL-Scan vs. Lenstar | 1.93E−10 | 0.0635542 | 0.0356876 | 0.0813546 | −0.0356876 | 0.1032361 | 0.73 |
| AL-Scan vs. IOLMaster 700 | 0.03 | 0.0932016 | −0.0085228 | 0.0718103 | 0.0385228 | 0.1176574 | 0.743 |
| Lenstar vs. Aladdin | −3.00E−02 | 0.0462305 | 0.0044797 | 0.0722009 | −0.0344797 | 0.0857335 | 0.688 |
| Lenstar vs. OA-2000 | −0.03 | 0.0504625 | −0.0425742 | 0.0825608 | 0.0125742 | 0.0967613 | 0.897 |
| IOLMaster 700 vs. OA-2000 | −0.03 | 0.0564397 | −0.017426 | 0.0785959 | −0.012574 | 0.0967613 | 0.897 |
| IOLMaster 700 vs. Pentacam AXL | −0.012202 | 0.0501306 | −0.0276452 | 109.9428 | 0.0154433 | 109.9428 | 1 |
| IOLMaster 700 vs. ANTERION | −8.44E−10 | 0.0205698 | −0.0313652 | 141.1353 | 0.0313652 | 141.1353 | 1 |
| Argos vs. Pentacam AXL | 0.01 | 0.052902 | 0.0148458 | 278.7067 | −0.0048458 | 278.7067 | 1 |

*J_0_* = anterior corneal power vectors for the cardinal (axes at 90 degrees and 180 degrees) meridians; *CI* = confidence intervals; *SE* = standard error

Appendix I Table 32. Node-splitting analysis of inconsistency in J_45_

| Devices | Direct estimate (95% Cl) | | Indirect estimate (95% Cl) | | Overall (95% Cl) | | *P* value |
| --- | --- | --- | --- | --- | --- | --- | --- |
|  | Coefficient | SE | Coefficient | SE | Coefficient | SE |  |
| IOLMaster vs. Lenstar | −5.41E−10 | 0.0329848 | −0.0240942 | 22.1635 | 0.0240942 | 22.16353 | 0.999 |
| IOLMaster 500 vs. AL-Scan | −3.00E−02 | 0.0513539 | 0.0535879 | 0.0489492 | −0.0835879 | 0.0709454 | 0.239 |
| IOLMaster 500 vs. Aladdin | −0.009645 | 0.0374506 | 0.0189061 | 0.058033 | −0.0285511 | 0.0690679 | 0.679 |
| IOLMaster 500 vs. IOLMaster 700 | 1.10E−01 | 0.0626241 | −0.0406233 | 0.0523948 | 0.1506232 | 0.0816517 | 0.065 |
| AL-Scan vs. Lenstar | −3.00E−02 | 0.0427836 | −0.0345496 | 0.0517004 | 0.0045496 | 0.0671071 | 0.946 |
| AL-Scan vs. IOLMaster 700 | −0.04 | 0.05198 | 0.0612911 | 0.0552048 | −0.1012911 | 0.0758254 | 0.182 |
| Lenstar vs. Aladdin | 2.00E−02 | 0.0231152 | −0.0085518 | 0.065085 | 0.0285518 | 0.0690679 | 0.679 |
| Lenstar vs. OA-2000 | 0.03 | 0.0199494 | 0.0621285 | 0.0778467 | −0.0321285 | 0.0803623 | 0.689 |
| IOLMaster 700 vs. OA-2000 | 0.01 | 0.0592592 | −0.0221291 | 0.0542812 | 0.0321291 | 0.0803623 | 0.689 |
| IOLMaster 700 vs. Pentacam AXL | −0.0104964 | 0.0274805 | −0.0597537 | 54.06605 | 0.0492572 | 54.06605 | 0.999 |
| IOLMaster 700 vs. ANTERION | −3.00E−02 | 0.020915 | −0.0739366 | 134.98 | 0.0439366 | 134.98 | 1 |
| Argos vs. Pentacam AXL | −0.03 | 0.0229091 | 0.0582107 | 119.6092 | −0.0882107 | 119.6092 | 0.999 |

*J_45_* = anterior corneal power vectors for the oblique (axes at 45 degrees and 135 degrees) meridians; *CI* = confidence intervals; *SE* = standard error

Appendix I Table 33. Node-splitting analysis of inconsistency in astigmatism

| Devices | Direct estimate (95% Cl) | | Indirect estimate (95% Cl) | | Overall (95% Cl) | | *P* value |
| --- | --- | --- | --- | --- | --- | --- | --- |
|  | Coefficient | SE | Coefficient | SE | Coefficient | SE |  |
| Galilei G6 vs. IOLMaster 500 | −2.40E−01 | 0.3520799 | 0.0309661 | 0.2038857 | −0.270966 | 0.4068533 | 0.505 |
| Galilei G6 vs. IOLMaster 700 | −1.26E−02 | 0.216884 | −0.0949994 | 0.299198 | 0.0824321 | 0.3717897 | 0.825 |
| Galilei G6 vs. Pentacam AXL | 0.0944939 | 0.2781931 | −0.2052543 | 0.2538237 | 0.2997482 | 0.3877454 | 0.439 |
| IOLMaster vs. Lenstar | 2.73E−02 | 0.0807747 | −1.080697 | 0.4139243 | 1.107986 | 0.4223414 | 0.009 |
| IOLMaster vs. OA-2000 | −3.86E−01 | 0.2226066 | 0.0259525 | 0.1053532 | −0.4120667 | 0.2468951 | 0.095 |
| IOLMaster 500 vs. AL-Scan | −0.0499972 | 0.0692042 | 0.0133687 | 0.1036014 | −0.0633659 | 0.1245893 | 0.611 |
| IOLMaster 500 vs. Lenstar | 1.22E−01 | 0.0808077 | 0.0046405 | 0.0760266 | 0.1175838 | 0.1108893 | 0.289 |
| IOLMaster 500 vs. Aladdin | −0.2462538 | 0.305029 | 0.2037076 | 0.2306248 | −0.4499614 | 0.3824009 | 0.239 |
| IOLMaster 500 vs. IOLMaster 700 | −0.017283 | 0.0579467 | 0.0700305 | 0.1370142 | −0.0873135 | 0.1487639 | 0.557 |
| IOLMaster 500 vs. OA-2000 | 0.0805301 | 0.1002382 | −0.0273894 | 0.0966024 | 0.1079195 | 0.1392263 | 0.438 |
| IOLMaster 500 vs. Pentacam AXL | −3.58E−02 | 0.0933903 | −0.0204377 | 0.144052 | −0.0153332 | 0.1719646 | 0.929 |
| AL-Scan vs. Lenstar | 0.0636759 | 0.0807498 | 0.1270406 | 0.0948786 | −0.0633646 | 0.1245893 | 0.611 |
| Lenstar vs. Aladdin | 0.1342086 | 0.223721 | −0.3157528 | 0.310128 | 0.4499614 | 0.3824009 | 0.239 |
| Lenstar vs. IOLMaster 700 | 0.0876752 | 0.1736339 | −0.0945586 | 0.0781171 | 0.1822338 | 0.190397 | 0.339 |
| Lenstar vs. OA-2000 | −0.0527923 | 0.0616811 | 0.0784758 | 0.1564507 | −0.1312681 | 0.1679342 | 0.434 |
| Lenstar vs. Pentacam AXL | −0.1 | 0.2213545 | −0.0892876 | 0.0988971 | −0.0107124 | 0.2424427 | 0.965 |
| IOLMaster 700 vs. OA-2000 | 0.15 | 0.3075443 | 0.0191056 | 0.0860713 | 0.1308944 | 0.3193615 | 0.682 |
| IOLMaster 700 vs. Pentacam AXL | −0.036028 | 0.0992382 | −0.0092479 | 0.1413418 | −0.0267801 | 0.1726566 | 0.877 |

*CI* = confidence intervals; *SE* = standard error

Appendix I Table 34. Node-splitting analysis of inconsistency in anterior chamber depth

| Devices | Direct estimate (95% Cl) | | Indirect estimate (95% Cl) | | Overall (95% Cl) | | *P* value |
| --- | --- | --- | --- | --- | --- | --- | --- |
|  | Coefficient | SE | Coefficient | SE | Coefficient | SE |  |
| Contact ultrasound vs. IOLMaster | 1.33E−01 | 0.0261938 | −0.0460233 | 0.0477302 | 0.1790627 | 0.0544389 | 0.001 |
| Contact ultrasound vs. AL-Scan | −1.34E−03 | 0.0794795 | 0.1406261 | 0.0489035 | −0.1419631 | 0.0933215 | 0.128 |
| Contact ultrasound vs. OA-1000 | 0.47 | 0.1126059 | 0.5354107 | 0.198881 | −0.0654107 | 0.227614 | 0.774 |
| Contact ultrasound vs. Lenstar | 1.30E−02 | 0.0516123 | 0.1703416 | 0.034247 | −0.1573602 | 0.0619428 | 0.011 |
| Contact ultrasound vs. Aladdin | 1.00E−01 | 0.1148491 | 0.1436414 | 0.0511587 | −0.0436415 | 0.125728 | 0.729 |
| Contact ultrasound vs. IOLMaster 700 | 0.1300001 | 0.1103026 | 0.0990797 | 0.0386152 | 0.0309204 | 0.1168666 | 0.791 |
| Galilei G6 vs. IOLMaster 500 | −4.85E−10 | 0.1162276 | 0.0217077 | 0.0698919 | −0.0217077 | 0.1356234 | 0.873 |
| Galilei G6 vs. Lenstar | 0.0099999 | 0.1127075 | 0.0516989 | 0.0708392 | −0.0416989 | 0.1331209 | 0.754 |
| Galilei G6 vs. IOLMaster 700 | 0.0732604 | 0.0880433 | −0.0235122 | 0.0804295 | 0.0967725 | 0.1195797 | 0.418 |
| Galilei G6 vs. Pentacam AXL | 0.0032085 | 0.1337292 | 0.0546718 | 0.0767905 | −0.0514633 | 0.1544344 | 0.739 |
| IOLMaster vs. IOLMaster 500 | 1.00E−02 | 0.1393702 | 0.0064712 | 0.0335834 | 0.003529 | 0.1433593 | 0.98 |
| IOLMaster vs. OA-1000 | 0.4100001 | 0.1120866 | 0.3445881 | 0.1997599 | 0.0654121 | 0.2276144 | 0.774 |
| IOLMaster vs. Lenstar | 0.0714114 | 0.0351401 | −0.0192692 | 0.0389387 | 0.0906807 | 0.0524625 | 0.084 |
| IOLMaster vs. Aladdin | 0.03 | 0.1040829 | 0.0488285 | 0.0505723 | −0.0188285 | 0.1157186 | 0.871 |
| IOLMaster vs. OA-2000 | 0.2512111 | 0.0596214 | −0.0008845 | 0.042897 | 0.2520955 | 0.0734268 | 0.001 |
| IOLMaster 500 vs. AL-Scan | 0.0309216 | 0.0562538 | −0.0180408 | 0.0503322 | 0.0489623 | 0.0754807 | 0.517 |
| IOLMaster 500 vs. Lenstar | 0.0653327 | 0.039083 | −0.0033283 | 0.0317675 | 0.0686609 | 0.0503645 | 0.173 |
| IOLMaster 500 vs. Aladdin | 0.0708066 | 0.0677674 | 0.0169328 | 0.055487 | 0.0538738 | 0.0875856 | 0.538 |
| IOLMaster 500 vs. IOLMaster 700 | −0.0015391 | 0.034908 | 0.011852 | 0.0378992 | −0.0133911 | 0.0515278 | 0.795 |
| IOLMaster 500 vs. OA-2000 | 0.011138 | 0.0400448 | 0.1771774 | 0.0483974 | −0.1660394 | 0.0628019 | 0.008 |
| IOLMaster 500 vs. Argos | 0.1064429 | 0.0781053 | 0.0682061 | 0.0660346 | 0.0382368 | 0.1023344 | 0.709 |
| IOLMaster 500 vs. Pentacam AXL | 0.0448584 | 0.0759967 | 0.017949 | 0.0493198 | 0.0269094 | 0.0905894 | 0.766 |
| AL-Scan vs. Lenstar | −0.0004388 | 0.0567775 | 0.0350837 | 0.0481459 | −0.0355226 | 0.0744491 | 0.633 |
| AL-Scan vs. IOLMaster 700 | −0.0150001 | 0.1303527 | 0.0026041 | 0.0430106 | −0.0176042 | 0.1372653 | 0.898 |
| Lenstar vs. Aladdin | −0.0048832 | 0.0776079 | 0.0229723 | 0.0507977 | −0.0278554 | 0.0927547 | 0.764 |
| Lenstar vs. IOLMaster 700 | −0.0168368 | 0.0419027 | −0.0210734 | 0.0348818 | 0.0042366 | 0.054523 | 0.938 |
| Lenstar vs. OA-2000 | 0.1169671 | 0.0478491 | 0.0049261 | 0.0428335 | 0.112041 | 0.064174 | 0.081 |
| Lenstar vs. Argos | −0.0283175 | 0.109298 | 0.0856188 | 0.0584855 | −0.1139362 | 0.124072 | 0.358 |
| Lenstar vs. Pentacam AXL | −0.0095294 | 0.0721392 | 0.0075818 | 0.0503368 | −0.0171112 | 0.0879649 | 0.846 |
| Aladdin vs. IOLMaster 700 | −0.0400001 | 0.1227569 | −0.0329923 | 0.0489821 | −0.0070078 | 0.1321685 | 0.958 |
| IOLMaster 700 vs. OA-2000 | 3.13E−12 | 0.1046246 | 0.0843797 | 0.038546 | −0.0843797 | 0.1114993 | 0.449 |
| IOLMaster 700 vs. Argos | 0.1106524 | 0.0779351 | 0.0579038 | 0.0650005 | 0.0527486 | 0.1014834 | 0.603 |
| IOLMaster 700 vs. Pentacam AXL | 0.0142756 | 0.0576376 | 0.0280902 | 0.0565327 | −0.0138146 | 0.0807462 | 0.864 |
| IOLMaster 700 vs. ANTERION | 0.0699999 | 0.1023249 | −0.2823112 | 264.7781 | 0.3523112 | 264.7781 | 0.999 |
| Argos vs. Pentacam AXL | −0.05 | 0.1109576 | −0.0611893 | 0.0666049 | 0.0111894 | 0.1294133 | 0.931 |

*CI* = confidence intervals; *SE* = Standard error

Appendix I Table 35. Node-splitting analysis of inconsistency in aqueous depth

| Devices | Direct estimate (95% Cl) | | Indirect estimate (95% Cl) | | Overall (95% Cl) | | *P* value |
| --- | --- | --- | --- | --- | --- | --- | --- |
|  | Coefficient | SE | Coefficient | SE | Coefficient | SE |  |
| Contact ultrasound vs. Lenstar | 2.00E−02 | 0.1453869 | −0.0084014 | 15.85157 | 0.0284014 | 15.85224 | 0.999 |
| Galilei G6 vs. IOLMaster 700 | 5.08E−02 | 0.106478 | −0.1461806 | 0.312628 | 0.1969678 | 0.3313072 | 0.552 |
| Galilei G6 vs. Pentacam AXL | −0.0080079 | 0.1596233 | 0.0564607 | 0.1540979 | −0.0644686 | 0.2225343 | 0.772 |
| IOLMaster 500 vs. Lenstar | −3.78E−08 | 0.1585489 | 0.0183017 | 0.1578512 | −0.0183017 | 0.2237292 | 0.935 |
| IOLMaster 500 vs. Pentacam AXL | 1.00E−02 | 0.1436466 | −0.0083016 | 0.171524 | 0.0183016 | 0.2237294 | 0.935 |
| AL-Scan vs. Lenstar | 0.0049147 | 0.0768724 | 0.0019984 | 0.1640992 | 0.0029162 | 0.1812136 | 0.987 |
| AL-Scan vs. IOLMaster 700 | 1.39E−10 | 0.1566757 | 0.0029163 | 0.0910557 | −0.0029163 | 0.1812138 | 0.987 |
| Lenstar vs. Aladdin | 0.0199999 | 0.1410851 | 0.0493202 | 0.1583278 | −0.0293203 | 0.2120677 | 0.89 |
| Lenstar vs. IOLMaster 700 | −0.007939 | 0.0621936 | 0.005559 | 0.0723609 | −0.0134981 | 0.0954156 | 0.888 |
| Lenstar vs. OA-2000 | 0.2149929 | 0.0762121 | −0.0455157 | 0.1347885 | 0.2605087 | 0.1548308 | 0.092 |
| Lenstar vs. Argos | −3.00E−02 | 0.1362727 | 0.1263166 | 0.0905325 | −0.1563167 | 0.1636043 | 0.339 |
| Lenstar vs. Pentacam AXL | −0.022848 | 0.0884139 | 0.0093714 | 0.0883759 | −0.0322195 | 0.1250067 | 0.797 |
| Aladdin vs. IOLMaster 700 | −0.0500002 | 0.1507536 | −0.0206798 | 0.1491509 | −0.0293205 | 0.2120675 | 0.89 |
| IOLMaster 700 vs. OA-2000 | −0.02 | 0.1266244 | 0.2405086 | 0.0891001 | −0.2605086 | 0.1548308 | 0.092 |
| IOLMaster 700 vs. Argos | 0.1583598 | 0.0959734 | −0.0160991 | 0.1071504 | 0.174459 | 0.1438508 | 0.225 |
| IOLMaster 700 vs. Pentacam AXL | −0.0163917 | 0.1047391 | 0.0029056 | 0.0830379 | −0.0192973 | 0.1336268 | 0.885 |
| IOLMaster 700 vs. ANTERION | 0.0800002 | 0.1313655 | −0.0397797 | 284.4358 | 0.1197799 | 284.4358 | 1 |
| Argos vs. Pentacam AXL | −0.04 | 0.1400963 | −0.1099172 | 0.1035965 | 0.0699172 | 0.1742389 | 0.688 |

*CI* = confidence intervals; *SE* = standard error

Appendix I Table 36. Node-splitting analysis of inconsistency in central corneal thickness

| Devices | Direct estimate (95% Cl) | | Indirect estimate (95% Cl) | | Overall (95% Cl) | | *P* value |
| --- | --- | --- | --- | --- | --- | --- | --- |
|  | Coefficient | SE | Coefficient | SE | Coefficient | SE |  |
| Contact ultrasound vs. Lenstar | −6 | 11.51023 | 0.7304122 | 759.3622 | −6.730412 | 759.4494 | 0.993 |
| Galilei G6 vs. IOLMaster 700 | −7.855086 | 6.075236 | 23.92055 | 17.50595 | −31.77564 | 18.55676 | 0.087 |
| Galilei G6 vs. Pentacam AXL | 5.813776 | 9.34678 | −17.23937 | 8.004199 | 23.05315 | 12.27776 | 0.06 |
| AL-Scan vs. Lenstar | −5.576143 | 3.520686 | 15.09111 | 8.583449 | −20.66725 | 9.278667 | 0.026 |
| AL-Scan vs. IOLMaster 700 | 15.013 | 8.309409 | −5.65429 | 4.12886 | 20.66729 | 9.278673 | 0.026 |
| Lenstar vs. Aladdin | −9.999998 | 8.589648 | −8.079436 | 9.256796 | −1.920562 | 12.62816 | 0.879 |
| Lenstar vs. IOLMaster 700 | 1.480075 | 2.823042 | 0.5370428 | 3.845786 | 0.9430325 | 4.762546 | 0.843 |
| Lenstar vs. OA-2000 | −13.71282 | 4.377455 | −7.925305 | 5.422022 | −5.787514 | 6.968558 | 0.406 |
| Lenstar vs. Argos | 0.000000267 | 7.910381 | −10.559 | 4.776874 | 10.559 | 9.240815 | 0.253 |
| Lenstar vs. Pentacam AXL | −7.292453 | 4.499523 | 2.579841 | 4.182376 | −9.872295 | 6.12638 | 0.107 |
| Aladdin vs. IOLMaster 700 | 9.289978 | 8.962793 | 11.21054 | 8.89595 | −1.920564 | 12.62813 | 0.879 |
| IOLMaster 700 vs. OA-2000 | −6.825061 | 5.184529 | −17.13408 | 4.653057 | 10.30902 | 6.968919 | 0.139 |
| IOLMaster 700 vs. Argos | −1.48E+01 | 5.153619 | −1.592852 | 5.674002 | −13.23073 | 7.665783 | 0.084 |
| IOLMaster 700 vs. Pentacam AXL | 4.890584 | 4.036869 | −10.39134 | 3.80145 | 15.28192 | 5.554161 | 0.006 |
| IOLMaster 700 vs. ANTERION | −5.660034 | 6.745601 | 9.755823 | 20974.86 | −15.41586 | 20974.86 | 0.999 |

*CI* = confidence intervals; *SE* = standard error

Appendix I Table 37. Node-splitting analysis of inconsistency in white-to-white

| Devices | Direct estimate (95% Cl) | | Indirect estimate (95% Cl) | | Overall (95% Cl) | | *P* value |
| --- | --- | --- | --- | --- | --- | --- | --- |
|  | Coefficient | SE | Coefficient | SE | Coefficient | SE |  |
| Galilei G6 vs. Lenstar | −1.60E−01 | 0.1905143 | 0.1202461 | 0.2066954 | −0.280246 | 0.2811026 | 0.319 |
| Galilei G6 vs. IOLMaster 700 | 6.00E−02 | 0.1939592 | −0.2202238 | 0.2034678 | 0.2802232 | 0.2811038 | 0.319 |
| IOLMaster vs. IOLMaster 500 | −0.2699995 | 0.2024979 | −0.0309459 | 0.0951364 | −0.2390536 | 0.2237328 | 0.285 |
| IOLMaster vs. Lenstar | −3.52E−02 | 0.0911932 | −0.0716104 | 0.1206853 | 0.036373 | 0.1512641 | 0.81 |
| IOLMaster vs. Aladdin | −3.90E−01 | 0.1905907 | −0.4212855 | 0.1588751 | 0.0312852 | 0.2481252 | 0.9 |
| IOLMaster vs. OA-2000 | −0.1745697 | 0.1430614 | −0.2602091 | 0.1147225 | 0.0856394 | 0.1833787 | 0.64 |
| IOLMaster 500 vs. AL-Scan | −5.20E−01 | 0.1922276 | −0.1309632 | 0.1082082 | −0.3890363 | 0.2205912 | 0.078 |
| IOLMaster 500 vs. Lenstar | 0.1490321 | 0.1105286 | −0.0456622 | 0.0841746 | 0.1946944 | 0.1389344 | 0.161 |
| IOLMaster 500 vs. IOLMaster 700 | −0.0128664 | 0.0906903 | −0.0210208 | 0.100731 | 0.0081544 | 0.1355455 | 0.952 |
| IOLMaster 500 vs. OA-2000 | −0.1833388 | 0.0904269 | −0.0952951 | 0.1232193 | −0.0880436 | 0.1528285 | 0.565 |
| IOLMaster 500 vs. Pentacam AXL | −2.39E−01 | 0.1372755 | −0.3471079 | 0.19079 | 0.1085353 | 0.2350416 | 0.644 |
| AL-Scan vs. Lenstar | 0.1399979 | 0.099087 | 0.495782 | 0.1475625 | −0.3557841 | 0.1777468 | 0.045 |
| AL-Scan vs. IOLMaster 700 | 0.3200006 | 0.1991598 | 0.1714854 | 0.1133738 | 0.1485153 | 0.2291686 | 0.517 |
| Lenstar vs. Aladdin | −0.3698395 | 0.1391804 | −0.3385534 | 0.2054136 | −0.0312861 | 0.2481249 | 0.9 |
| Lenstar vs. IOLMaster 700 | 0.0539854 | 0.1105426 | −0.1033572 | 0.0882352 | 0.1573426 | 0.1414448 | 0.266 |
| Lenstar vs. OA-2000 | −0.1250957 | 0.1370277 | −0.2049415 | 0.0971269 | 0.0798458 | 0.1679549 | 0.635 |
| Lenstar vs. Argos | −0.5599995 | 0.1581035 | 0.4738187 | 0.1565369 | −1.033818 | 0.2224872 | 0 |
| IOLMaster 700 vs. OA-2000 | −0.1400003 | 0.1978533 | −0.1350905 | 0.0951369 | −0.0049099 | 0.2195381 | 0.982 |
| IOLMaster 700 vs. Argos | 0.4499998 | 0.1463975 | -0.5838182 | 0.1675359 | 1.033818 | 0.2224872 | 0 |
| IOLMaster 700 vs. Pentacam AXL | −0.2757747 | 0.1363483 | −0.2253936 | 0.1939349 | −0.0503811 | 0.2371252 | 0.832 |
| IOLMaster 700 vs. ANTERION | −0.1199999 | 0.1999812 | 0.1380587 | 258.0584 | −0.2580586 | 258.0585 | 0.999 |

*CI* = confidence intervals; *SE* = standard error

Appendix I Table 38. Node-splitting analysis of inconsistency in lens thickness

| Devices | Direct estimate (95% Cl) | | Indirect estimate (95% Cl) | | Overall (95% Cl) | | *P* value |
| --- | --- | --- | --- | --- | --- | --- | --- |
|  | Coefficient | SE | Coefficient | SE | Coefficient | SE |  |
| Contact ultrasound vs. Lenstar | −2.08E−01 | 0.0458225 | −0.040639 | 18.93839 | −0.1669067 | 18.93845 | 0.993 |
| Galilei G6 vs. Lenstar | −1.10E−01 | 0.0853939 | 0.0712736 | 0.1055404 | −0.1812737 | 0.1357604 | 0.182 |
| Galilei G6 vs. IOLMaster 700 | 0.1181653 | 0.1023506 | −0.0631085 | 0.0894162 | 0.1812737 | 0.1357605 | 0.182 |
| Lenstar vs. Aladdin | 1.60E−01 | 0.0874935 | 0.070956 | 0.1070088 | 0.0890443 | 0.1382244 | 0.519 |
| Lenstar vs. IOLMaster 700 | 3.43E−02 | 0.0297923 | 0.114404 | 0.0526332 | −0.0800913 | 0.0598683 | 0.181 |
| Lenstar vs. OA-2000 | 0.1213432 | 0.0439411 | 0.1366092 | 0.0781725 | −0.0152659 | 0.0896537 | 0.865 |
| Lenstar vs. Argos | 2.00E−01 | 0.0839118 | 0.0960385 | 0.0638907 | 0.1039613 | 0.1054666 | 0.324 |
| Aladdin vs. IOLMaster 700 | −0.02 | 0.103505 | −0.1090445 | 0.0916115 | 0.0890445 | 0.1382243 | 0.519 |
| IOLMaster 700 vs. OA-2000 | 0.0800002 | 0.0726843 | 0.0647345 | 0.052486 | 0.0152657 | 0.0896538 | 0.865 |
| IOLMaster 700 vs. Argos | 0.0488704 | 0.0579 | 0.1528317 | 0.0882355 | −0.1039613 | 0.1054665 | 0.324 |

*CI* = confidence intervals; *SE* = standard error


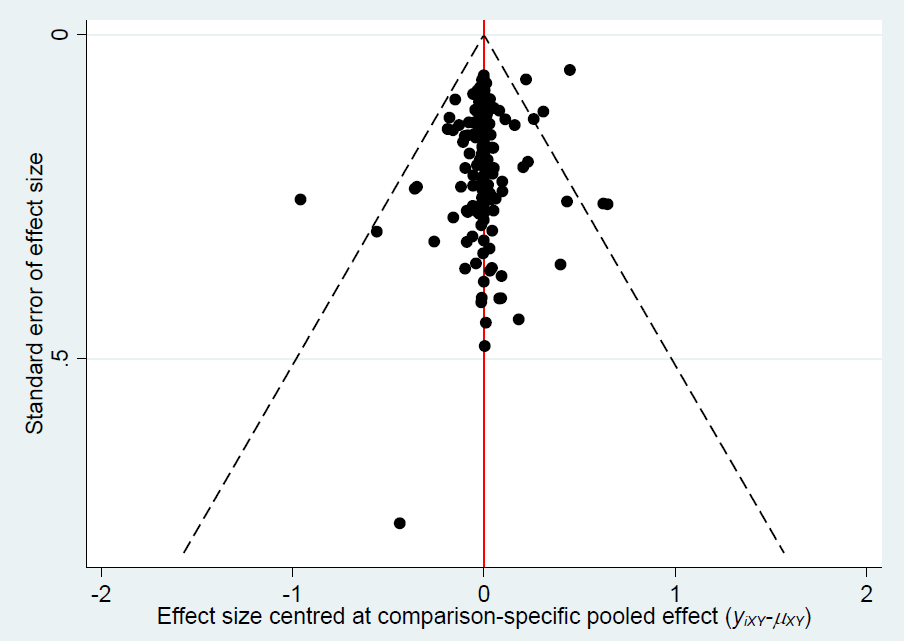


Appendix I Figure 1. The funnel plot in axial length


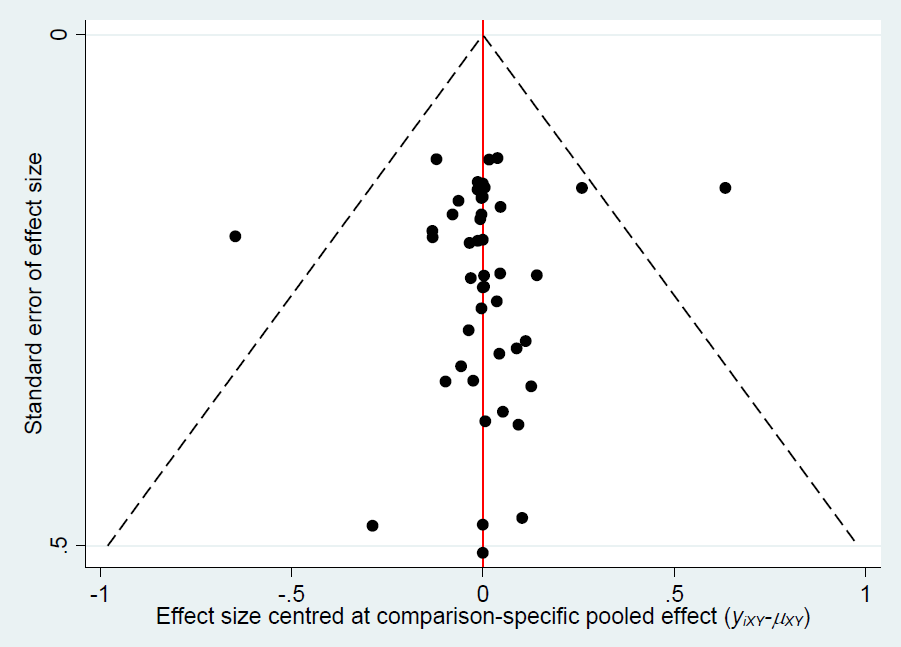


Appendix I Figure 2. The funnel plot in keratometry in the flattest meridian


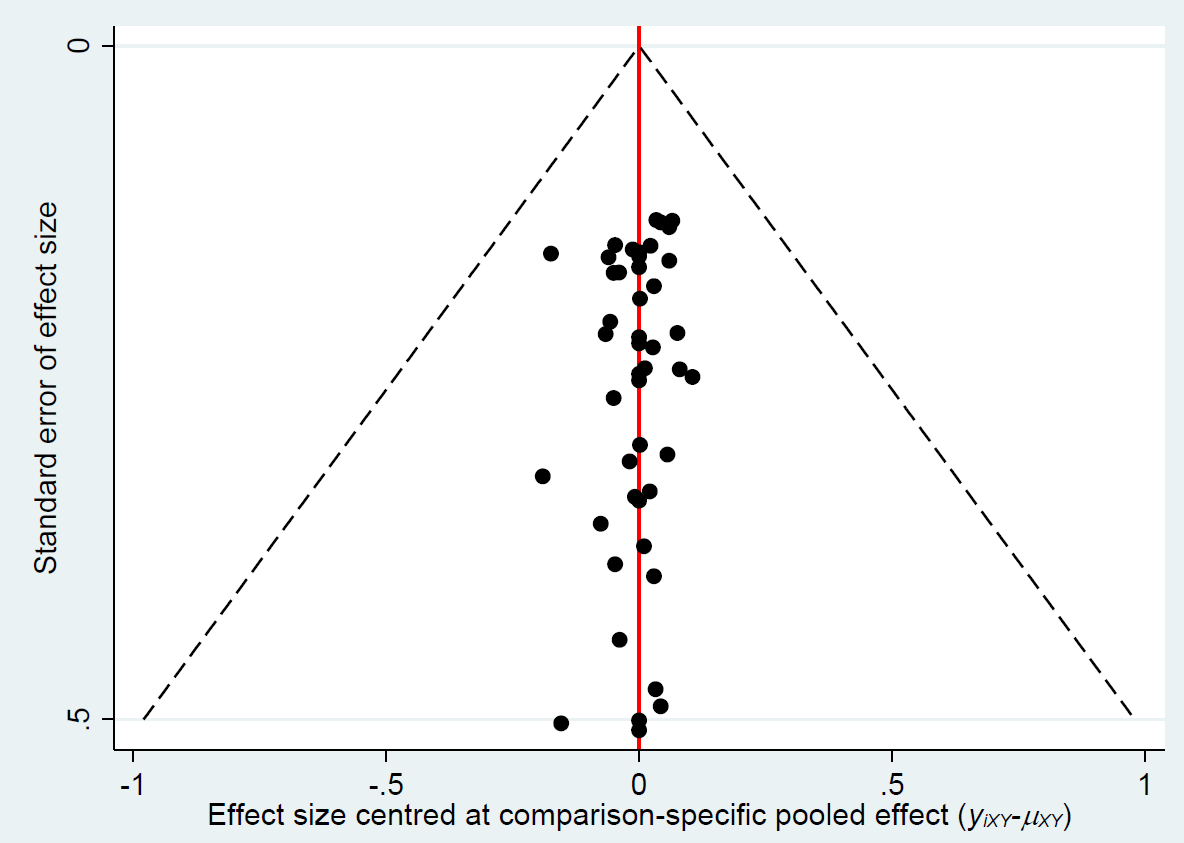


Appendix I Figure 3. The funnel plot in keratometry in the steepest meridian


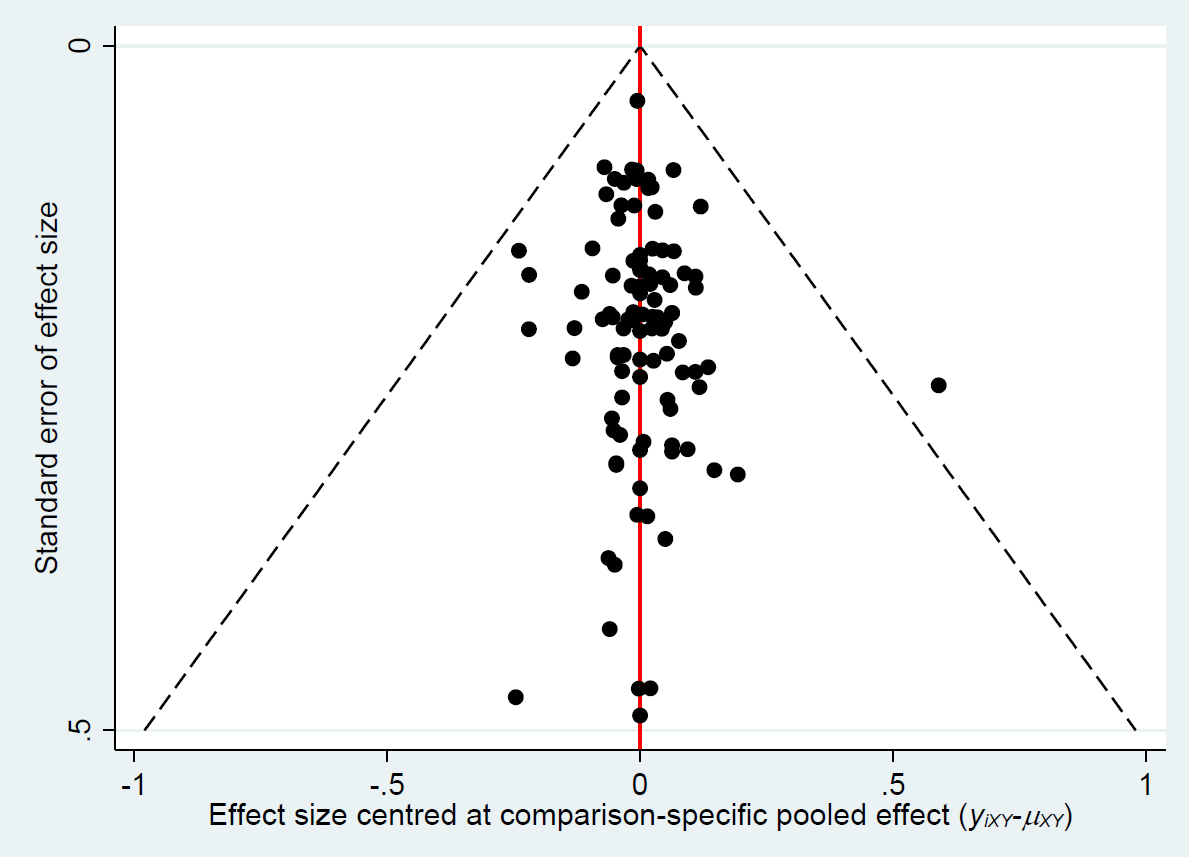


Appendix I Figure 4. The funnel plot in mean keratometry


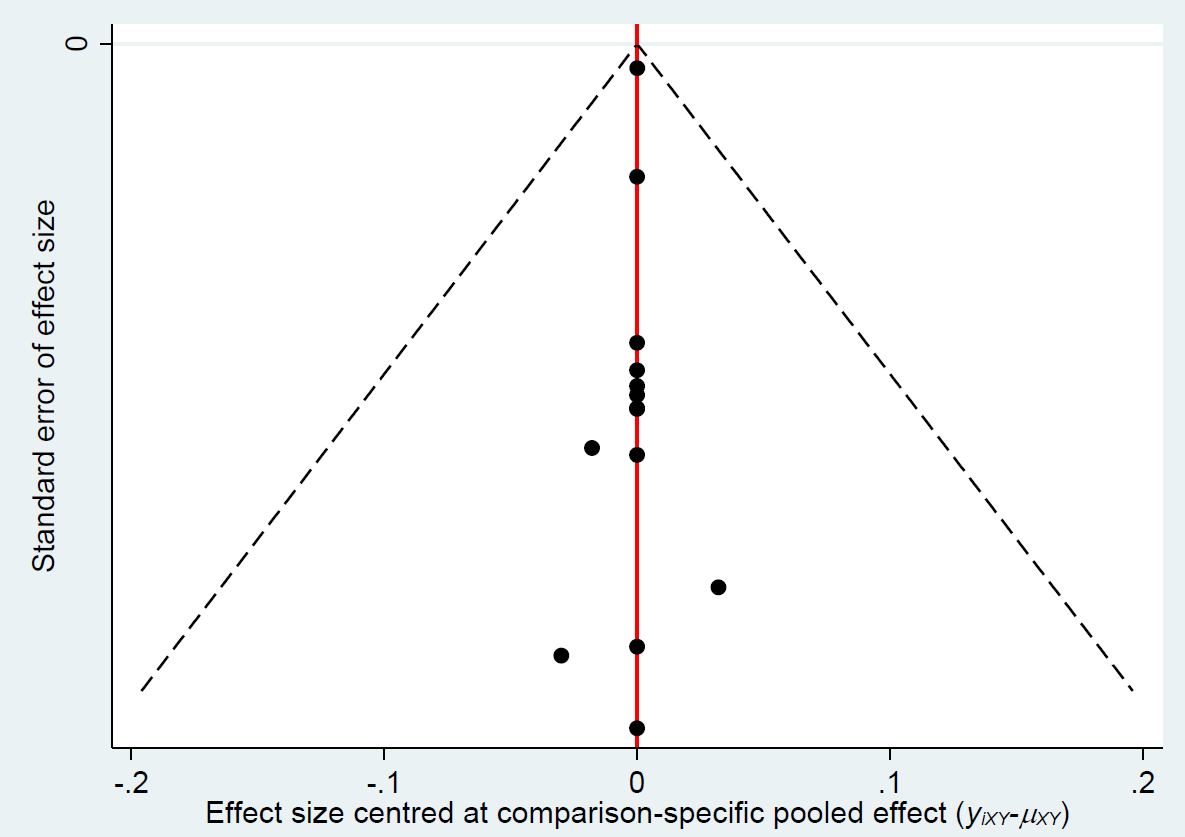


Appendix I Figure 5. The funnel plot in J_0_


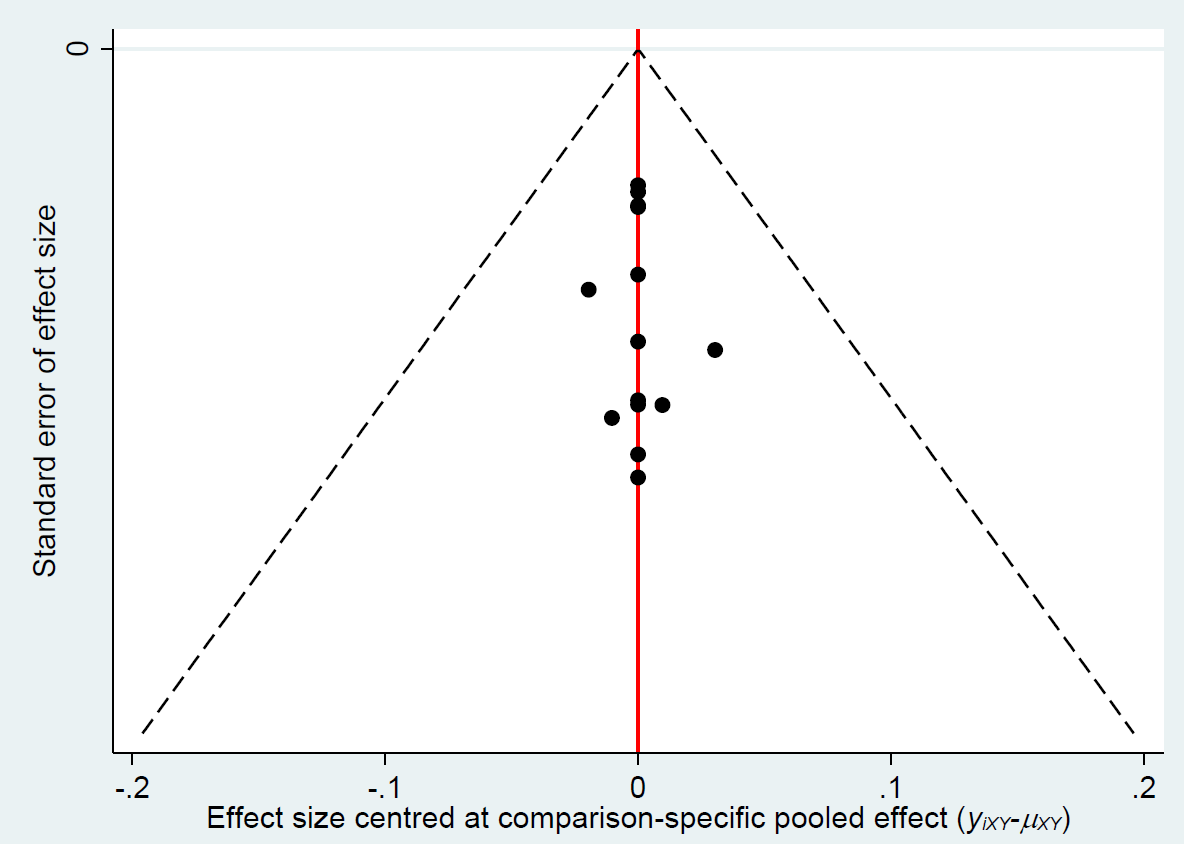


Appendix I Figure 6. The funnel plot in J_45_


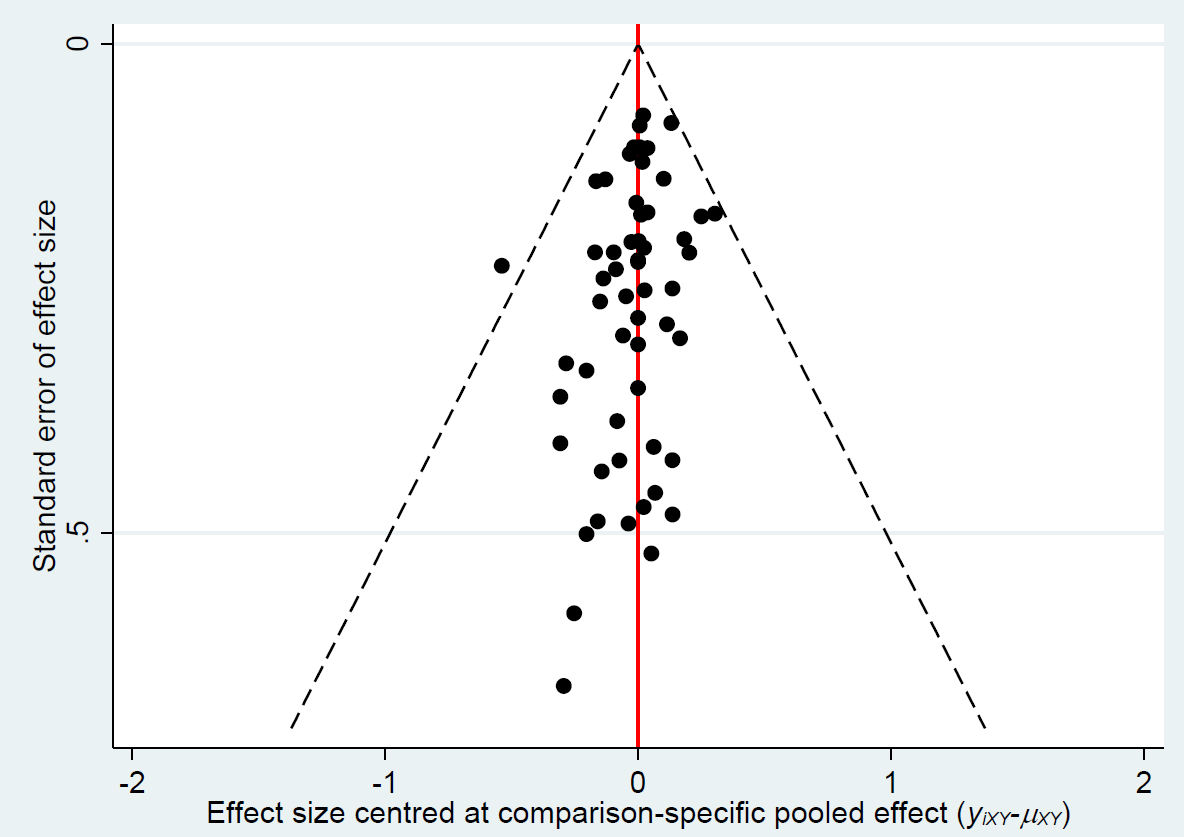


Appendix I Figure 7. The funnel plot in astigmatism


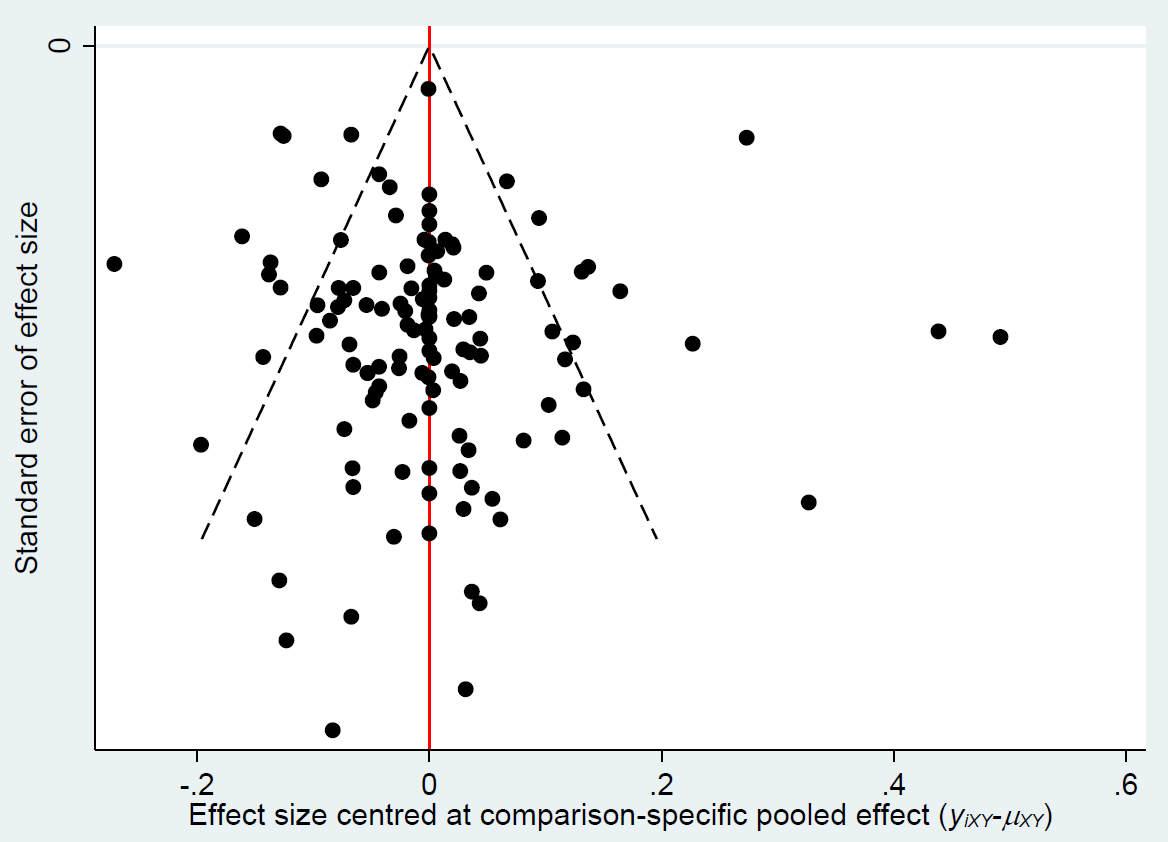


Appendix I Figure 8. The funnel plot in anterior chamber depth


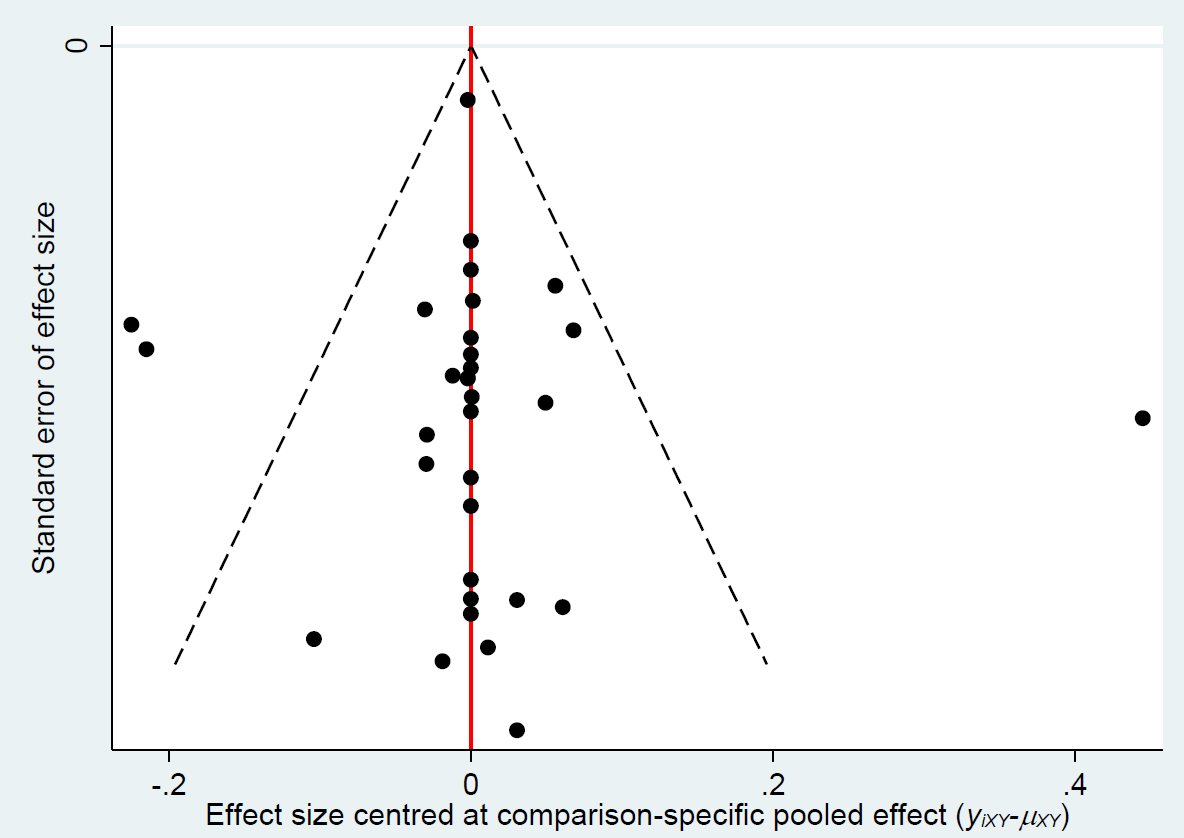


Appendix I Figure 9. The funnel plot in aqueous depth


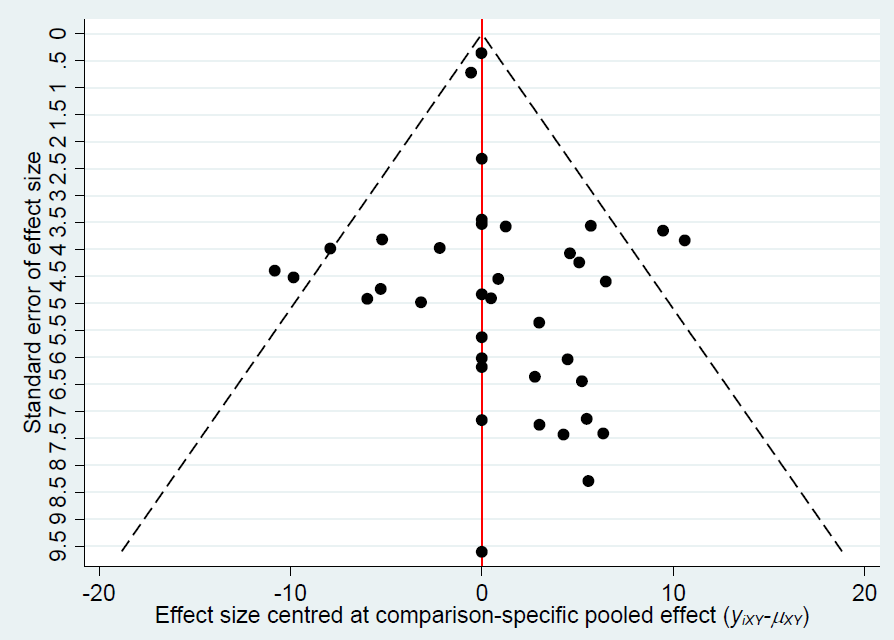


Appendix I Figure 10. The funnel plot in central corneal thickness


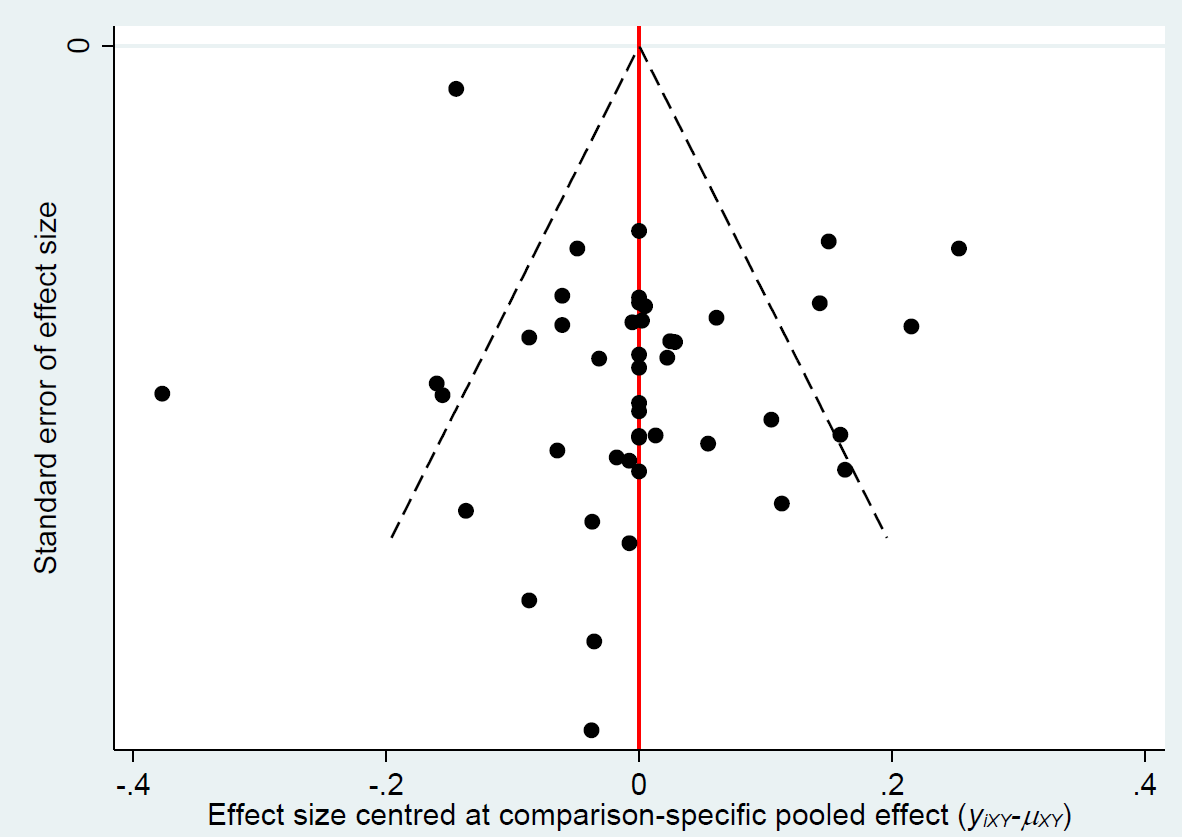


Appendix I Figure 11. The funnel plot in corneal diameter


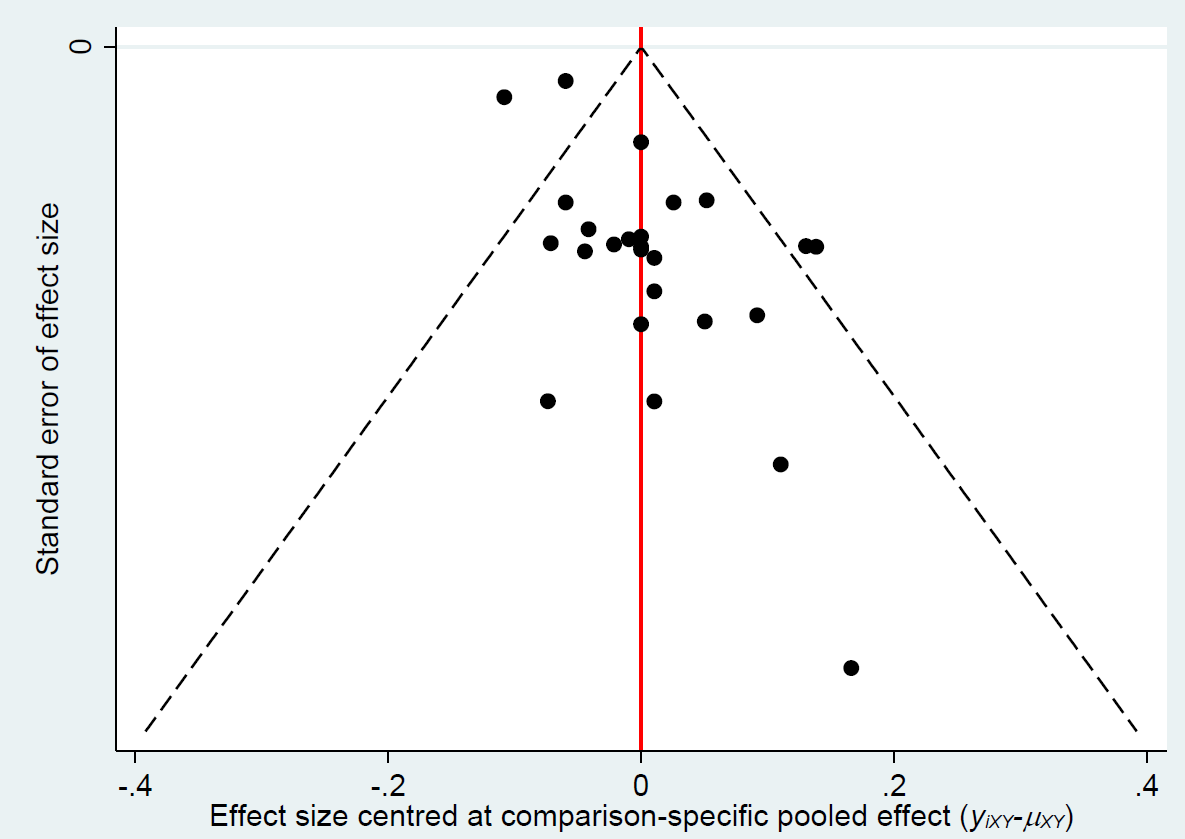


Appendix I Figure 12. The funnel plot in lens thickness
