# Supplementary material for: Comprehensive comparisons of ocular biometry: A network-based big data analysis
Source: Eye Vis (Lond). 2022 Dec 10;10:1. doi: 10.1186/s40662-022-00320-3 (PMC9808957; doi:10.1186/s40662-022-00320-3)

**Supplemental Figure 1.** Formula rank in axial length (AL), anterior chamber depth (ACD), and lens thickness (LT). **a** Ranking probability results in the AL; **b** Ranking probability results in the ACD; **c** Ranking probability results in LT.

**
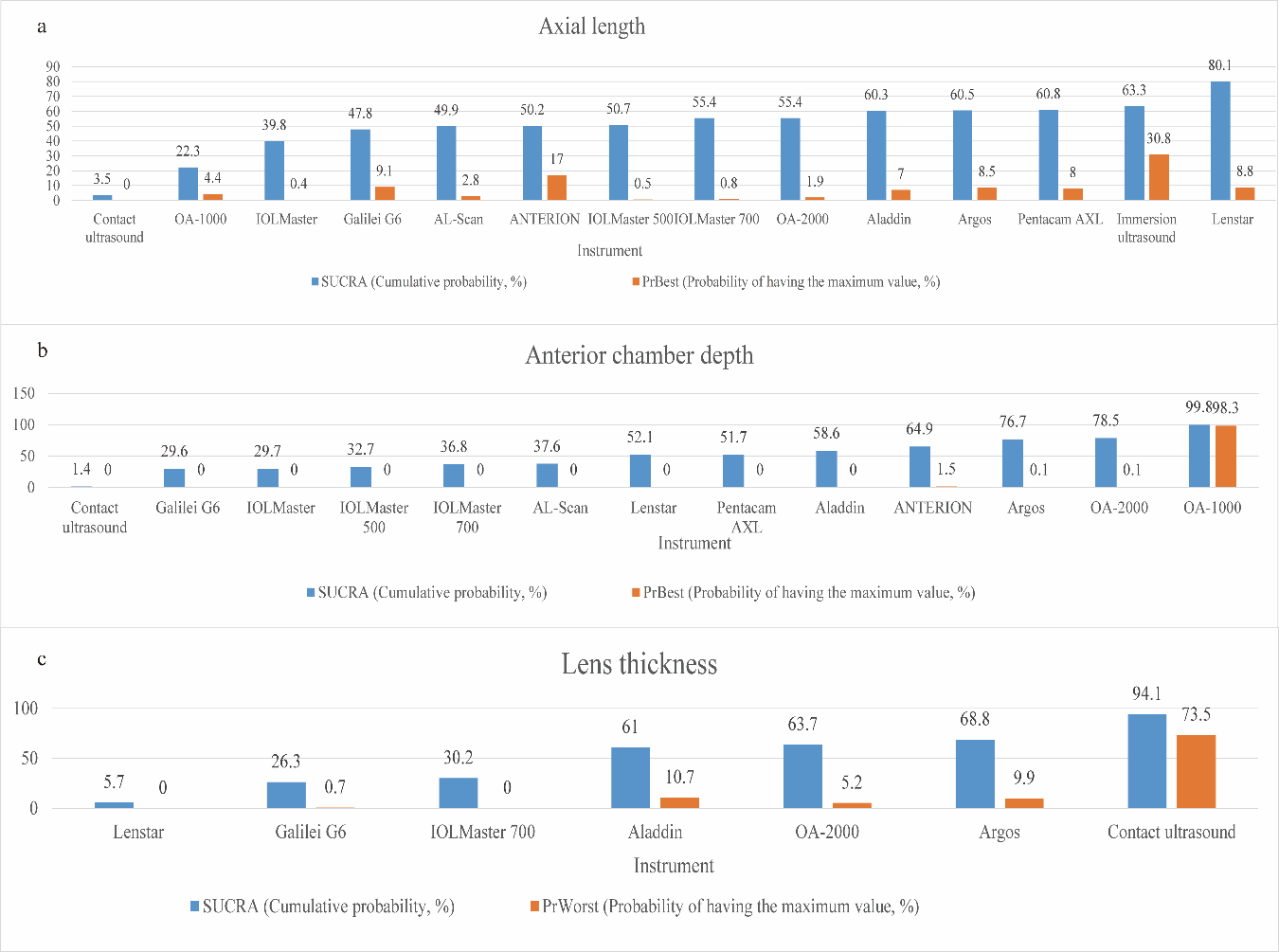
**

**Supplemental Figure 2.** Formula rank in keratometry and corneal diameter (CD). **a** Ranking probability results in the keratometry in the flattest meridian (Kf); **b** Ranking probability results in the keratometry in the steepest meridian (Ks); **c** Ranking probability results in the mean keratometry (Km); **d** Ranking probability results in CD.

**
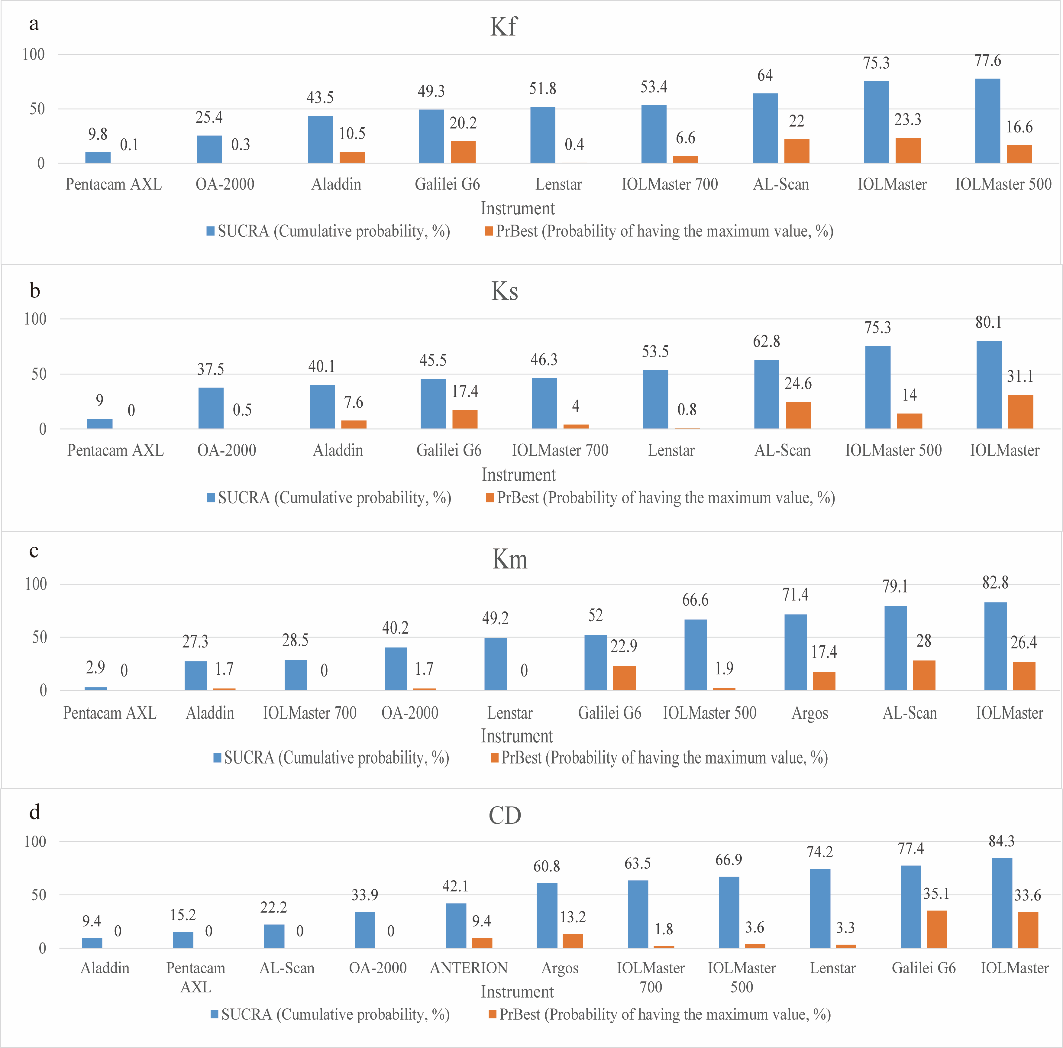
**

**Supplemental Figure 3.** Formula rank in astigmatism. **a** Ranking probability results in the J_0_; **b** Ranking probability results in the J_45_; **c** Ranking probability results in astigmatism. J_0_, anterior corneal power vectors for the cardinal (axes at 90 degrees and 180 degrees) meridians; J_45_, anterior corneal power vectors for the oblique (axes at 45 degrees and 135 degrees) meridians.


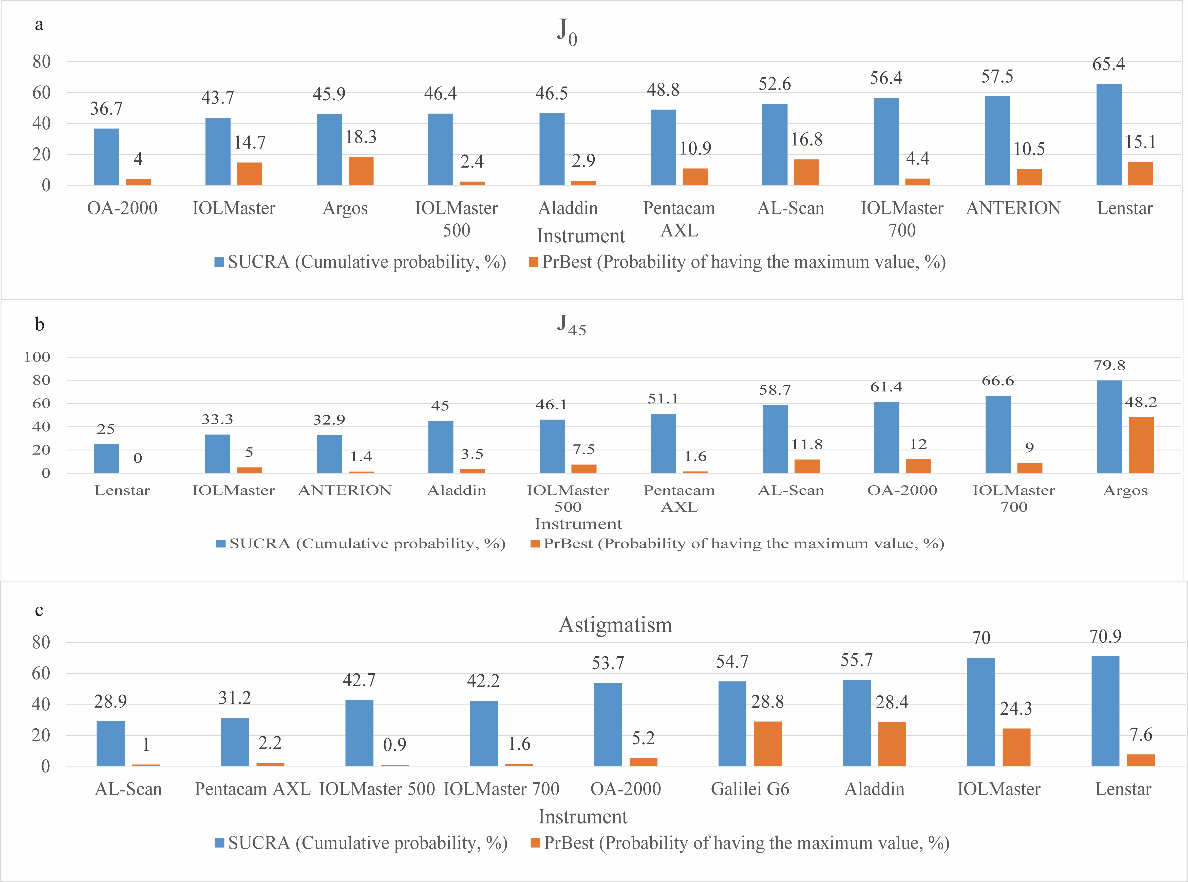

Supplement: Supplementary file 2 — Additional file 2. Supplemental Figure 1. Formula rank in axial length (AL), anterior chamber depth (ACD), and lens thickness (LT). a Ranking probability results in the AL; b Ranking probability results in the ACD; c Ranking probability results in LT. Supplemental Figure 2. Formula rank in keratometry and corneal diameter (CD). a Ranking probability results in the keratometry in the flattest meridian (Kf); b Ranking probability results in the keratometry in the steepest meridian (Ks); c Ranking probability results in the mean keratometry (Km); d Ranking probability results in CD. Supplemental Figure 3. Formula rank in astigmatism. a Ranking probability results in the J0; b Ranking probability results in the J45; c Ranking probability results in astigmatism. J0, anterior corneal power vectors for the cardinal (axes at 90 degrees and 180 degrees) meridians; J45, anterior corneal power vectors for the oblique (axes at 45 degrees and 135 degrees) meridians. [file 40662_2022_320_MOESM2_ESM.docx]
