# Supplementary material for: Comprehensive comparisons of ocular biometry: A network-based big data analysis
Source: Eye Vis (Lond). 2022 Dec 10;10:1. doi: 10.1186/s40662-022-00320-3 (PMC9808957; doi:10.1186/s40662-022-00320-3)
Supplement: Supplementary file 3 — Additional file 3. Supplementary tables. [file 40662_2022_320_MOESM3_ESM.docx]

Appendix II Table 1: Node-splitting analysis of inconsistency in axial length for cataract people.

| Devices | Direct estimate (95% Cl) | | Indirect estimate (95% Cl) | | Overall (95% Cl) | | *P* value |
| --- | --- | --- | --- | --- | --- | --- | --- |
|  | Coefficient | SE | Coefficient | SE | Coefficient | SE |  |
| Contact ultrasound vs. IOLMaster | 0.2567541 | 0.039076 | 0.1458129 | 0.0740138 | 0.1109413 | 0.0836953 | 0.185 |
| Contact ultrasound vs. IOLMaster 500 | 0.137007 | 0.0703417 | 0.1873234 | 0.0592008 | −0.0503163 | 0.0919693 | 0.584 |
| Contact ultrasound vs. AL-Scan | 0.0480083 | 0.104776 | 0.1907234 | 0.0679988 | −0.1427151 | 0.1249073 | 0.253 |
| Contact ultrasound vs. OA-1000 | 0.1799984 | 0.1320607 | −0.2232306 | 0.240673 | 0.403229 | 0.2702088 | 0.136 |
| Contact ultrasound vs. Lenstar | 0.2399998 | 0.19296 | 0.2041217 | 0.0506072 | 0.0358781 | 0.1994859 | 0.857 |
| Contact ultrasound vs. Aladdin | 0.1999989 | 0.1627467 | 0.2334945 | 0.0583014 | −0.0334956 | 0.1728743 | 0.846 |
| Contact ultrasound vs. IOLMaster 700 | 0.0699997 | 0.2017768 | 0.17782 | 0.0590525 | −0.1078203 | 0.2102405 | 0.608 |
| Contact ultrasound vs. Argos | 0.0888311 | 0.2756229 | 0.1656082 | 0.0695711 | −0.0767772 | 0.2842106 | 0.787 |
| Immersion ultrasound vs. IOLMaster | 0.0099401 | 0.1645988 | −0.0554589 | 0.2457836 | 0.0653989 | 0.295808 | 0.825 |
| Immersion ultrasound vs. Lenstar | −0.0799999 | 0.2413895 | −0.0146008 | 0.1709781 | −0.0653992 | 0.295808 | 0.825 |
| Galilei G6 vs. IOLMaster 500 | −0.0200005 | 0.1673456 | 0.0018609 | 0.1184962 | −0.0218613 | 0.205051 | 0.915 |
| Galilei G6 vs. Lenstar | 0.039999 | 0.1195288 | 0.0256831 | 0.1520955 | 0.0143159 | 0.193443 | 0.941 |
| Galilei G6 vs. IOLMaster 700 | 0.0100002 | 0.3026707 | −0.0041495 | 0.1067591 | 0.0141497 | 0.3209471 | 0.965 |
| IOLMaster vs. IOLMaster 500 | −0.0224308 | 0.2200176 | −0.0681338 | 0.0473185 | 0.045703 | 0.2250465 | 0.839 |
| IOLMaster vs. OA-1000 | −0.2399998 | 0.1346999 | 0.1632298 | 0.2362443 | −0.4032295 | 0.2702088 | 0.136 |
| IOLMaster vs. Lenstar | 0.0246147 | 0.068706 | −0.0664829 | 0.0612474 | 0.0910976 | 0.0920831 | 0.323 |
| IOLMaster vs. Aladdin | 0.0190706 | 0.0574647 | −0.0618739 | 0.0950933 | 0.0809445 | 0.1120199 | 0.47 |
| IOLMaster vs. OA-2000 | −0.0180451 | 0.0622545 | −0.0495901 | 0.0757971 | 0.031545 | 0.0990323 | 0.75 |
| IOLMaster vs. Argos | 0.0220936 | 0.2737425 | −0.0774448 | 0.0689114 | 0.0995385 | 0.2821678 | 0.724 |
| IOLMaster 500 vs. AL-Scan | −0.0069126 | 0.0611644 | −0.0368184 | 0.0795135 | 0.0299058 | 0.1003169 | 0.766 |
| IOLMaster 500 vs. Lenstar | 0.117183 | 0.090333 | 0.0169566 | 0.0491974 | 0.1002265 | 0.1030292 | 0.331 |
| IOLMaster 500 vs. Aladdin | 0.0100002 | 0.1816636 | 0.0691449 | 0.0605723 | −0.0591446 | 0.1914959 | 0.757 |
| IOLMaster 500 vs. IOLMaster 700 | −0.0450175 | 0.0639118 | 0.054941 | 0.0666682 | −0.0999585 | 0.0924258 | 0.279 |
| IOLMaster 500 vs. OA-2000 | 0.0303676 | 0.0869535 | 0.0377921 | 0.0617517 | −0.0074245 | 0.1066544 | 0.945 |
| IOLMaster 500 vs. Argos | −0.0000865 | 0.0716052 | −0.0154551 | 0.0984934 | 0.0153686 | 0.122659 | 0.9 |
| IOLMaster 500 vs. Pentacam AXL | −0.0070127 | 0.0879616 | 0.0047601 | 0.0814251 | −0.0117728 | 0.1195581 | 0.922 |
| AL-Scan vs. Lenstar | 0.0014132 | 0.0965728 | 0.0854897 | 0.0673049 | −0.0840765 | 0.1177126 | 0.475 |
| AL-Scan vs. IOLMaster 700 | 0.0100002 | 0.3177081 | 0.0213175 | 0.0632509 | −0.0113173 | 0.3239431 | 0.972 |
| Lenstar vs. Aladdin | −0.0100002 | 0.173085 | 0.027473 | 0.0616499 | −0.0374732 | 0.1837366 | 0.838 |
| Lenstar vs. IOLMaster 700 | 0.0074985 | 0.0813523 | −0.0627426 | 0.0616535 | 0.070241 | 0.1020771 | 0.491 |
| Lenstar vs. OA-2000 | 0.0046308 | 0.107803 | −0.0075439 | 0.0599128 | 0.0121747 | 0.1233487 | 0.921 |
| Lenstar vs. Argos | −0.0489477 | 0.1302298 | −0.0442907 | 0.0730294 | −0.004657 | 0.1494897 | 0.975 |
| Lenstar vs. Pentacam AXL | −0.0284707 | 0.0703643 | −0.0612327 | 0.0915141 | 0.0327619 | 0.1154382 | 0.777 |
| Aladdin vs. IOLMaster 700 | −3.51E−10 | 0.3813898 | −6.22E−02 | 0.0655845 | 0.0621563 | 0.3869877 | 0.872 |
| Aladdin vs. OA-2000 | −0.0195745 | 0.0627123 | −0.0464651 | 0.0934372 | 0.0268907 | 0.1125127 | 0.811 |
| IOLMaster 700 vs. OA-2000 | −0.0200005 | 0.1114121 | 0.0508097 | 0.0659234 | −0.0708102 | 0.1294548 | 0.584 |
| IOLMaster 700 vs. Argos | −2.24E−02 | 0.1112401 | −0.0015646 | 0.076841 | −0.0208224 | 0.1351995 | 0.878 |
| IOLMaster 700 vs. Pentacam AXL | −0.0403111 | 0.1158224 | 0.0132028 | 0.078283 | −0.0535139 | 0.1394358 | 0.701 |
| IOLMaster 700 vs. ANTERION | −0.0099983 | 0.0846101 | −0.1739153 | 828.5529 | 0.163917 | 828.5529 | 1 |
| OA-2000 vs. Argos | −0.0303284 | 0.1347619 | −0.044294 | 0.0787319 | 0.0139656 | 0.156132 | 0.929 |

*CI* = confidence intervals; *SE* = standard error

Appendix II Table 2: Node-splitting analysis of inconsistency in axial length for healthy people.

| Devices | Direct estimate (95% Cl) | | Indirect estimate (95% Cl) | | Overall (95% Cl) | | *P* value |
| --- | --- | --- | --- | --- | --- | --- | --- |
|  | Coefficient | SE | Coefficient | SE | Coefficient | SE |  |
| Contact ultrasound vs. IOLMaster | 0.080765 | 0.0640848 | 0.2065185 | 0.2181011 | −0.1257535 | 0.2275085 | 0.58 |
| Contact ultrasound vs. AL-Scan | 0.2999992 | 0.1395961 | 0.1496667 | 0.2029756 | 0.1503326 | 0.2463456 | 0.542 |
| Contact ultrasound vs. Lenstar | 0.2327521 | 0.1180432 | 0.0914665 | 0.1120199 | 0.1412856 | 0.1625102 | 0.385 |
| IOLMaster vs. Lenstar | 0.0166049 | 0.0878575 | 0.1808662 | 0.1309161 | −0.1642613 | 0.1575244 | 0.297 |
| IOLMaster vs. OA-2000 | 0.0264816 | 0.1296226 | 0.0749013 | 0.147587 | −0.0484197 | 0.196428 | 0.805 |
| IOLMaster 500 vs. Lenstar | 0.0184602 | 0.0775339 | 0.0411875 | 0.1524883 | −0.0227272 | 0.1710678 | 0.894 |
| IOLMaster 500 vs. Aladdin | −2.63E−10 | 0.1937091 | 0.0353247 | 0.2106022 | −0.0353247 | 0.2861407 | 0.902 |
| IOLMaster 500 vs. IOLMaster 700 | 0.0400009 | 0.2531139 | −0.0119223 | 0.1587962 | 0.0519232 | 0.2988025 | 0.862 |
| IOLMaster 500 vs. OA-2000 | 0.0200005 | 0.1640527 | −0.007032 | 0.1274166 | 0.0270324 | 0.2077216 | 0.896 |
| AL-Scan vs. Lenstar | −0.0100002 | 0.1834004 | −0.1603328 | 0.1644702 | 0.1503325 | 0.2463456 | 0.542 |
| Lenstar vs. Aladdin | 0.0099983 | 0.1981409 | −0.0253265 | 0.2064381 | 0.0353248 | 0.2861407 | 0.902 |
| Lenstar vs. OA-2000 | 0.0100002 | 0.2146221 | −0.0275271 | 0.1075973 | 0.0375274 | 0.2400829 | 0.876 |
| Lenstar vs. Pentacam AXL | −0.1299992 | 0.3611406 | −0.0334643 | 0.1713212 | −0.0965349 | 0.3997167 | 0.809 |
| IOLMaster 700 vs. OA-2000 | 1.85E−10 | 0.1323793 | 0.0016235 | 0.2327364 | −0.0016235 | 0.2677508 | 0.995 |
| IOLMaster 700 vs. Pentacam AXL | −0.0255481 | 0.093336 | −0.1220832 | 0.3886666 | 0.0965352 | 0.3997165 | 0.809 |
| Argos vs. Pentacam AXL | −0.0200005 | 0.1685414 | 0.2480949 | 933.5292 | −0.2680954 | 933.5292 | 1 |

*CI* = confidence intervals; *SE* = standard error

Appendix II Table 3: Node-splitting analysis of inconsistency in keratometry in the flattest meridian for cataract people.

| Devices | Direct estimate (95% Cl) | | Indirect estimate (95% Cl) | | Overall (95% Cl) | | *P* value |
| --- | --- | --- | --- | --- | --- | --- | --- |
|  | Coefficient | SE | Coefficient | SE | Coefficient | SE |  |
| Galilei G6 vs. IOLMaster 500 | 0.0300026 | 0.2472577 | 0.0855981 | 0.2652084 | −0.0555955 | 0.3625905 | 0.878 |
| Galilei G6 vs. IOLMaster 700 | 0.0382986 | 0.2541869 | −0.0298275 | 0.2652531 | 0.0681261 | 0.366088 | 0.852 |
| Galilei G6 vs. Pentacam AXL | −0.32944 | 0.5070181 | −0.1222674 | 0.2214017 | −0.2071727 | 0.5531976 | 0.708 |
| IOLMaster vs. Lenstar | −0.068923 | 0.0927054 | 0.4612689 | 0.4590286 | −0.5301919 | 0.4682965 | 0.258 |
| IOLMaster vs. OA-2000 | 0.2909726 | 0.1494464 | 0.1220944 | 0.2119321 | 0.1688782 | 0.2622492 | 0.52 |
| IOLMaster 500 vs. AL-Scan | 0.0599976 | 0.1456186 | 0.1181948 | 0.2710542 | −0.0581973 | 0.3076933 | 0.85 |
| IOLMaster 500 vs. Lenstar | −0.0499992 | 0.2016591 | −0.1025321 | 0.1135812 | 0.0525328 | 0.2314456 | 0.82 |
| IOLMaster 500 vs. Aladdin | −0.0499992 | 0.3122019 | −0.0712488 | 0.2550224 | 0.0212496 | 0.4031208 | 0.958 |
| IOLMaster 500 vs. IOLMaster 700 | −0.0463036 | 0.1433179 | −0.0547593 | 0.1565597 | 0.0084557 | 0.2122521 | 0.968 |
| IOLMaster 500 vs. OA-2000 | 0.0399971 | 0.3069595 | 0.2320802 | 0.1563246 | −0.1920831 | 0.3444728 | 0.577 |
| IOLMaster 500 vs. Pentacam AXL | −0.201138 | 0.1408707 | −0.2281294 | 0.1814694 | 0.0269914 | 0.2297296 | 0.906 |
| AL-Scan vs. Lenstar | −0.201256 | 0.2497533 | −0.1430587 | 0.1797177 | −0.0581973 | 0.3076933 | 0.85 |
| Lenstar vs. Aladdin | 0.0200005 | 0.2335986 | 0.0412501 | 0.3285393 | −0.0212496 | 0.4031208 | 0.958 |
| Lenstar vs. IOLMaster 700 | 0.0251068 | 0.1367544 | 0.06261 | 0.1711393 | −0.0375033 | 0.2190672 | 0.864 |
| Lenstar vs. OA-2000 | 0.3098149 | 0.1267005 | 0.1898153 | 0.2393344 | 0.1199996 | 0.2755139 | 0.663 |
| Lenstar vs. Pentacam AXL | −0.1300011 | 0.1588655 | −0.1113283 | 0.1720369 | −0.0186727 | 0.2341686 | 0.936 |
| IOLMaster 700 vs. Pentacam AXL | −0.0649159 | 0.4785492 | −0.1695296 | 0.1422568 | 0.1046137 | 0.4987181 | 0.834 |

*CI* = confidence intervals; *SE* = standard error

Appendix II Table 4: Node-splitting analysis of inconsistency in keratometry in the flattest meridian for healthy people.

| Devices | Direct estimate (95% Cl) | | Indirect estimate (95% Cl) | | Overall (95% Cl) | | *P* value |
| --- | --- | --- | --- | --- | --- | --- | --- |
|  | Coefficient | SE | Coefficient | SE | Coefficient | SE |  |
| IOLMaster vs. Lenstar | −0.0294777 | 0.1868411 | −0.9392632 | 0.2455333 | 0.9097854 | 0.3085388 | 0.003 |
| IOLMaster vs. OA-2000 | −1 | 0.1973247 | −0.0902125 | 0.2371902 | −0.9097875 | 0.3085389 | 0.003 |
| IOLMaster 500 vs. Lenstar | −0.0450397 | 0.1897992 | 0.0853812 | 0.2966449 | −0.1304208 | 0.3521674 | 0.711 |
| IOLMaster 500 vs. Aladdin | −0.1300011 | 0.3647503 | −0.0593647 | 0.3454546 | −0.0706364 | 0.502376 | 0.888 |
| IOLMaster 500 vs. OA-2000 | −0.1800005 | 0.2913994 | −0.4117759 | 0.2914688 | 0.2317754 | 0.41215 | 0.574 |
| Lenstar vs. Aladdin | −0.0599976 | 0.3015259 | −0.1306335 | 0.4018257 | 0.0706359 | 0.5023761 | 0.888 |
| Lenstar vs. OA-2000 | 3.52E−10 | 0.2410315 | −0.4832906 | 0.2107799 | 0.4832906 | 0.3201942 | 0.131 |
| IOLMaster 700 vs. OA-2000 | −0.0499993 | 0.2678751 | −1.171541 | 946.7231 | 1.121541 | 946.7231 | 0.999 |

*CI* = confidence intervals; *SE* = standard error

Appendix II Table 5: Node-splitting analysis of inconsistency in keratometry in the steepest meridian for cataract people.

| Devices | Direct estimate (95% Cl) | | Indirect estimate (95% Cl) | | Overall (95% Cl) | | *P* value |
| --- | --- | --- | --- | --- | --- | --- | --- |
|  | Coefficient | SE | Coefficient | SE | Coefficient | SE |  |
| Galilei G6 vs. IOLMaster 500 | 0.0600014 | 0.2434809 | 0.1379794 | 0.2692773 | −0.077978 | 0.3630334 | 0.83 |
| Galilei G6 vs. IOLMaster 700 | 0.0230201 | 0.2597692 | 0.0054842 | 0.264197 | 0.0175359 | 0.3702061 | 0.962 |
| Galilei G6 vs. Pentacam AXL | −0.0688996 | 0.5080715 | −0.1926204 | 0.2208564 | 0.1237208 | 0.5539062 | 0.823 |
| IOLMaster vs. Lenstar | −0.0520812 | 0.091849 | −0.5069066 | 0.4854499 | 0.4548254 | 0.4947063 | 0.358 |
| IOLMaster vs. OA-2000 | −0.2341763 | 0.1511133 | −0.1283024 | 0.219004 | −0.1058739 | 0.2674607 | 0.692 |
| IOLMaster 500 vs. AL-Scan | −0.0499992 | 0.1531946 | 0.0576252 | 0.3215464 | −0.1076244 | 0.356175 | 0.763 |
| IOLMaster 500 vs. Lenstar | −0.1199989 | 0.204875 | −0.0637332 | 0.1197293 | −0.0562657 | 0.2372949 | 0.813 |
| IOLMaster 500 vs. Aladdin | −0.1899986 | 0.3085558 | −0.0913383 | 0.2704713 | −0.0986603 | 0.4103187 | 0.81 |
| IOLMaster 500 vs. IOLMaster 700 | −0.076192 | 0.147705 | −0.0860746 | 0.1620471 | 0.0098826 | 0.2192625 | 0.964 |
| IOLMaster 500 vs. OA-2000 | −0.1199989 | 0.354683 | −0.2281565 | 0.1617231 | 0.1081575 | 0.3898132 | 0.781 |
| IOLMaster 500 vs. Pentacam AXL | −0.2504796 | 0.1378482 | −0.3008338 | 0.1883048 | 0.0503542 | 0.2333684 | 0.829 |
| AL-Scan vs. Lenstar | −0.1256893 | 0.3026758 | −0.0180648 | 0.1877445 | −0.1076245 | 0.3561751 | 0.763 |
| Lenstar vs. Aladdin | −0.0200005 | 0.2483709 | −0.1186608 | 0.3266088 | 0.0986603 | 0.4103187 | 0.81 |
| Lenstar vs. IOLMaster 700 | −7.62E−12 | 0.1418331 | −0.0067332 | 0.1777465 | 0.0067332 | 0.2273993 | 0.976 |
| Lenstar vs. OA-2000 | −0.1511598 | 0.1306402 | −0.0596067 | 0.2548486 | −0.0915531 | 0.2897905 | 0.752 |
| Lenstar vs. Pentacam AXL | −0.2399979 | 0.164319 | −0.1337492 | 0.1742637 | −0.1062487 | 0.2395174 | 0.657 |
| IOLMaster 700 vs. Pentacam AXL | 0.0602426 | 0.5006918 | −0.2079912 | 0.1446381 | 0.2682338 | 0.5210706 | 0.607 |

*CI* = confidence intervals; *SE* = standard error

Appendix II Table 6 = Node-splitting analysis of inconsistency in the steepest meridian for healthy people.

| Devices | Direct estimate (95% Cl) | | Indirect estimate (95% Cl) | | Overall (95% Cl) | | *P* value |
| --- | --- | --- | --- | --- | --- | --- | --- |
|  | Coefficient | SE | Coefficient | SE | Coefficient | SE |  |
| IOLMaster vs. Lenstar | −0.1661579 | 0.202366 | −0.1870453 | 0.2742918 | 0.0208874 | 0.3408636 | 0.951 |
| IOLMaster vs. OA-2000 | −0.1899986 | 0.2239068 | −0.1691106 | 0.2570094 | −0.0208881 | 0.3408637 | 0.951 |
| IOLMaster 500 vs. Lenstar | −0.0751366 | 0.1047992 | −0.1201879 | 0.2262272 | 0.0450513 | 0.2493224 | 0.857 |
| IOLMaster 500 vs. Aladdin | −0.1500015 | 0.3307405 | −0.1002146 | 0.237493 | −0.049787 | 0.4071759 | 0.903 |
| IOLMaster 500 vs. OA-2000 | −0.1100006 | 0.2140022 | −0.0733356 | 0.201457 | −0.0366651 | 0.2939079 | 0.901 |
| Lenstar vs. Aladdin | −0.0200005 | 0.2163444 | −0.0697873 | 0.3449454 | 0.0497869 | 0.4071759 | 0.903 |
| Lenstar vs. OA-2000 | 2.00E−02 | 0.2132055 | −0.0284333 | 0.1862834 | 0.0484337 | 0.283122 | 0.864 |
| IOLMaster 700 vs. OA-2000 | 0.0099983 | 0.2208655 | −0.3628059 | 1026.915 | 0.3728042 | 1026.915 | 1 |

*CI* = confidence intervals; *SE* = standard error

Appendix II Table 7: Node-splitting analysis of inconsistency in mean keratometry for cataract people.

| Devices | Direct estimate (95% Cl) | | Indirect estimate (95% Cl) | | Overall (95% Cl) | | *P* value |
| --- | --- | --- | --- | --- | --- | --- | --- |
|  | Coefficient | SE | Coefficient | SE | Coefficient | SE |  |
| Galilei G6 vs. IOLMaster 500 | 0.0500031 | 0.241964 | 0.0491969 | 0.1492205 | 0.0008061 | 0.2842769 | 0.998 |
| Galilei G6 vs. Lenstar | −0.0499992 | 0.1757027 | 0.0279185 | 0.174343 | −0.0779178 | 0.2475215 | 0.753 |
| Galilei G6 vs. IOLMaster 700 | 0.0131305 | 0.2504326 | −0.0525615 | 0.1437472 | 0.065692 | 0.2882693 | 0.82 |
| Galilei G6 vs. Pentacam AXL | −0.1558143 | 0.4890821 | −0.2233683 | 0.1463638 | 0.067554 | 0.5103151 | 0.895 |
| IOLMaster vs. IOLMaster 500 | −0.0400009 | 0.2964307 | 0.0310263 | 0.0744105 | −0.0710272 | 0.3056274 | 0.816 |
| IOLMaster vs. Lenstar | −0.0245772 | 0.0653869 | −0.0660631 | 0.1261327 | 0.0414859 | 0.1419325 | 0.77 |
| IOLMaster vs. Aladdin | −0.1599998 | 0.1567475 | −0.0827252 | 0.1503199 | −0.0772746 | 0.217177 | 0.722 |
| IOLMaster vs. OA-2000 | 0.114079 | 0.1659448 | −0.0342891 | 0.1290437 | 0.1483681 | 0.2007053 | 0.46 |
| IOLMaster vs. Argos | 0.0200005 | 0.1856345 | 0.0139748 | 0.0969858 | 0.0060256 | 0.2094431 | 0.977 |
| IOLMaster 500 vs. AL-Scan | 0.0269002 | 0.0518625 | 0.0770552 | 0.1437776 | −0.050155 | 0.1528454 | 0.743 |
| IOLMaster 500 vs. Lenstar | −0.0710787 | 0.0895031 | −0.0556119 | 0.0577291 | −0.0154668 | 0.1065179 | 0.885 |
| IOLMaster 500 vs. Aladdin | −0.1300011 | 0.3048989 | −0.149227 | 0.1223154 | 0.0192259 | 0.3285185 | 0.953 |
| IOLMaster 500 vs. IOLMaster 700 | −0.074065 | 0.0669097 | −0.0979423 | 0.0683092 | 0.0238773 | 0.0956407 | 0.803 |
| IOLMaster 500 vs. OA-2000 | −2.79E−02 | 0.1436418 | 0.0121584 | 0.1373502 | −0.0400462 | 0.1986293 | 0.84 |
| IOLMaster 500 vs. Argos | −0.0274889 | 0.1342415 | −0.0041338 | 0.0916014 | −0.0233551 | 0.1624324 | 0.886 |
| IOLMaster 500 vs. Pentacam AXL | −0.2347538 | 0.1027911 | −0.3064526 | 0.112887 | 0.0716988 | 0.1527246 | 0.639 |
| AL-Scan vs. Lenstar | −0.1208065 | 0.1519978 | −0.086689 | 0.0712232 | −0.0341174 | 0.1678574 | 0.839 |
| AL-Scan vs. IOLMaster 700 | −0.1999969 | 0.2951955 | −0.1143229 | 0.0662511 | −0.085674 | 0.3025386 | 0.777 |
| Lenstar vs. Aladdin | −0.0800018 | 0.175265 | −0.0901932 | 0.1348053 | 0.0101913 | 0.2211115 | 0.963 |
| Lenstar vs. IOLMaster 700 | −0.0246357 | 0.0359796 | −0.0301344 | 0.0780262 | 0.0054987 | 0.0859229 | 0.949 |
| Lenstar vs. OA-2000 | 0.0341782 | 0.1063223 | 0.1091663 | 0.1679223 | −0.0749881 | 0.1851202 | 0.685 |
| Lenstar vs. Argos | 1.01E−10 | 0.2291288 | 0.0538905 | 0.075538 | −0.0538905 | 0.2412592 | 0.823 |
| Lenstar vs. Pentacam AXL | −0.2420161 | 0.1092007 | −0.177057 | 0.1012935 | −0.0649591 | 0.1489469 | 0.663 |
| Aladdin vs. IOLMaster 700 | −0.0699997 | 0.323259 | 0.0778122 | 0.1165609 | −0.1478119 | 0.3436318 | 0.667 |
| IOLMaster 700 vs. Argos | 0.081938 | 0.0857455 | 0.0608828 | 0.112573 | 0.0210552 | 0.1415096 | 0.882 |
| IOLMaster 700 vs. Pentacam AXL | −0.1647815 | 0.1425507 | −0.1881536 | 0.0898348 | 0.0233721 | 0.1686648 | 0.89 |

*CI* = confidence intervals; *SE* = standard error

Appendix II Table 8: Node-splitting analysis of inconsistency in mean keratometry for healthy people.

| Devices | Direct estimate (95% Cl) | | Indirect estimate (95% Cl) | | Overall (95% Cl) | | *P* value |
| --- | --- | --- | --- | --- | --- | --- | --- |
|  | Coefficient | SE | Coefficient | SE | Coefficient | SE |  |
| IOLMaster vs. Lenstar | −0.1520094 | 0.1297489 | −0.1152627 | 0.2118044 | −0.0367467 | 0.2483866 | 0.882 |
| IOLMaster vs. OA-2000 | −0.1289597 | 0.1669124 | −0.1657057 | 0.1839463 | 0.0367461 | 0.2483867 | 0.882 |
| IOLMaster 500 vs. Lenstar | −0.0363891 | 0.0850763 | −0.02143 | 0.1885899 | −0.0149591 | 0.2068916 | 0.942 |
| IOLMaster 500 vs. Aladdin | −0.1300011 | 0.3061746 | −0.071271 | 0.2212493 | −0.05873 | 0.3777488 | 0.876 |
| IOLMaster 500 vs. OA-2000 | −0.0149904 | 0.171711 | −0.056833 | 0.1596851 | 0.0418426 | 0.2344867 | 0.858 |
| AL-Scan vs. Lenstar | 0.0400009 | 0.2493563 | −0.2846307 | 940.418 | 0.3246316 | 940.4181 | 1 |
| Lenstar vs. Aladdin | −4.00E−02 | 0.2065425 | −0.0987308 | 0.316282 | 0.0587299 | 0.3777487 | 0.876 |
| Lenstar vs. OA-2000 | 0.0100021 | 0.201835 | −0.0094524 | 0.1328676 | 0.0194545 | 0.2416427 | 0.936 |
| Lenstar vs. Pentacam AXL | −0.170002 | 0.291809 | 0.1024359 | 0.2758051 | −0.2724379 | 0.4015233 | 0.497 |
| IOLMaster 700 vs. OA-2000 | −6.76E−10 | 0.2083243 | 0.2724371 | 0.3432518 | −0.2724371 | 0.4015231 | 0.497 |
| IOLMaster 700 vs. Pentacam AXL | 0.0833014 | 0.1386842 | −0.1891362 | 0.3768125 | 0.2724376 | 0.4015233 | 0.497 |
| Argos vs. Pentacam AXL | −0.2800026 | 0.1638355 | −0.3013227 | 789.706 | 0.0213201 | 789.706 | 1 |

*CI* = confidence intervals; *SE* = standard error

Appendix II Table 9: Node-splitting analysis of inconsistency in astigmatism for cataract people.

| Devices | Direct estimate (95% Cl) | | Indirect estimate (95% Cl) | | Overall (95% Cl) | | *P* value |
| --- | --- | --- | --- | --- | --- | --- | --- |
|  | Coefficient | SE | Coefficient | SE | Coefficient | SE |  |
| Galilei G6 vs. IOLMaster 500 | −2.40E−01 | 0.3520799 | 0.04402 | 0.2094433 | −0.2840199 | 0.4096666 | 0.488 |
| Galilei G6 vs. IOLMaster 700 | −1.28E−02 | 0.2168903 | −0.1214766 | 0.3151847 | 0.108687 | 0.3843973 | 0.777 |
| Galilei G6 vs. Pentacam AXL | 0.0933985 | 0.2783554 | −0.2384254 | 0.2996888 | 0.3318238 | 0.4158422 | 0.425 |
| IOLMaster vs. Lenstar | 6.57E−02 | 0.1297606 | −1.013879 | 0.4621003 | 1.079553 | 0.4813544 | 0.025 |
| IOLMaster vs. OA-2000 | −3.92E−01 | 0.2226865 | 0.0047125 | 0.1998993 | −0.3965635 | 0.3005756 | 0.187 |
| IOLMaster 500 vs. AL-Scan | −0.0499963 | 0.0692042 | −0.0702327 | 0.1220261 | 0.0202364 | 0.140284 | 0.885 |
| IOLMaster 500 vs. Lenstar | −1.93E−02 | 0.1172976 | 0.0202373 | 0.0857581 | −0.0395685 | 0.1452735 | 0.785 |
| IOLMaster 500 vs. Aladdin | −0.39 | 0.4372661 | −0.0582926 | 0.341426 | −0.3317074 | 0.5547733 | 0.55 |
| IOLMaster 500 vs. IOLMaster 700 | −0.0392359 | 0.0786164 | 0.0690395 | 0.15769 | −0.1082753 | 0.1762007 | 0.539 |
| IOLMaster 500 vs. OA-2000 | −0.0467896 | 0.1386512 | −0.3285592 | 0.182594 | 0.2817696 | 0.2300885 | 0.221 |
| IOLMaster 500 vs. Pentacam AXL | −0.0551789 | 0.1986656 | −0.0102682 | 0.1795934 | −0.0449107 | 0.2678093 | 0.867 |
| AL-Scan vs. Lenstar | 0.0681858 | 0.0813675 | 0.0479496 | 0.1142757 | 0.0202363 | 0.140284 | 0.885 |
| Lenstar vs. Aladdin | −0.07 | 0.3342155 | −0.4017073 | 0.4428017 | 0.3317073 | 0.5547733 | 0.55 |
| Lenstar vs. IOLMaster 700 | 0.0876752 | 0.1736339 | −0.0647715 | 0.1046719 | 0.1524467 | 0.2027436 | 0.452 |
| Lenstar vs. OA-2000 | −0.172575 | 0.1133952 | −0.0778665 | 0.2512984 | −0.0947085 | 0.2756454 | 0.731 |
| Lenstar vs. Pentacam AXL | −0.1 | 0.2213545 | 0.0027154 | 0.1754679 | −0.1027155 | 0.2824655 | 0.716 |
| IOLMaster 700 vs. Pentacam AXL | 0.0645058 | 0.2861108 | −0.0372933 | 0.1624349 | 0.1017992 | 0.3271022 | 0.756 |

*CI* = confidence intervals; *SE* = standard error

Appendix II Table 10: Node-splitting analysis of inconsistency in astigmatism for healthy people.

| Devices | Direct estimate (95% Cl) | | Indirect estimate (95% Cl) | | Overall (95% Cl) | | *P* value |
| --- | --- | --- | --- | --- | --- | --- | --- |
|  | Coefficient | SE | Coefficient | SE | Coefficient | SE |  |
| IOLMaster vs. Lenstar | −5.97E−02 | 0.1394017 | 0.0350135 | 110.4822 | −0.0946867 | 110.4823 | 0.999 |
| IOLMaster 500 vs. Lenstar | 1.24E−01 | 0.1454076 | −0.0101885 | 0.2127096 | 0.1344752 | 0.2576601 | 0.602 |
| IOLMaster 500 vs. Aladdin | −0.11 | 0.4257179 | 0.4089601 | 0.3254263 | −0.5189601 | 0.5358526 | 0.333 |
| IOLMaster 500 vs. IOLMaster 700 | 2.00E−02 | 0.0906831 | −0.0637431 | 0.3388444 | 0.083743 | 0.3507691 | 0.811 |
| IOLMaster 500 vs. OA-2000 | 2.00E−02 | 0.2983854 | 0.1187834 | 0.1444553 | −0.0987833 | 0.3315135 | 0.766 |
| IOLMaster 500 vs. Pentacam AXL | −0.03 | 0.1058121 | −0.0644686 | 0.3583875 | 0.0344686 | 0.3741317 | 0.927 |
| Lenstar vs. Aladdin | 3.00E−01 | 0.3011416 | −0.21896 | 0.4432287 | 0.51896 | 0.5358525 | 0.333 |
| Lenstar vs. OA-2000 | 0.02 | 0.0810163 | 0.0038753 | 0.2594819 | 0.0161246 | 0.2718354 | 0.953 |
| IOLMaster 700 vs. OA-2000 | 0.15 | 0.3075443 | 0.0662566 | 0.1686875 | 0.0837434 | 0.3507691 | 0.811 |
| IOLMaster 700 vs. Pentacam AXL | −0.05 | 0.1058121 | −0.0155311 | 0.3583876 | −0.0344688 | 0.3741318 | 0.927 |

*CI* = confidence intervals; *SE* = standard error

Appendix II Table 11: Node-splitting analysis of inconsistency in anterior chamber depth for cataract people.

| Devices | Direct estimate (95% Cl) | | Indirect estimate (95% Cl) | | Overall (95% Cl) | | *P* value |
| --- | --- | --- | --- | --- | --- | --- | --- |
|  | Coefficient | SE | Coefficient | SE | Coefficient | SE |  |
| Contact ultrasound vs. IOLMaster | 2.00E−01 | 0.0541675 | −0.1243932 | 0.0767001 | 0.3243124 | 0.0938855 | 0.001 |
| Contact ultrasound vs. AL-Scan | −3.00E−02 | 0.1288087 | 0.1736596 | 0.0748939 | −0.2036595 | 0.1489993 | 0.172 |
| Contact ultrasound vs. OA-1000 | 0.47 | 0.1320419 | 0.548626 | 0.2502757 | −0.078626 | 0.2822187 | 0.781 |
| Contact ultrasound vs. Lenstar | −1.40E−01 | 0.1184159 | 0.214269 | 0.0558794 | −0.3542689 | 0.1309383 | 0.007 |
| Contact ultrasound vs. Aladdin | 1.00E−01 | 0.1337817 | 0.184139 | 0.0835884 | −0.084139 | 0.1577485 | 0.594 |
| Contact ultrasound vs. IOLMaster 700 | 0.1300004 | 0.1301183 | 0.1294134 | 0.065366 | 0.000587 | 0.1456142 | 0.997 |
| Galilei G6 vs. IOLMaster 500 | −1.99E−07 | 0.1351074 | 0.0144923 | 0.0828568 | −0.0144925 | 0.1584906 | 0.927 |
| Galilei G6 vs. Lenstar | 0.0100001 | 0.1320241 | 0.062362 | 0.0831444 | −0.052362 | 0.1560236 | 0.737 |
| Galilei G6 vs. IOLMaster 700 | 0.0749163 | 0.1006264 | −0.0183587 | 0.0957442 | 0.093275 | 0.1391672 | 0.503 |
| Galilei G6 vs. Pentacam AXL | 0.0023207 | 0.1503698 | 0.0375859 | 0.1001604 | −0.0352652 | 0.1807612 | 0.845 |
| IOLMaster vs. IOLMaster 500 | 1.00E−02 | 0.1553466 | 0.0216772 | 0.0573211 | −0.011677 | 0.1655847 | 0.944 |
| IOLMaster vs. OA-1000 | 0.4100001 | 0.1315991 | 0.3313741 | 0.2509744 | 0.078626 | 0.2822186 | 0.781 |
| IOLMaster vs. Lenstar | 0.1164175 | 0.0622776 | −0.0194908 | 0.0708835 | 0.1359083 | 0.0943755 | 0.15 |
| IOLMaster vs. Aladdin | 0.03 | 0.12471 | 0.0824613 | 0.0813643 | −0.0524613 | 0.1489051 | 0.725 |
| IOLMaster vs. OA-2000 | 0.7037856 | 0.1042166 | 0.0114617 | 0.0643001 | 0.692324 | 0.1223456 | 0 |
| IOLMaster 500 vs. AL-Scan | 0.0320705 | 0.0660059 | −0.0200008 | 0.0725129 | 0.0520713 | 0.0980507 | 0.595 |
| IOLMaster 500 vs. Lenstar | 0.0843674 | 0.0583443 | 0.0086852 | 0.0450339 | 0.0756822 | 0.0737018 | 0.304 |
| IOLMaster 500 vs. Aladdin | 0.1800001 | 0.1427364 | 0.0094027 | 0.0751609 | 0.1705973 | 0.161316 | 0.29 |
| IOLMaster 500 vs. IOLMaster 700 | 0.0161412 | 0.0501064 | 0.0147376 | 0.0523617 | 0.0014037 | 0.0724707 | 0.985 |
| IOLMaster 500 vs. OA-2000 | 0.0306434 | 0.0706073 | 0.394442 | 0.0829818 | −0.3637986 | 0.1089412 | 0.001 |
| IOLMaster 500 vs. Argos | 0.1060576 | 0.0920892 | 0.0789907 | 0.0937623 | 0.0270668 | 0.1314822 | 0.837 |
| IOLMaster 500 vs. Pentacam AXL | 0.0448821 | 0.0901454 | −0.0081274 | 0.083063 | 0.0530095 | 0.1225642 | 0.665 |
| AL-Scan vs. Lenstar | 0.0114989 | 0.0825007 | 0.0386164 | 0.0637337 | −0.0271175 | 0.1042519 | 0.795 |
| AL-Scan vs. IOLMaster 700 | −0.0150001 | 0.1473439 | 0.0103287 | 0.0574844 | −0.0253288 | 0.1581603 | 0.873 |
| Lenstar vs. Aladdin | −0.0099998 | 0.1307106 | 0.0159543 | 0.0747519 | −0.025954 | 0.1505759 | 0.863 |
| Lenstar vs. IOLMaster 700 | −0.0169338 | 0.0493978 | −0.0264183 | 0.0513005 | 0.0094844 | 0.0712177 | 0.894 |
| Lenstar vs. OA-2000 | 0.2094024 | 0.0744127 | 0.0523948 | 0.0920718 | 0.1570076 | 0.1182603 | 0.184 |
| Lenstar vs. Argos | −0.0288858 | 0.1288528 | 0.0877619 | 0.079075 | −0.1166477 | 0.1512896 | 0.441 |
| Lenstar vs. Pentacam AXL | −0.0220047 | 0.0987064 | −0.0199994 | 0.0776556 | −0.0020053 | 0.1255849 | 0.987 |
| Aladdin vs. IOLMaster 700 | −0.0399999 | 0.1407355 | −0.0284218 | 0.076292 | −0.0115781 | 0.1600842 | 0.942 |
| IOLMaster 700 vs. Argos | 1.10E−01 | 0.0918363 | 0.0445846 | 0.0912163 | 0.0658864 | 0.1294381 | 0.611 |
| IOLMaster 700 vs. Pentacam AXL | −0.030499 | 0.0976638 | 0.0219207 | 0.0803724 | −0.0524198 | 0.1265092 | 0.679 |
| IOLMaster 700 vs. ANTERION | 0.0699999 | 0.1225029 | −0.2586232 | 283.1701 | 0.3286231 | 283.1701 | 0.999 |

*CI* = confidence intervals; *SE* = standard error

Appendix II Table 12: Node-splitting analysis of inconsistency in anterior chamber depth for healthy people.

| Devices | Direct estimate (95% Cl) | | Indirect estimate (95% Cl) | | Overall (95% Cl) | | *P* value |
| --- | --- | --- | --- | --- | --- | --- | --- |
|  | Coefficient | SE | Coefficient | SE | Coefficient | SE |  |
| Contact ultrasound vs. IOLMaster | 6.91E−02 | 0.0252109 | −0.0828906 | 0.1033345 | 0.1519466 | 0.1062949 | 0.153 |
| Contact ultrasound vs. AL-Scan | 3.00E−02 | 0.0686204 | 0.1819473 | 0.0811791 | −0.1519473 | 0.1062958 | 0.153 |
| IOLMaster vs. Lenstar | 0.0471144 | 0.0375373 | 0.0557857 | 0.049571 | −0.0086713 | 0.0627319 | 0.89 |
| IOLMaster vs. OA-2000 | 6.02E−02 | 0.0350414 | −0.005937 | 0.0506661 | 0.0661134 | 0.0619071 | 0.286 |
| IOLMaster 500 vs. Lenstar | 1.38E−01 | 0.0351263 | 0.0312554 | 0.0384548 | 0.1066647 | 0.052887 | 0.044 |
| IOLMaster 500 vs. Aladdin | 0.0499999 | 0.0590045 | 0.0892235 | 0.0656967 | −0.0392236 | 0.0883039 | 0.657 |
| IOLMaster 500 vs. IOLMaster 700 | 8.11E−10 | 0.0701235 | 0.0793958 | 0.0538879 | −0.0793958 | 0.0884376 | 0.369 |
| IOLMaster 500 vs. OA-2000 | 0.0502848 | 0.0458132 | 0.0926482 | 0.0456709 | −0.0423634 | 0.0652858 | 0.516 |
| AL-Scan vs. Lenstar | −0.05 | 0.0713055 | 0.1019474 | 0.078831 | −0.1519474 | 0.1062958 | 0.153 |
| Lenstar vs. Aladdin | −8.42E−10 | 0.056028 | −0.0392233 | 0.0682531 | 0.0392233 | 0.0883041 | 0.657 |
| Lenstar vs. OA-2000 | −3.09E−10 | 0.0593333 | −0.016582 | 0.0368576 | 0.016582 | 0.0698492 | 0.812 |
| Lenstar vs. Pentacam AXL | 0.02 | 0.0993658 | 0.0148207 | 0.0645542 | 0.0051793 | 0.1184939 | 0.965 |
| IOLMaster 700 vs. OA-2000 | 3.66E−12 | 0.0478375 | 0.064513 | 0.0666073 | −0.064513 | 0.0820059 | 0.431 |
| IOLMaster 700 vs. Pentacam AXL | 0.05 | 0.0422811 | 0.055179 | 0.1106939 | −0.0051789 | 0.118494 | 0.965 |
| Argos vs. Pentacam AXL | −0.05 | 0.0596263 | 0.2560912 | 273.574 | −0.3060911 | 273.5741 | 0.999 |

*CI* = confidence intervals; *SE* = standard error

Appendix II Table 13: Node-splitting analysis of inconsistency in aqueous depth for cataract people.

| Devices | Direct estimate (95% Cl) | | Indirect estimate (95% Cl) | | Overall (95% Cl) | | *P* value |
| --- | --- | --- | --- | --- | --- | --- | --- |
|  | Coefficient | SE | Coefficient | SE | Coefficient | SE |  |
| Galilei G6 vs. IOLMaster 700 | 1.00E−02 | 0.1568392 | −0.024016 | 17.75562 | 0.034016 | 17.75631 | 0.998 |
| IOLMaster 500 vs. Pentacam AXL | 1.00E−02 | 0.1553758 | 0.0188121 | 246.8297 | −0.0088121 | 246.8297 | 1 |
| AL-Scan vs. Lenstar | 0.0137267 | 0.1185458 | 0.0291257 | 0.1848877 | −0.0153989 | 0.2196279 | 0.944 |
| AL-Scan vs. IOLMaster 700 | 7.85E−11 | 0.1735909 | −0.0153988 | 0.134546 | 0.0153988 | 0.2196279 | 0.944 |
| Lenstar vs. Aladdin | 2.00E−02 | 0.1600009 | 0.0223265 | 0.1801102 | −0.0023265 | 0.2409149 | 0.992 |
| Lenstar vs. IOLMaster 700 | −0.0079439 | 0.0695244 | −0.0869661 | 0.1199172 | 0.0790222 | 0.1386139 | 0.569 |
| Lenstar vs. OA-2000 | 3.24E−01 | 0.1083835 | −0.0725736 | 184.2277 | 0.3962169 | 184.2277 | 0.998 |
| Lenstar vs. Argos | −0.03 | 0.1526346 | 0.1485636 | 0.1251493 | −0.1785636 | 0.1973821 | 0.366 |
| Lenstar vs. Pentacam AXL | −0.0284495 | 0.1136045 | −0.0474201 | 99.73541 | 0.0189706 | 99.73548 | 1 |
| Aladdin vs. IOLMaster 700 | −0.0500002 | 0.1685881 | −0.0476735 | 0.1720987 | −0.0023267 | 0.2409147 | 0.992 |
| IOLMaster 700 vs. Argos | 1.59E−01 | 0.1086701 | −0.0198438 | 0.1647744 | 0.1785637 | 0.197382 | 0.366 |
| IOLMaster 700 vs. ANTERION | 0.0800002 | 0.1471084 | −0.0199726 | 288.9025 | 0.0999728 | 288.9026 | 1 |

*CI* = confidence intervals; *SE* = standard error

Appendix II Table 14: Node-splitting analysis of inconsistency in central corneal thickness for cataract people.

| Devices | Direct estimate (95% Cl) | | Indirect estimate (95% Cl) | | Overall (95% Cl) | | *P* value |
| --- | --- | --- | --- | --- | --- | --- | --- |
|  | Coefficient | SE | Coefficient | SE | Coefficient | SE |  |
| Galilei G6 vs. IOLMaster 700 | −8.02E+00 | 5.548831 | 36.01382 | 17.91102 | −44.0387 | 18.77358 | 0.019 |
| Galilei G6 vs. Pentacam AXL | 5.86E+00 | 8.687454 | −23.58731 | 8.320893 | 29.44982 | 12.01273 | 0.014 |
| AL-Scan vs. Lenstar | −0.8621448 | 5.464869 | 14.27082 | 8.684253 | −15.13296 | 10.26083 | 0.14 |
| AL-Scan vs. IOLMaster 700 | 1.50E+01 | 8.331221 | −0.1199765 | 5.989612 | 15.13298 | 10.26083 | 0.14 |
| Lenstar vs. Aladdin | −1.00E+01 | 8.505622 | −7.532416 | 9.256627 | −2.467585 | 12.57103 | 0.844 |
| Lenstar vs. IOLMaster 700 | 1.139732 | 2.900596 | 3.336649 | 5.267318 | −2.196916 | 6.015698 | 0.715 |
| Lenstar vs. OA-2000 | −1.41E+01 | 5.07229 | 17.74053 | 12963.86 | −31.84532 | 12963.86 | 0.998 |
| Lenstar vs. Argos | 2.91E−07 | 7.477266 | −14.17176 | 5.543489 | 14.17176 | 9.308049 | 0.128 |
| Lenstar vs. Pentacam AXL | −8.315905 | 4.27012 | 13.71073 | 8.366534 | −22.02663 | 9.388772 | 0.019 |
| Aladdin vs. IOLMaster 700 | 9.289977 | 8.882315 | 11.75755 | 8.895792 | −2.467577 | 12.57102 | 0.844 |
| IOLMaster 700 vs. Argos | −1.48E+01 | 4.976352 | −0.6176179 | 7.869584 | −14.17177 | 9.308037 | 0.128 |
| IOLMaster 700 vs. Pentacam AXL | 8.221266 | 9.14949 | −9.048854 | 5.11323 | 17.27012 | 10.50268 | 0.1 |

*CI* = confidence intervals; *SE* = standard error

Appendix II Table 15: Node-splitting analysis of inconsistency in central corneal thickness for healthy people.

| Devices | Direct estimate (95% Cl) | | Indirect estimate (95% Cl) | | Overall (95% Cl) | | *P* value |
| --- | --- | --- | --- | --- | --- | --- | --- |
|  | Coefficient | SE | Coefficient | SE | Coefficient | SE |  |
| AL-Scan vs. Lenstar | −1.13E+01 | 3.410027 | 1.445925 | 3922.539 | −12.78252 | 3922.541 | 0.997 |
| Lenstar vs. IOLMaster 700 | 5.00E+00 | 7.144345 | 2.688278 | 5.260606 | 2.311722 | 8.872184 | 0.794 |
| Lenstar vs. OA-2000 | −12.83002 | 4.546497 | −14.15937 | 7.230622 | 1.329349 | 8.541225 | 0.876 |
| Lenstar vs. Pentacam AXL | −3.50E+00 | 8.295812 | 1.614555 | 6.216911 | −5.114557 | 10.3668 | 0.622 |
| IOLMaster 700 vs. OA-2000 | −1.71E+01 | 4.520251 | −15.75066 | 7.247059 | −1.329362 | 8.541226 | 0.876 |
| IOLMaster 700 vs. Pentacam AXL | −2.969972 | 3.985192 | −8.084553 | 9.570198 | 5.114581 | 10.3668 | 0.622 |
| Argos vs. Pentacam AXL | 1.16E+00 | 3.530963 | −23.12408 | 20902.73 | 24.28405 | 20902.74 | 0.999 |

*CI* = confidence intervals; *SE* = standard error

Appendix II Table 16: Node-splitting analysis of inconsistency in corneal diameter for cataract people.

| Devices | Direct estimate (95% Cl) | | Indirect estimate (95% Cl) | | Overall (95% Cl) | | *P* value |
| --- | --- | --- | --- | --- | --- | --- | --- |
|  | Coefficient | SE | Coefficient | SE | Coefficient | SE |  |
| Galilei G6 vs. Lenstar | −1.60E−01 | 0.240568 | 0.1111245 | 0.2645569 | −0.2711243 | 0.3575798 | 0.448 |
| Galilei G6 vs. IOLMaster 700 | 6.00E−02 | 0.2433061 | −0.2110994 | 0.2620437 | 0.2710989 | 0.3575818 | 0.448 |
| IOLMaster vs. IOLMaster 500 | −0.2699995 | 0.2504133 | −0.0107341 | 0.2427661 | −0.2592654 | 0.3487724 | 0.457 |
| IOLMaster vs. Lenstar | −7.00E−02 | 0.2675879 | −0.1749697 | 0.2190425 | 0.10497 | 0.3458075 | 0.761 |
| IOLMaster vs. Aladdin | −3.90E−01 | 0.2408963 | −0.5781646 | 0.3135432 | 0.188164 | 0.395399 | 0.634 |
| IOLMaster 500 vs. AL-Scan | −0.5199995 | 0.2399881 | −0.1727526 | 0.1736305 | −0.347247 | 0.2962125 | 0.241 |
| IOLMaster 500 vs. Lenstar | 8.00E−02 | 0.2444718 | −0.0196442 | 0.1342483 | 0.0996796 | 0.2788981 | 0.721 |
| IOLMaster 500 vs. IOLMaster 700 | 0.002527 | 0.1244107 | −0.0822587 | 0.1803622 | 0.0847858 | 0.2190957 | 0.699 |
| IOLMaster 500 vs. OA-2000 | −0.1253186 | 0.178115 | −0.2177024 | 0.5062748 | 0.0923838 | 0.5364749 | 0.863 |
| IOLMaster 500 vs. Pentacam AXL | −0.2407389 | 0.1686752 | −0.7163463 | 0.4654443 | 0.4756073 | 0.4950629 | 0.337 |
| AL-Scan vs. Lenstar | 1.55E−01 | 0.1685264 | 0.4850365 | 0.19575 | −0.3296284 | 0.2583008 | 0.202 |
| AL-Scan vs. IOLMaster 700 | 0.3200007 | 0.250192 | 0.2431209 | 0.1700092 | 0.0768798 | 0.3024883 | 0.799 |
| Lenstar vs. Aladdin | −0.3999998 | 0.2462865 | −0.2118341 | 0.3093277 | −0.1881657 | 0.3953994 | 0.634 |
| Lenstar vs. IOLMaster 700 | 0.0551028 | 0.1390094 | −0.1140329 | 0.1410773 | 0.1691358 | 0.1980595 | 0.393 |
| Lenstar vs. OA-2000 | −0.1201587 | 0.2456361 | −0.1628515 | 0.2777145 | 0.0426928 | 0.3706828 | 0.908 |
| Lenstar vs. Argos | −0.5599995 | 0.1645723 | 0.5101177 | 0.167304 | −1.070117 | 0.2346799 | 0 |
| IOLMaster 700 vs. Argos | 0.4499998 | 0.1533609 | −0.6201172 | 0.1776376 | 1.070117 | 0.2346799 | 0 |
| IOLMaster 700 vs. Pentacam AXL | −0.3297098 | 0.2426112 | −0.2061819 | 0.2562876 | −0.123528 | 0.3529711 | 0.726 |

*CI* = confidence intervals; *SE* = standard errorAppendix II Table 17: Node-splitting analysis of inconsistency in corneal diameter for healthy people.

| Devices | Direct estimate (95% Cl) | | Indirect estimate (95% Cl) | | Overall (95% Cl) | | *P* value |
| --- | --- | --- | --- | --- | --- | --- | --- |
|  | Coefficient | SE | Coefficient | SE | Coefficient | SE |  |
| IOLMaster vs. Lenstar | −6.11E−02 | 0.149827 | 0.1309393 | 0.2091125 | −0.1919953 | 0.257243 | 0.455 |
| IOLMaster vs. OA-2000 | −1.75E−01 | 0.151473 | −0.3666055 | 0.2079194 | 0.1919899 | 0.257243 | 0.455 |
| IOLMaster 500 vs. Lenstar | 0.1849673 | 0.1285587 | −0.1662874 | 0.1744401 | 0.3512546 | 0.2166249 | 0.105 |
| IOLMaster 500 vs. IOLMaster 700 | −1.00E−01 | 0.2357731 | −0.0336371 | 0.2516677 | −0.0663633 | 0.3448558 | 0.847 |
| IOLMaster 500 vs. OA-2000 | −2.98E−01 | 0.1393707 | −0.0309621 | 0.1600551 | −0.267465 | 0.2122812 | 0.208 |
| AL-Scan vs. Lenstar | 0.1300001 | 0.2087255 | 0.0079569 | 310.9894 | 0.1220432 | 310.9894 | 1 |
| Lenstar vs. Aladdin | −3.40E−01 | 0.2004803 | −0.0097659 | 273.1038 | −0.3302343 | 273.1038 | 0.999 |
| Lenstar vs. OA-2000 | −0.1299991 | 0.2081119 | −0.3031142 | 0.1474037 | 0.1731151 | 0.2550263 | 0.497 |
| IOLMaster 700 vs. OA-2000 | −0.1400003 | 0.2171663 | −0.0736411 | 0.2678882 | −0.0663592 | 0.3448555 | 0.847 |
| IOLMaster 700 vs. Pentacam AXL | −0.2200003 | 0.1990518 | 0.2531292 | 245.2845 | −0.4731295 | 245.2845 | 0.998 |
| IOLMaster 700 vs. ANTERION | −1.20E−01 | 0.2071432 | 0.2527343 | 260.4344 | −0.3727342 | 260.4344 | 0.999 |

*CI* = confidence intervals; *SE* = standard error

Appendix II Table 18: Node-splitting analysis of inconsistency in lens thickness for cataract people.

| Devices | Direct estimate (95% Cl) | | Indirect estimate (95% Cl) | | Overall (95% Cl) | | *P* value |
| --- | --- | --- | --- | --- | --- | --- | --- |
|  | Coefficient | SE | Coefficient | SE | Coefficient | SE |  |
| Galilei G6 vs. Lenstar | −1.10E−01 | 0.0849326 | 0.0699578 | 0.1060036 | −0.1799579 | 0.1358319 | 0.185 |
| Galilei G6 vs. IOLMaster 700 | 1.18E−01 | 0.1021731 | −0.0619528 | 0.0899395 | 0.1799591 | 0.1358325 | 0.185 |
| Lenstar vs. Aladdin | 0.1600003 | 0.0880444 | 0.0734933 | 0.1084126 | 0.086507 | 0.1396607 | 0.536 |
| Lenstar vs. IOLMaster 700 | 3.02E−02 | 0.0296851 | 0.1671079 | 0.0644552 | −0.1369283 | 0.0704192 | 0.052 |
| Lenstar vs. OA-2000 | 1.36E−01 | 0.0497656 | 0.0803836 | 175.1099 | 0.0556612 | 175.1099 | 1 |
| Lenstar vs. Argos | 0.1999998 | 0.0837753 | 0.0974563 | 0.0653236 | 0.1025435 | 0.1062331 | 0.334 |
| Aladdin vs. IOLMaster 700 | −2.00E−02 | 0.1039712 | −0.106507 | 0.0932473 | 0.086507 | 0.1396605 | 0.536 |
| IOLMaster 700 vs. Argos | 0.0488835 | 0.0578089 | 0.1514269 | 0.0892948 | −0.1025434 | 0.1062334 | 0.334 |

*CI* = confidence intervals; *SE* = standard error

Appendix II Table 19: Node-splitting analysis of inconsistency in keratometry in the flattest meridian for different measurement principles.

| Measurement principles | Direct estimate (95% Cl) | | Indirect estimate (95% Cl) | | Overall (95% Cl) | | *P* value |
| --- | --- | --- | --- | --- | --- | --- | --- |
|  | Coefficient | SE | Coefficient | SE | Coefficient | SE |  |
| Placido vs. Automated keratometer | 0.0769109 | 0.08009 | −0.0488324 | 0.9876723 | 0.1257434 | 0.9902758 | 0.899 |
| Placido vs. Scheimflug | −0.3294597 | 0.547897 | −0.0697014 | 0.1796012 | −0.2597583 | 0.5766275 | 0.652 |
| Automated keratometer vs. Scheimflug | −0.1570341 | 0.1540194 | −0.7567543 | 0.9475256 | 0.5997202 | 0.9575981 | 0.531 |

*CI* = confidence intervals; *SE* = standard error

Appendix II Table 20: Node-splitting analysis of inconsistency in central corneal thickness for different measurement principles.

| Measurement principles | Direct estimate (95% Cl) | | Indirect estimate (95% Cl) | | Overall (95% Cl) | | *P* value |
| --- | --- | --- | --- | --- | --- | --- | --- |
|  | Coefficient | SE | Coefficient | SE | Coefficient | SE |  |
| A-Scan ultrasound vs. OLCR | −6.000003 | 11.65243 | 0.2785806 | 764.7603 | −6.278584 | 764.849 | 0.993 |
| OLCR vs. OLCI | −9.999998 | 8.784301 | −11.79421 | 9.384211 | 1.794207 | 12.85408 | 0.889 |
| OLCR vs. SS-OCT | −2.661465 | 2.351677 | −1.75823 | 4.298001 | −0.9032352 | 4.893263 | 0.854 |
| OLCR vs. Scheimpflug | 0.5999374 | 3.04856 | −0.1047656 | 4.10822 | 0.7047031 | 5.114174 | 0.89 |
| OLCI vs. SS-OCT | 9.289968 | 9.149553 | 7.495752 | 9.028505 | 1.794216 | 12.85411 | 0.889 |
| SS-OCT vs. Scheimpflug | 2.491971 | 3.397463 | 3.196832 | 3.821048 | −0.7048618 | 5.11418 | 0.89 |

*CI* = confidence intervals; *SE* = Standard error; *OLCR* = optical low-coherence reflectometry; *OLC*I = optical low-coherence interferometry; *SS-OCT* = swept-source optical coherence tomography

Appendix II Table 21: Node-splitting analysis of inconsistency in anterior chamber depth for different measurement principles.

| Measurement principles | Direct estimate (95% Cl) | | Indirect estimate (95% Cl) | | Overall (95% Cl) | | *P* value |
| --- | --- | --- | --- | --- | --- | --- | --- |
|  | Coefficient | SE | Coefficient | SE | Coefficient | SE |  |
| A-Scan ultrasound vs. PCI | 0.1329841 | 0.0268858 | −0.0137624 | 0.0416563 | 0.1467465 | 0.0495732 | 0.003 |
| A-Scan ultrasound vs. OCLR | 0.0119178 | 0.0527865 | 0.1568341 | 0.0313762 | −0.1449163 | 0.0614066 | 0.018 |
| A-Scan ultrasound vs. OCLI | 0.0999999 | 0.1171003 | 0.1416503 | 0.0496459 | −0.0416504 | 0.1271896 | 0.743 |
| A-Scan ultrasound vs. SS-OCT | 0.1300001 | 0.1126705 | 0.1281571 | 0.0297021 | 0.001843 | 0.1165198 | 0.987 |
| A-Scan ultrasound vs. Scheimpflug | −0.001284 | 0.0811106 | 0.1288398 | 0.0350928 | −0.1301238 | 0.0883786 | 0.141 |
| PCI vs. OCLR | 0.0687089 | 0.0263624 | −0.0200293 | 0.0294797 | 0.0887382 | 0.0395516 | 0.025 |
| PCI vs. OCLI | 0.058844 | 0.0579799 | 0.0317287 | 0.0601557 | 0.0271153 | 0.0835471 | 0.746 |
| PCI vs. SS-OCT | 0.0476037 | 0.0238521 | 0.0190743 | 0.0355924 | 0.0285294 | 0.0428445 | 0.505 |
| PCI vs. Scheimpflug | 0.0312127 | 0.0430569 | 0.0109798 | 0.0338364 | 0.0202329 | 0.054761 | 0.712 |
| OCLR vs. OCLI | −0.0048881 | 0.0792613 | 0.0257697 | 0.0516845 | −0.0306578 | 0.0946239 | 0.746 |
| OCLR vs. SS-OCT | 0.0353401 | 0.0308772 | −0.0176956 | 0.0317533 | 0.0530357 | 0.0442835 | 0.231 |
| OCLR vs. Scheimpflug | −0.00435 | 0.042232 | −0.0148179 | 0.0356615 | 0.010468 | 0.0552759 | 0.85 |
| OCLI vs. SS-OCT | −0.04 | 0.1248161 | −0.0022897 | 0.047269 | −0.0377103 | 0.1334669 | 0.778 |
| SS-OCT vs. Scheimpflug | −0.0037099 | 0.0448862 | −0.0302117 | 0.0353784 | 0.0265018 | 0.0571532 | 0.643 |

*CI* = confidence intervals; *SE* = Standard error; *PCI* = partial coherence interferometry; *OLCR* = optical low-coherence reflectometry; *OLCI* = optical low-coherence interferometry; *SS-OC*T = swept-source optical coherence tomography.

Appendix II Table 22: Results of network rank test in axial length for cataract people.

| Devices | SUCRA value (%) | PrBest (%) |
| --- | --- | --- |
| Contact ultrasound | 2.9 | 0.0 |
| OA-1000 | 28.6 | 4.1 |
| AL-Scan | 38.3 | 0.7 |
| Argos | 42.5 | 1.7 |
| IOLMaster 500 | 44.7 | 0.2 |
| ANTERION | 46.3 | 8.2 |
| IOLMaster 700 | 46.7 | 0.7 |
| Pentacam AXL | 46.8 | 2.3 |
| Galilei G6 | 50.7 | 10.7 |
| OA-2000 | 62.5 | 3.5 |
| Lenstar | 67.3 | 3.2 |
| Immersion ultrasound | 69.3 | 41.8 |
| Aladdin | 75.0 | 13.3 |
| IOLMaster | 78.4 | 9.6 |

*SCURA* = surface under the cumulative ranking curve; *PrBest* = the probability of having the maximum value

Appendix II Table 23: Results of network rank test in axial length for healthy people.

| Devices | SUCRA value (%) | PrBest (%) |
| --- | --- | --- |
| Contact ultrasound | 13.6 | 0.1 |
| IOLMaster | 38.8 | 0.6 |
| Pentacam AXL | 44.3 | 4.7 |
| Argos | 49.4 | 19.1 |
| IOLMaster 500 | 52.2 | 2.5 |
| Aladdin | 53.7 | 16.5 |
| IOLMaster 700 | 52.9 | 7.1 |
| OA-2000 | 53.8 | 4.8 |
| Lenstar | 61.9 | 3.1 |
| AL-Scan | 79.3 | 41.5 |

*SCURA* = surface under the cumulative ranking curve; *PrBest* = the probability of having the maximum value

Appendix II Table 24. Results of network rank test in keratometry in the flattest meridian for cataract people.

| Devices | SUCRA value (%) | PrBest (%) |
| --- | --- | --- |
| Pentacam AXL | 11.5 | 0.2 |
| Lenstar | 34.1 | 0.0 |
| Aladdin | 44.0 | 8.3 |
| Galilei G6 | 45.5 | 8.8 |
| IOLMaster 700 | 45.9 | 1.1 |
| IOLMaster | 48.0 | 1.0 |
| IOLMaster 500 | 58.4 | 1.3 |
| AL-Scan | 71.0 | 16.8 |
| OA-2000 | 91.7 | 62.5 |

*SCURA* = surface under the cumulative ranking curve; *PrBest* = the probability of having the maximum value

Appendix II Table 25. Results of network rank test in keratometry in the flattest meridian for healthy people.

| Devices | SUCRA value (%) | PrBest (%) |
| --- | --- | --- |
| OA-2000 | 14.2 | 0.0 |
| IOLMaster 700 | 29.1 | 4.5 |
| Aladdin | 44.7 | 5.9 |
| Lenstar | 58.1 | 1.7 |
| IOLMaster 500 | 59.7 | 6.9 |
| IOLMaster | 94.2 | 81.0 |

*SCURA* = surface under the cumulative ranking curve; *PrBest* = the probability of having the maximum value

Appendix II Table 26. Results of network rank test in keratometry in the steepest meridian for cataract people.

| Devices | SUCRA value (%) | PrBest (%) |
| --- | --- | --- |
| Pentacam AXL | 13.8 | 0.1 |
| OA-2000 | 25.0 | 0.8 |
| Aladdin | 42.6 | 12.1 |
| Galilei G6 | 49.5 | 15.8 |
| IOLMaster 700 | 52.6 | 5.7 |
| Lenstar | 53.8 | 2.4 |
| AL-Scan | 65.4 | 22.3 |
| IOLMaster | 71.8 | 24.2 |
| IOLMaster 500 | 75.5 | 16.6 |

*SCURA* = surface under the cumulative ranking curve; *PrBest* = the probability of having the maximum value

Appendix II Table 27. Results of network rank test in keratometry in the steepest meridian for healthy people.

| Devices | SUCRA value (%) | PrBest (%) |
| --- | --- | --- |
| Aladdin | 36.2 | 10.9 |
| Lenstar | 39.3 | 1.6 |
| OA-2000 | 39.1 | 2.6 |
| IOLMaster 700 | 42.6 | 18 |
| IOLMaster 500 | 64.7 | 18.5 |
| IOLMaster | 78.2 | 48.4 |

*SCURA* = surface under the cumulative ranking curve; *PrBest* = the probability of having the maximum value

Appendix II Table 28. Results of network rank test in mean keratometry for cataract people.

| Devices | SUCRA value (%) | PrBest (%) |
| --- | --- | --- |
| Pentacam AXL | 3.0 | 0.0 |
| Aladdin | 23.0 | 1.9 |
| IOLMaster 700 | 32.1 | 0.0 |
| Lenstar | 44.5 | 0.3 |
| Galilei G6 | 52.6 | 18.2 |
| IOLMaster | 60.1 | 6.4 |
| OA-2000 | 64.8 | 21.4 |
| Argos | 65.7 | 15.4 |
| IOLMaster 500 | 71.9 | 5.3 |
| AL-Scan | 82.5 | 31.1 |

*SCURA* = surface under the cumulative ranking curve; *PrBest* = the probability of having the maximum value

Appendix II Table 29. Results of network rank test in mean keratometry for healthy people.

| Devices | SUCRA value (%) | PrBest (%) |
| --- | --- | --- |
| IOLMaster 700 | 30.7 | 0.8 |
| Aladdin | 34.0 | 4.2 |
| Pentacam AXL | 41.5 | 0.3 |
| AL-Scan | 42.3 | 13.3 |
| Lenstar | 43.7 | 0.5 |
| OA-2000 | 43.2 | 0.8 |
| IOLMaster 500 | 54.1 | 2.5 |
| IOLMaster | 75.7 | 19.5 |
| Argos | 84.8 | 58.1 |

*SCURA* = surface under the cumulative ranking curve; *PrBest* = the probability of having the maximum value

Appendix II Table 30. Results of network rank test in astigmatism for cataract people.

| Devices | SUCRA value (%) | PrBest (%) |
| --- | --- | --- |
| OA-2000 | 20.6 | 0.5 |
| Aladdin | 28.3 | 11.2 |
| AL-Scan | 40.9 | 1.6 |
| Pentacam AXL | 49.9 | 11.4 |
| IOLMaster 700 | 55.9 | 5.9 |
| Galilei G6 | 60.7 | 28.9 |
| IOLMaster 500 | 63.9 | 6.1 |
| IOLMaster | 65.2 | 27.1 |
| Lenstar | 64.6 | 7.3 |

*SCURA* = surface under the cumulative ranking curve; *PrBest* = the probability of having the maximum value

Appendix II Table 31. Results of network rank test in astigmatism for healthy people.

| Devices | SUCRA value (%) | PrBest (%) |
| --- | --- | --- |
| Pentacam AXL | 25.5 | 1.8 |
| IOLMaster 500 | 31.9 | 1.7 |
| IOLMaster 700 | 39.3 | 3.8 |
| Lenstar | 53.0 | 2.9 |
| OA-2000 | 58.0 | 7.6 |
| IOLMaster | 66.4 | 27.5 |
| Aladdin | 75.9 | 54.7 |

*SCURA* = surface under the cumulative ranking curve; *PrBest* = the probability of having the maximum value

Appendix II Table 32. Results of network rank test in anterior chamber depth for cataract people.

| Devices | SUCRA value (%) | PrBest (%) |
| --- | --- | --- |
| Contact ultrasound | 2.7 | 0.0 |
| IOLMaster | 27.4 | 0.0 |
| Galilei G6 | 32.1 | 0.0 |
| IOLMaster 500 | 34.7 | 0.0 |
| AL-Scan | 40.2 | 0.0. |
| Pentacam AXL | 42.5 | 0.1 |
| IOLMaster 700 | 43.0 | 0.0 |
| Aladdin | 53.6 | 0.0 |
| Lenstar | 55.0 | 0.0 |
| ANTERION | 61.5 | 4.6 |
| Argos | 70.8 | 0.6 |
| OA-2000 | 88.4 | 7.7 |
| OA-1000 | 98.3 | 87 |

*SCURA* = surface under the cumulative ranking curve; *PrBest* = the probability of having the maximum value

Appendix II Table 33. Results of network rank test in anterior chamber depth for healthy people.

| Devices | SUCRA value (%) | PrBest (%) |
| --- | --- | --- |
| Contact ultrasound | 5.6 | 0.0 |
| IOLMaster 500 | 14.7 | 0.0 |
| IOLMaster | 34.4 | 0.0 |
| IOLMaster 700 | 42.4 | 0.3 |
| AL-Scan | 55.0 | 10.7 |
| Aladdin | 56.0 | 5.9 |
| OA-2000 | 61.4 | 3.2 |
| Lenstar | 69.7 | 6.7 |
| Pentacam AXL | 74.1 | 10.6 |
| Argos | 86.6 | 62.6 |

*SCURA* = surface under the cumulative ranking curve; *PrBest* = the probability of having the maximum value

Appendix II Table 34. Results of network rank test in aqueous depth for cataract people.

| Devices | SUCRA value (%) | PrBest (%) |
| --- | --- | --- |
| IOLMaster 700 | 33.8 | 0.0 |
| Pentacam AXL | 37.9 | 0.5 |
| Galilei G6 | 39.0 | 1.6 |
| IOLMaster 500 | 38.5 | 4.6 |
| AL-Scan | 39.4 | 0.3 |
| Lenstar | 44.2 | 0.0 |
| Aladdin | 50.2 | 1.2 |
| ANTERION | 53.9 | 6.3 |
| Argos | 65.8 | 2.1 |
| OA-2000 | 97.2 | 83.4 |

*SCURA* = surface under the cumulative ranking curve; *PrBest* = the probability of having the maximum value

Appendix II Table 35. Results of network rank test in central corneal thickness for cataract people.

| Devices | SUCRA value (%) | PrBest (%) |
| --- | --- | --- |
| OA-2000 | 8.5 | 0.0 |
| Argos | 23.9 | 0.1 |
| Aladdin | 28.6 | 1.7 |
| AL-Scan | 48.9 | 4.6 |
| Pentacam AXL | 50.6 | 3.7 |
| Lenstar | 68.9 | 4.7 |
| IOLMaster 700 | 80.4 | 14.3 |
| Galilei G6 | 90.2 | 70.9 |

*SCURA* = surface under the cumulative ranking curve; *PrBest* = the probability of having the maximum value

Appendix II Table 36. Results of network rank test in central corneal thickness for healthy people.

| Devices | SUCRA value (%) | PrBest (%) |
| --- | --- | --- |
| OA-2000 | 0.7 | 0.0 |
| Argos | 39.7 | 2.3 |
| Lenstar | 45.5 | 0.0 |
| Pentacam AXL | 46.1 | 0.7 |
| IOLMaster700 | 70.9 | 6.7 |
| AL-Scan | 97.1 | 90.3 |

*SCURA* = surface under the cumulative ranking curve; *PrBest* = the probability of having the maximum value

Appendix II Table 37. Results of network rank test in corneal diameter for cataract people.

| Devices | SUCRA value (%) | PrBest (%) |
| --- | --- | --- |
| Aladdin | 16.4 | 0.0 |
| AL-Scan | 17.2 | 0.1 |
| Pentacam AXL | 18.3 | 0.1 |
| OA-2000 | 42.5 | 4.2 |
| Argos | 54.9 | 10.5 |
| IOLMaster 700 | 60.7 | 2.1 |
| IOLMaster 500 | 66.1 | 5.3 |
| Lenstar | 68.2 | 4.3 |
| Galilei G6 | 71.3 | 25.4 |
| IOLMaster | 84.4 | 48.0 |

*SCURA* = surface under the cumulative ranking curve; *PrBest* = the probability of having the maximum value

Appendix II Table 38. Results of network rank test in corneal diameter for healthy people.

| Devices | SUCRA value (%) | PrBest (%) |
| --- | --- | --- |
| Aladdin | 23.6 | 2.1 |
| Pentacam AXL | 23.6 | 3.2 |
| OA-2000 | 30.6 | 0.0 |
| ANTERION | 38.2 | 9.5 |
| AL-Scan | 52.8 | 14.0 |
| IOLMaster 700 | 55.1 | 6.6 |
| IOLMaster 500 | 67.9 | 10.6 |
| IOLMaster | 78.1 | 29.7 |

*SCURA* = surface under the cumulative ranking curve; *PrBest* = the probability of having the maximum value

Appendix II Table 39. Results of network rank test in lens thickness for cataract people.

| Devices | SUCRA value (%) | PrBest (%) |
| --- | --- | --- |
| Lenstar | 7.3 | 0.0 |
| Galilei G6 | 29.8 | 3.0 |
| IOLMaster700 | 37.7 | 0.1 |
| Aladdin | 70.5 | 29.6 |
| OA-2000 | 77.5 | 34.0 |
| Argos | 77.3 | 33.3 |

*SCURA* = surface under the cumulative ranking curve; *PrBest* = the probability of having the maximum value

Appendix II Table 40. Results of network rank test in keratometry in the flattest meridian for different measurement principles.

| Measurement principles | SUCRA value (%) | PrBest (%) |
| --- | --- | --- |
| Scheimflug | 21.6 | 13.7 |
| Placido | 43.1 | 14.0 |
| Automated keratometer | 85.3 | 72.3 |

*SCURA* = surface under the cumulative ranking curve; *PrBest* = the probability of having the maximum value

Appendix II Table 41. Results of network rank test in central corneal thickness for different measurement principles.

| Measurement principles | SUCRA value (%) | PrBest (%) |
| --- | --- | --- |
| OLCI | 7.4 | 1.1 |
| SS-OCT | 35.4 | 1.2 |
| OLCR | 65.1 | 13.1 |
| Scheimpflug | 66.5 | 19.3 |
| A-Scan ultrasound | 75.6 | 65.3 |

*SCURA* = surface under the cumulative ranking curve; *PrBest* = the probability of having the maximum value; *OLCR* = optical low-coherence reflectometry; *OLCI* = optical low-coherence interferometry; *SS-OCT* = swept-source optical coherence tomography

Appendix II Table 42. Results of network rank test in anterior chamber depth for different measurement principles.

| Measurement principles | SUCRA value (%) | PrBest (%) |
| --- | --- | --- |
| A-Scan ultrasound | 0.1 | 0.0 |
| PCI | 29.7 | 0.0 |
| Scheimpflug | 53.0 | 10.3 |
| OLCR | 65.0 | 11.6 |
| OLCI | 75.2 | 48.8 |
| SS-OCT | 77.1 | 29.3 |

*SCURA* = surface under the cumulative ranking curve; *PrBest* = the probability of having the maximum value; *PCI* = partial coherence interferometry; *OLCR* = optical low-coherence reflectometry; *OLCI* = optical low-coherence interferometry; *SS-OCT* = swept-source optical coherence tomography


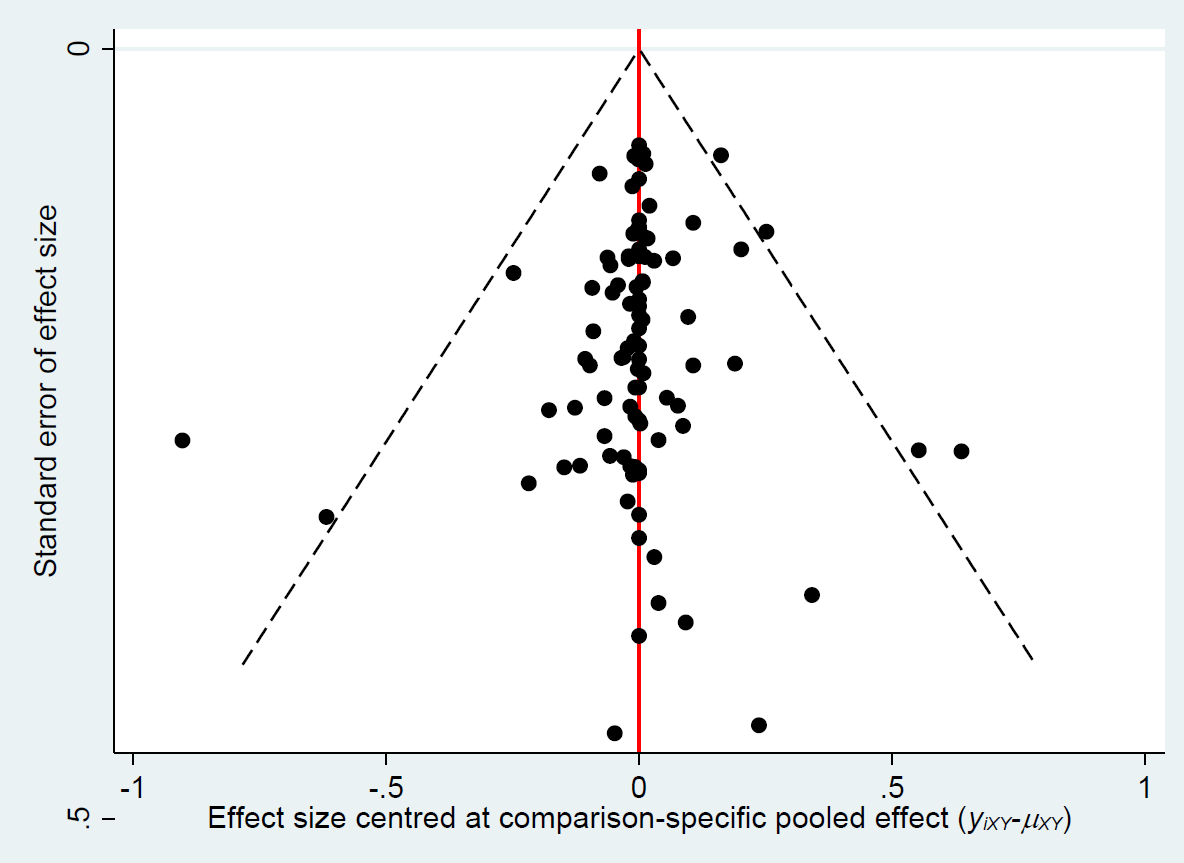


Appendix II Figure 1: The funnel plot in subgroup analysis for the axial length measurement in cataract subjects.


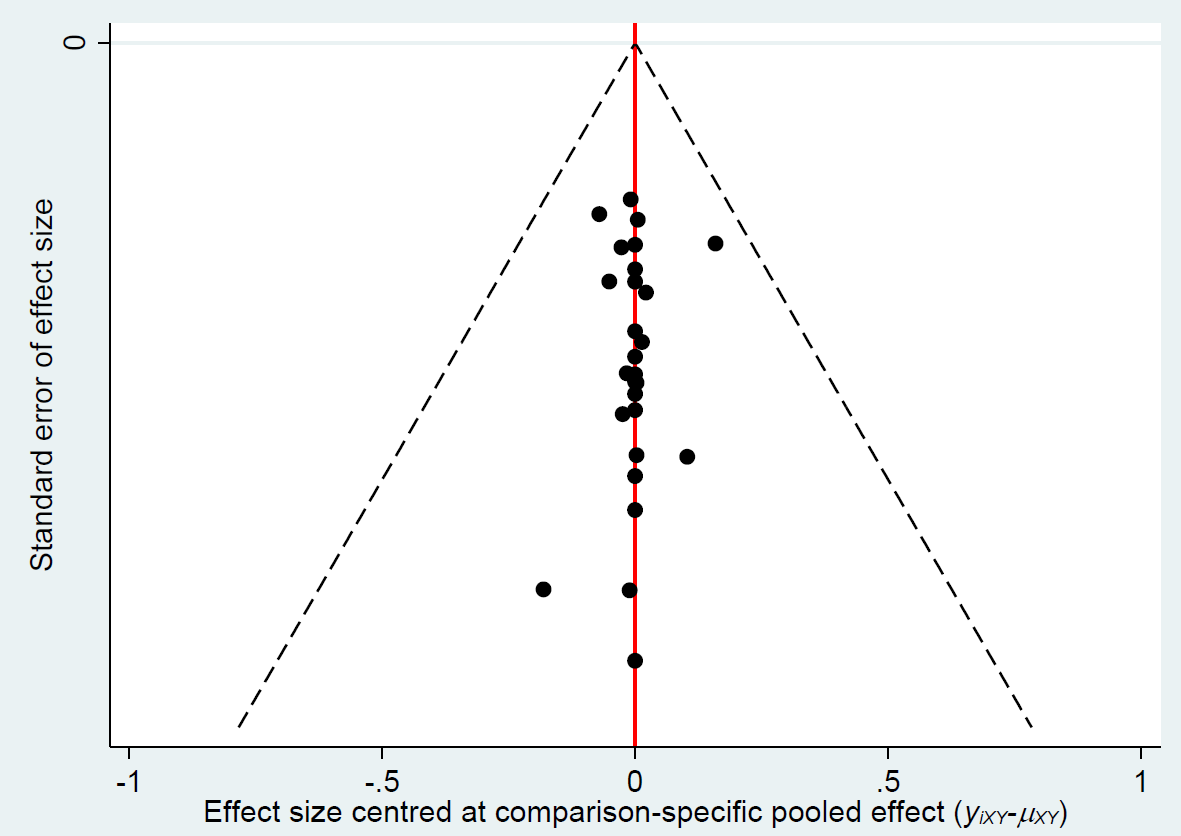


Appendix II Figure 2: The funnel plot in subgroup analysis for the axial length measurement in healthy subjects.


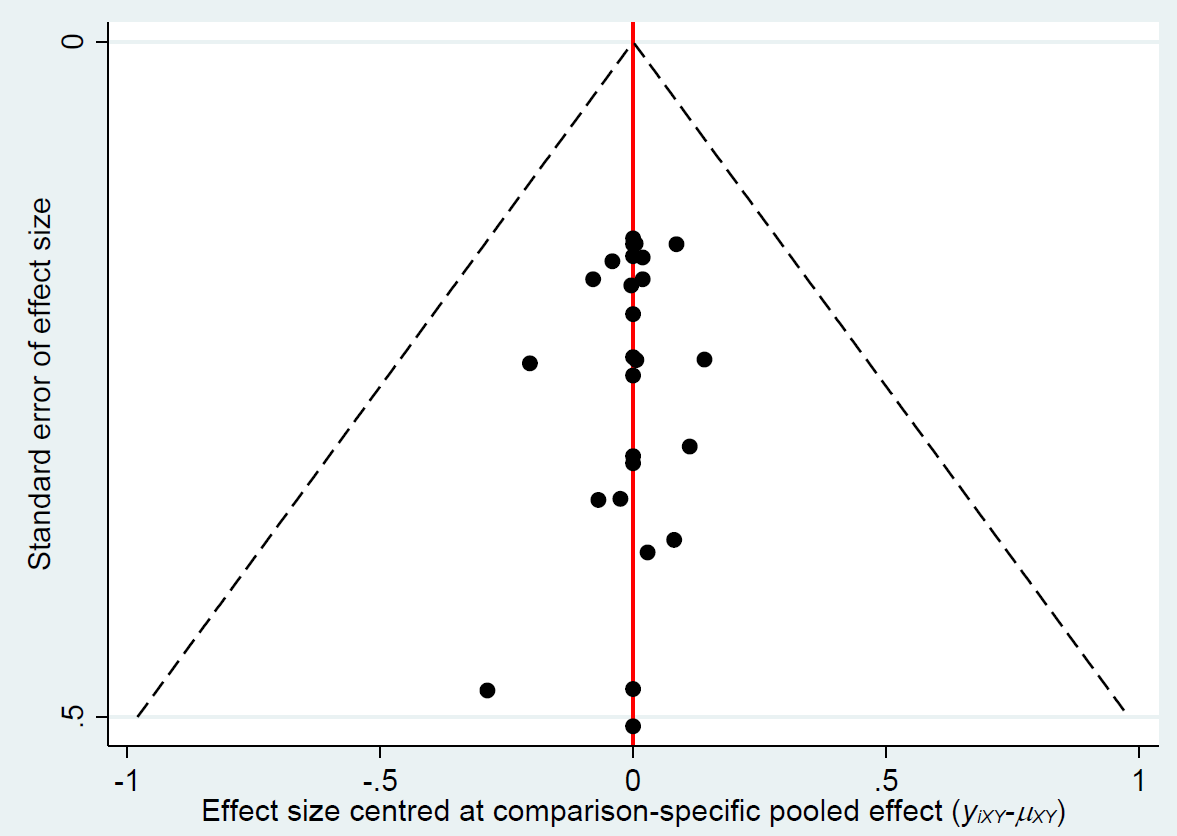


Appendix II Figure 3: The funnel plot in subgroup analysis for the keratometry in the flattest meridian measurement in cataract subjects.


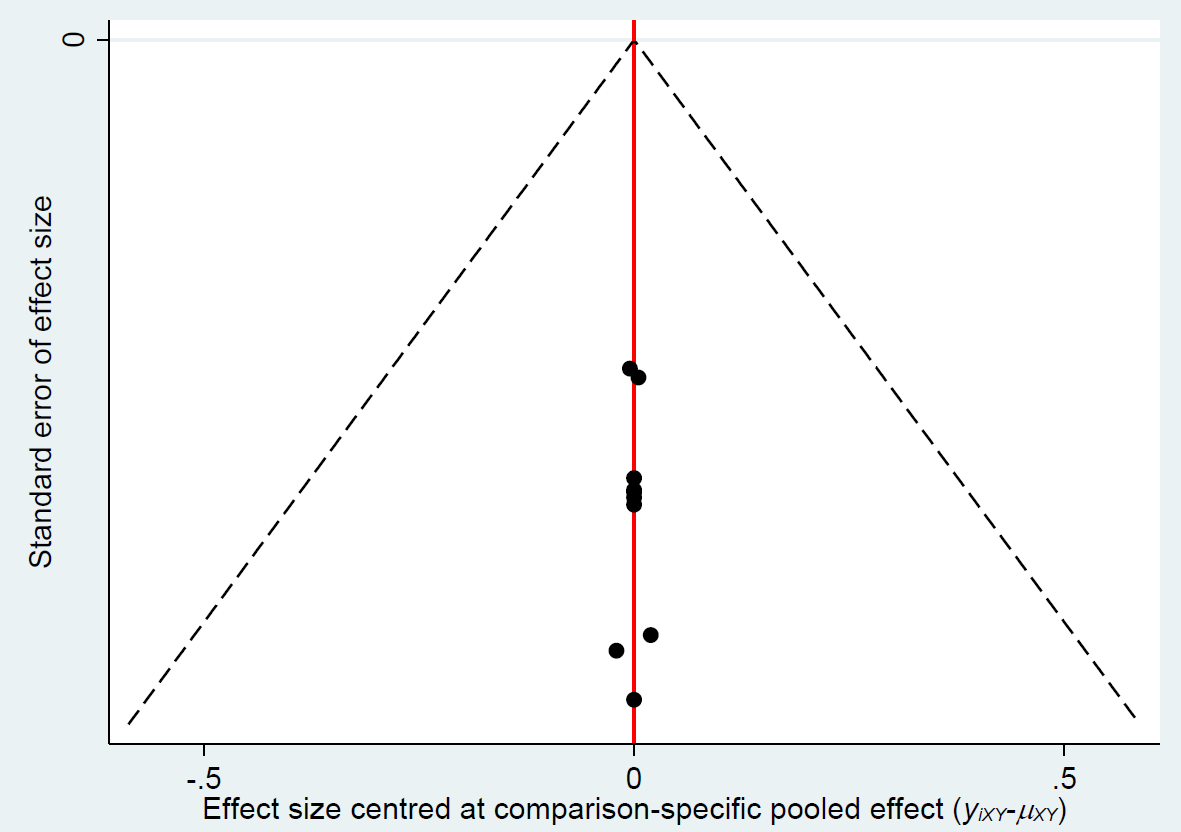


Appendix II Figure 4: The funnel plot in subgroup analysis for the keratometry in the flattest meridian measurement in healthy subjects.


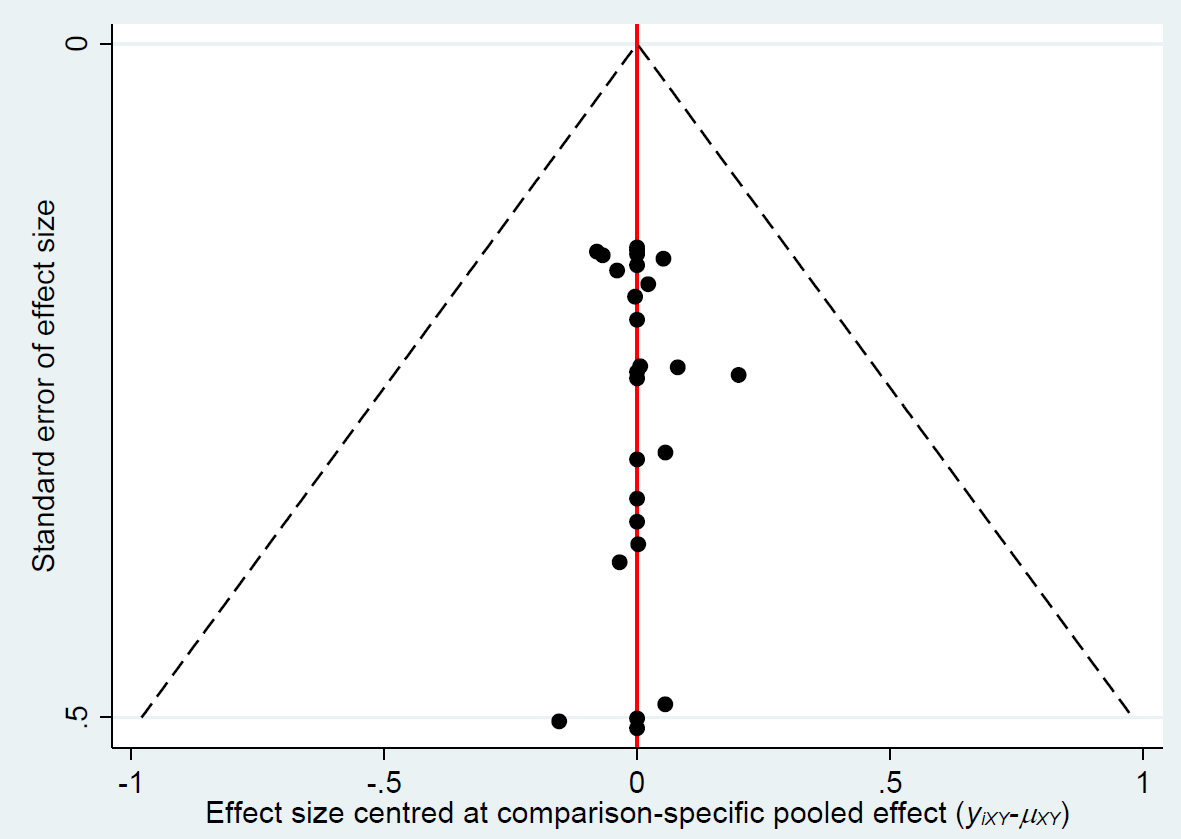


Appendix II Figure 5: The funnel plot in subgroup analysis for the keratometry in the steepest meridian measurement in cataract subjects.


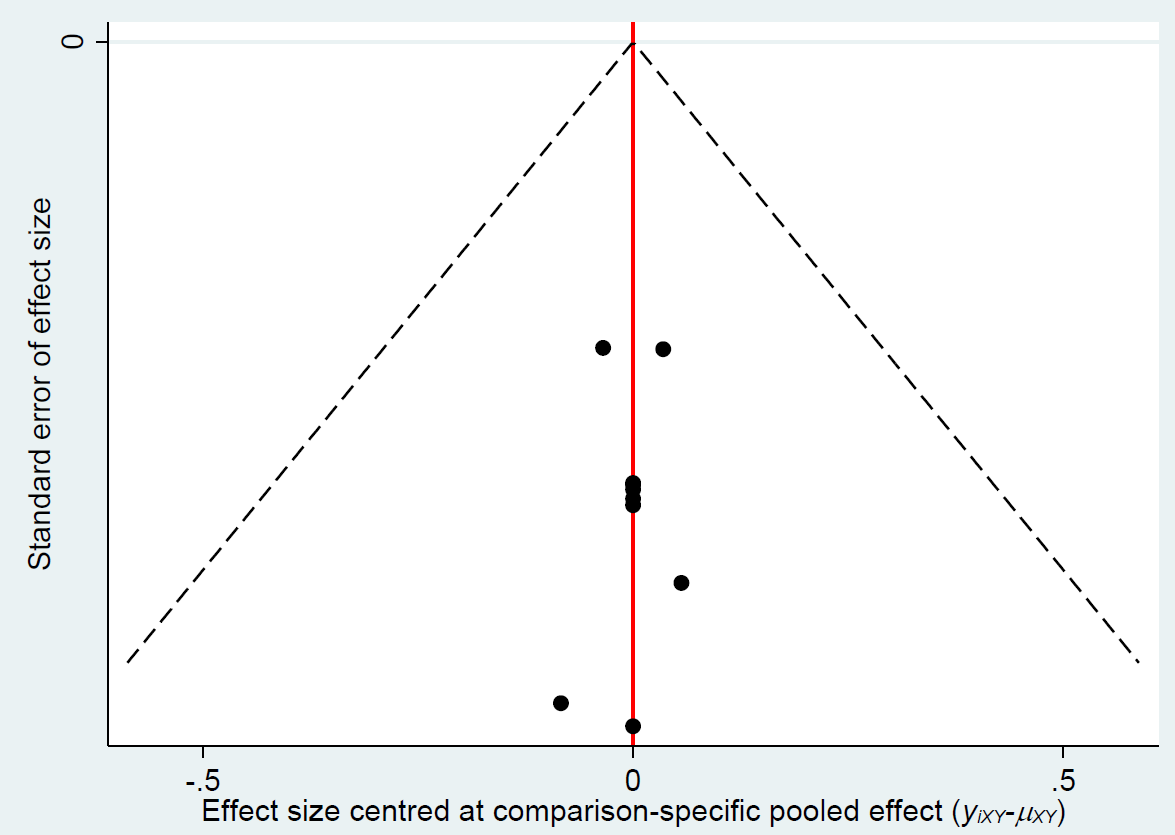


Appendix II Figure 6: The funnel plot in subgroup analysis for the keratometry in the steepest meridian measurement in healthy subjects.


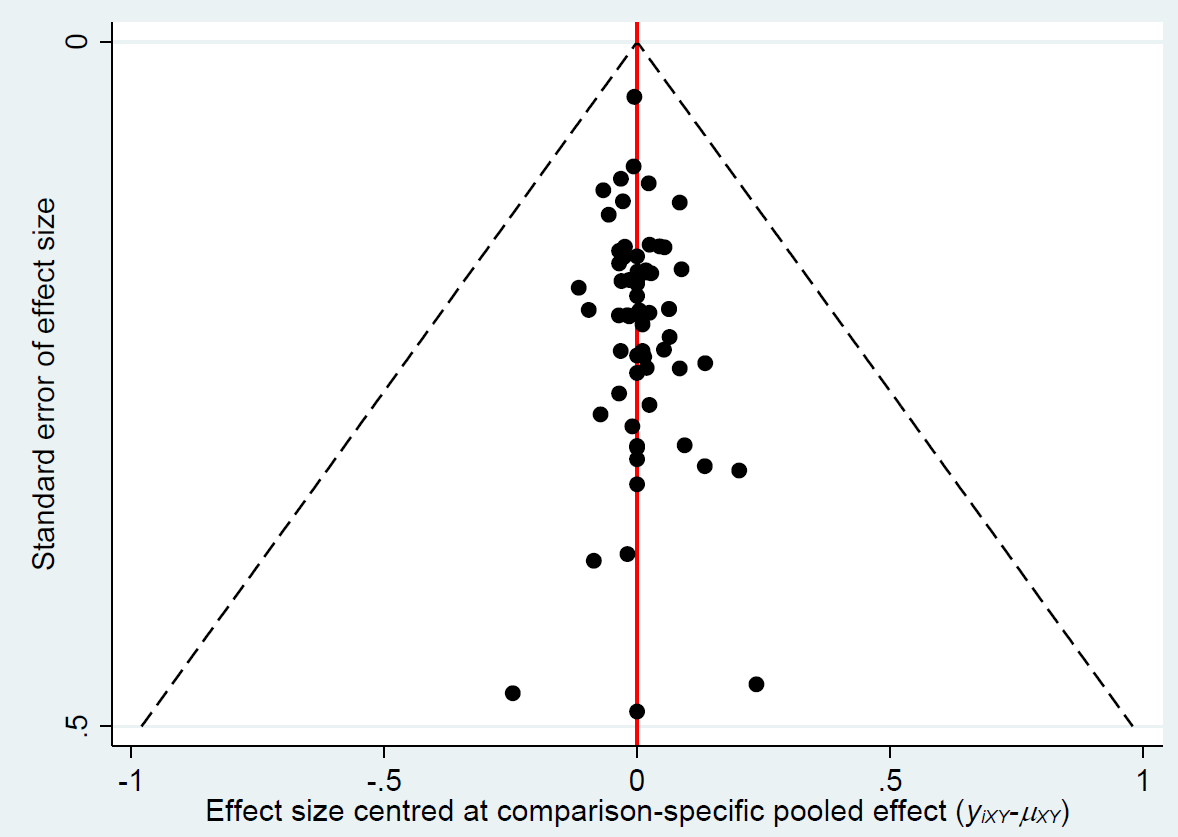


Appendix II Figure 7: The funnel plot in subgroup analysis for the mean keratometry measurement in cataract subjects.


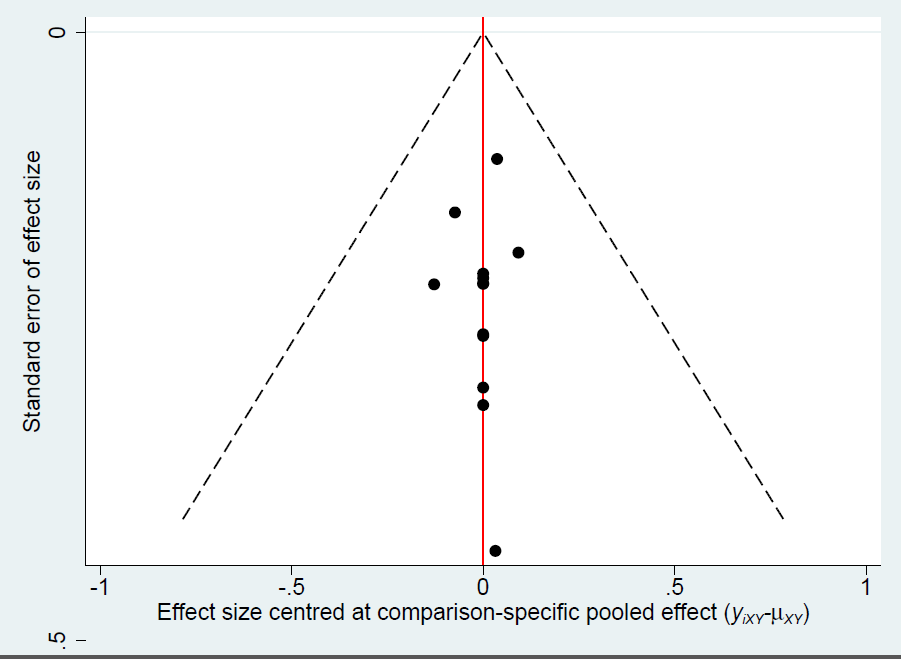


Appendix II Figure 8: The funnel plot in subgroup analysis for the mean keratometry measurement in healthy subjects.


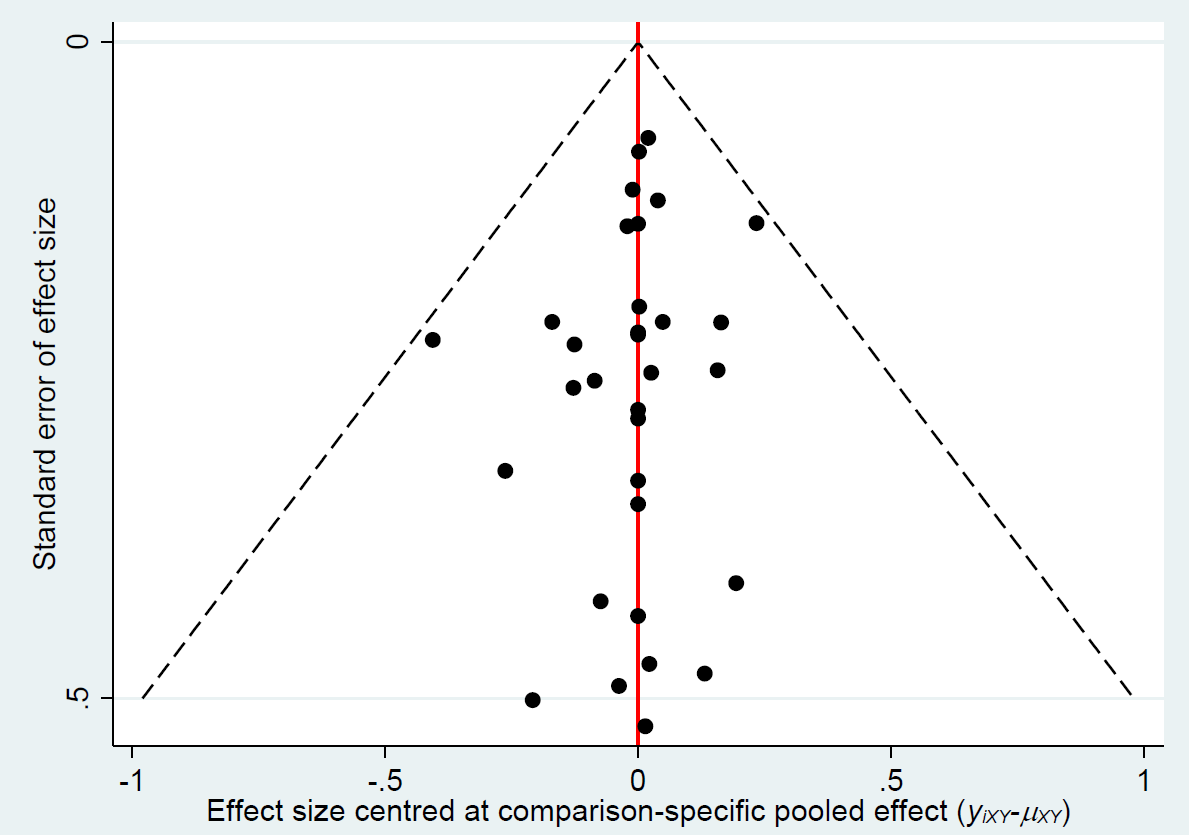


Appendix II Figure 9: The funnel plot in subgroup analysis for the astigmatism measurement in cataract subjects.


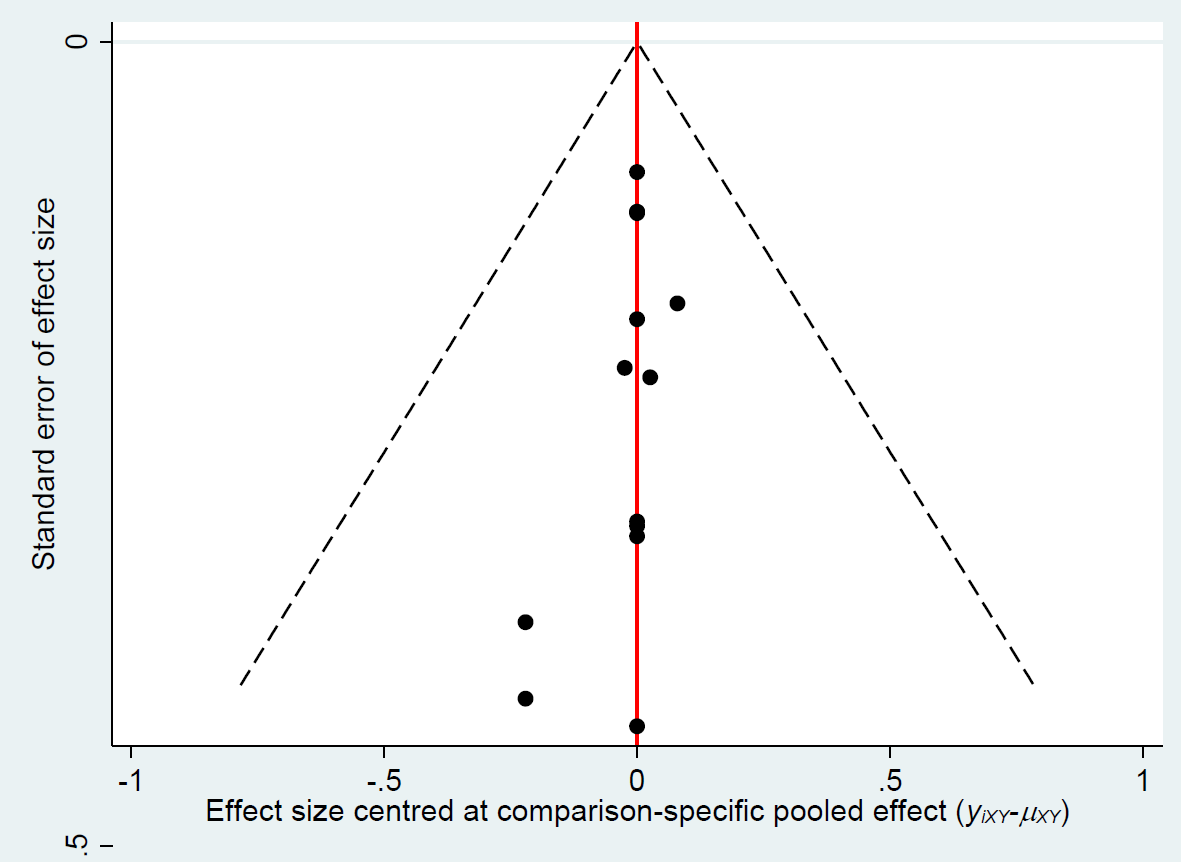


Appendix II Figure 10: The funnel plot in subgroup analysis for the astigmatism measurement in healthy subjects.


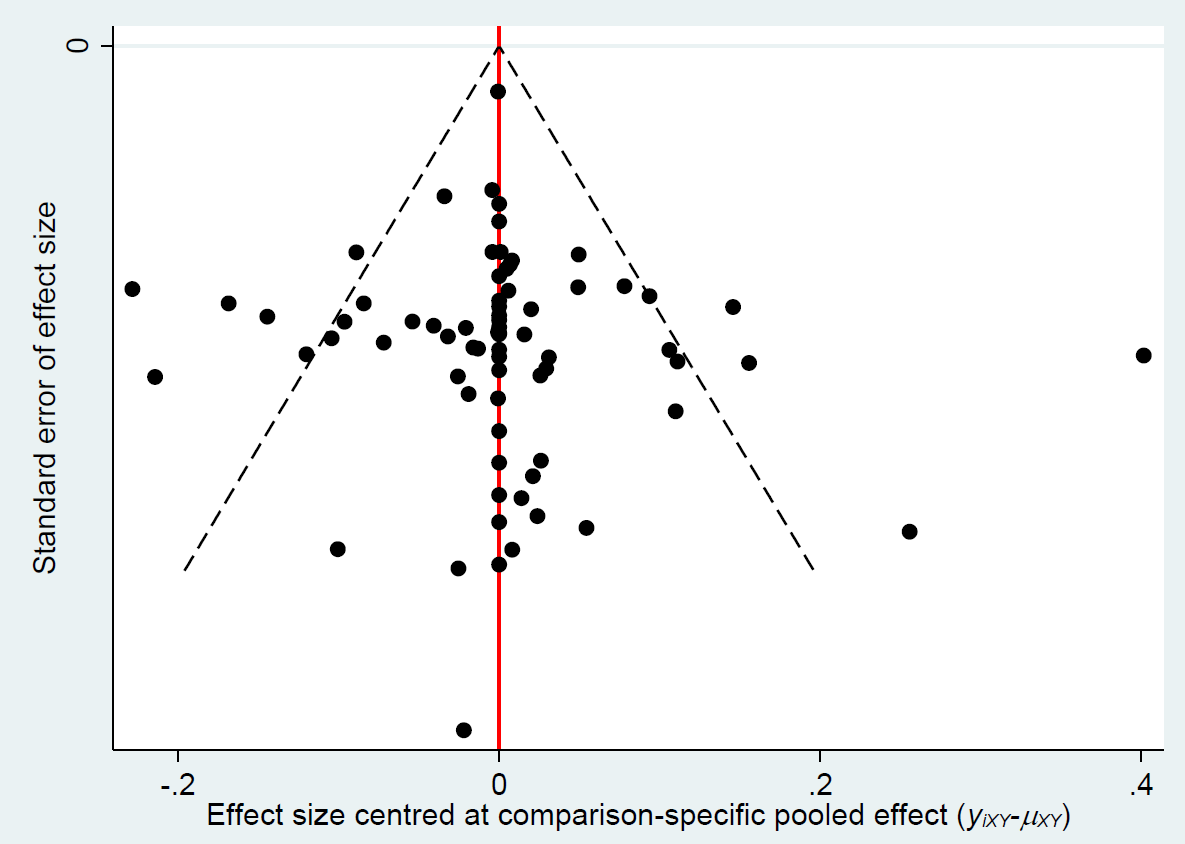


Appendix II Figure 11: The funnel plot in subgroup analysis for the anterior chamber depth measurement in cataract subjects.


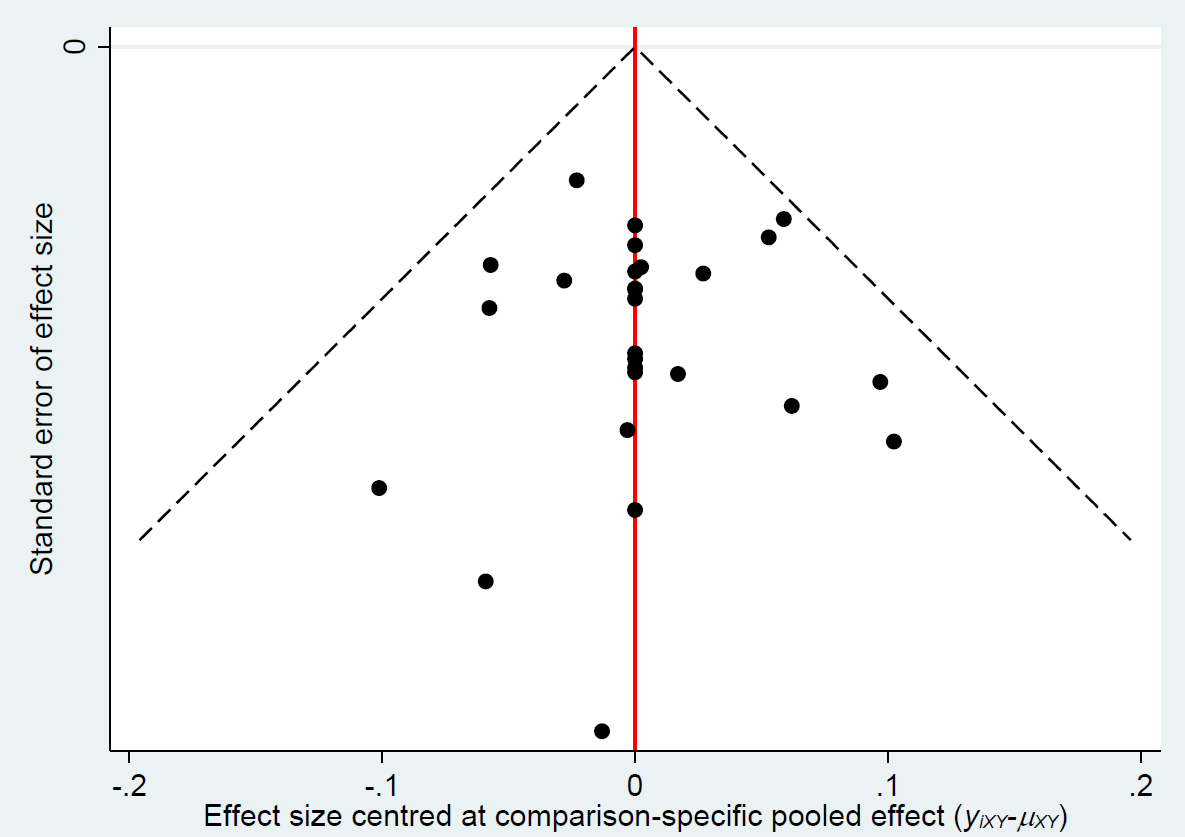


Appendix II Figure 12: The funnel plot in subgroup analysis for the anterior chamber depth measurement in healthy subjects.


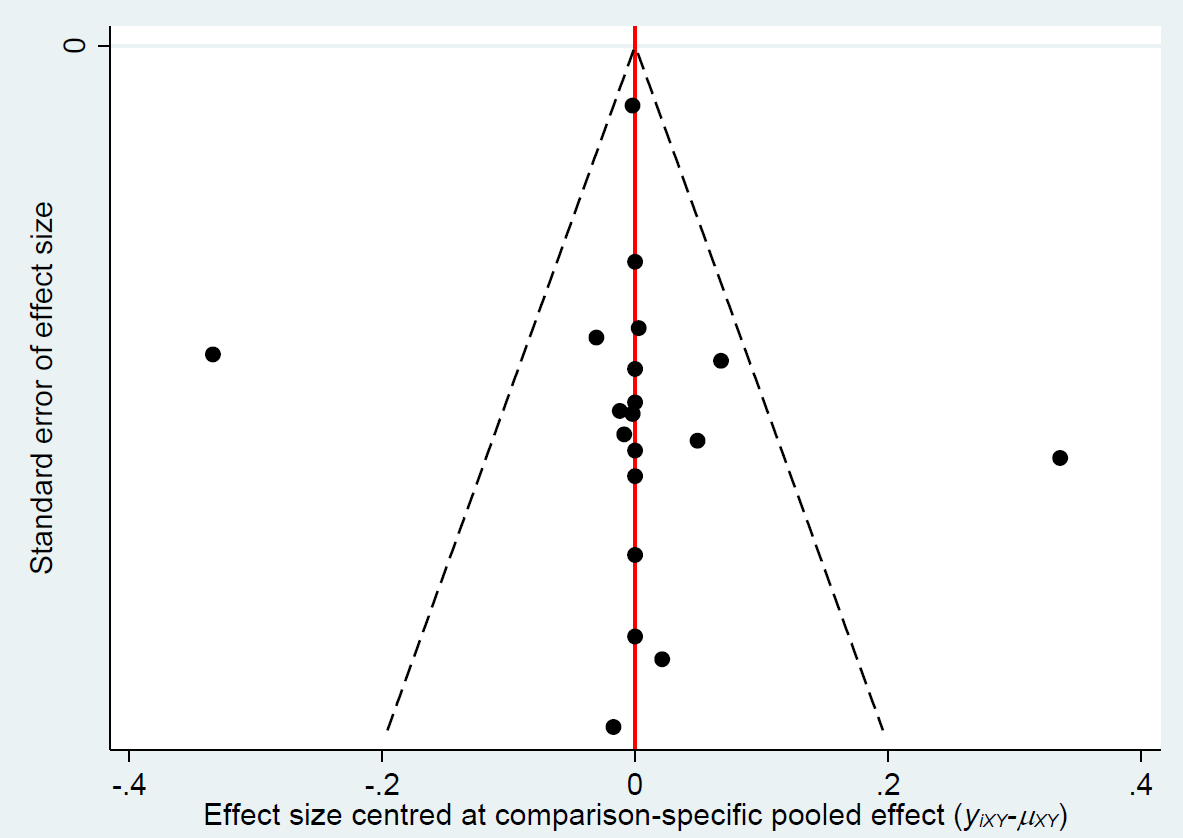


Appendix II Figure 13: The funnel plot in subgroup analysis for the aqueous depth measurement in cataract subjects.


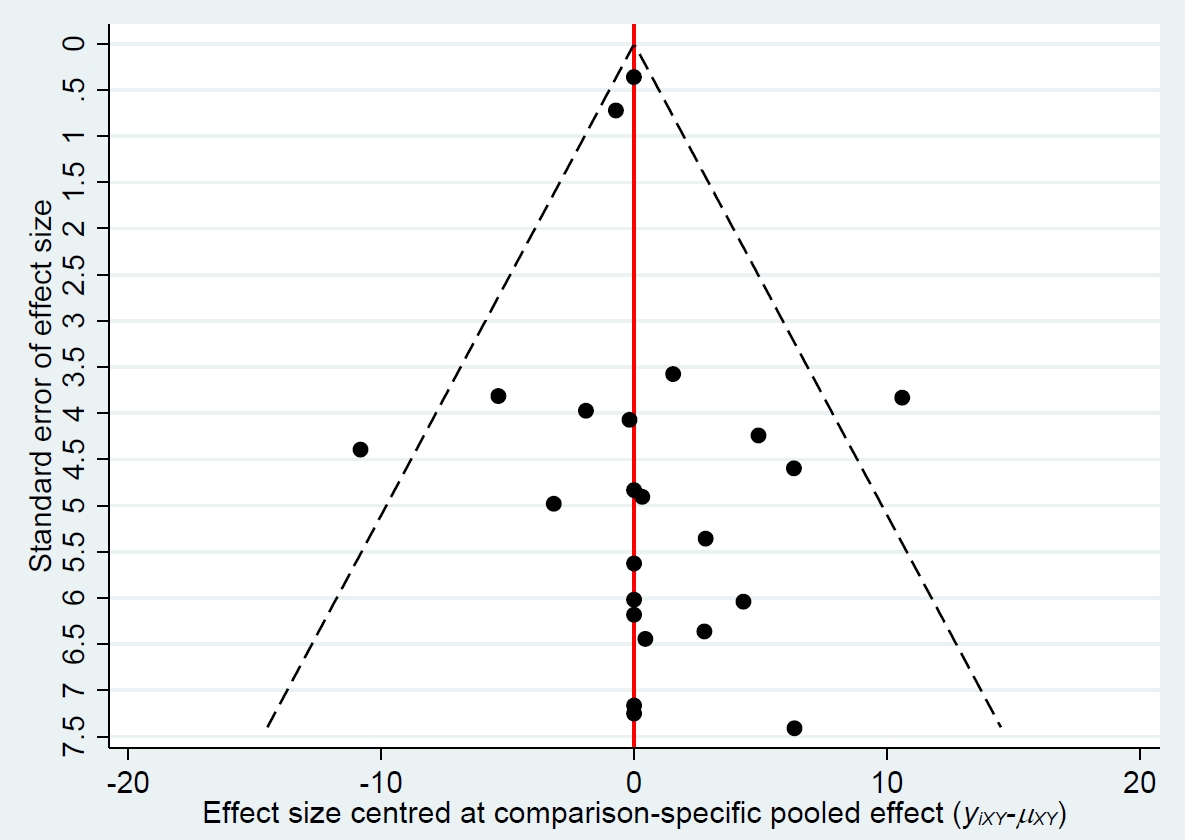


Appendix II Figure 14: The funnel plot in subgroup analysis for the central corneal thickness measurement in cataract subjects.


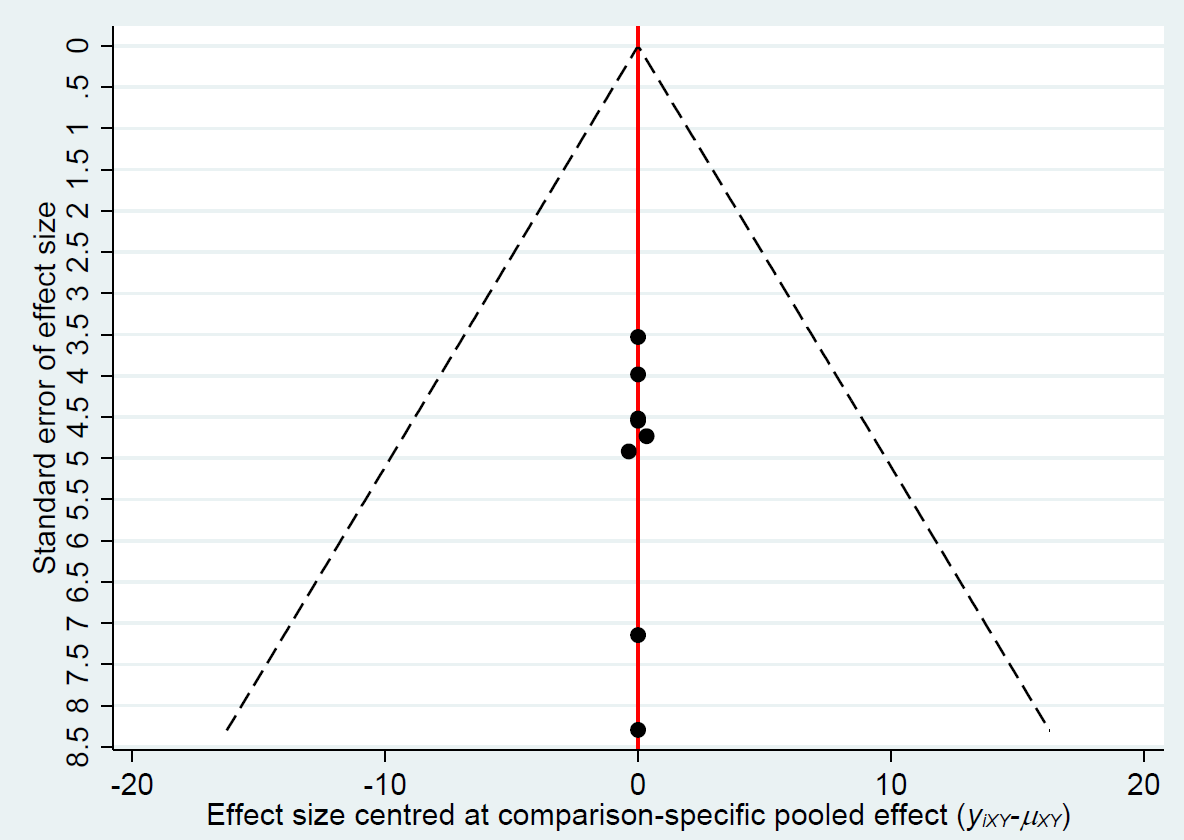


Appendix II Figure 15: The funnel plot in subgroup analysis for the central corneal thickness measurement in healthy subjects.


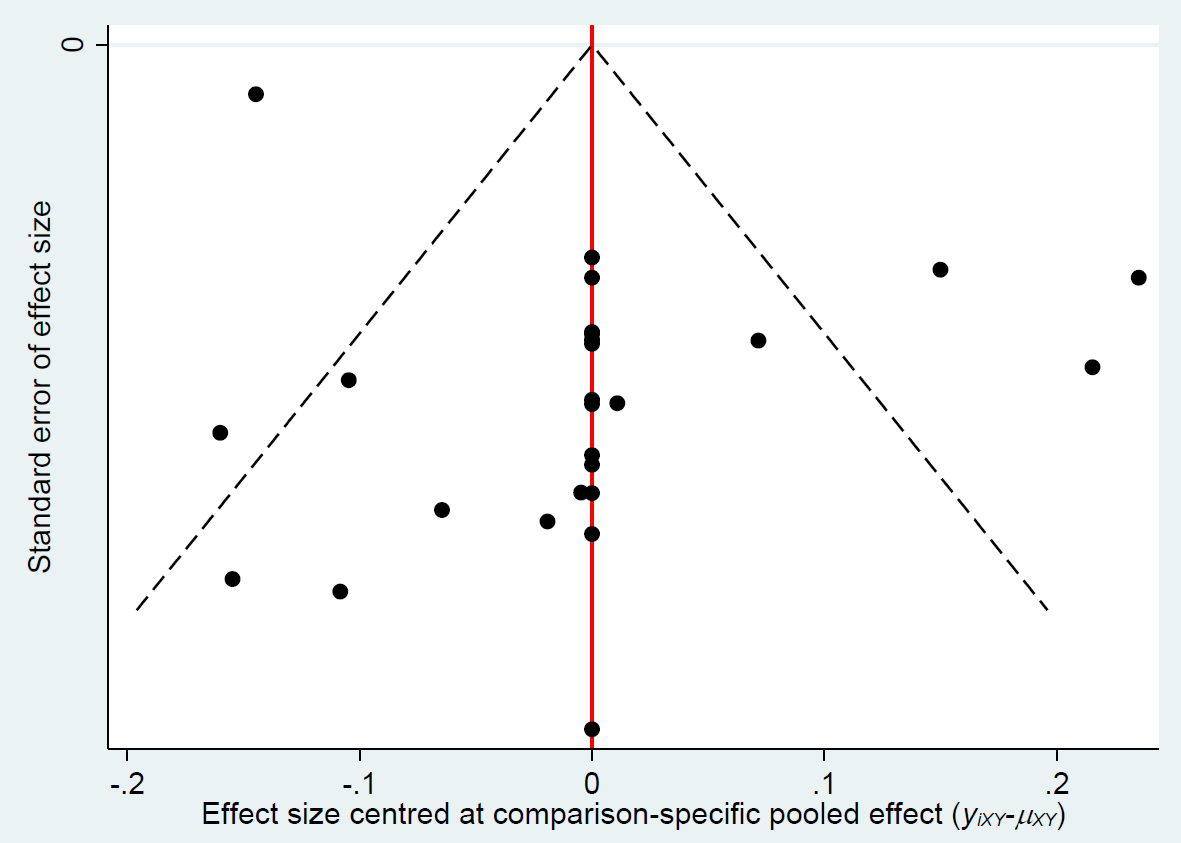


Appendix II Figure 16: The funnel plot in subgroup analysis for the corneal diameter measurement in cataract subjects.


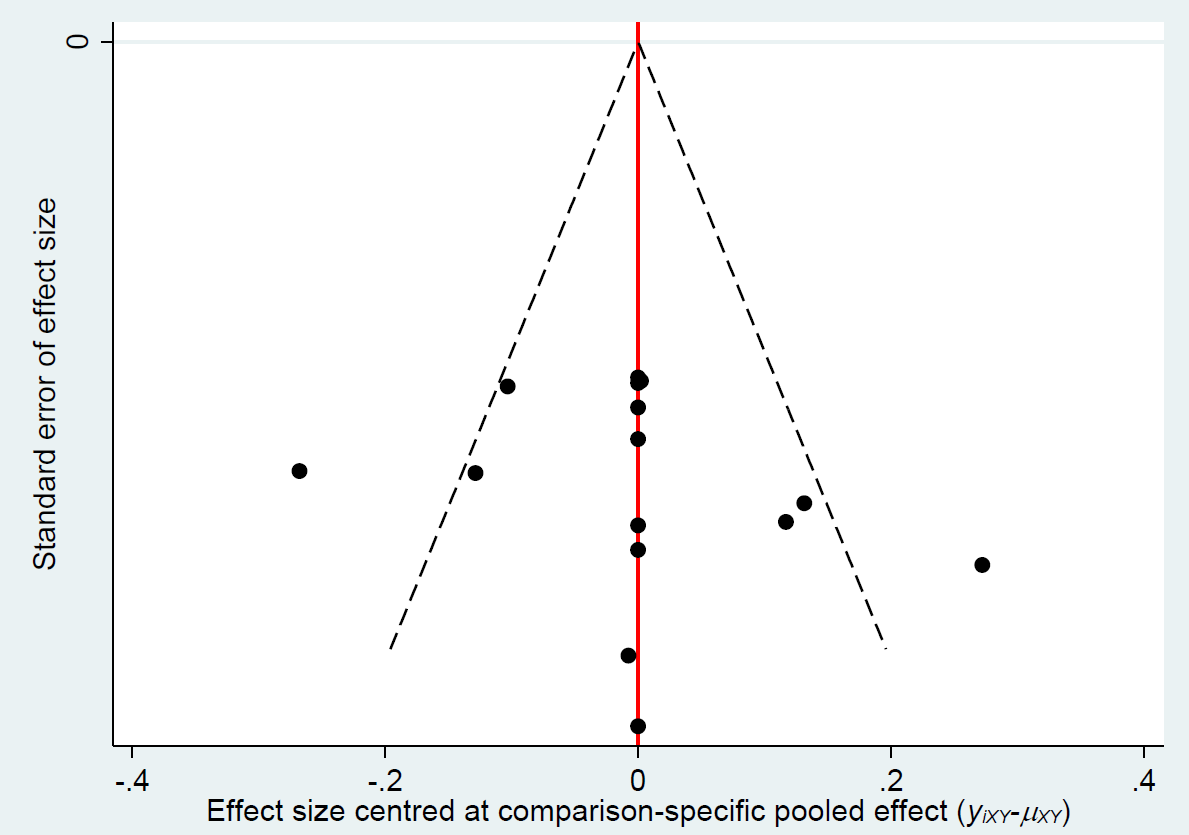


Appendix II Figure 17: The funnel plot in subgroup analysis for the corneal diameter measurement in healthy subjects.


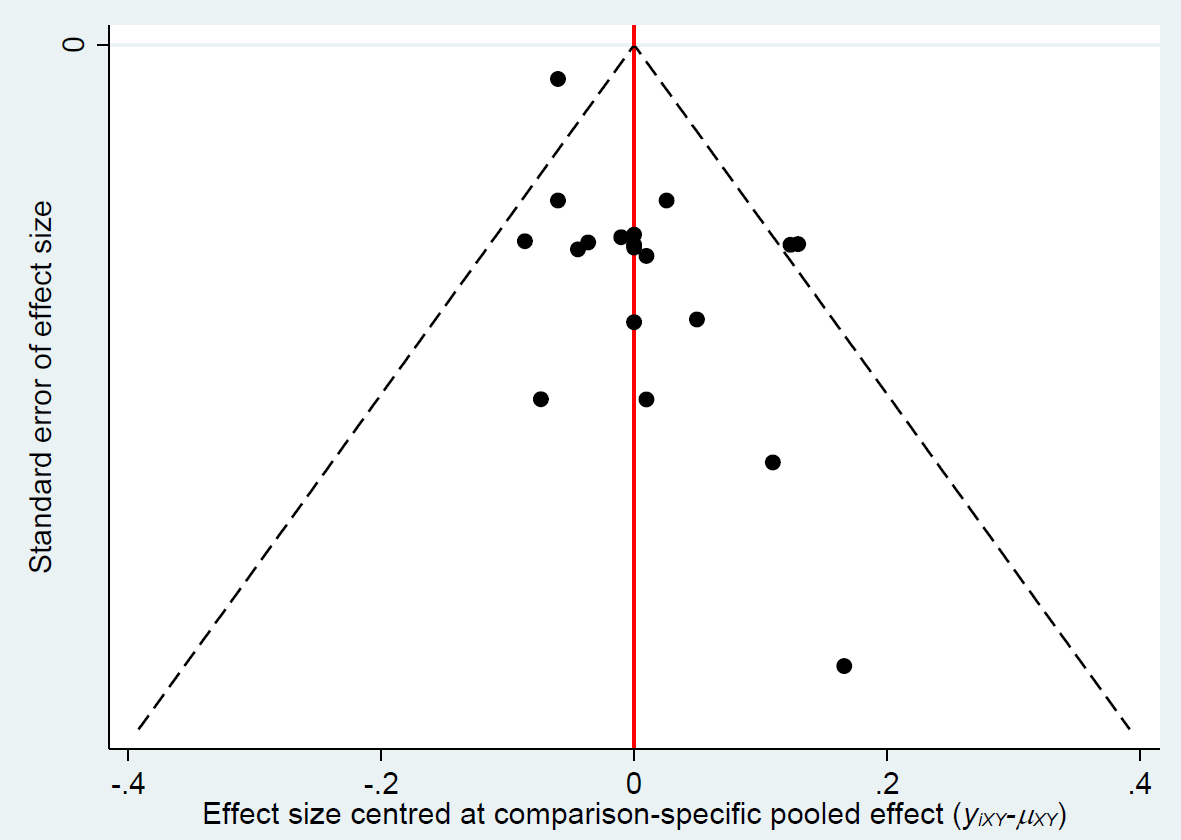


Appendix II Figure 18: The funnel plot in subgroup analysis for the lens thickness measurement in cataract subjects.
